# Lewis acid/NHC dual catalysis for regioselective vicinal decarboxylative carbonylation–acylation of alkenes

**DOI:** 10.1039/d5sc09978a

**Published:** 2026-02-23

**Authors:** Mao-Lin Yang, Xiao-Feng Wu

**Affiliations:** a Leibniz-Institut für Katalyse e.V. Albert-Einstein-Str. 29a 18059 Rostock Germany xiao-feng.wu@catalysis.de; b Dalian National Laboratory for Clean Energy, Dalian Institute of Chemical Physics, Chinese Academy of Sciences 116023 Liaoning China

## Abstract

Vicinal difunctionalization of alkenes offers a powerful route to complex carbon architectures by installing two distinct groups. Herein, we describe a novel Lewis acid–thiazolium NHC catalytic system that enables precise control of a single-electron-transfer (SET) process to achieve CO-incorporated vicinal decarboxylative carbonylation–acylation of alkenes. The reaction is proposed to proceed *via* a concerted SET between the enolate form of the Breslow intermediate and a Lewis acid–activated NHPI ester, followed by CO trapping and regioselective recombination of multiple radical intermediates to install two distinct carbonyl groups. This strategy constitutes a new platform for C1 exchange and enables efficient CO incorporation even with sterically demanding or conformationally rigid alkyl radicals.

## Abstract 2

Herein, we describe a novel Lewis acid–thiazolium NHC catalytic system that enables precise control of a single-electron-transfer (SET) process to achieve CO-incorporated vicinal decarboxylative carbonylation–acylation of alkenes.

## Graphical abstract

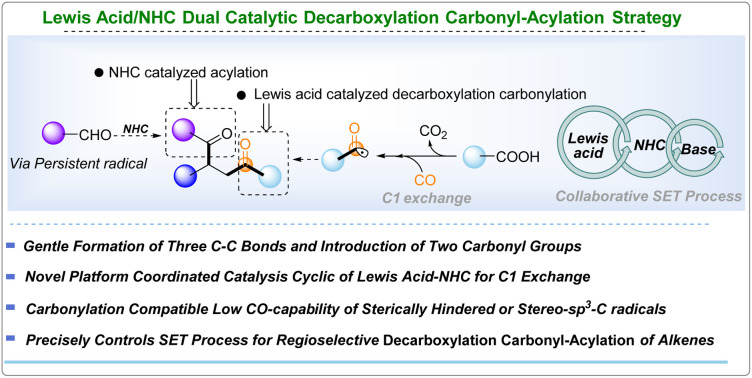

## Introduction

**Fig. 1:**
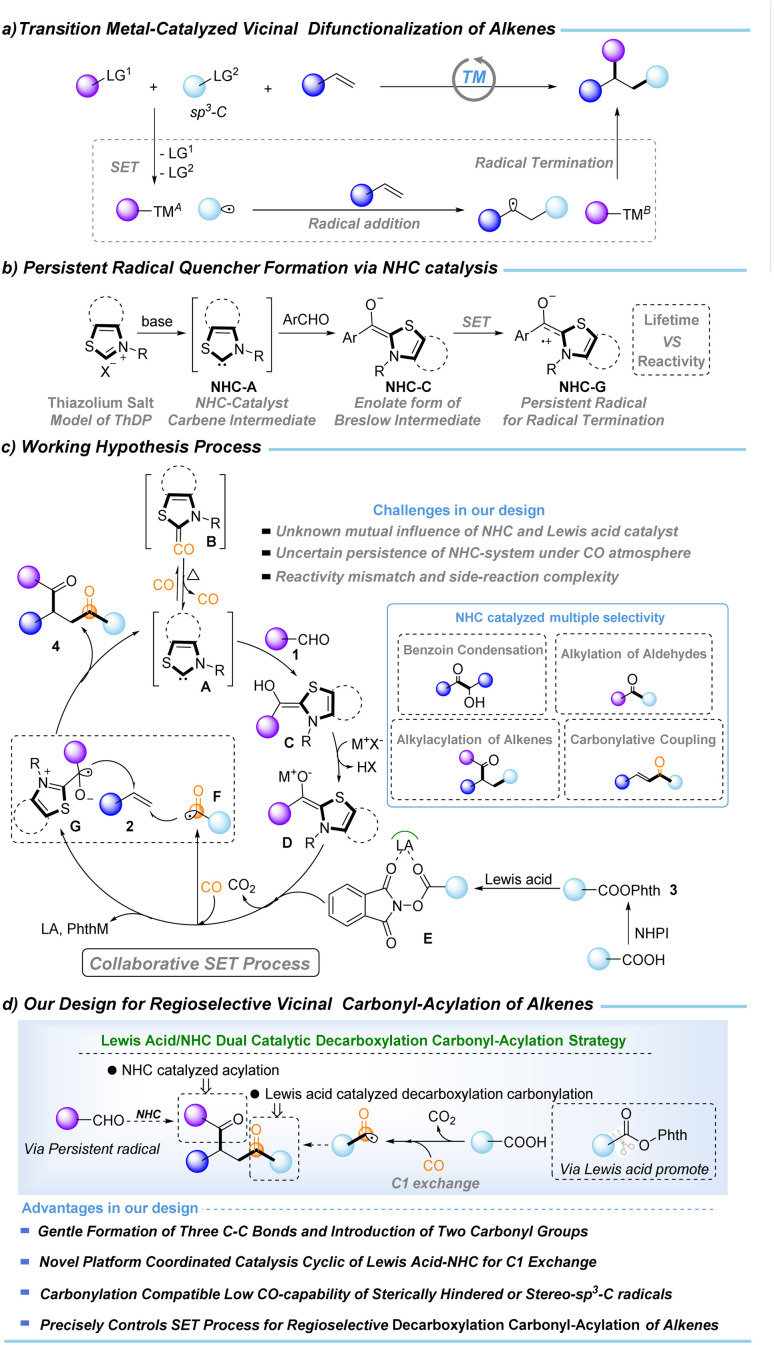
Background of regioselective vicinal decarboxylative carbonylation–acylation of alkenes.

Vicinal difunctionalization of alkenes offers a powerful route to complex carbon architectures by installing two distinct groups.^1–5^ However, transition-metal-catalyzed variants that introduce an sp^3^-alkyl unit remain intrinsically difficult, as the *in situ* generated alkylmetal intermediates readily undergo β–H elimination, isomerization, and other deleterious pathways (Fig. 1a). Radical-relay approaches circumvent these organometallic instabilities,^6–13^ yet multi-radical systems introduce a different challenge: achieving high-fidelity SET-controlled radical matching amid short radical lifetimes and multiple competing coupling channels. Overcoming this challenge is essential for general alkene regioselective vicinal dicarbon-functionalization and its wider synthetic applications.

C1 chemistry has emerged as a frontier in modern carbon functionalization,^14–18^ with C1-exchange methodologies garnering significant attention due to their potential impact in biological and medicinal contexts.^19–23^ Consequently, the development of efficient C1-conversion platforms is highly desirable. Carbon monoxide (CO), as an abundant, highly reactive, and versatile C1 feedstock, represents an ideal surrogate for C1 exchange.^24–30^ In parallel, *N*-heterocyclic carbene (NHC) catalysis has evolved into a powerful and versatile platform for C1 chemistry, enabling diverse carbonyl-based transformations.^31–42^ While two-electron NHC catalysis has been extensively developed, NHC-mediated radical processes have also gained recognition. A canonical biochemical precedent is the thiamine diphosphate (ThDP)-dependent decarboxylation of pyruvate.^43–46^ Analogously, the enolate form of the Breslow intermediate—generated from aryl aldehydes and thiamine analogues under strong base—displays remarkable reducing capability (
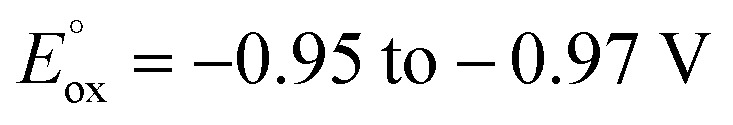
, Fig. 1b).^35,36^ Such potent single-electron-donating ability enables decarboxylative coupling transformations. We envisioned that incorporating a reductive Lewis acid could effectively coordinate the NHC-driven SET process, thereby enhancing the decarboxylation of NHPI esters to release CO_2_ and the corresponding sp^3^-alkyl radicals for CO trapping, to realize the C1-exchange transformation.

Based on the above considerations and our continued interest in C1 chemistry,^47–59^ we designed a new regioselective vicinal decarboxylative carbonylation–acylation of alkenes coordinated by NHC, Lewis acid, and NHPI ester. The mechanistic details of our working hypothesis are shown in Fig. 1c. Condensation of aryl aldehydes 1 with NHC-catalyst A affords the Breslow intermediate C, which is deprotonated by the base (MX) to generate the reducing enolate D. The SET process between D and E produces the persistent radical quencher G (with a suitable reactivity match) and an sp^3^-alkyl radical that rapidly traps CO to deliver the acyl radical F (favoring decarbonylation). Moreover, because D (
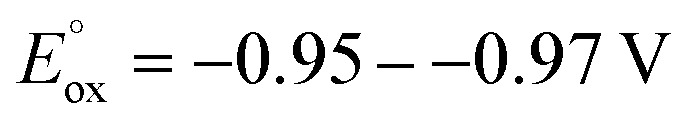
*vs.* SCE in MeCN) may be inefficient at reducing NHPI ester 3 (*E*_red_^0^ < −1.28 V *vs.* SCE in MeCN),^36^ reductive Lewis-acid (*via*E) activation of the phthalimide moiety facilitates the SET event.^60^ This cooperative activation ensures key intermediate G generation and releases sufficient concentration of sp^3^-alkyl radicals for productive CO trapping (ensuring efficient C1 exchange). A further challenge is achieving regioselective radical coupling to deliver product 4. Both the sp^3^-alkyl radical and the acyl F can add to the alkene, generating competing alkylation and acylation pathways. In parallel, the Breslow-derived ketyl radical G can engage in 5-exo cyclization with the alkyl radical, further diverting the reaction. These competing channels significantly impact the selectivity of the transformation. Additional inefficiencies may arise from NHC-mediated benzoin condensation or acylation alone. Moreover, catalyst longevity under high CO pressure is crucial: the strong σ-donating and π-accepting properties of NHCs render them prone to CO coordination and deactivation,^61,62^ requiring conditions that sustain the active carbene species. Thus, the regioselective Lewis acid-NHC catalytic decarboxylative carbonylative acylative coupling of alkenes is a significant challenge in C1 exchange chemistry.

Herein, we report a regioselective Lewis acid and NHC dual catalytic decarboxylative carbonylation–acylation strategy (LN-DCA strategy). This strategy capitalizes on Lewis-acid-enabled decarboxylative carbonylation and NHC-mediated acylation, thereby allowing secondary-alkyl (*via* cyclic, linear, or stereorigid radicals) and tertiary-alkyl (*via* bulky or stereorigid radicals) carboxylic acids to undergo selective decarboxylative carbonylation–acylation. As a result, two different acyl groups can be installed across C=C bonds with complete regioselectivity, furnishing substituted 1,4-dicarbonyl compounds.

## Results and discussion

After judicious screening of the reaction conditions based on our working hypothesis, to realize our design of the CO-inclusive four-component LN-DCA strategy for accessing substituted 1,4-dicarbonyl compounds, we initially employed benzaldehyde 1a, styrene 2a, and NHPI ester 3a as model substrates to find ideal conditions, as shown in Table 1. In our CO-inclusive multiple radical system, two distinct acyl groups were introduced at the α carbon (derived from CO trapping of the alkyl radical) and the β carbon (derived from benzaldehyde) of styrene with complete regioselectivity. We found that the reaction of 1a (0.4 mmol), 2a (0.8 mmol), and 3a (0.2 mmol) in the presence of an *N*-2,6-diisopropylphenyl-substituted six-membered ring-fused thiazolium salt (NHC-2, 10 mol%) as the NHC precursor and Cs_2_CO_3_ (15 mol%) in DMSO (3 mL) under a CO atmosphere (50 bar) at 80 °C afforded the target 1,4-dicarbonyl product 4a in 80% yield, with only trace amounts of the no-carbonylation byproduct 4a′, a little NHC-catalyzed benzoin condensation byproduct 4a″ and two-component coupling byproducts 4a‴ (Table 1, entry 1). The steric and electronic natures of the NHC catalyst had a marked influence on the reactivity (Table 1, entries 2–6). First, we focused our investigation of NHC catalysts on commonly used NHC precursor salts featuring N,S-containing backbones, two N-backbones, and tri-nitrogen-containing frameworks. We found that NHC precursors (NHC-2) bearing an N,S-based core, particularly those incorporating a fused seven-membered ring, were electronically favorable for the desired transformation. In addition, *N*-aryl substituents bearing 2,6-diisopropylphenyl groups provided the most favorable steric effects. Reaction performance was highly sensitive to the choice of base, solvent, temperature, and CO pressure (Table 1, entries 7–16). Cs_2_CO_3_ proved to be the optimal base, while alternative inorganic bases or strong organic bases led to diminished efficiency (Table 1, entry 7). DMSO was identified as the optimal solvent (Table 1, entry 8), and 80 °C effectively suppressed competing aldehyde self-condensation and two-component coupling pathways (Table 1, entries 9 and 10). Efficient decarboxylation and CO capture further required ZnCl_2_ as the Lewis acid and a CO pressure of 50 bar (Table 1, entries 9–16).

**Table 1:** Optimization of the reaction conditions^a^

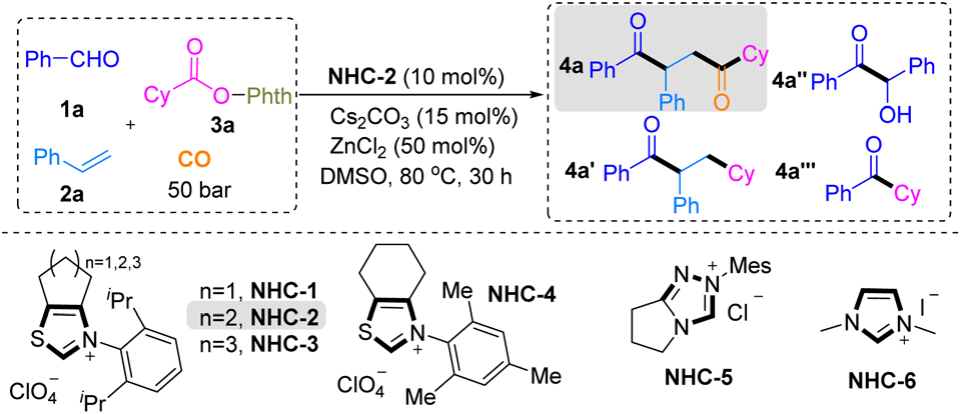		
Entry	Variations	Yield of 4a-4a′-4a″-4a‴ (%)
1	None	80 (75)-trace-5-trace
2	NHC-1 instead of NHC-2	N.D.-N.D.-N.D.-N.D.
3	NHC-3 instead of NHC-2	32- Trace-10-59
4	NHC-4 instead of NHC-2	16- Trace-22-20
5	NHC-5 instead of NHC-2	10- Trace-38-18
6	NHC-6 instead of NHC-2	N.D.-N.D.-trace-N.D.
7	K_2_CO_3_ (or DBU, KO^*t*^Bu) instead of Cs_2_CO_3_	55-20-Trace-10 (or 38-15-trace-10, N.D.– N.D.-20- N.D.)
8	THF, DCE, toluene, MeCN instead of DMSO	N.D. for 4a
9	60 °C instead of 80 °C	20-Trace-20-43
10	100 °C instead of 80 °C	46-Trace-10-trace
11	No Lewis acid	60-Trace-15-10
12	Zn(OAc)_2_ instead of ZnCl_2_	56-Trace-trace-5
13	ZnF_2_ instead of ZnCl_2_	70-Trace-8-11
14	ZnBr_2_ instead of ZnCl_2_	50-Trace-10-trace
15	40 bar instead of 50 bar CO	61-10-20-5
16	60 bar instead of 50 bar CO	70-Trace-trace-trace

a Reaction conditions: 1a (0.2 mmol), 2a (0.4 mmol), 3a (0.1 mmol), and DMSO (1.5 mL). Determined by GC with hexadecane as the internal standard. Isolated yield is shown in parentheses.

**Scheme 1:**
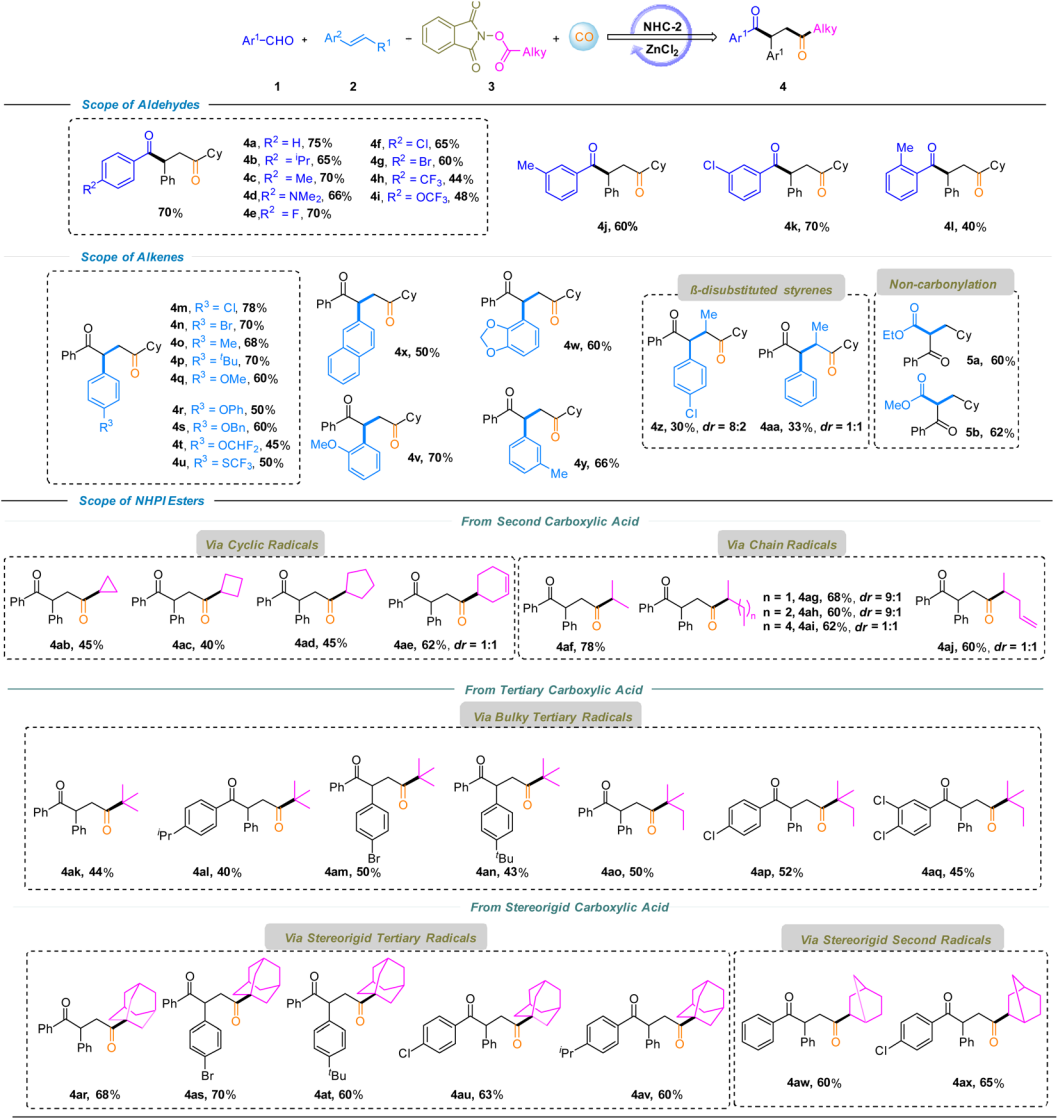
Substrate scope. Reaction conditions: 1 (0.4 mmol), 2 (0.8 mmol), 3 (0.2 mmol), DMSO (3.0 mL), CO (50 bar), ZnCl_2_ (50 mol%), NHC-2 (10 mol%), and Cs_2_CO_3_ (15 mol%), at 80 °C for 30 h. Isolated yields.

The generality of the Lewis acid–NHC–catalyzed four-component regioselective decarboxylative carbonylation–acylation of alkenes was evaluated by exploring different substrates (Scheme 1). A series of substituted aryl aldehydes were tested at the first stage, and the carbonylated products 1,4-diketones 4a–4l, were obtained in moderate to good yields (40–75%). Among them, *para*-substituted benzaldehydes exhibited good reactivity in the transformation, affording the corresponding products 4a–4g (*p*-H, ^*i*^Pr, Me, NMe_2_, F, Cl, and Br) in good yields ranging from 60% to 75%, except for 4h (*p*-CF_3_, 44%) and 4i (*p*-OCF_3_, 48%). And *meta*-substituted benzaldehydes also performed well under the reaction conditions, delivering products 4j (*m*-Me) and 4k (*m*-Cl) in 60% and 70% yields, respectively. However, the *ortho*-substituted substrate showed a lower conversion rate due to steric hindrance, yielding the target product 4l in only 40% yield. In the cases of aliphatic aldehydes, no desired products were formed which might be due to the increased difficulty in the corresponding Breslow intermediate formation. The bottom panel summarizes the results of the reactions of various alkenes under this system. *Para*-Substituted styrenes bearing electron-withdrawing and electron-donating groups (Cl, Br, Me, ^*t*^Bu, OMe, OPh, OBn, OCHF_2_, and SCF_3_) furnished the corresponding products 4m–4u in 45–78% yields. *Ortho*- and *meta*-Substituted styrenes were also tolerated, affording products 4v (*o*-OMe) and 4y (*m*-Me) in 70% and 66% yields, respectively. Naphthyl and heteroaryl-substituted alkenes were also compatible, delivering products 4x and 4w in moderate yields (50–60%). Notably, α-methyl-substituted styrenes underwent transformation to afford 4z (30%) and 4aa (33%) despite increased steric hindrance at the α-position. Michael acceptors such as acrylates exclusively afforded non-carbonylated products (5a and 5b). However, the reaction failed with aliphatic alkenes and no desired product was detected. Furthermore, a range of secondary and tertiary alkyl carboxylic acid-derived NHPI esters were evaluated under the LN-DCA strategy. Secondary alkyl acids were competent substrates, delivering the desired products *via* cyclic radicals (4ab–4ae) or chain radicals (4af–4aj) in 40–78% yields.

Sterically hindered tertiary alkyl radicals are generally reluctant to undergo carbonylation due to severe steric congestion, weak and reversible radical–CO interactions, and rapid competition from noncarbonylative pathways—a longstanding challenge our group have sought to address. Notably, under this LN-DCA strategy, bulky tertiary alkyl acids undergo decarboxylation to generate congested radicals that capture CO and engage in regioselective carbonylation–acylation, delivering products 4ak–4aq in 40–52% yields. Encouraged by this reactivity, conformationally constrained substrates such as adamantane and bicyclo[2.2.1]heptane-2-carboxylic acids were successfully transformed into densely functionalized diketones 4ar–4av (40–52%) and 4aw–4ax (60–65%). Overcoming the intrinsic resistance of rigid tertiary radicals to CO capture expands the structural space of radical carbonylation and highlights the ability of the LN-DCA strategy to precisely control radical reactivity under sterically demanding conditions.

**Scheme 2:**
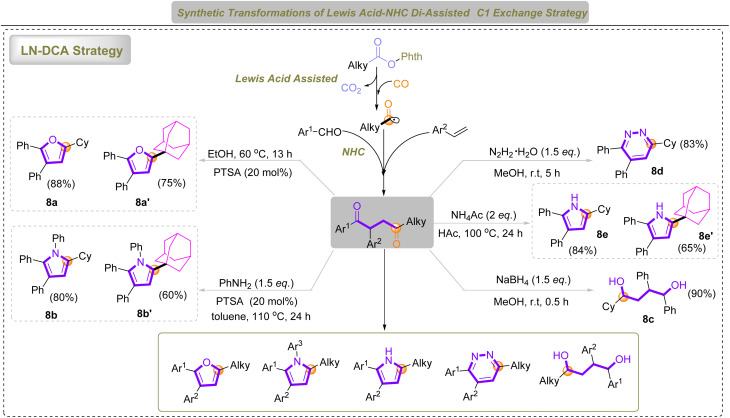
The application of the LN-DCA strategy in heterocycle synthesis and heterocycle–adamantane coupling.

Further transformations using our LN-DCA strategy afforded 1,4-diketones *via* C1 exchange, which could be efficiently converted into a broad array of multi-substituted furans, pyrroles, pyridazines, and 1,4-diols under mild conditions (Scheme 2). The controlled generation of radicals also enables selective C–C bond formation at the sterically congested bridgehead positions of adamantane, facilitating direct installation of heterocyclic motifs and circumventing the limitations of traditional C–H activation. These results demonstrate new insights into CO-mediated C1 exchange and highlight the broad applicability of the LN-DCA system in constructing pharmacologically^63–70^ relevant stereomolecules and rigid, three-dimensional heterocycles.

**Scheme 3:**
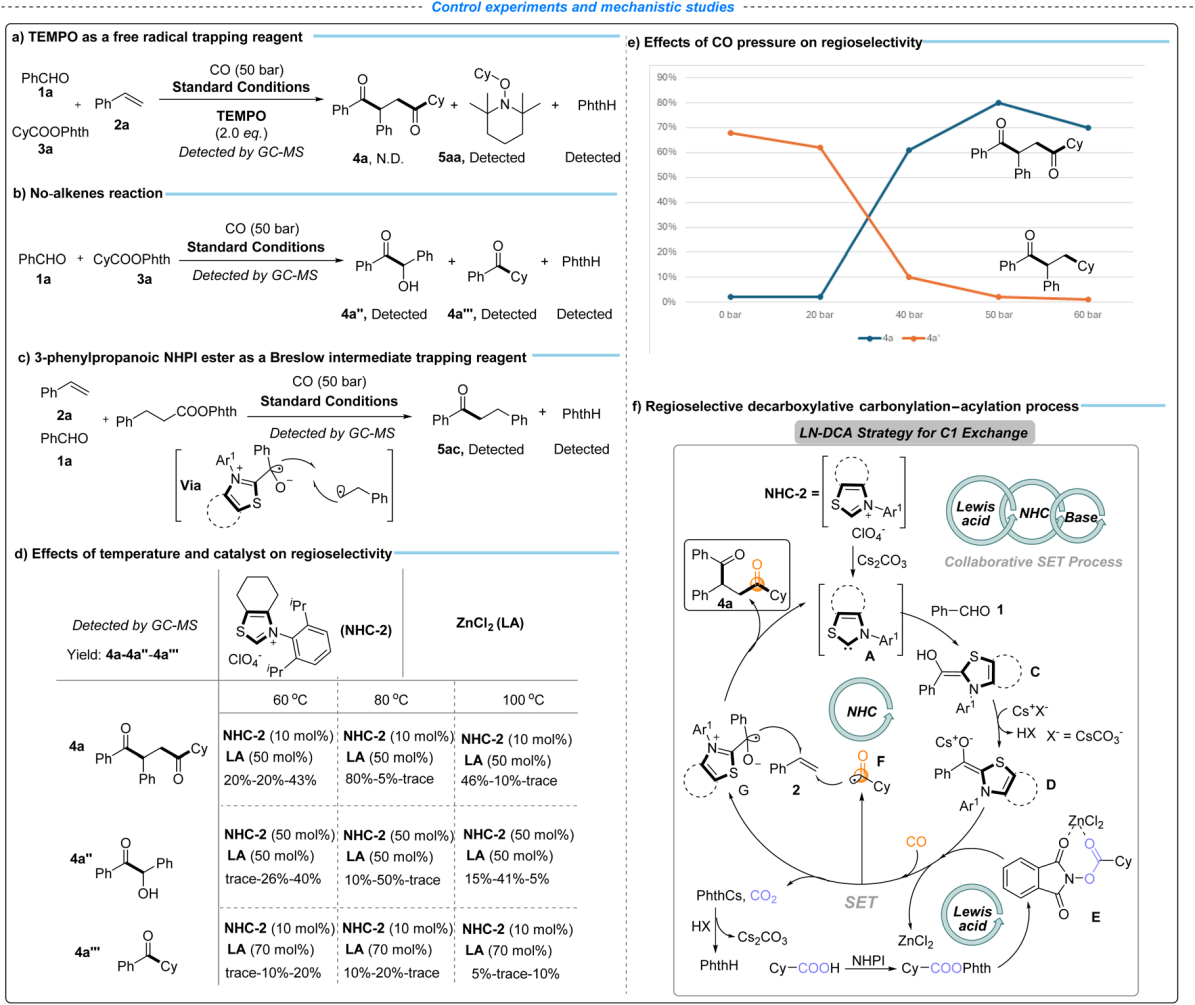
Radical capture experiments and exploration of influencing factors of the catalytic system. 1a (0.2 mmol), 2a (0.4 mmol), and 3a (0.1 mmol). (f) CO, DMSO (1.5 mL), NHC-2 (10 mol%), ZnCl_2_ (50 mol%), 80 °C, and 30 h. Determined by GC with hexadecane as the internal standard.

To further elucidate the reaction mechanism, a series of control experiments were conducted (Scheme 3, eqn (a)–(c)). First, the addition of 2,2,6,6-tetramethylpiperidin-1-oxyl (TEMPO) as a radical scavenger completely suppressed the formation of product 4a under standard conditions (Scheme 3, eqn (a)). Instead, cyclohexyl – TEMPO adduct 5aa and PhthH were detected by GC-MS, supporting the involvement of alkyl radical intermediates. Next, a control experiment without alkenes was performed (Scheme 3b), with the benzoin condensation (4a″) and aldehyde alkylation (4a‴) detected by GC-MS analysis as the main by-products. In addition, treatment of the reaction with 3-phenylpropanoic NHPI ester, employed to probe the involvement of the Breslow intermediate, led to the formation of adduct 5ac (Scheme 3, eqn (c)). Collectively, these results support a radical-based single-electron-transfer (SET) pathway, in which the NHC-derived Breslow intermediate plays a key role in mediating the decarboxylative carbonylation–acylation process.

To further clarify the origin of regioselectivity in the LN-DCA strategy, a series of control experiments were conducted to evaluate the key reaction parameters, including the NHC catalyst loading, ZnCl_2_ concentration, temperature, and CO pressure (Scheme 3, eqn (d) and (e)). As shown in Scheme 3d, lower temperature (60 °C) favors NHC-catalyzed benzoin condensation and aldehyde alkylation, whereas elevated temperature (100 °C) suppresses both the desired transformation and competing NHC-catalyzed processes, underscoring the importance of an optimal temperature to maintain catalyst activity while minimizing side reactions. Excess NHC-2 led to increased formation of byproducts arising from benzoin condensation (4a″) and aldehyde alkylation (4a‴), thereby diminishing the yield of 4a, whereas excessive ZnCl_2_ broadly inhibited NHC catalysis, likely due to strong coordination with NHC-A that disrupts the catalytic cycle. The competition between carbonylative and non-carbonylative pathways was further examined by varying CO pressure (Scheme 3, eqn (e)). Low CO pressure favored the formation of the non-carbonylated product 4a′, while increasing CO pressure suppressed this pathway without significantly enhancing the yield of 4a, from 0 to 20 bar. Notably, further elevation of CO pressure to 60 bar resulted in a decreased yield of 4a, presumably due to CO coordination attenuating the catalytic activity of NHC-A. Collectively, these results highlight the importance of precisely balancing reaction parameters to preserve catalyst activity and enable effective cooperation between NHC and Lewis acid catalytic cycles, thereby achieving regioselective decarboxylative carbonylation–acylation.

Based on these experimental results and relevant literature precedents,^35–39^ we propose a plausible catalytic cycle as depicted in Scheme 3, eqn (f), which is essentially consistent with our original reaction design (Fig. 1c). Benzaldehyde 1a reacts with NHC-A (generated from NHC-2) to form the Breslow intermediate C, which is deprotonated by Cs_2_CO_3_ to afford the corresponding reducing enolate D. The SET process between D and the activated NHPI ester E (derived from cyclohexyl carboxylic acid in the presence of ZnCl_2_) generates the oxidized Breslow intermediate G, liberates ZnCl_2_ for subsequent catalytic turnover, and produces a cyclohexyl radical.^71,72^ The resulting cyclohexyl free radical rapidly traps CO to form the acyl radical F. The decarboxylation process releases Phth-Cs, which participates in the Cs_2_CO_3_ catalytic cycle. Subsequent regioselective vicinal carbonylation–acylation involving G, F, and styrene delivers the desired product 4a and regenerates NHC-A, thereby completing the catalytic cycle.

## Conclusions

In summary, we have developed a novel Lewis acid/thiazolium NHC dual catalytic CO-inclusive vicinal decarboxylative carbonylation–acylation strategy for alkenes, using simple aldehydes and alkyl carboxylic acid derived NHPI esters to produce functionalized 1,4-dicarbonyl derivatives. The reaction proceeds through a radical relay mechanism, in which the NHC organocatalyst and Lewis acid can precisely control the SET process, for regioselective decarboxylative carbonylation–acylation, radical addition and radical–radical coupling. Additionally, the Lewis acid–NHC–based DCA strategy presents a new platform for C1 exchange in organic synthesis and enables efficient CO incorporation even with sterically demanding or stereorigid alkyl radicals.

## Author contributions

X.-F. W. conceived and directed the project. M.-L. Y. performed all the experiments and prepared the manuscript and the SI.

## Conflicts of interest

There are no conflicts to declare.

## Supplementary Material

SC-OLF-D5SC09978A-s001

## Data Availability

The data supporting this article have been included as part of the supplementary information (SI). Supplementary information: general comments, general procedure, analytic data, and NMR spectra. See DOI: https://doi.org/10.1039/d5sc09978a.
