# Supplementary material for: Lewis acid/NHC dual catalysis for regioselective vicinal decarboxylative carbonylation–acylation of alkenes
Source: Chem Sci. 2026 Feb 23;17(16):8054–60. doi: 10.1039/d5sc09978a (PMC12947376; doi:10.1039/d5sc09978a)

Supplementary Information (SI) for Chemical Science.
This journal is © The Royal Society of Chemistry 2026

Supporting Information

Lewis Acid/NHC Dual Catalysis for Vicinal Regioselective Decarboxylative Carbonylation Acylation of Alkenes

Mao-Lin Yang[a], Xiao-Feng Wu[a,b*]

[a.] Leibniz-Institut für Katalyse e.V, Albert-Einstein-Straße 29a, 18059 Rostock (Germany), E-mail: xiao-feng.wu@catalysis.de

[b.] Dalian National Laboratory for Clean Energy, Dalian Institute of Chemical Physics, Chinese Academy of Sciences, Dalian, Liaoning (China)

Table of Contents

1. **General information**......S2
2. **Preparation of substrates**......S2
   2.1 Preparation of NHPI Ester 3......S2
3. **Complementary reaction optimization Data**......S3
4. **Characterization and procedure of 1,4-dione products 4**......S5
   4.1 General diacylation procedure for the synthesis of 1,4-diones 4......S5
5. **Mechanistic investigation**......S24
   5.1 Control Experiments and Mechanistic Studies......S24
6. **References**......S26
7. **NMR Spectra**......S27

1. General information

Unless otherwise noted, materials were purchased from commercial suppliers and used without further purification. Flash column chromatography was performed using 200-300 mesh silica gel. ^{1}H NMR spectra were recorded on 300 or 400 MHz spectrophotometers. Chemical shifts are reported in ppm relative to tetramethylsilane (TMS) with the solvent resonance employed as the internal standard ($CDCl_3$: δ = 7.26 ppm). ^{13}C NMR was recorded at 75 MHz or 101 MHz: chemical shifts are reported in ppm from tetramethylsilane (TMS) with the solvent resonance as the internal standard ($CDCl_3$: δ = 77.00 ppm). Electron impact (EI) mass spectra were recorded on AMD 402 mass spectrometer (70 eV). High resolution mass spectra (HR-MS) were recorded on Agilent 6210. The data were given as mass units per charge (m/z). Gas chromatography analysis was performed on an Agilent HP-5890 instrument with an FID detector and HP-5 capillary column (polydimethylsiloxane with 5% phenyl groups, 30 m, 0.32 mm i.d., 0.25 μm film thickness) using argon as carrier gas. Because of the high toxicity of carbon monoxide, all the reactions should be performed in an autoclave. The laboratory should be well-equipped with a CO detector and alarm system.

2. Preparation of substrates

2.1 Preparation of NHPI Ester 3

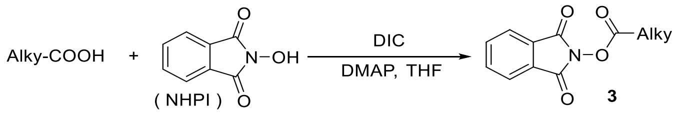

A Schlenk flask equipped with a stir bar and a septum was flushed with argon and charged with dry THF (0.5 M). The carboxylic acid (1.0 equiv.), N,N'-dicyclohexylcarbodiimide (DIC, 1.2 equiv.), 4-dimethylaminopyridine (DMAP, 0.1 equiv.) were added. After stirring for one minute, N-hydroxyphthalimide (NHPI, 1.1 equiv.) was added and the reaction mixture was stirred for 24 h at room temperature. The precipitating dicyclohexyl urea was filtered off and the solution was concentrated by evaporation of the solvent. Flash column chromatography afforded the desired product. N-(Cyclohexylcarbonyloxy)phthalimide (3).[1]

3. Complementary reaction optimization data

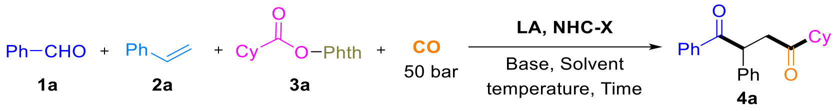

Table 1. Optimization of NHC-X (CO)

Entry	NHC-X	4a, Yield (%)
1	**NHC-1**	N.D.
2	**NHC-2**	60
3	**NHC-3**	20
4	**NHC-4**	10
5	**NHC-5**	trace
6	**NHC-6**	N.D.

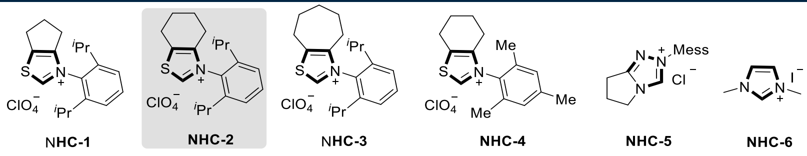

Reaction conditions: **1a** (0.2 mmol), **2a** (0.4 mmol), **3a** (0.1 mmol), DMSO (1.5 mL), Cs_2CO_3 (15 mmol%), **NHC-2** (10 mol%), CO (50 bar), 80 °C, 30 h. Determined by GC with hexadecane as internal standard.

Table 2. Optimization of Lewis acid (LA)

Entry	LA	Yield (%)
1 [a]	NO	60
2 [a]	$AgBF_4$	N.D.
3 [a]	$Cu(OTf)_2$	N.D.
4 [a]	$Zn(OAc)_2$	40
5 [a]	$Zn(OTf)_2$	34
6 [a]	ZnF_2	32
7 [a]	$ZnCl_2$	75
8 [a]	$ZnBr_2$	62
9 [a]	ZnI_2	trace
10 [a]	$ZnCl_2$	71
11 [b]	$ZnCl_2$	80
12 [c]	$ZnCl_2$	10

Reaction conditions: **1a** (0.2 mmol), **2a** (0.4 mmol), **3a** (0.1 mmol), DMSO (1.5 mL), Cs_2CO_3 (15 mmol%), **NHC-2** (10 mol%), **LA** (20 mol%[a], 50 mol%[b], 70 mol%[c]), CO (50 bar), 80 °C, 30 h. Determined by GC with hexadecane as internal standard.

Table 3. Optimization of temperature

Entry	Temperature (ºC)	Yield (%)
1	40	trace
2	60	20
3	80	80
4	100	46

Reaction conditions: **1a** (0.2 mmol), **2a** (0.4 mmol), **3a** (0.1 mmol), DMSO (1.5 mL), Cs_2CO_3 (15 mmol%), **NHC-2** (10 mol%), $ZnCl_2$ (50 mol%), CO (50 bar), Temperture, 30 h. Determined by GC with hexadecane as internal standard.

Table 4. Optimization of solvent

Entry	Solvent	Yield (%)
1 [a]	MeCN	N.D.
2 [a]	THF	N.D.
3 [a]	DMF	N.D.
4 [a]	DMAc	N.D.
5 [a]	DMSO	80
6 [b]	DMSO	70
7 [c]	DMSO	79

Reaction conditions: **1a** (0.2 mmol), **2a** (0.4 mmol), **3a** (0.1 mmol), Solvent (1.5 mL[a], 1.0 mL[b], 2.0 mL[c]), Cs_2CO_3 (15 mmol%), **NHC-2** (10 mol%), $ZnCl_2$ (50 mol%), CO (50 bar), 80 ºC, 30 h. Determined by GC with hexadecane as internal standard.

Table 5. Optimization of CO pressure

Entry	**CO presure**	Yield (%)
1	20 bar	trace
2	40 bar	61
3	50 bar	80
4	60 bar	68

Reaction conditions: Reaction conditions: **1a** (0.2 mmol), **2a** (0.4 mmol), **3a** (0.1 mmol), DMSO (1.5 mL), Cs_2CO_3 (15 mmol%), **NHC-2** (10 mol%), $ZnCl_2$ (50 mol%), CO, 80 ºC, 30 h. Determined by GC with hexadecane as internal standard.

4. Characterization and procedure of 1,4-dione products

4.1 General diacylation procedure for the synthesis of 1,4-diones 4

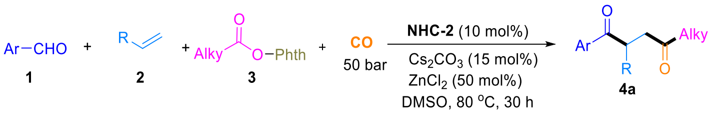

A 4 mL screw-cap vial was charged with NHPI ester **3** (1.0 equiv), **NHC-2** (10 mol%), $ZnCl_2$ (50 mol%), Cs_2CO_3 (15 mol%) and an oven-dried stirring bar. The vial was closed with a Teflon septum and cap and connected to the atmosphere via a needle. After replacing the nitrogen in the vial three times, alkenes **2** (4.0 equiv) was added. Then, aryl aldehydes **1** (2.0 equiv) and DMSO was added using injector. The vial was then moved to a cannula and transferred into a 300 mL photoautoclave (manufactured by Parr Instrument Company®), under a nitrogen atmosphere. At room temperature, the autoclave was washed with CO three times and charged with 50 bar of CO. The autoclave was placed on a heating plate equipped with a magnetic stirrer and an aluminum block. The reaction mixture was allowed to react at 80 °C for 30 hours. After the reaction was complete, the pressure of the autoclave was carefully released, and the residual CO was washed away with nitrogen. The solvent was wash by NH_4Cl (aq.) and ethyl acetate, then removed under vacuum, and the product was purified by column chromatography on silica gel using petroleum ether and ethyl acetate (50:1) to afford the corresponding product **4**.

4-cyclohexyl-1,2-diphenylbutane-1,4-dione (4a)

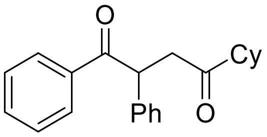

4a

Chromatography Pentane/EA = 50:1 (v/v), 48.0 mg (75%), white solid.

^{1}H NMR (300 MHz, $CDCl_3$) δ 7.91 – 7.89 (m, 2H), 7.41 – 7.08 (m, 8H), 5.09 – 5.04 (m, 1H), 3.60 – 3.51 (m, 1H), 2.88 – 2.80 (m, 1H), 2.77 – 2.68 (m, 1H), 1.80 – 1.18 (m, 10H).
^{13}C NMR (75 MHz, $CDCl_3$) δ 211.2, 199.0, 138.6, 136.4, 132.8, 129.1, 128.8, 128.4, 128.1, 127.2, 51.2, 48.5, 46.5, 28.7, 28.6, 25.9, 25.9.

HRMS (ESI-TOF): m/z calcd. for $C_{22}H_{25}O_2{}^+$ [M+H $^+$] 321.1849, found 321.1855.

4-cyclohexyl-1-(4-isopropylphenyl)-2-phenylbutane-1,4-dione (4b)

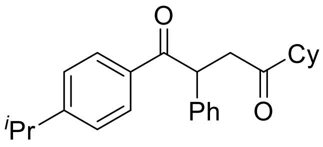

4b

Chromatography Pentane/EA = 50:1 (v/v), 47.0 mg (65%; 95% purity), white solid.

^{1}H NMR (400 MHz, $CDCl_3$) δ 7.84 (d, J = 8.6 Hz, 2H), 7.21 – 7.12 (m, 7H), 5.06 – 5.03 (m, 1H), 3.58 – 3.51 (m, 1H), 2.85 – 2.78 (m, 1H), 2.70 – 2.65 (m, 1H), 2.33 – 2.25 (m, 1H), 1.86 – 1.16 (m, 10H), 1.13 (d, J = 7.0 Hz, 6H).
^{13}C NMR (101 MHz, $CDCl_3$) δ 212.3, 198.6, 154.3, 142.31, 138.9, 135.3, 134.2, 129.1, 129.0, 128.1, 127.1, 126.5, 122.3, 120.5, 50.7, 48.3, 45.5, 34.2, 29.6, 28.3, 25.8, 25.6, 25.6, 23.6.
HRMS (ESI-TOF): m/z calcd. for $C_{25}H_{31}O_2{}^+$ [M+H $^+$] 363.2319, found 363.2311.

4-cyclohexyl-2-phenyl-1-(p-tolyl)butane-1,4-dione (4c)

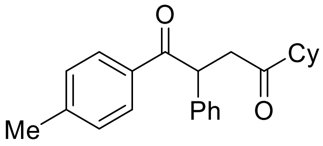

4c

Chromatography Pentane/EA = 50:1 (v/v), 46.2 mg (70%), white solid.

^{1}H NMR (300 MHz, $CDCl_3$) δ 7.82 – 7.79 (m, 2H), 7.20 – 7.07 (m, 7H), 5.07 – 5.02 (m, 1H), 3.58 – 3.49 (m, 1H), 2.87 – 2.77 (m, 1H), 2.74 – 2.67 (m, 1H), 2.25 (s, 3H), 1.80 – 1.18 (m, 10H).
^{13}C NMR (75 MHz, $CDCl_3$) δ 211.2, 198.6, 143.6, 138.9, 133.8, 129.1, 129.0, 128.9, 128.1, 127.1, 51.3, 48.4, 46.4, 28.7, 28.7, 25.9, 25.9, 21.5.

HRMS (ESI-TOF): m/z calcd. for $C_{23}H_{27}O_2{}^+$ [M+H $^+$] 335.2006, found 335.2002.

4-cyclohexyl-1-(4-(dimethylamino)phenyl)-2-phenylbutane-1,4-dione (4d)

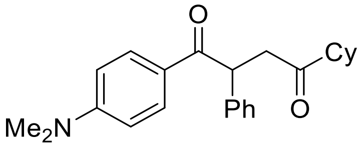

4d

Chromatography Pentane/EA = 50:1 (v/v), 47.9 mg (66%), yellow solid.

^{1}H NMR (300 MHz, $CDCl_3$) δ 7.84 – 7.81 (m, 2H), 7.22 – 6.49 (m, 7H), 5.03 – 4.99 (m, 1H), 3.54 – 3.45 (m, 1H), 2.91 (s, 6H), 2.68 – 2.60 (m, 1H), 2.33 – 2.25 (m, 1H), 1.86 – 1.11 (m, 10H).
^{13}C NMR (75 MHz, $CDCl_3$) δ 212.3, 196.7, 153.1, 140.1, 131.1, 128.9, 128.0, 126.8, 124.4, 110.8, 50.8, 47.8, 45.2, 40.0, 30.9, 28.3, 28.3, 25.9, 25.7, 25.6.

HRMS (ESI-TOF): m/z calcd. for $C_{24}H_{30}NO_2{}^+$ [M+H $^+$] 364.2271, found 364.2278.

4-cyclohexyl-1-(4-fluorophenyl)-2-phenylbutane-1,4-dione (4e)

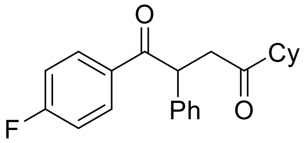

4e

Chromatography Pentane/EA = 50:1 (v/v), 47.0 mg (70%), white solid.

^{1}H NMR (300 MHz, $CDCl_3$) δ 7.94 – 7.89 (m, 2H), 7.24 – 6.93 (m, 7H), 5.01 – 4.97 (m, 1H), 3.60 – 3.50 (m, 1H), 2.72 – 2.64 (m, 1H), 2.35 – 2.26 (m, 1H), 1.89 – 1.14 (m, 10H).
^{13}C NMR (75 MHz, $CDCl_3$) δ 212.2, 197.5, 138.5, 131.5, 131.4, 129.2, 128.0, 127.3, 115.7, 115.4, 50.6, 48.5, 45.5, 28.3, 25.8, 25.6, 25.6.

^{19}F NMR (300 MHz, $CDCl_3$) δ – 105.54 (s, 1H).

HRMS (ESI-TOF): m/z calcd. for $C_{22}H_{24}FO_2{}^+$ [M+H $^+$] 339.1755, found 339.1750.

1-(4-chlorophenyl)-4-cyclohexyl-2-phenylbutane-1,4-dione (4f)

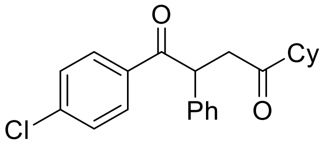

4f

Chromatography Pentane/EA = 50:1 (v/v), 45.5 mg (65%), white solid.

^{1}H NMR (400 MHz, $CDCl_3$) δ 7.84 – 7.82 (m, 2H), 7.29 – 7.12 (m, 7H), 4.99–4.96 (m, 1H), 3.58–3.51 (m, 1H), 2.71–2.66 (m, 1H), 2.534 – 2.27 (m, 1H), 1.87–1.14 (m, 10H).
^{13}C NMR (101 MHz, $CDCl_3$) δ 212.1, 197.9, 139.2, 138.3, 134.7, 130.3, 129.2, 128.8, 128.0, 127.4, 50.6, 48.6, 45.4, 30.4, 28.4, 25.8, 25.6, 25.6.
HRMS (ESI-TOF): m/z calcd. for $C_{22}H_{24}ClO_2{}^{+}$ [M+H $^{+}$] 355.1459, found 355.1450.

1-(4-bromophenyl)-4-cyclohexyl-2-phenylbutane-1,4-dione (4g)

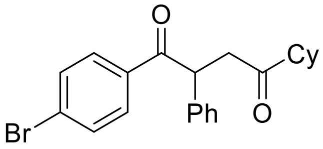

4g

Chromatography Pentane/EA = 50:1 (v/v), 47.0 mg (60%), white solid.

^{1}H NMR (300 MHz, $CDCl_3$) δ 7.84 – 7.81 (m, 2H), 7.28 – 7.10 (m, 8H), 5.00 – 4.95 (m, 1H), 3.59 – 3.50 (m, 1H), 2.72 – 2.64 (m, 1H), 2.34 – 2.26 (m, 1H), 1.88 – 1.11 (m, 10H).
^{13}C NMR (75 MHz, $CDCl_3$) δ 212.1, 197.9, 139.2, 138.3, 134.7, 130.2, 129.2, 128.7, 128.0, 127.4, 50.6, 48.6, 45.4, 28.3, 25.8, 25.6, 25.5.
HRMS (ESI-TOF): m/z calcd. for $C_{22}H_{24}BrO_2{}^{+}$ [M+H $^{+}$] 399.0954, found 399.0950.

4-cyclohexyl-2-phenyl-1-(4-(trifluoromethyl)phenyl)butane-1,4-dione (4h)

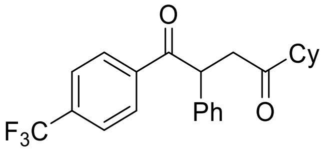

4h

Chromatography Pentane/EA = 50:1 (v/v), 34.0 mg (44%), white solid.

^{1}H NMR (400 MHz, $CDCl_3$) δ 8.00 – 7.97 (m, 2H), 7.58 – 7.13 (m, 7H), 5.03 – 4.99 (m, 1H), 3.62 – 3.55 (m, 1H), 2.75 – 2.69 (m, 1H), 2.35 – 2.28 (m, 1H), 1.88 – 1.13 (m, 10H).
^{13}C NMR (101 MHz, $CDCl_3$) δ 212.1, 198.3, 139.3, 137.8, 129.3, 129.1, 128.1, 127.6, 125.5, 125.5, 50.6, 48.9, 45.5, 28.4, 28.4, 25.8, 25.6, 25.6.
HRMS (ESI-TOF): m/z calcd. for $C_{23}H_{24}FO_2{}^{+}$ [M+H $^{+}$] 389.1723, found 389.1729.
^{19}F NMR (282 MHz, $CDCl_3$) δ – 63.18 (s, 3F).

4-cyclohexyl-2-phenyl-1-(4-(trifluoromethoxy)phenyl)butane-1,4-dione (4i)

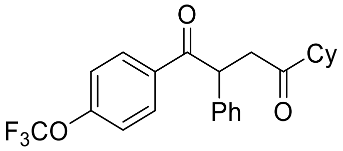

4i

Chromatography Pentane/EA = 50:1 (v/v), 38.0 mg (48%; 95% purity), white solid.

^{1}H NMR (400 MHz, $CDCl_3$) δ 8.03 – 7.97 (m, 2H), 7.57 – 7.14 (m, 7H), 5.03 – 4.99 (m, 1H), 3.62 – 3.55 (m, 1H), 2.75 – 2.69 (m, 1H), 2.35 – 2.28 (m, 1H), 1.88 – 1.12 (m, 10H).

^{13}C NMR (101 MHz, CDCl$_3$) δ 212.1, 198.3, 139.3, 137.8, 129.5, 129.3, 129.1, 128.1, 127.5, 125.6, 125.5, 125.5, 125.4, 50.6, 48.9, 45.5, 28.4, 28.4, 25.8, 25.6, 25.6.
HRMS (ESI-TOF): m/z calcd. for $C_{23}H_{24}F_3O_3^+$ [M+H$^+$] 405.1672, found 405.1665.
^{19}F NMR (282 MHz, CDCl$_3$) δ – 63.18 (s, 3F).

4-cyclohexyl-2-phenyl-1-(m-tolyl)butane-1,4-dione (4j)

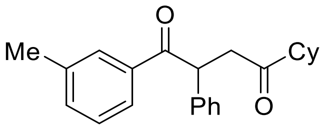

4j

Chromatography Pentane/EA = 50:1 (v/v), 39.6 mg (60%), white solid.

^{1}H NMR (300 MHz, CDCl$_3$) δ 7.71 – 7.75 (m, 2H), 7.18 – 7.08 (m, 7H), 5.04 – 4.99 (m, 1H), 3.54 – 3.44 (m, 1H), 2.69 – 2.62 (m, 1H), 2.36 – 2.28 (m, 1H), 2.27 (s, 3H), 1.87 – 1.13 (m, 10H).
^{13}C NMR (75 MHz, CDCl$_3$) δ 212.1, 199.3, 138.8, 138.1, 136.4, 133.6, 129.3, 129.0, 128.3, 128.1, 127.1, 126.1, 50.7, 48.5, 45.4, 28.3, 25.8, 25.6, 25.6, 21.3.

HRMS (ESI-TOF): m/z calcd. for $C_{23}H_{27}O_3^+$ [M+H$^+$] 335.2006, found 335.2000.

1-(3-chlorophenyl)-4-cyclohexyl-2-phenylbutane-1,4-dione (4k)

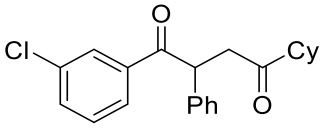

4k

Chromatography Pentane/EA = 50:1 (v/v), 49.0 mg (70%), white solid.

^{1}H NMR (300 MHz, CDCl$_3$) δ 7.84 – 7.81 (m, 2H), 7.27 – 7.09 (m, 7H), 5.00 – 4.95 (m, 1H), 3.59 – 3.50 (m, 1H), 2.71 – 2.64 (m, 1H), 2.33 – 2.25 (m, 1H), 2.00 – 1.07 (m, 10H).
^{13}C NMR (75 MHz, CDCl$_3$) δ 212.1, 197.9, 139.2, 138.3, 134.7, 134.6, 130.2, 129.2, 128.7, 128.0, 127.4, 123.9, 50.6, 48.5, 45.4, 30.2, 28.3, 25.8, 25.6, 25.5.
HRMS (ESI-TOF): m/z calcd. for $C_{22}H_{24}O_2^+$ [M+H$^+$] 355.1459, found 355.1466.

4-cyclohexyl-2-phenyl-1-(o-tolyl)butane-1,4-dionedione (4l)

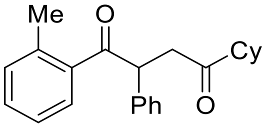

4l

Chromatography Pentane/EA = 50:1 (v/v), 28.1 mg (40%), white solid.

^{1}H NMR (300 MHz, CDCl$_3$) δ 7.76 – 7.73 (m, 1H), 7.21 – 7.01 (m, 8H), 4.89 – 4.84 (m, 1H), 3.67 – 3.58 (m, 1H), 2.69 – 2.62 (m, 1H), 2.40 – 2.29 (m, 1H), 2.15 (s, 3H), 1.91 – 1.13 (m, 10H).
^{13}C NMR (75 MHz, CDCl$_3$) δ 212.4, 202.97, 138.4, 137.9, 137.6, 131.3, 130.7, 128.9, 128.3, 128.3, 127.2, 125.4, 51.5, 50.6, 44.7, 29.7, 28.4, 25.8, 25.6, 25.6, 20.4.

HRMS (ESI-TOF): m/z calcd. for $C_{22}H_{24}O_2^+$ [M+H$^+$] 355.2006, found 355.2011.

2-(4-chlorophenyl)-4-cyclohexyl-1-phenylbutane-1,4-dione (4m)

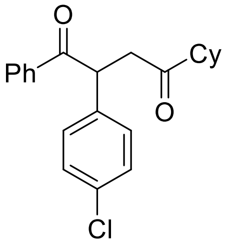

4m

Chromatography Pentane/EA = 50:1 (v/v), 55.0 mg (78%), white solid.

^{1}H NMR (300 MHz, $CDCl_3$) δ 7.88 – 7.86 (m, 2H), 7.44 – 7.11 (m, 7H), 5.06 – 5.01 (m, 1H), 3.56 – 3.47 (m, 1H), 2.71 – 2.64 (m, 1H), 2.34 – 2.26 (m, 1H), 1.88 – 1.16 (m, 10H).
^{13}C NMR (75 MHz, $CDCl_3$) δ 211.8, 198.8, 137.2, 136.1, 133.1, 133.0, 129.5, 129.2, 128.8, 128.5, 50.6, 47.7, 45.2, 29.7, 28.3, 25.8, 25.6, 25.5.

HRMS (ESI-TOF): m/z calcd. for $C_{22}H_{24}ClO_2^+$ [M+H$^+$] 355.1459, found 355.1463.

2-(4-bromophenyl)-4-cyclohexyl-1-phenylbutane-1,4-dione (4n)

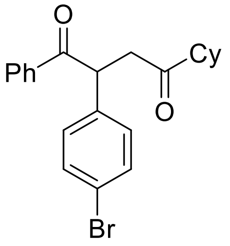

4n

Chromatography Pentane/EA = 50:1 (v/v), 55.0 mg (70%), white solid.

^{1}H NMR (400 MHz, $CDCl_3$) δ 7.88 – 7.66 (m, 3H), 7.42 – 7.06 (m, 6H), 5.04– 5.01 (m, 1H), 3.55 – 3.48 (m, 1H), 2.70 – 2.65 (m, 1H), 2.34 – 2.26 (m, 1H), 1.87 – 1.05 (m, 10H).
^{13}C NMR (101 MHz, $CDCl_3$) δ 211.8, 198.7, 137.7, 136.1, 134.3, 133.0, 132.2, 129.8, 128.8, 128.5, 123.6, 121.2, 50.6, 47.8, 45.1, 28.3, 25.8, 25.6, 25.5.
HRMS (ESI-TOF): m/z calcd. for $C_{22}H_{23}BrO_2^+$ [M+H $^+$] 399.0954, found 399.0950.

4-cyclohexyl-1-phenyl-2-(p-tolyl)butane-1,4-dione (4o)

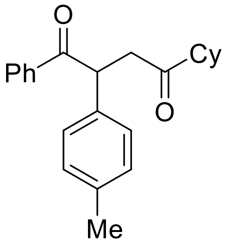

4o

Chromatography Pentane/EA = 50:1 (v/v), 45.4 mg (68%), white solid.

^{1}H NMR (300 MHz, $CDCl_3$) δ 7.90 – 7.88 (m, 2H), 7.41 – 6.71 (m, 7H), 5.02 – 4.98 (m, 1H), 3.66 (s, 3H), 3.56 – 3.47 (m, 1H), 2.70 – 2.63 (m, 1H), 2.34 – 2.25 (m, 1H), 1.87 – 1.12 (m, 10H).
^{13}C NMR (75 MHz, $CDCl_3$) δ 212.3, 199.3, 158.7, 136.4, 132.7, 130.6, 129.2, 128.8, 128.4, 114.5, 55.2, 50.7, 47.6, 45.4, 28.3, 28.3, 25.8, 25.6, 25.6.

HRMS (ESI-TOF): m/z calcd. for $C_{23}H_{27}O_2^+$ [M+H $^+$] 335.2006, found 335.2009.

2-(4-(tert-butyl)phenyl)-4-cyclohexyl-1-phenylbutane-1,4-dione (4p)

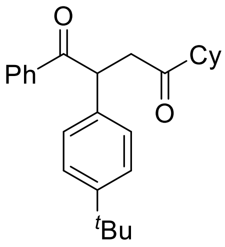

4p

Chromatography Pentane/EA = 50:1 (v/v), 52.0 mg (70%; 95% purity), white solid.

^{1}H NMR (400 MHz, $CDCl_3$) δ 7.93 – 7.90 (m, 2H), 7.41 – 7.10 (m, 7H), 5.04 – 5.01 (m, 1H), 3.60 – 3.52 (m, 1H), 2.70 – 2.65 (m, 1H), 2.33 – 2.26 (m, 1H), 1.86 – 1.21 (m, 10H), 1.18 (s, 9H).
^{13}C NMR (101 MHz, $CDCl_3$) δ 212.3, 199.2, 150.1, 136.5, 135.4, 132.7, 128.9, 128.4, 127.7, 126.0, 50.6, 47.9, 45.6, 34.4, 31.23, 28.4, 28.3, 25.8, 25.6, 25.6.
HRMS (ESI-TOF): m/z calcd. for $C_{26}H_{33}O_2^+$ [M+H$^+$] 377.2475, found 377.2470.

4-cyclohexyl-2-(4-methoxyphenyl)-1-phenylbutane-1,4-dione (4q)

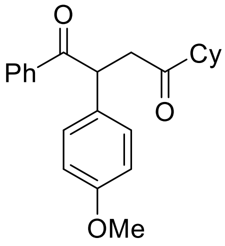

4q

Chromatography Pentane/EA = 50:1 (v/v), 42.0 mg (60%), white solid.

^{1}H NMR (300 MHz, $CDCl_3$) δ 7.90 – 7.87 (m, 2H), 7.42 – 6.72 (m, 7H), 5.03 – 4.98 (m, 1H), 3.67 (s, 3H), 3.56 – 3.47 (m, 1H), 2.71 – 2.63 (m, 1H), 2.34 – 2.24 (m, 1H), 1.76 – 1.14 (m, 10H).
^{13}C NMR (75 MHz, $CDCl_3$) δ 212.3, 199.3, 158.7, 136.5, 132.7, 130.6, 129.2, 128.8, 128.4, 114.5, 55.2, 50.7, 47.6, 45.4, 28.3, 28.3, 25.8, 25.6, 25.6.

HRMS (ESI-TOF): m/z calcd. for $C_{23}H_{27}O_2^+$ [M+H $^+$] 351.1955, found 351.1958.

4-cyclohexyl-2-(4-phenoxyphenyl)-1-phenylbutane-1,4-dione (4r)

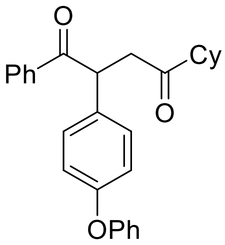

4r

Chromatography Pentane/EA = 50:1 (v/v), 41.1 mg (50%), white solid.

^{1}H NMR (300 MHz, $CDCl_3$) δ 7.92 – 7.89 (m, 2H), 7.44 – 6.80 (m, 12H), 5.06 – 5.01 (m, 1H), 3.58 – 3.49 (m, 1H), 2.74 – 2.66 (m, 1H), 2.35 – 2.26 (m, 1H), 1.88 – 1.07 (m, 10H).
^{13}C NMR (75 MHz, $CDCl_3$) δ 212.1, 199.1, 156.7, 156.5, 136.4, 133.2, 132.8, 129.7, 129.4, 128.9, 128.4, 123.4, 119.1, 119.0, 50.7, 47.6, 45.5, 28.3, 28.3, 25.8, 25.6, 25.5.

HRMS (ESI-TOF): m/z calcd. for $C_{28}H_{29}O_3^+$ [M+H $^+$] 413.2111, found 413.2119.

2-(4-(benzyloxy)phenyl)-4-cyclohexyl-1-phenylbutane-1,4-dione (4s)

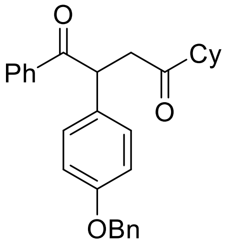

4s

Chromatography Pentane/EA = 50:1 (v/v), 50.0 mg (60%), yellow solid.

^{1}H NMR (300 MHz, $CDCl_3$) δ 7.90 – 7.88 (m, 2H), 7.42 – 6.79 (m, 12H), 5.03 – 4.98 (m, 1H), 4.91 (s, 2H), 3.56 – 3.47 (m, 1H), 2.71 – 2.63 (m, 1H), 2.34 – 2.25 (m, 1H), 1.86 – 1.07 (m, 10H).
^{13}C NMR (75 MHz, $CDCl_3$) δ 212.3, 199.3, 158.0, 136.8, 136.5, 132.7, 130.9, 129.2, 128.8, 128.6, 128.4, 128.0, 127.4, 115.4, 70.0, 50.7, 47.6, 45.4, 28.4, 28.3, 25.8, 25.6, 25.6.

HRMS (ESI-TOF): m/z calcd. for $C_{29}H_{31}O_3^+$ [M+H $^+$] 427.2268, found 427.2260.

4-cyclohexyl-2-(4-(difluoromethoxy)phenyl)-1-phenylbutane-1,4-dione (4t)

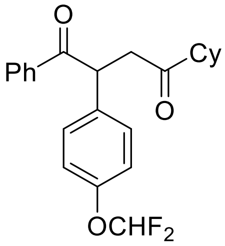

4t

Chromatography Pentane/EA = 50:1 (v/v), 34.2 mg (45%), white solid.

^{1}H NMR (300 MHz, $CDCl_3$) δ 7.91 – 7.87 (m, 2H), 7.44 – 6.94 (m, 7H), 6.25 (t, J = 73.8 Hz, 1H), 5.08 – 5.04 (m, 1H), 5.57 – 3.48 (m, 1H), 2.73 – 2.65 (m, 1H), 2.34 – 2.26 (m, 1H), 1.86 – 1.15 (m, 10H).
^{13}C NMR (75 MHz, $CDCl_3$) δ 211.9, 198.9, 150.2, 141.6, 136.2, 135.8, 133.0, 129.5, 128.8, 128.5, 120.2, 115.7, 50.66, 47.6, 45.3, 29.7, 28.3, 25.8, 25.6, 25.5.
^{19}F NMR (282 MHz, $CDCl_3$) δ -80.89 (d, J = 73.9 Hz).
HRMS (ESI-TOF): m/z calcd. for $C_{23}H_{25}F_2O_3^+$ [M+H $^+$] 387.1766, found 387.1760.

4-cyclohexyl-1-phenyl-2-(4-((trifluoromethyl)thio)phenyl)butane-1,4-dione (4u)

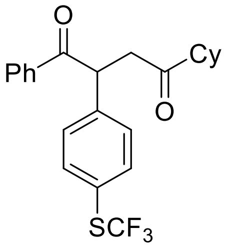

4u

Chromatography Pentane/EA = 50:1 (v/v), 42.0 mg (50%), yellow solid.

^{1}H NMR (300 MHz, $CDCl_3$) δ 7.92 – 7.89 (m, 2H), 7.59 – 7.12 (m, 7H), 5.01 – 4.96 (m, 1H), 3.62 – 3.53 (m, 1H), 3.75 – 2.68 (m, 1H), 2.37 – 2.26 (m, 1H), 1.87 – 1.05 (m, 10H).
^{13}C NMR (75 MHz, $CDCl_3$) δ 212.1, 198.3, 138.1, 137.9, 135.5, 129.6, 129.3, 128.1, 127.5, 127.2, 50.6, 48.8, 45.6, 28.4, 25.8, 25.6, 25.6.
HRMS (ESI-TOF): m/z calcd. for $C_{23}H_{24}F_3O_2S^+$ [M+H $^+$] 421.1444, found 421.1440.
^{19}F NMR (282 MHz, $CDCl_3$) δ – 41.70 (s, 3F).

4-cyclohexyl-2-(naphthalen-2-yl)-1-phenylbutane-1,4-dione (4v)

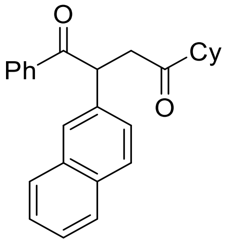

4v

Chromatography Pentane/EA = 50:1 (v/v), 51.8 mg (70%), yellow solid.

^{1}H NMR (300 MHz, $CDCl_3$) δ 7.94 – 7.91 (m, 2H), 7.82 – 7.24 (m, 10H), 5.24 – 5.19 (m, 1H), 3.69 – 3.60 (m, 1H), 2.79 – 2.72 (m, 1H), 2.37 – 2.28 (m, 1H), 1.90 – 1.14 (m, 10H).
^{13}C NMR (75 MHz, $CDCl_3$) δ 212.1, 199.0, 136.4, 136.2, 134.6, 133.6, 132.8, 132.4, 128.9, 128.9, 128.4, 127.7, 127.6, 127.0, 126.3, 126.0, 126.0, 123.9, 50.7, 48.6, 45.5, 28.8, 28.4, 25.8, 25.6, 25.0.
HRMS (ESI-TOF): m/z calcd. for $C_{26}H_{27}O_2{}^+$ [M+H$^+$] 371.2006, found 371.2000.

2-(benzo[d][1,3]dioxol-4-yl)-4-cyclohexyl-1-phenylbutane-1,4-dione (4w)

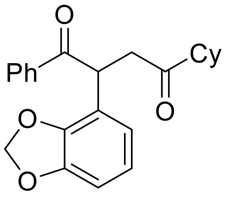

4w

Chromatography Pentane/EA = 50:1 (v/v), 43.4 mg (60%), yellow solid.

^{1}H NMR (400 MHz, $CDCl_3$) δ 7.90 – 7.88 (m, 2H), 7.41 – 6.61 (m, 6H), 5.82– 5.80 (m, 2H), 4.98 – 4.94 (m, 1H), 3.53 – 3.46 (m, 1H), 2.69 – 2.63 (m, 1H), 2.34 – 2.26 (m, 1H), 1.86 – 1.16 (m, 10H).
^{13}C NMR (101 MHz, $CDCl_3$) δ 212.1, 199.0, 148.1, 146.7, 136.3, 132.8, 132.3, 128.8, 128.4, 121.5, 108.7, 108.3, 101.1, 50.7, 48.0, 45.4, 28.3, 25.8, 25.6, 25.5.
HRMS (ESI-TOF): m/z calcd. for $C_{23}H_{24}O_4{}^+$ [M+H$^+$] 365.1747, found 365.1751.

4-cyclohexyl-2-(2-methoxyphenyl)-1-phenylbutane-1,4-dione (4x)

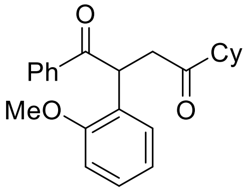

4x

Chromatography Pentane/EA = 50:1 (v/v), 35.0 mg (50%), white solid.

^{1}H NMR (300 MHz, $CDCl_3$) δ 7.90 – 7.86 (m, 2H), 7.39 – 6.72 (m, 7H), 5.49 – 5.44 (m, 1H), 3.81 (s, 3H), 3.50 – 3.41 (m, 1H), 2.60 – 2.53 (m, 1H), 2.39 – 2.29 (m, 1H), 1.92 – 1.09 (m, 10H).
^{13}C NMR (75 MHz, $CDCl_3$) δ 212.4, 199.7, 155.9, 136.4, 132.6, 128.7, 128.6, 128.3, 128.2, 127.2, 121.0, 110.9, 55.4, 50.7, 43.8, 41.3, 28.4, 28.4, 25.9, 25.7, 25.6.

HRMS (ESI-TOF): m/z calcd. for $C_{23}H_{27}O_3{}^+$ [M+H$^+$] 351.1955, found 351.1950.

4-cyclohexyl-1-phenyl-2-(m-tolyl)butane-1,4-dione (4y)

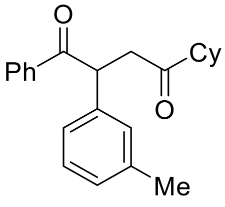

4y

Chromatography Pentane/EA = 50:1 (v/v), 44.2 mg (66%), white solid.

^{1}H NMR (300 MHz, $CDCl_3$) δ 7.92 – 7.88 (m, 2H), 7.42 – 6.91 (m, 7H), 5.03 – 4.98 (m, 1H), 3.60 – 3.51 (m, 1H), 2.70 – 2.62 (m, 1H), 2.34 – 2.25 (m, 1H), 2.21 (s, 3H), 1.88 – 1.08 (m, 10H).
^{13}C NMR (75 MHz, $CDCl_3$) δ 212.2, 199.1, 138.8, 138.6, 136.5, 132.8, 128.9, 128.8, 128.6, 128.4, 128.0, 125.2, 50.6, 48.4, 45.5, 30.9, 28.4, 25.8, 25.6, 25.6, 21.4.

HRMS (ESI-TOF): m/z calcd. for $C_{23}H_{27}O_2^+$ [M+H$^+$] 335.2006, found 335.2009.

2-(4-chlorophenyl)-4-cyclohexyl-3-methyl-1-phenylbutane-1,4-dione (4z)

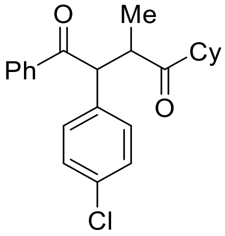

4z

Chromatography Pentane/EA = 50:1 (v/v), 21.9 mg (30%), viscous liquid.

^{1}H NMR (300 MHz, $CDCl_3$) δ 7.91 – 7.82 (m, 2H), 7.48 – 7.11 (m, 7H), 4.84 – 4.81 (m, 0.8H), 4.76 – 4.72 (m, 0.2H), 2.02 – 1.93 (m, 1H), 1.63 – 0.67 (m, 14H).
^{13}C NMR (75 MHz, $CDCl_3$) δ 216.9(minor), 215.9, 200.8(minor), 198.6, 139.6(minor), 139.0(minor), 138.4, 136.8, 135.8(minor), 135.7(minor), 133.3, 132.9(minor), 130.4, 130.2(minor), 129.2, 128.9, 128.7, 128.6, 128.5(minor), 128.5(minor), 55.5(minor), 54.8, 51.3, 49.6(minor), 48.3, 48.2(minor), 29.1(minor), 28.2(minor), 27.8, 27.2, 25.9(minor), 25.8, 25.77, 25.6(minor), 25.5, 25.4(minor), 16.9, 15.1(minor).

HRMS (ESI-TOF): m/z calcd. for $C_{23}H_{26}ClO_2^+$ [M+H$^+$] 369.1616, found 369.1610.

1-cyclohexyl-2-methyl-3,4-diphenylbutane-1,4-dione (4aa)

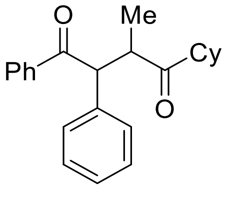

4aa

Chromatography Pentane/EA = 50:1 (v/v), 40.0 mg (60%; 95% purity), viscous liquid.

^{1}H NMR (400 MHz, $CDCl_3$) δ 7.94 – 7.84 (m, 2H), 7.42 – 7.08 (m, 8H), 4.83 – 4.78 (m, 1H), 3.73 – 3.63 (m, 0.5H), 3.60 – 3.48 (m, 0.5H), 2.88 – 2.80 (m, 1H), 2.77 – 2.68 (m, 1H), 1.78 – 0.86 (m, 12H).
^{13}C NMR (75 MHz, $CDCl_3$) δ 217.4, 216.5(minor), 200.0(minor), 198.8, 137.0, 137.0(minor), 136.9(minor), 136.6, 135.3(minor), 133.1(minor), 132.7, 131.6, 130.0(minor), 129.0, 129.0, 128.9, 128.7(minor), 128.7, 128.6(minor), 128.3, 127.9(minor), 127.4, 122.3(minor), 120.5(minor), 56.3, 55.9(minor), 51.7, 49.7, 48.3, 29.6(minor), 29.1, 28.3, 27.9(minor), 26.9, 26.0(minor), 25.9(minor), 25.7, 25.6(minor), 25.5, 25.3(minor), 16.9, 15.1(minor).
HRMS (ESI-TOF): m/z calcd. for $C_{23}H_{27}O_2^+$ [M+H$^+$] 335.2006, found 335.2008.

ethyl 2-(cyclohexylmethyl)-3-oxo-3-phenylpropanoate (5a)

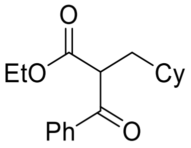

5a

Chromatography Pentane/EA = 50:1 (v/v), 34.5 mg (60%), white solid..

^{1}H NMR (300 MHz, $CDCl_3$) δ 7.94 – 7.90 (m, 2H), 7.54 – 7.37 (m, 3H), 4.37 – 4.32 (m, 1H), 4.11 – 4.04 (m, 2H), 1.85 – 1.54 (m, 6H), 1.29 – 1.15 (m, 3H), 1.13 – 1.05 (m, 5H), 0.92 – 0.82 (m, 2H).
^{13}C NMR (75 MHz, $CDCl_3$) δ 195.4, 170.2, 136.1, 133.4, 128.7, 128.5, 61.2, 51.8, 36.3, 35.8, 33.3, 32.9, 26.3, 26.1, 26.0, 14.0.

HRMS (ESI-TOF): m/z calcd. for $C_{18}H_{25}O_3^+$ [M+H$^+$] 289.1798, found 289.1790.

methyl 2-(cyclohexylmethyl)-3-oxo-3-phenylpropanoate (5b)

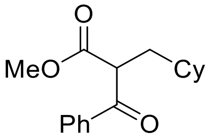

5b

Chromatography Pentane/EA = 50:1 (v/v), 35.6 mg (65%), white solid..

^{1}H NMR (300 MHz, $CDCl_3$) δ 7.93 – 7.90 (m, 2H), 7.55 – 7.38 (m, 3H), 4.41 – 4.36 (m, 1H), 3.61 (s, 3H), 1.90 – 1.57 (m, 5H), 1.29 – 1.05 (m, 6H), 0.93 – 0.78 (m, 2H).
^{13}C NMR (75 MHz, $CDCl_3$) δ 195.3, 170.7, 136.1, 133.5, 128.8, 128.6, 52.4, 51.5, 36.4, 35.8, 33.2, 33.0, 26.3, 26.1.

HRMS (ESI-TOF): m/z calcd. for $C_{17}H_{23}O_3^+$ [M+H$^+$] 275.1642, found 275.1644.

4-cyclopropyl-1,2-diphenylbutane-1,4-dione (4ab)

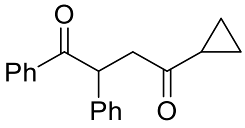

4ab

Chromatography Pentane/EA = 50:1 (v/v), 25.0 mg (45%), white solid.

^{1}H NMR (400 MHz, $CDCl_3$) δ 7.91 – 7.88 (m, 2H), 7.41 – 7.10 (m, 8H), 5.08 – 5.03 (m, 1H), 3.72 – 3.63 (m, 1H), 2.88 – 2.81 (m, 1H), 1.92 – 1.84 (m, 1H), 1.30 – 0.73 (m, 4H).
^{13}C NMR (75 MHz, $CDCl_3$) δ 208.8, 198.9, 138.5, 136.4, 132.9, 129.1, 128.8, 128.4, 128.1, 127.2, 48.5, 47.8, 20.6, 10.8, 10.7.
HRMS (ESI-TOF): m/z calcd. for $C_{19}H_{19}O_2^+$ [M+H$^+$] 279.1380, found 279.1372.

4-cyclobutyl-1,2-diphenylbutane-1,4-dione (4ac)

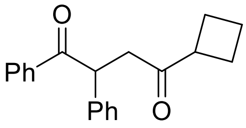

4ac

Chromatography Pentane/EA = 50:1 (v/v), 23.4 mg (40%), white solid..

^{1}H NMR (300 MHz, $CDCl_3$) δ 7.91 – 7.88 (m, 2H), 7.41 – 7.08 (m, 8H), 5.08 – 5.08 (m, 1H), 3.48– 3.39 (m, 1H), 3.27– 3.15 (m, 1H), 2.65–2.58 (m, 1H), 2.29–1.18 (m, 10H).

^{13}C NMR (75 MHz, $CDCl_3$) δ 209.8, 199.0, 138.6, 136.34, 132.80, 129.1, 128.8, 128.4, 128.1, 127.2, 48.4, 45.2, 44.6, 24.2, 24.1, 17.7.
HRMS (ESI-TOF): m/z calcd. for $C_{20}H_{21}O_2^+$ [M+H$^+$] 293.1536, found 293.1540.

4-cyclopentyl-1,2-diphenylbutane-1,4-dione (4ad)

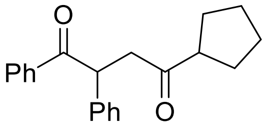

4ad

Chromatography Pentane/EA = 50:1 (v/v), 35.5 mg (58%), white solid..

^1H NMR (300 MHz, $CDCl_3$) δ 7.92 – 7.87 (m, 2H), 7.39 –7.07 (m, 8H), 5.08 – 5.04 (m, 1H), 3.60 – 3.50 (M, 1H), 2.87–2.77 (m, 1H), 2.75–2.67 (m, 1H), 1.79 – 1.20 (m, 8H).
^{13}C NMR (75 MHz, $CDCl_3$) δ 211.1, 199.0, 138.6, 136.4, 132.8, 129.0, 128.8, 128.4, 128.0, 127.2, 51.2, 48.5, 46.4, 28.7, 28.6, 25.9, 25.9.
HRMS (ESI-TOF): m/z calcd. for $C_{21}H_{23}O_2^+$ [M+H$^+$] 307.1693, found 307.1690.

4-(cyclohex-3-en-1-yl)-1,2-diphenylbutane-1,4-dione (4ae)

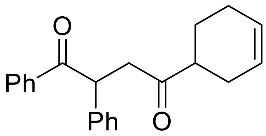

4ae

Chromatography Pentane/EA = 50:1 (v/v), 39.4 mg (62%), viscous liquid.

^1H NMR (400 MHz, $CDCl_3$) δ 7.91 – 7.89 (m, 2H), 7.41 – 7.10 (m, 8H), 5.63 – 5.58 (m, 2H), 5.09 – 5.06 (m, 1H), 3.67– 3.60 (m, 0.5H), 3.60– 3.53 (m, 0.5H), 2.80 – 2.67 (m, 1H), 2.63 – 2.54 (m, 1H), 2.17 – 1.18 (m, 6H).
^{13}C NMR (101 MHz, $CDCl_3$) δ 211.6, 199.0, 138.6, 136.4, 132.8, 129.1, 128.8, 128.4, 128.1, 127.2, 126.8, 126.5, 125.4, 125.1, 48.6, 48.5, 46.5, 46.5, 45.5, 45.5, 26.7, 26.7, 24.6, 24.6, 24.5, 24.5.
HRMS (ESI-TOF): m/z calcd. for $C_{22}H_{23}O_2^+$ [M+H$^+$] 319.1693, found 319.1695.

5-methyl-1,2-diphenylhexane-1,4-dione (4af)

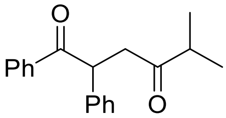

4af

Chromatography Pentane/EA = 50:1 (v/v), 43.6 mg (78%), white solid..

^1H NMR (300 MHz, $CDCl_3$) δ 7.91 – 7.88 (m, 2H), 7.41– 7.10 (m, 8H), 5.08 – 5.03 (m, 1H), 3.60 – 3.51 (m, 1H), 2.75 – 2.68 (m, 1H), 2.61 – 2.52 (m, 1H), 1.05 (d, J = 6.0, 3H), 1.00 (d, J = 6.0, 3H).
^{13}C NMR (75 MHz, $CDCl_3$) δ 212.8, 199.0, 138.6, 136.4, 134.7, 132.8, 129.1, 128.8, 128.4, 128.1, 127.2, 123.9, 48.6, 45.1, 40.8, 18.1.
HRMS (ESI-TOF): m/z calcd. for $C_{19}H_{21}O_2^+$ [M+H$^+$] 281.1536, found 281.1530.

5-methyl-1,2-diphenylheptane-1,4-dione (4ag)

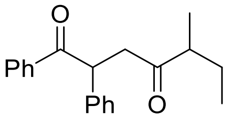

4ag

Chromatography Pentane/EA = 50:1 (v/v), 39.9 mg (68%), viscous liquid.

^1H NMR (400 MHz, $CDCl_3$) δ 7.91 – 7.89 (m, 2H), 7.40 – 7.09 (m, 8H), 5.09– 5.04 (m, 1H), 3.59 – 3.51 (m, 1H), 2.75 – 2.66 (m, 1H), 2.46 – 2.36 (m, 1H), 1.90 – 1.85 (m, 0.1H), 1.70 – 1.54 (m, 0.9H), 1.39 – 1.25 (m, 1H), 1.03 – 0.96 (m, 3H), 0.83 – 0.74 (m, 3H).

^{13}C NMR (101 MHz, $CDCl_3$) δ 212.8(minor), 212.8, 199.0(minor), 199.0, 138.6, 136.4(minor), 136.4, 134.8(minor), 134.7, 132.8(minor), 132.8, 129.1, 128.8(minor), 128.8, 128.4, 128.1, 127.2, 123.9(minor), 123.9, 48.4, 47.8(minor), 47.7(minor), 46.0, 25.9(minor), 25.9, 15.7, 15.6(minor), 11.5, 10.6, 10.3(minor).
HRMS (ESI-TOF): m/z calcd. for $C_{20}H_{23}O_2^+$ [M+H $^+$] 295.1693, found 295.1690.

5-methyl-1,2-diphenyloctane-1,4-dione (4ah)

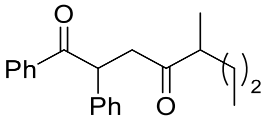

4ah

Chromatography Pentane/EA = 50:1 (v/v), 36.9 mg (60%), viscous liquid.

^1H NMR (400 MHz, $CDCl_3$) δ 8.02 – 7.46 (m, 4H), 7.51 – 7.20 (m, 6H), 5.19 – 5.13 (m, 1H), 3.70 – 2.60 (m, 1H), 2.91 – 2.76 (m, 1H), 2.64 – 2.55 (m, 0.9H), 2.42 – 2.41 (m, 0.1H), 1.40 – 0.86 (m, 10H).
^{13}C NMR (101 MHz, $CDCl_3$) δ 212.9(minor), 212.9, 199.2(minor), 199.0, 138.6(minor),, 138.6, 136.4(minor),, 136.4, 134.6(minor), 134.3, 132.8, 132.6(minor), 129.9(minor), 129.1, 128.8, 128.4, 128.1, 127.2(minor), 123.9(minor), 123.5, 48.4, 46.0, 46.0(minor), 45.9, 45.8(minor), 35.0, 20.3, 20.1(minor), 16.9(minor), 16.1, 16.0(minor), 14.1, 14.0(minor).
HRMS (ESI-TOF): m/z calcd. for $C_{21}H_{25}O_2^+$ [M+H$^+$] 309.1849, found 309.1840.

5-methyl-1,2-diphenyldecane-1,4-dione (4ai)

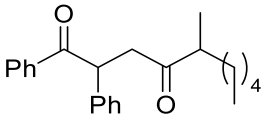

4ai

Chromatography Pentane/EA = 50:1 (v/v), 41.6 mg (62%), viscous liquid.

^1H NMR (400 MHz, $CDCl_3$) δ 7.92 – 7.89 (m, 2H), 7.46 – 7.10 (m, 8H), 5.09– 5.03 (m, 1H), 3.58 – 3.54 (m, 0.5H), 3.52 – 3.49 (m, 0.5H), 2.76 – 2.67 (m, 1H), 2.53 – 2.43 (m, 1H), 1.30 – 0.77 (m, 14H).
^{13}C NMR (101 MHz, $CDCl_3$) δ 212.9(minor), 212.9, 199.0(minor), 199.0, 138.6, 136.4(minor), 136.4, 134.9(minor), 134.3, 132.8(minor), 132.8, 129.9(minor), 129.1, 129.0(minor), 128.8, 128.4, 128.1, 127.2, 123.6(minor), 48.5, 48.4(minor), 46.3, 46.0, 45.7(minor), 32.9, 32.8(minor), 31.8, 26.8(minor), 26.8, 22.5(minor), 22.4, 16.1, 14.0, 14.0(minor).
HRMS (ESI-TOF): m/z calcd. for $C_{23}H_{29}O_2^+$ [M+H $^+$] 337.2162, found 337.2160.

5-methyl-1,2-diphenyloct-7-ene-1,4-dione (4aj)

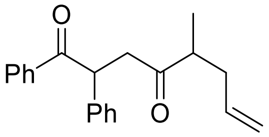

4aj

Chromatography Pentane/EA = 50:1 (v/v), 39.1 mg (65%), viscous liquid.

^1H NMR (300 MHz, $CDCl_3$) δ 7.91 – 7.88 (m, 2H), 7.41– 7.10 (m, 8H), 5.75– 5.53 (m, 1H), 5.08 – 5.06 (m, 0.5H), 5.05 – 5.03 (m, 0.5H), 5.01 – 4.89 (m, 2H), 3.62 – 3.50 (m, 1H), 2.77 – 2.52 (m, 2H), 2.43 – 2.34 (m, 0.51H), 2.31 – 2.25 (m, 0.56H), 2.08 – 2.03 (m, 0.5H), 2.00 – 1.95 (m, 0.5H), 1.06 – 1.04 (m, 1.50H), 0.99 – 0.97 (m, 1.57H).
^{13}C NMR (75 MHz, $CDCl_3$) δ 211.9(minor), 211.9, 198.9(minor), 198.9, 138.6(minor), 138.6, 136.4, 136.4(minor), 135.6(minor), 135.5, 134.3, 132.8, 129.9, 129.1, 129.0, 128.8(minor), 128.4, 128.1(minor), 127.3, 127.2(minor), 126.0(minor), 123.6, 116.8(minor), 116.8, 48.5, 48.5(minor), 46.3(minor), 45.8, 36.9(minor), 36.8, 15.9(minor), 15.7.
HRMS (ESI-TOF): m/z calcd. for $C_{21}H_{23}O_2^+$ [M+H $^+$] 307.1693, found 307.1690.

5,5-dimethyl-1,2-diphenylhexane-1,4-dione (4ak)

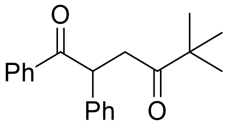

4ak

Chromatography Pentane/EA = 50:1 (v/v), 25.8 mg (44%), white solid.

^{1}H NMR (400 MHz, CDCl$_3$) δ 7.92 – 7.90 (m, 2H), 7.41 – 7.11 (m, 8H), 5.07 – 5.03 (m, 1H), 3.65 – 3.58 (m, 1H), 2.78 – 2.73 (m, 1H), 1.08 (s, 9H).
^{13}C NMR (101 MHz, CDCl$_3$) δ 214.2, 199.1, 138.7, 136.5, 132.8, 129.1, 128.8, 128.4, 128.2, 127.2, 48.5, 43.9, 42.2, 26.4.
HRMS (ESI-TOF): m/z calcd. for $C_{20}H_{23}O_2^+$ [M+H$^+$] 295.1693, found 295.1699.

1-(4-isopropylphenyl)-5,5-dimethyl-2-phenylhexane-1,4-dione (4al)

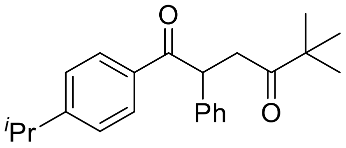

4al

Chromatography Pentane/EA = 50:1 (v/v), 26.8 mg (40%), white solid.

^{1}H NMR (300 MHz, CDCl$_3$) δ 7.87 – 7.84 (m, 2H), 7.22 – 7.13 (m, 7 H), 5.06 – 5.02 (m, 1H), 3.66 – 3.56 (m, 1H), 2.87 – 2.76 (m, 1H), 2.72 – 2.70 (m, 1H), 1.15 – 1.13 (m, 6H), 1.07 (s, 9H).
^{13}C NMR (75 MHz, CDCl$_3$) δ 214.3, 198.6, 154.3, 138.9, 134.3, 129.1, 129.1, 128.2, 127.2, 126.6, 48.3, 43.9, 42.3, 34.2, 26.4, 23.6.

HRMS (ESI-TOF): m/z calcd. for $C_{23}H_{29}O_2^+$ [M+H$^+$] 337.2162, found 337.2160.

2-(4-bromophenyl)-5,5-dimethyl-1-phenylhexane-1,4-dione (4am)

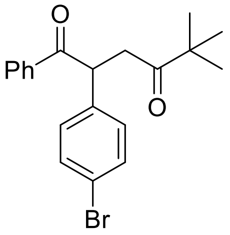

4am

Chromatography Pentane/EA = 50:1 (v/v), 37.2 mg (50%), white solid.

^{1}H NMR (300 MHz, CDCl$_3$) δ 7.83 – 7.80 (m, 2H), 7.36 – 7.05 (m, 7H), 4.96 – 4.92 (m, 1H), 3.61 – 3.52 (m, 1H), 2.77 – 2.70 (m, 1H), 1.08 (s, 9H).
^{13}C NMR (75 MHz, CDCl$_3$) δ 213.9, 197.6, 139.5, 137.3, 134.6, 132.3, 130.2, 129.8, 128.9, 121.5, 47.9, 43.9, 42.0, 26.4.
HRMS (ESI-TOF): m/z calcd. for $C_{20}H_{22}B_rO_2^+$ [M+H$^+$] 373.0798, found 373.0790.

2-(4-(tert-butyl)phenyl)-5,5-dimethyl-1-phenylhexane-1,4-dione (4an)

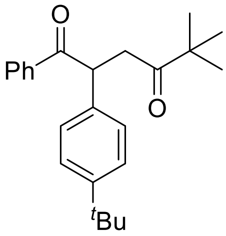

4an

Chromatography Pentane/EA = 50:1 (v/v), 30.1 mg (43%), white solid.

^{1}H NMR (300 MHz, $CDCl_3$) δ 7.95 – 7.92 (m, 2H), 7.43 – 7.11 (m, 7H), 5.04 – 5.00 (m, 1H), 3.69 – 3.59 (m, 1H), 2.77 – 2.70 (m, 1H), 1.19 (s, 9H), 1.08 (s, 9H).
^{13}C NMR (75 MHz, $CDCl_3$) δ 214.4, 199.2, 150.1, 136.6, 135.4, 134.9, 132.7, 129.9, 129.0, 128.9, 128.4, 127.8, 126.0, 47.9, 43.8, 42.4, 34.4, 31.3, 26.5.
HRMS (ESI-TOF): m/z calcd. for $C_{24}H_{31}O_2^+$ [M+H$^+$] 351.2319, found 351.2311.

5,5-dimethyl-1,2-diphenylheptane-1,4-dione (4ao)

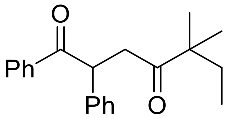

4ao

Chromatography Pentane/EA = 50:1 (v/v), 30.8 mg (50%), white solid.

^{1}H NMR (300 MHz, $CDCl_3$) δ 7.94 – 7.89 (m, 2H), 7.42 – 7.09 (m, 8H), 5.07 – 5.02 (m, 1H), 3.64 – 3.55 (m, 1H), 2.77 – 2.70 (m, 1H), 1.55 – 1.46 (m, 2H), 1.05 (s, 3H), 1.02 (s, 3H), 0.73 – 0.68 (m, 3H).
^{13}C NMR (75 MHz, $CDCl_3$) δ 214.1, 199.1, 138.7, 136.6, 132.8, 129.1, 128.8, 128.4, 128.2, 127.2, 48.4, 47.5, 42.7, 32.7, 23.9, 23.9, 8.9.
HRMS (ESI-TOF): m/z calcd. for $C_{21}H_{25}O_2^+$ [M+H$^+$] 309.1849, found 309.1845.

1-(4-chlorophenyl)-5,5-dimethyl-2-phenylheptane-1,4-dione (4ap)

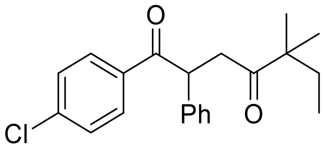

4ap

Chromatography Pentane/EA = 50:1 (v/v), 35.6 mg (52%), white solid.

^{1}H NMR (300 MHz, $CDCl_3$) δ 7.87 – 7.82 (m, 2H), 7.29 – 7.11 (m, 7H), 4.99 – 4.95 (m, 1H), 3.63 – 3.54 (m, 1H), 2.76 – 2.69 (m, 1H), 1.52 – 1.48 (m, 2H), 1.05 (s, 3H), 1.02 (s, 3H), 0.73 – 0.68 (m, 3H).
^{13}C NMR (75 MHz, $CDCl_3$) δ 214.1, 198.0, 139.2, 138.3, 134.9, 130.2, 129.2, 128.7, 128.1, 127.4, 124.6, 48.5, 47.5, 42.7, 32.7, 23.9, 23.9, 8.9.

HRMS (ESI-TOF): m/z calcd. for $C_{21}H_{24}ClO_2^+$ [M+H$^+$] 343.1459, found 343.1450.

1-(3,4-dichlorophenyl)-5,5-dimethyl-2-phenylheptane-1,4-dione (4aq)

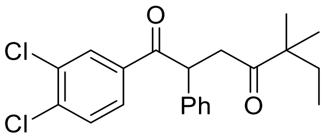

4aq

Chromatography Pentane/EA = 50:1 (v/v), 33.6 mg (45%), white solid.

^{1}H NMR (300 MHz, $CDCl_3$) δ 7.98 (d, *J* = 2.0 Hz, 1H), 7.74 – 7.70– 7.15 (m, 7H), 4.96 – 4.86 (m, 1H), 3.67 – 3.51 (m, 1H), 2.78 – 2.70 (m, 1H), 1.55 – 1.48 (m, 2H), 1.05 (s, 3H), 1.03 (s, 3H), 0.70 (t, *J* = 7.6 Hz, 3H).

^{13}C NMR (75 MHz, $CDCl_3$) δ 214.1, 197.0, 137.7, 137.3, 136.2, 133.1, 130.8, 130.5, 129.3, 128.1, 127.8, 127.6, 48.6, 47.5, 42.8, 32.7, 24.0, 23.9, 8.9.
HRMS (ESI-TOF): m/z calcd. for $C_{21}H_{23}Cl_2O_2^+$ [M+H$^+$] 377.1070, found 377.1077.

4-((3r,5r,7r)-adamantan-1-yl)-2-(4-bromophenyl)-1-phenylbutane-1,4-dione (4ar)

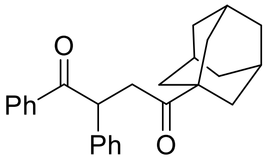

4ar

Chromatography Pentane/EA = 50:1 (v/v), 50.5 mg (68%), white solid.

^1H NMR (300 MHz, $CDCl_3$) δ 8.02 – 8.00 (m, 2H), 7.51 – 7.19 (m, 8H), 5.18 – 5.13 (m, 0.99H), 3.76 – 3.66 (m, 1H), 2.82 – 2.75 (m, 1H), 2.08 – 1.67 (m, 15H).
^{13}C NMR (75 MHz, $CDCl_3$) δ 213.8, 199.1, 138.8, 136.5, 132.7, 129.0, 128.8, 128.4, 128.1, 127.1, 48.3, 46.1, 41.8, 38.2, 36.5, 27.9.
HRMS (ESI-TOF): m/z calcd. for $C_{26}H_{29}O_2^+$ [M+H$^+$] 373.2162, found 373.2160.

4-((3r,5r,7r)-adamantan-1-yl)-2-(4-bromophenyl)-1-phenylbutane-1,4-dione (4as)

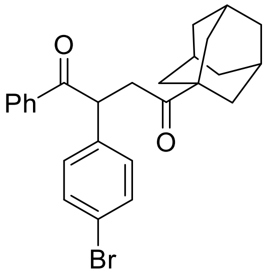

4as

Chromatography Pentane/EA = 50:1 (v/v), 63.0 mg (70%), white solid.

^1H NMR (300 MHz, $CDCl_3$) δ 7.89 – 7.86 (m, 2H), 7.43 – 7.07 (m, 7H), 5.04 – 5.00 (m, 1H), 3.60 – 3.50 (m, 1H), 2.71 – 2.46 (m, 1H), 1.97 – 1.54 (m, 15H).
^{13}C NMR (75 MHz, $CDCl_3$) δ 213.5, 198.8, 137.8, 136.2, 133.0, 129.8, 128.9, 121.2, 47.6, 46.1, 41.6, 38.2, 36.5, 27.9.
HRMS (ESI-TOF): m/z calcd. for $C_{26}H_{28}BrO_2^+$ [M+H$^+$] 451.1267, found 451.1260.

4-((3r,5r,7r)-adamantan-1-yl)-2-(4-(tert-butyl)phenyl)-1-phenylbutane-1,4-dione (4at)

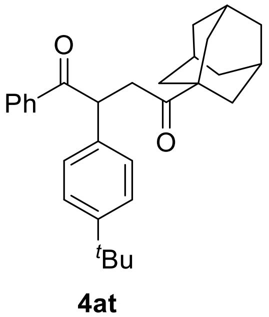

4at

Chromatography Pentane/EA = 50:1 (v/v), 51.4 mg (60%), white solid.

^1H NMR (300 MHz, $CDCl_3$) δ 7.94 – 7.91 (m, 2H), 7.41 – 7.10 (m, 7H), 5.04 – 5.09 (m, 1H), 3.67 – 3.57 (m, 1H), 2.69 – 2.62 (m, 1H), 1.97 – 1.56 (m, 15H), 1.70 (s, 9H).
^{13}C NMR (75 MHz, $CDCl_3$) δ 214.0, 198.3, 150.0, 136.6, 135.5, 132.7, 128.9, 128.4, 127.7, 125.9, 47.7, 46.0, 42.0, 38.2, 36.5, 34.4, 31.2, 27.9.
HRMS (ESI-TOF): m/z calcd. for $C_{30}H_{37}O_2^+$ [M+H$^+$] 429.2788, found 429.2785.

4-((3r,5r,7r)-adamantan-1-yl)-1-(4-chlorophenyl)-2-phenylbutane-1,4-dione (4au)

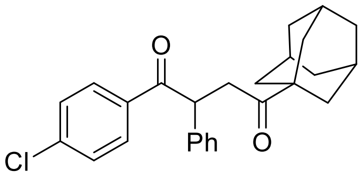

4au

Chromatography Pentane/EA = 50:1 (v/v), 51.1 mg (63%), white solid.

^{1}H NMR (300 MHz, $CDCl_3$) δ 7.84 (d, J = 8.5 Hz, 2H), 7.28 – 7.10 (m, 7H), 4.99 – 4.94 (m, 1H), 3.64 – 3.55 (m, 1H), 2.71 – 2.64 (m, 1H), 1.97 – 1.57 (m, 15H).
^{13}C NMR (75 MHz, $CDCl_3$) δ 213.8, 198.0, 139.1, 138.4, 134.8, 130.2, 129.2, 128.7, 128.1, 127.3, 48.4, 46.1, 41.9, 38.2, 36.5, 27.9.
HRMS (ESI-TOF): m/z calcd. for $C_{26}H_{28}ClO_2^+$ [M+H$^+$] 407.1772, found 407.1778.

4-((3r,5r,7r)-adamantan-1-yl)-1-(4-isopropylphenyl)-2-phenylbutane-1,4-dione (4av)

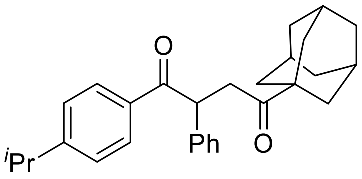

4av

Chromatography Pentane/EA = 50:1 (v/v), 49.2 mg (60%; 95% purity), white solid.

^{1}H NMR (300 MHz, $CDCl_3$) δ 7.85 (d, J = 8.5 Hz, 2H), 7.22 – 7.12 (m, 7H), 5.06 – 5.01 (m, 1H), 3.64 – 3.55 (m, 1H), 2.86 – 2.77 (m, 1H), 2.71 – 2.63 (m, 1H), 1.96 – 1.60 (m, 15H), 1.14 – 1.12 (m, 6H).
^{13}C NMR (75 MHz, $CDCl_3$) δ 213.9, 198.7, 154.2, 139.0, 134.6, 134.3, 129.1, 129.0, 128.1, 127.1, 126.5, 123.8, 48.1, 46.1, 41.9, 38.2, 36.5, 34.2, 27.9, 23.6.
HRMS (ESI-TOF): m/z calcd. for $C_{29}H_{35}O_2^+$ [M+H$^+$] 415.2632, found 415.2628.

4-((1S,2S,4R)-bicyclo[2.2.1]heptan-2-yl)-1,2-diphenylbutane-1,4-dione (4aw)

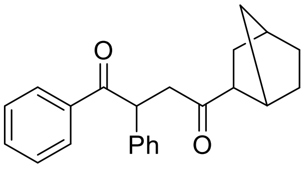

4aw

Chromatography Pentane/EA = 50:1 (v/v), 39.8 mg (60%), viscous liquid.

^{1}H NMR (300 MHz, $CDCl_3$) δ 7.91 – 7.87 (m, 2H), 7.41 – 7.08 (m, 8H), 5.08 – 5.06 (m, 0.5H), 5.05 – 5.03 (m, 0.5H), 3.66 – 3.57 (m, 0.51H), 3.53 – 3.44 (m, 0.5H), 2.81 – 2.73 (m, 0.5H), 2.68 – 2.61 (m, 0.5H), 2.58 – 2.57 (m, 0.5H), 2.44 – 2.35 (m, 1H), 2.27 – 2.25 (m, 0.5H), 2.20 – 2.15 (m, 1H), 1.90 – 1.82 (m, 0.5H), 1.73 – 1.65 (m, 0.5H), 1.49 – 1.46 (m, 0.5H), 1.44 – 1.42 (m, 0.5H), 1.32 – 0.94 (m, 5H).
^{13}C NMR (75 MHz, $CDCl_3$) δ 210.1, 210.1(minor), 199.1, 199.1(minor), 138.7, 138.7(minor), 136.4, 136.4(minor), 132.8, 132.8(minor), 129.1, 128.8,128.8(minor), 128.4, 128.1, 127.2(minor), 54.0(minor), 53.9, 48.8(minor), 48.6, 46.5, 46.4(minor), 40.0, 39.9(minor), 36.1, 36.0, 36.0(minor), 32.5, 32.4(minor), 29.7, 29.7(minor), 28.8.

HRMS (ESI-TOF): m/z calcd. for $C_{23}H_{25}O_2^+$ [M+H$^+$] 333.1849, found 333.1845.

4-((1S,2S,4R)-bicyclo[2.2.1]heptan-2-yl)-1-(4-chlorophenyl)-2-phenylbutane-1,4-dione (4ax)

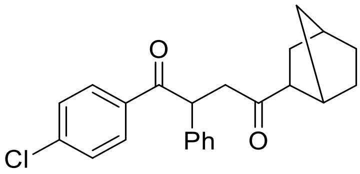

4ax

Chromatography Pentane/EA = 50:1 (v/v), 47.5 mg (65%), viscous liquid.

^{1}H NMR (300 MHz, $CDCl_3$) δ 7.84 – 7.81 (m, 2H), 7.27– 7.12 (m, 7H), 5.05 – 4.99 (m, 0.50H), 4.97 – 4.95 (m, 0.5H), 3.65 – 3.56 (m, 0.5H), 3.52 – 3.42 (m, 0.5H), 2.80 – 2.73 (m, 0.5H), 2.67 – 2.60 (m, 0.50H), 2.57 – 2.56 (m, 0.50H), 2.43 – 2.34 (m, 1H), 2.27 – 2.25 (m, 0.5H), 2.20 – 2.15 (m, 1H), 1.85 – 0.94 (m, 7H).
^{13}C NMR (75 MHz, $CDCl_3$) δ 210.1, 210.0, 197.9, 197.9, 139.2, 139.2, 138.3, 138.3, 134.8, 134.7, 130.2, 130.2, 129.2, 128.7, 128.0, 127.4, 53.9, 53.8, 48.9, 48.6, 46.5, 46.3, 40.1, 39.9, 36.1, 36.0, 36.0, 36.0, 32.5, 32.4, 29.7, 29.7, 28.8.
HRMS (ESI-TOF): m/z calcd. for $C_{23}H_{24}ClO_2^+$ [M+H$^+$] 367.1459, found 367.1450.

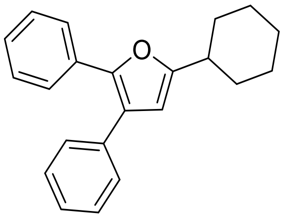

8a

5-cyclohexyl-2,3-diphenylfuran (8a)

Chromatography Pentane/EA = 50:1 (v/v), 26.6 mg (88%), yellow solid.

^{1}H NMR (300 MHz, $CDCl_3$) δ 7.44 – 7.07 (m, 10H), 6.04 (s, 1H), 2.65 – 1.16 (m, 11H).

^{13}C NMR (75 MHz, $CDCl_3$) δ 160.1, 146.2, 134.9, 131.6, 128.6, 128.5, 128.3, 126.9, 126.8, 125.9, 122.7, 107.4, 37.2, 31.5, 26.1, 25.9.
HRMS (ESI-TOF): m/z calcd. for $C_{22}H_{23}O^+$ [M+H$^+$] 303.1743, found 303.1740.

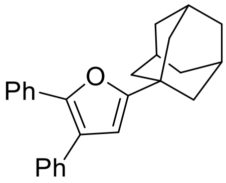

8a'

5-((3r,5r,7r)-adamantan-1-yl)-2,3-diphenylfuran (8a')

Chromatography Pentane/EA = 50:1 (v/v), 26.5 mg (75%), white solid.

^{1}H NMR (300 MHz, $CDCl_3$) δ 7.92 (s, 1H), 7.31 – 7.04 (m, 10H), 6.04 (d, *J* = 2.9 Hz, 1H), 2.06 – 2.00 (m, 3H), 1.90 (d, *J* = 2.9 Hz, 6H), 1.76 – 1.66 (m, 6H).
^{13}C NMR (75 MHz, $CDCl_3$) δ 142.9, 136.9, 133.8, 128.6, 128.3, 128.2, 127.3, 126.3, 126.0, 125.4, 121.5, 105.0, 42.8, 41.3, 36.7, 33.3, 28.6, 28.5.

HRMS (ESI-TOF): m/z calcd. for $C_{26}H_{27}O^+$ [M+H$^+$] 355.2056, found 355.2059.

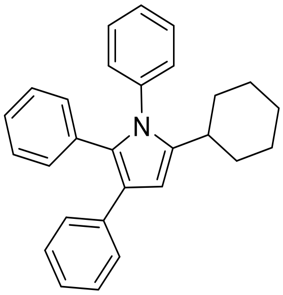

8b

5-cyclohexyl-1,2,3-triphenyl-1H-pyrrole **(8b)**

Chromatography Pentane/EA = 20:1 (v/v), 30.2 mg (80%), white solid.

^{1}H NMR (300 MHz, $CDCl_3$) δ 7.22 – 6.90 (m, 15H), 6.23 (s, 1H), 2.37 – 1.01 (m, 11H).
^{13}C NMR (75 MHz, $CDCl_3$) δ 141.1, 138.8, 136.7, 133.0, 131.2, 129.9, 129.1, 128.5, 128.1, 128.0, 127.7, 127.4, 126.3, 125.0, 122.2, 104.8, 35.7, 34.0, 26.6, 26.1.

HRMS (ESI-TOF): m/z calcd. for $C_{28}H_{28}N^+$ [M+H $^+$] 378.2216, found 378.2210.

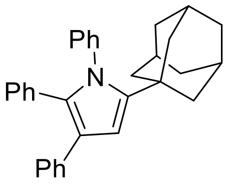

8b'

5-((3r,5r,7r)-adamantan-1-yl)-1,2,3-triphenyl-1H-pyrrole **(8b')**

Chromatography Pentane/EA = 50:1 (v/v), 34.3 mg (80%), yellow solid.

^{1}H NMR (300 MHz, $CDCl_3$) δ 7.18 – 6.95 (m, 15H), 6.29 (s, 1H), 1.82 – 1.43 (m, 15H).
^{13}C NMR (75 MHz, $CDCl_3$) δ 144.5, 140.8, 136.5, 133.2, 132.7, 131.7, 131.1, 129.0, 128.2, 127.9, 127.7, 127.6, 126.6, 125.3, 124.9, 121.0, 105.6, 42.4, 36.6, 35.3, 28.6.
HRMS (ESI-TOF): m/z calcd. for $C_{32}H_{32}O^+$ [M+H $^+$] 430.2529, found 430.2520.

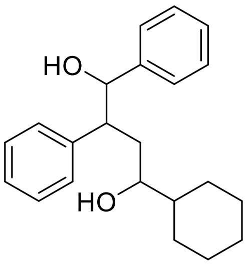

8c

4-cyclohexyl-1,2-diphenylbutane-1,4-diol **(8c)**

Chromatography Pentane/EA = 20:1 (v/v), 29.2 mg (90%), white solid.

^{1}H NMR (400 MHz, $CDCl_3$) δ 7.26 – 7.09 (m, 10H), 4.71 - 3.17 (m, 1H), 3.19 – 2.86 (m, 2H), 1.96 – 0.72 (m, 15H).
^{13}C NMR (75 MHz, $CDCl_3$) δ 142.3, 140.8, 129.0, 128.8, 128.6, 128.5, 128.2, 127.7, 127.6, 126.9, 126.9, 78.7, 78.1, 74.5, 73.5, 51.1, 50.5, 44.2, 42.7, 36.7, 36.2, 29.4, 28.9, 27.9, 26.5, 26.4, 26.4, 26.3, 26.1, 26.0.
HRMS (ESI-TOF): m/z calcd. for $C_{22}H_{29}O_2^+$ [M+H $^+$] 325.2162, found 325.2170.

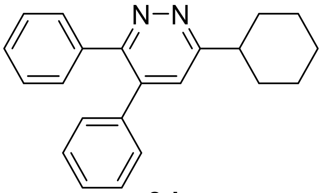

8d

6-cyclohexyl-3,4-diphenylpyridazine (8d)

Chromatography Pentane/EA = 20:1 (v/v), 26.0 mg (83%), yellow solid.

^{1}H NMR (300 MHz, $CDCl_3$) δ 7.37 – 7.09 (m, 11H), 3.05 – 2.97 (m, 1H), 2.09 – 1.15 (m, 10H).
^{13}C NMR (75 MHz, $CDCl_3$) δ 180.7, 166.1, 157.9, 139.4, 137.2, 136.8, 130.0, 129.0, 128.6, 128.5, 128.5, 128.0, 125.7, 44.3, 42.9, 32.7, 28.8, 26.4, 25.9, 25.35.

HRMS (ESI-TOF): m/z calcd. for $C_{22}H_{23}N_2{}^+$ [M+H $^+$] 315.1856, found 315.1848.

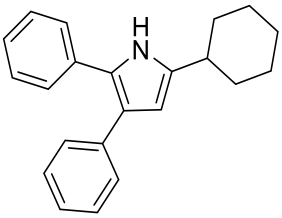

8e

5-cyclohexyl-2,3-diphenyl-1H-pyrrole (8e)

Chromatography Pentane/EA = 50:1 (v/v), 25.2 mg (84%), yellow solid.

^{1}H NMR (300 MHz, $CDCl_3$) δ 7.89 (s, 1H), 7.30 – 7.05 (m, 10H), 6.03 (s, 1H), 2.59 – 2.44 (m, 1H), 2.03 – 1.14 (m, 10H).
^{13}C NMR (75 MHz, $CDCl_3$) δ 138.6, 136.9, 133.7, 128.6, 128.6, 128.3, 128.2, 127.2, 126.3, 125.5, 121.7, 106.1, 36.7, 33.1, 26.2, 26.1.

HRMS (ESI-TOF): m/z calcd. for $C_{22}H_{24}N^+$ [M+H $^+$] 302.1904, found 302.1900.

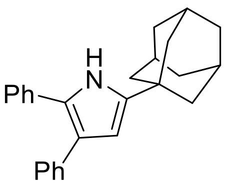

8e'

5-((3r,5r,7r)-adamantan-1-yl)-1,2,3-triphenyl-1H-pyrrole (8e‘)

Chromatography Pentane/EA = 50:1 (v/v), 22.9 mg (65%), white solid.

^{1}H NMR (300 MHz, $CDCl_3$) δ 7.92 (s, 1H), 7.30 – 7.04 (m, 10H), 6.04 (d, J = 2.9 Hz, 1H), 2.06 – 1.65 (m, 15H).
^{13}C NMR (75 MHz, $CDCl_3$) δ 143.0, 137.0, 133.8, 128.6, 128.3, 128.2, 127.3, 126.3, 126.0, 125.4, 121.5, 105.0, 42.8, 41.3, 36.7, 33.3, 28.6, 28.5.

HRMS (ESI-TOF): m/z calcd. for $C_{26}H_{28}N^+$ [M+H $^+$] 354.2216, found 354.2210.

5. Mechanistic investigation

5.1 Control Experiments and Mechanistic Studies

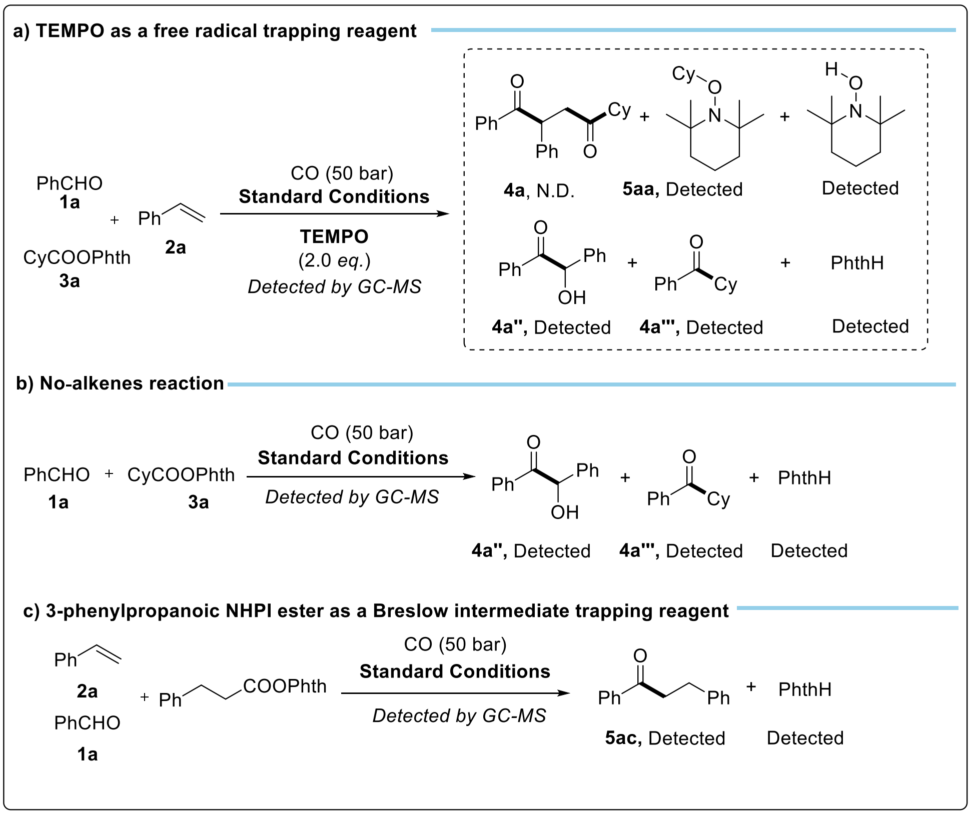

Scheme 1 Control Experiments and Mechanistic Studies

Base the standard conditions, a series of control experiments were conducted (Scheme 1, eqs a–c). First, the addition of 2,2,6,6-tetramethylpiperidin-1-oxyl (TEMPO) as a radical scavenger completely suppressed the formation of product 4a under standard conditions (**Figure 1** and **Scheme 1a**). Instead, the benzoin condensation (**4a''**) and aldehyde alkylation (**4a'''**), cyclohexyl–TEMPO adduct **5aa** and PhthH was detected by GC–MS, supporting the involvement of alkyl radical intermediates.[2] Next, a control experiment without alkenes to explore main by-product (**Figure 2** and **Scheme 1b**), with the benzoin condensation (**4a''**) and aldehyde alkylation (**4a'''**) detected by GC–MS analysis.[3] In addition, treatment of the reaction with 3-phenylpropanoic NHPI ester, employed to probe the involvement of the Breslow intermediate, led to the formation of adduct **5ac** (**Figure 3** and **Scheme 1c**).[4] Collectively, these results support a radical-based single-electron-transfer (SET) pathway.

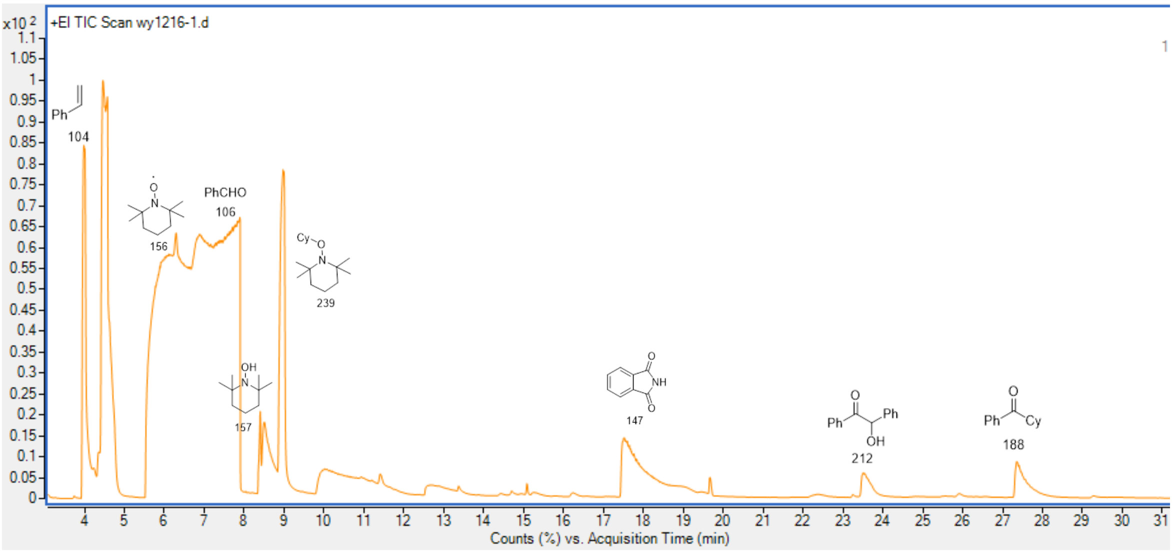

Figure 1 GC-MS of the reaction solvent (TEMPO as a radical scavenger)

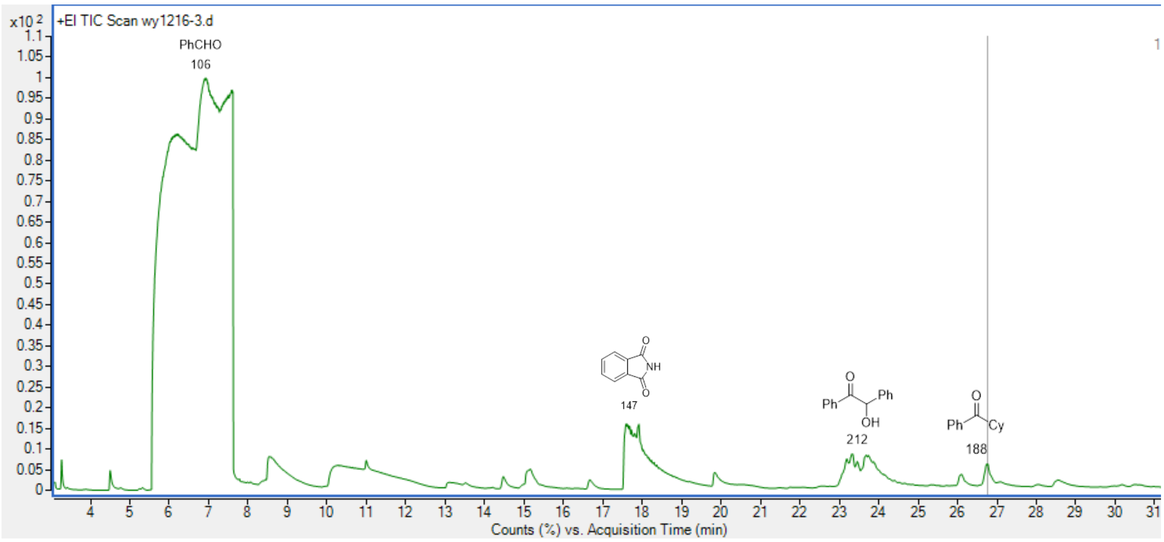

Figure 2 GC-MS of the reaction solvent (without alkenes)

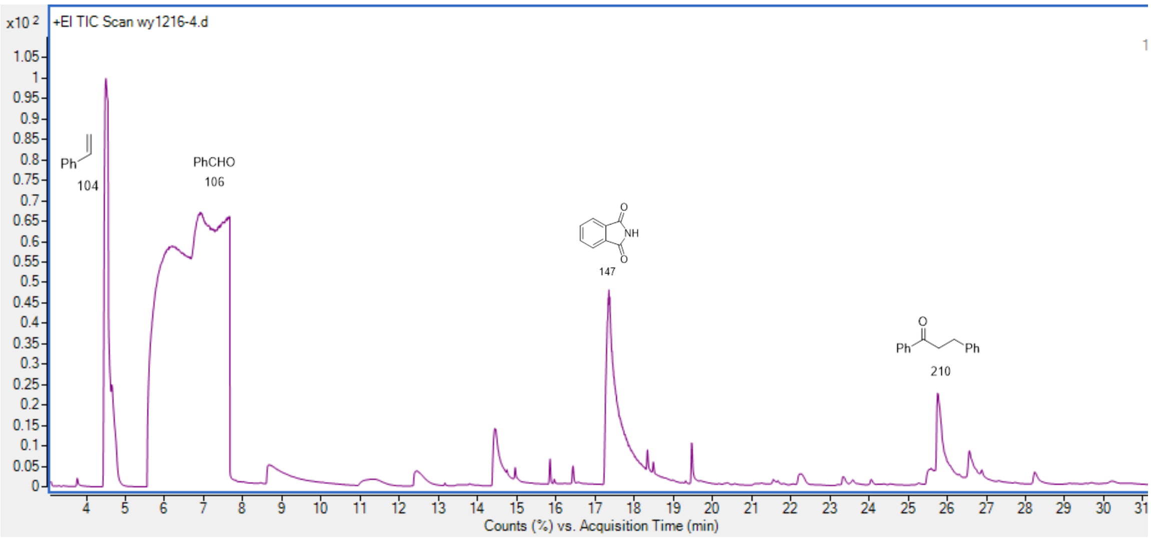

Figure 3 GC-MS of the reaction solvent (use 3-phenylpropanoic NHPI ester)

6. References

[1] L.M. Kammer, A. Rahman and T. Opatz, *Molecules.*, 2018, **23**, 764.

[2] a) D. Liu, Y. Li, X. Qi, C. Liu, Y. Lan, A. Lei, *Org. Lett.*, 2015, **17**, 998 – 1001; b) F. Zhao, X.-W. Gu, R. Franke, X.-F. Wu, *Angew. Chem. Int. Ed.*, 2022, **61**, e202214812.

[3] Z. M. Rubanov, V. V. Levin and A. D. Dilman, *Org. Lett.,* 2023, **25**, 8751 – 8755.

[4] T. Song, Z. Ma, Y. Yang, *ChemCatChem.*, 2019, **11**, 1313 – 1319.

7. NMR Spectra

4a ^{1}H NMR (300 MHz, $CDCl_3$)

250928.332.10.fid — Mao-Lin Yang, YML-5-12 — Au1H CDCl3 {C:\Bruker\TopSpin3.6.2} 2509 32

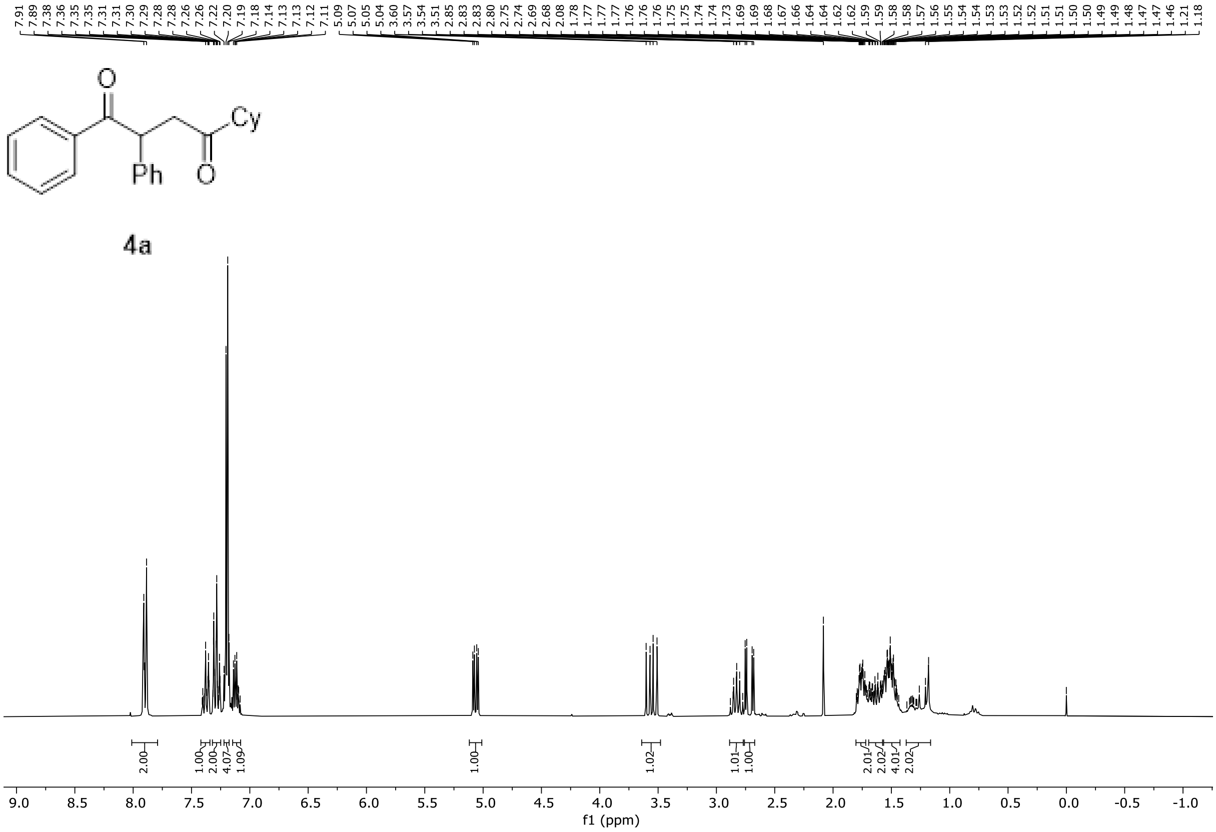

4a ^{13}C NMR (75 MHz, $CDCl_3$)

250928.332.11.fid — Mao-Lin Yang, YML-5-12 — Au13C CDCl3 {C:\Bruker\TopSpin3.6.2} 2509 32

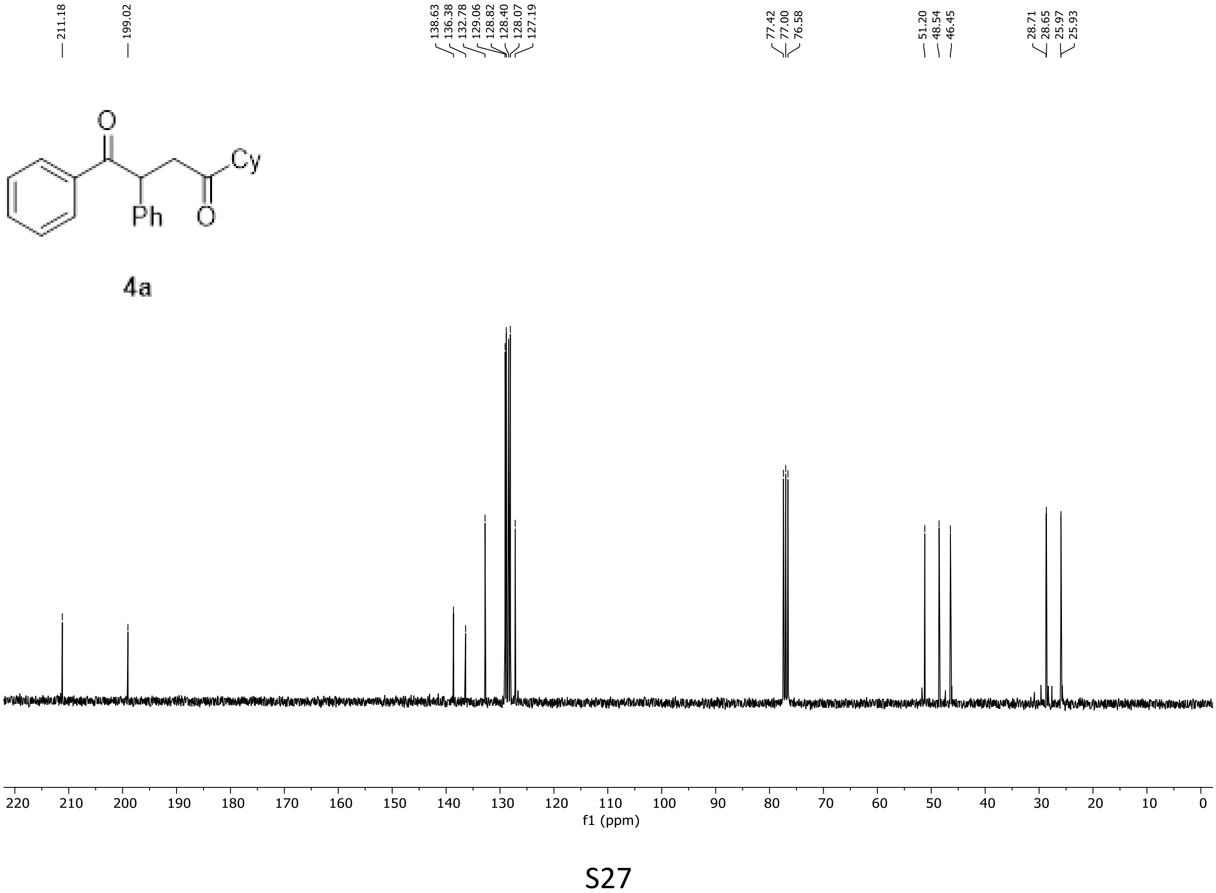

4b ^{1}H NMR (400 MHz, $CDCl_3$)

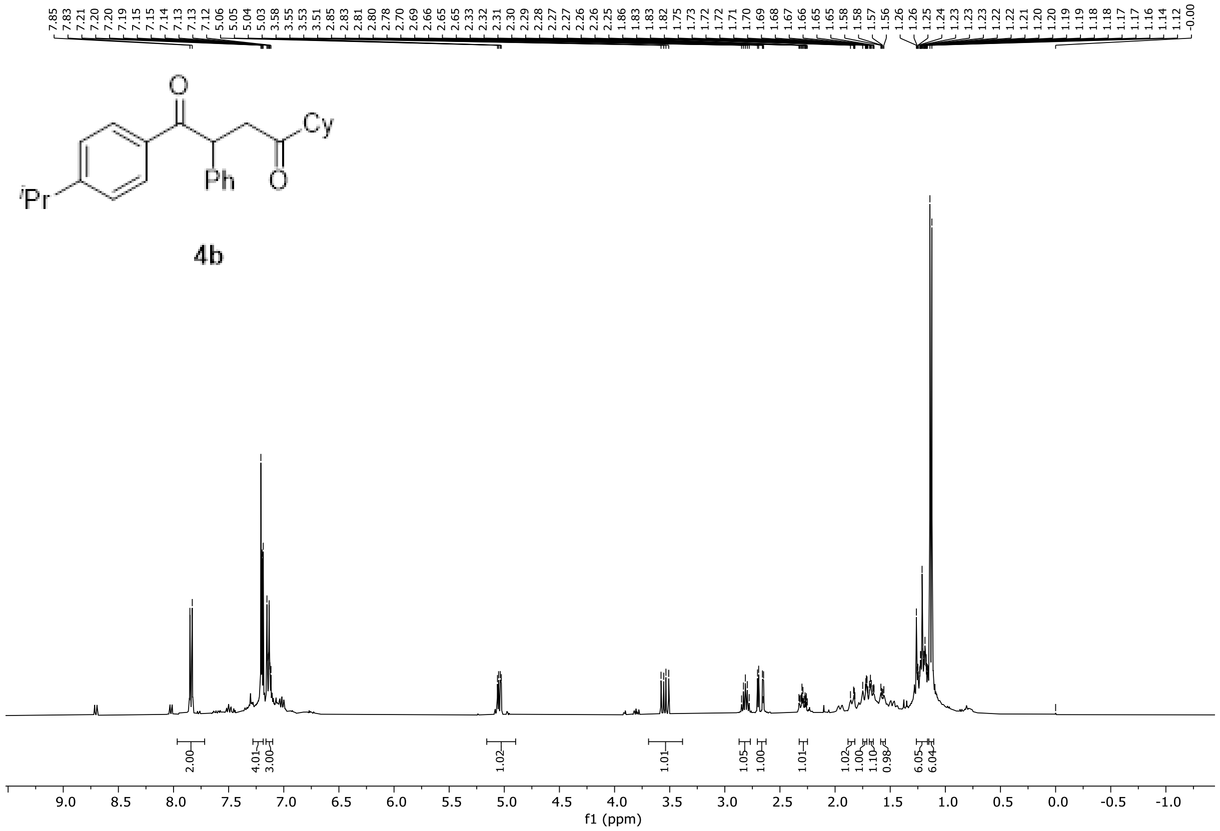

4b ^{13}C NMR (101 MHz, $CDCl_3$)

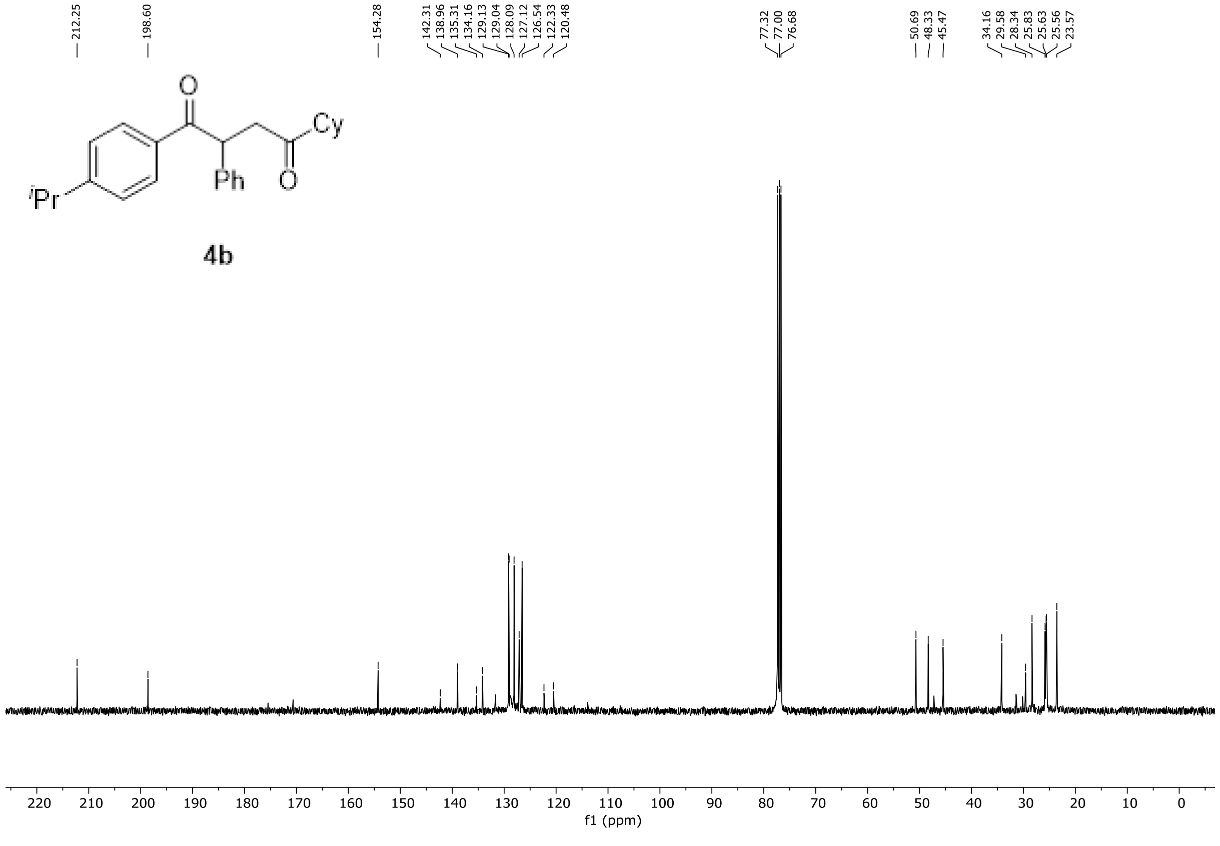

4c ^{1}H NMR (300 MHz, $CDCl_3$)

251006.f326.10.fid — Mao-Lin Yang YML-5-33 — Au1H CDCl3 {C:\Bruker\TopSpin3.6.2} 2510 26

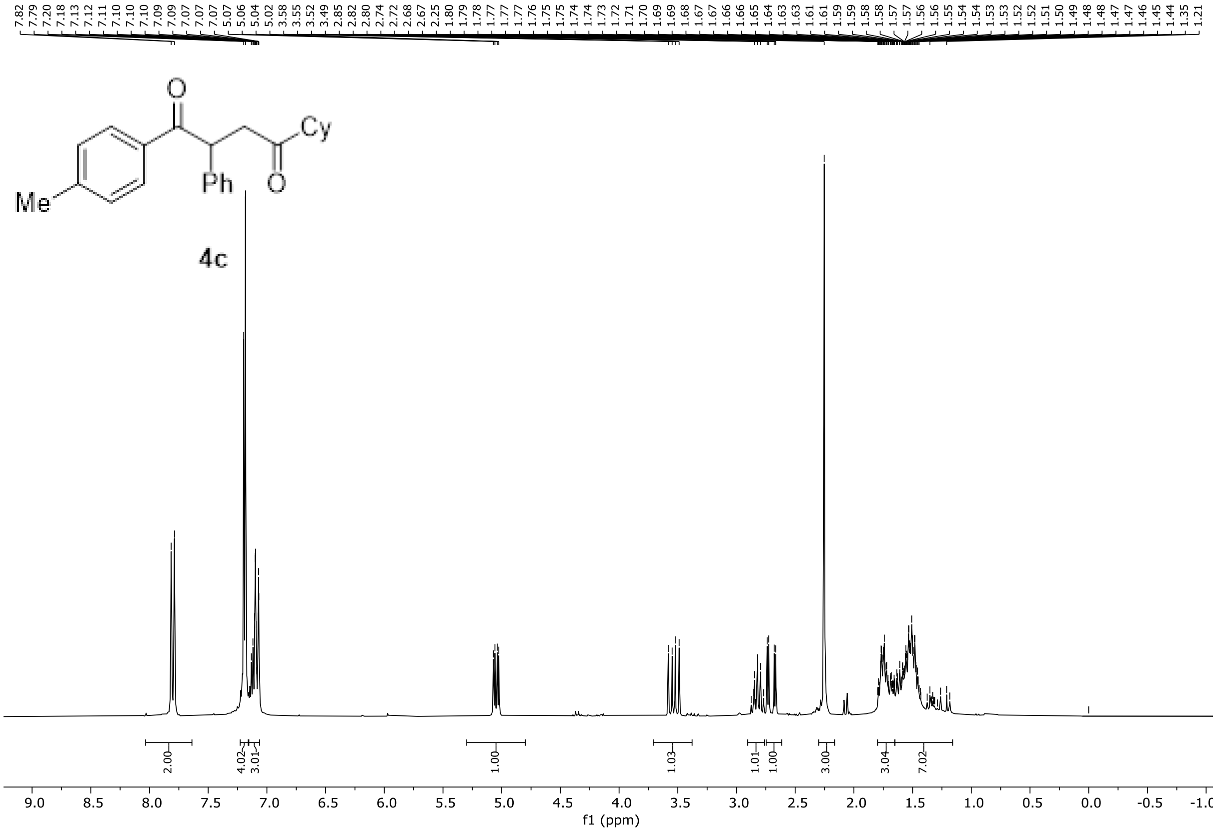

4c ^{13}C NMR (75 MHz, $CDCl_3$)

251006.f326.11.fid — Mao-Lin Yang YML-5-33 — Au13C CDCl3 {C:\Bruker\TopSpin3.6.2} 2510 26

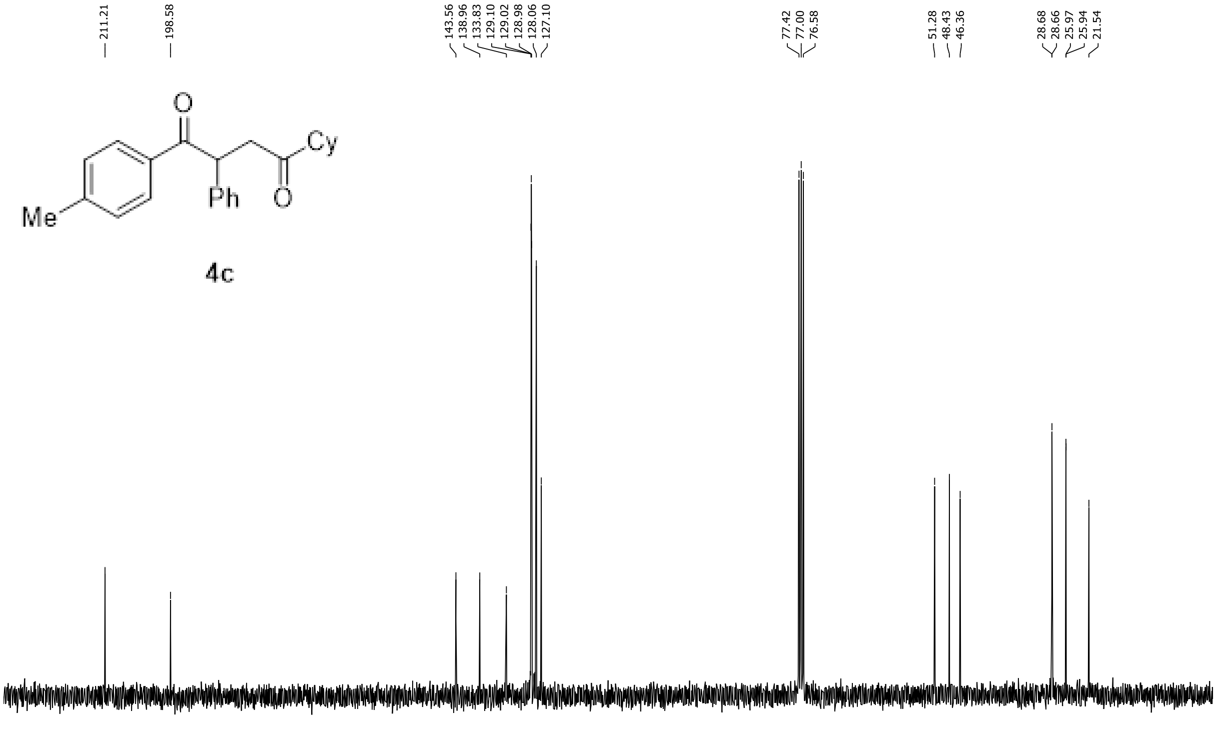

4d ^{1}H NMR (300 MHz, $CDCl_3$)

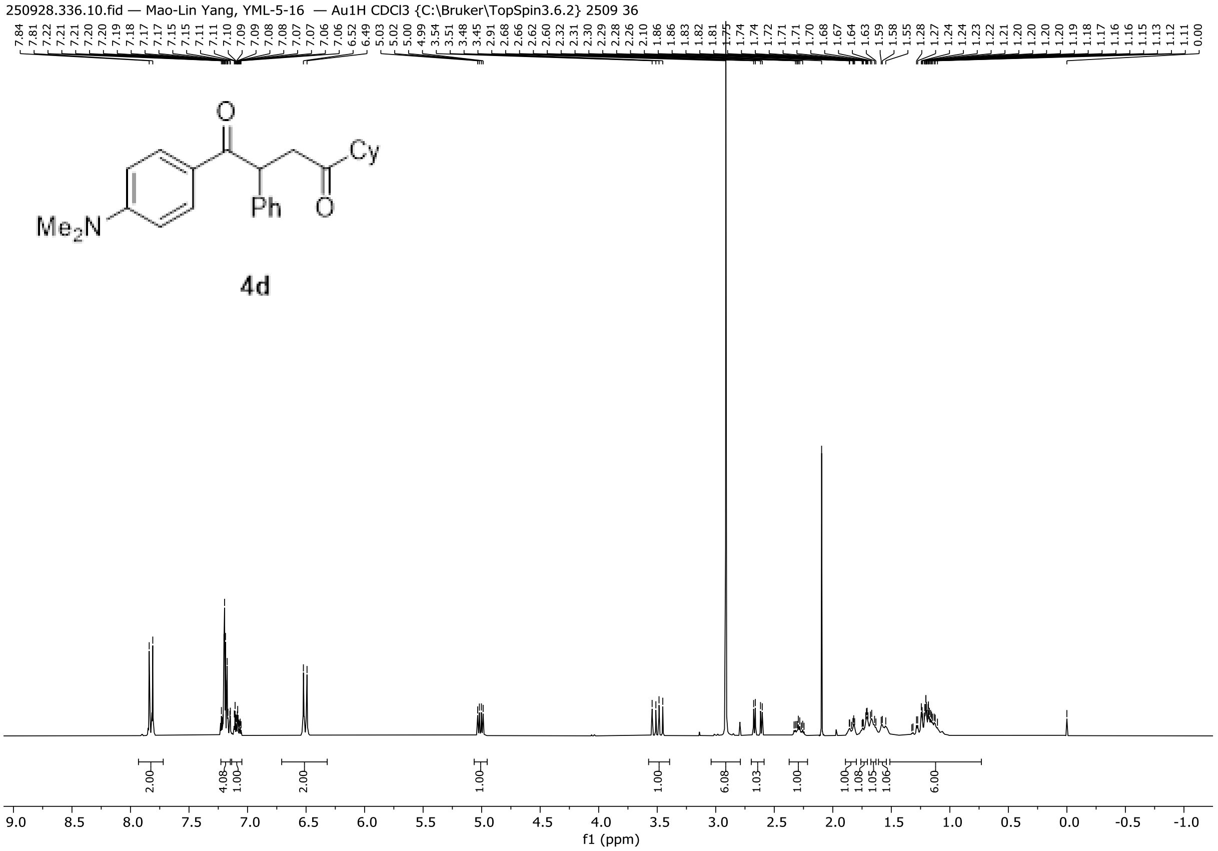

4d ^{13}C NMR (75 MHz, $CDCl_3$)

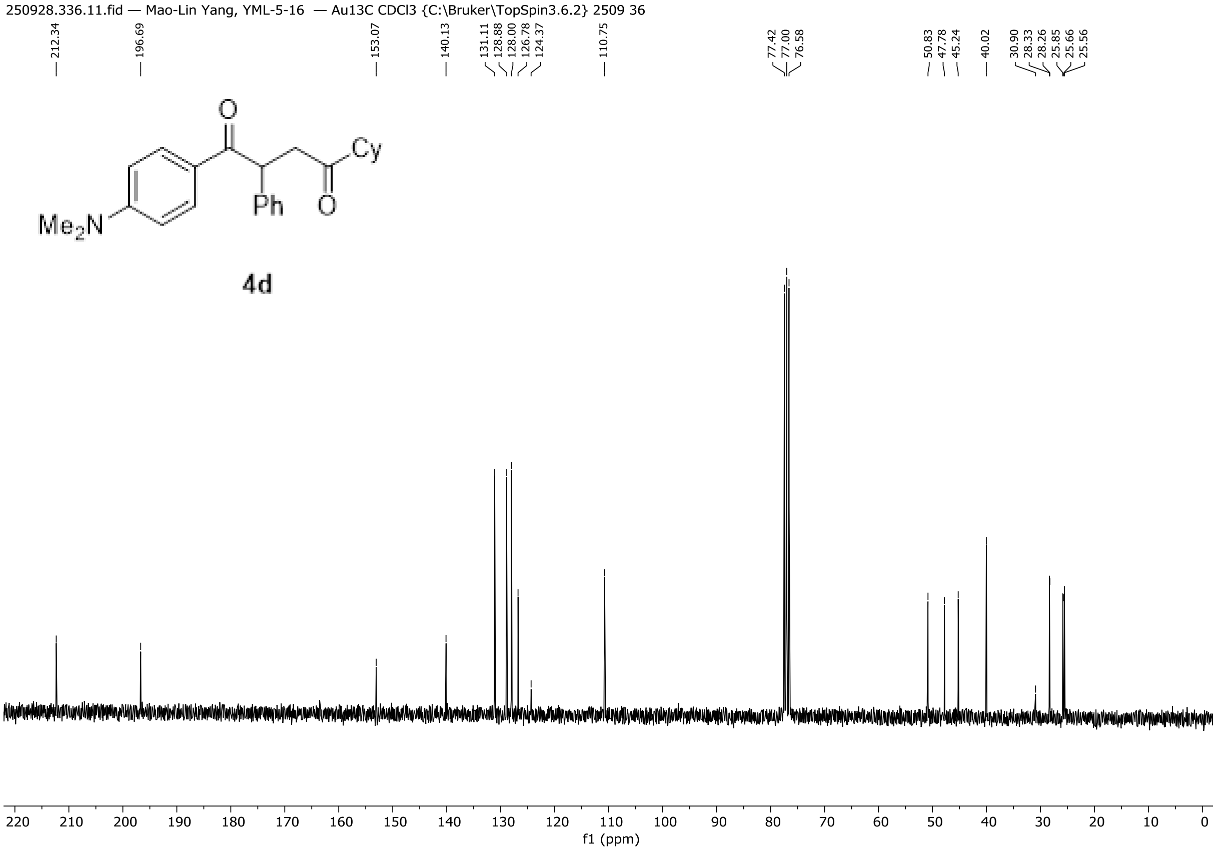

4e ^{1}H NMR (300 MHz, $CDCl_3$)

251006.302.10.fid — Lin-Mao Yang YML-5-25 — Au1H CDCl3 {C:\Bruker\TopSpin3.6.2} 2510 2

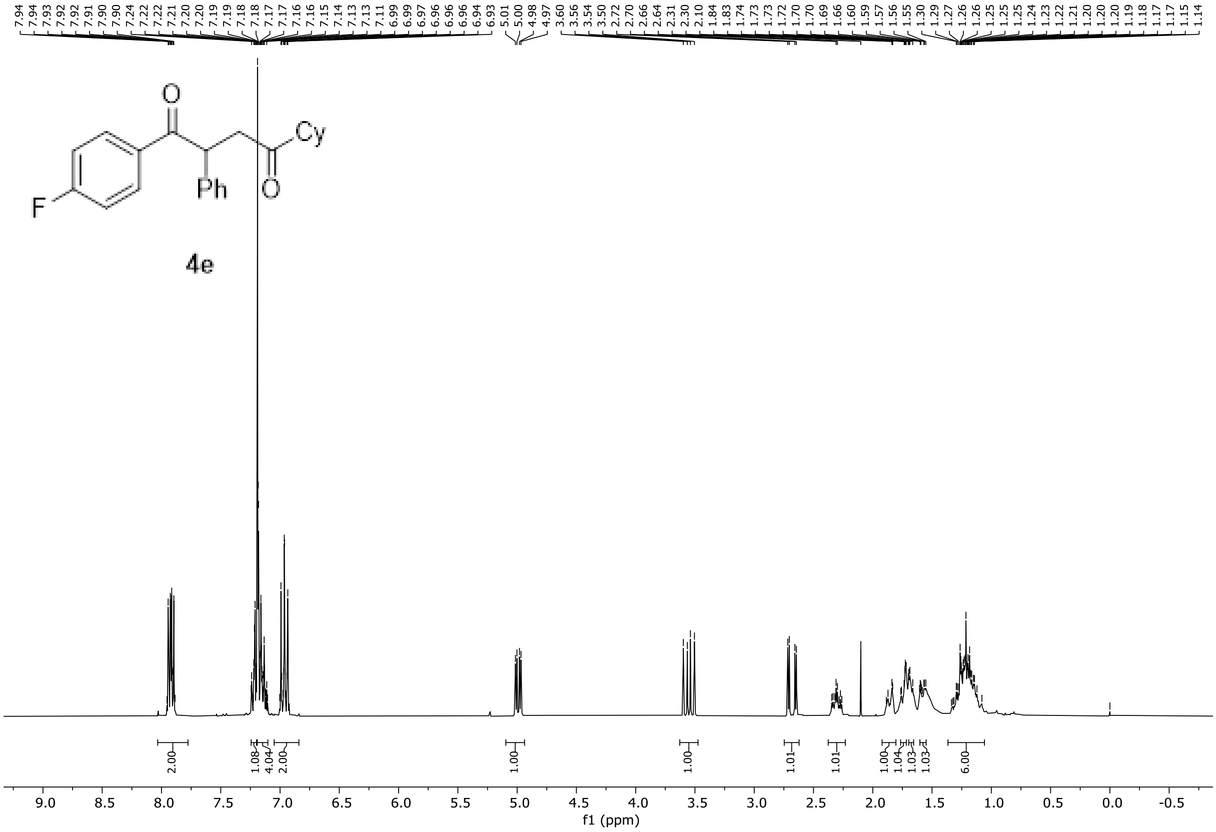

4e ^{13}C NMR (75 MHz, $CDCl_3$)

251006.302.11.fid — Lin-Mao Yang YML-5-25 — Au13C CDCl3 {C:\Bruker\TopSpin3.6.2} 2510 2

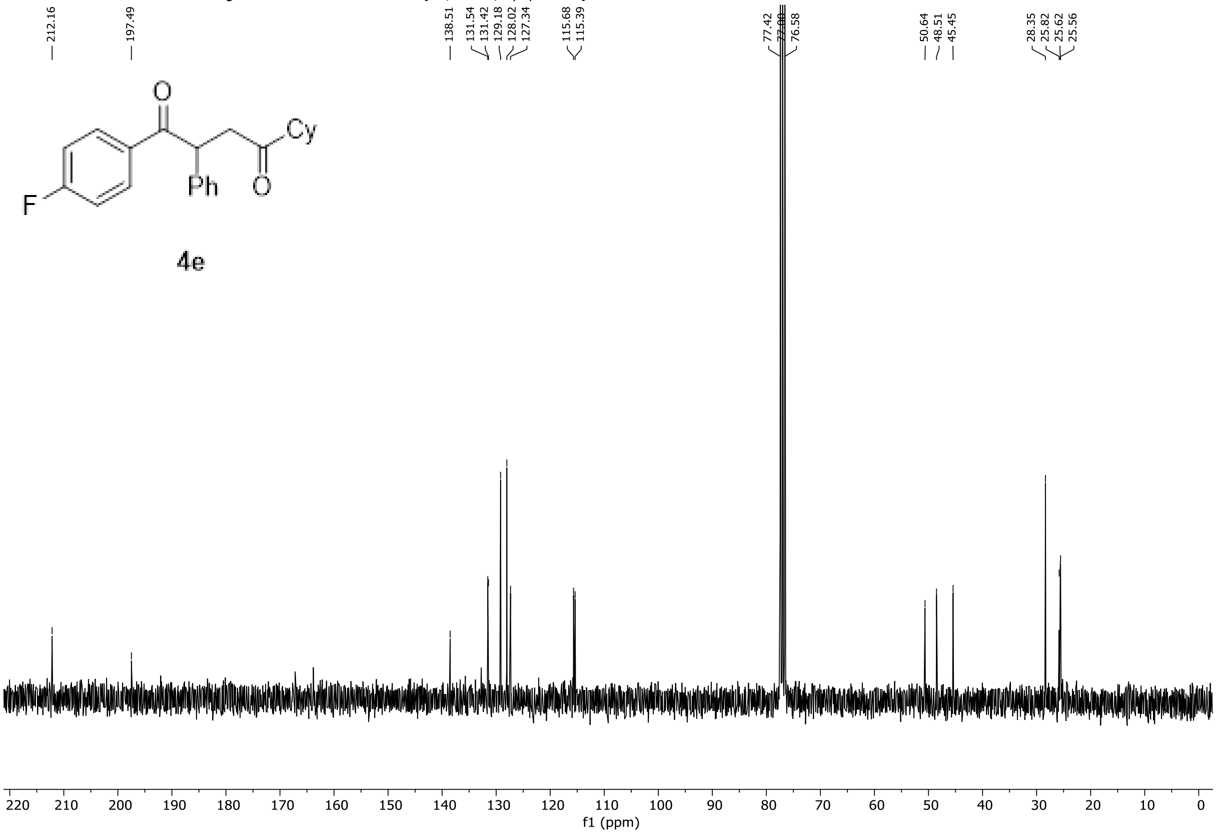

4e ^{19}F NMR (282 MHz, $CDCl_3$)

251006.302.12.fid — Lin-Mao Yang YML-5-25 — Au19F CDCl3 {C:\Bruker\TopSpin3.6.2} 2510 2

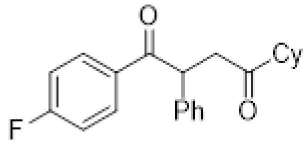

4e

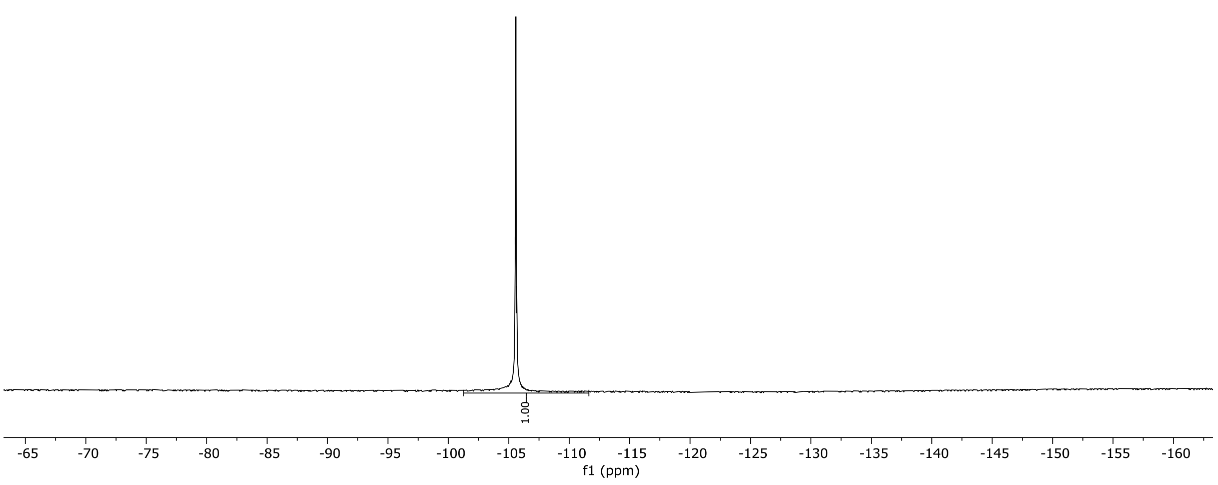

4f ^{1}H NMR (400 MHz, $CDCl_3$)

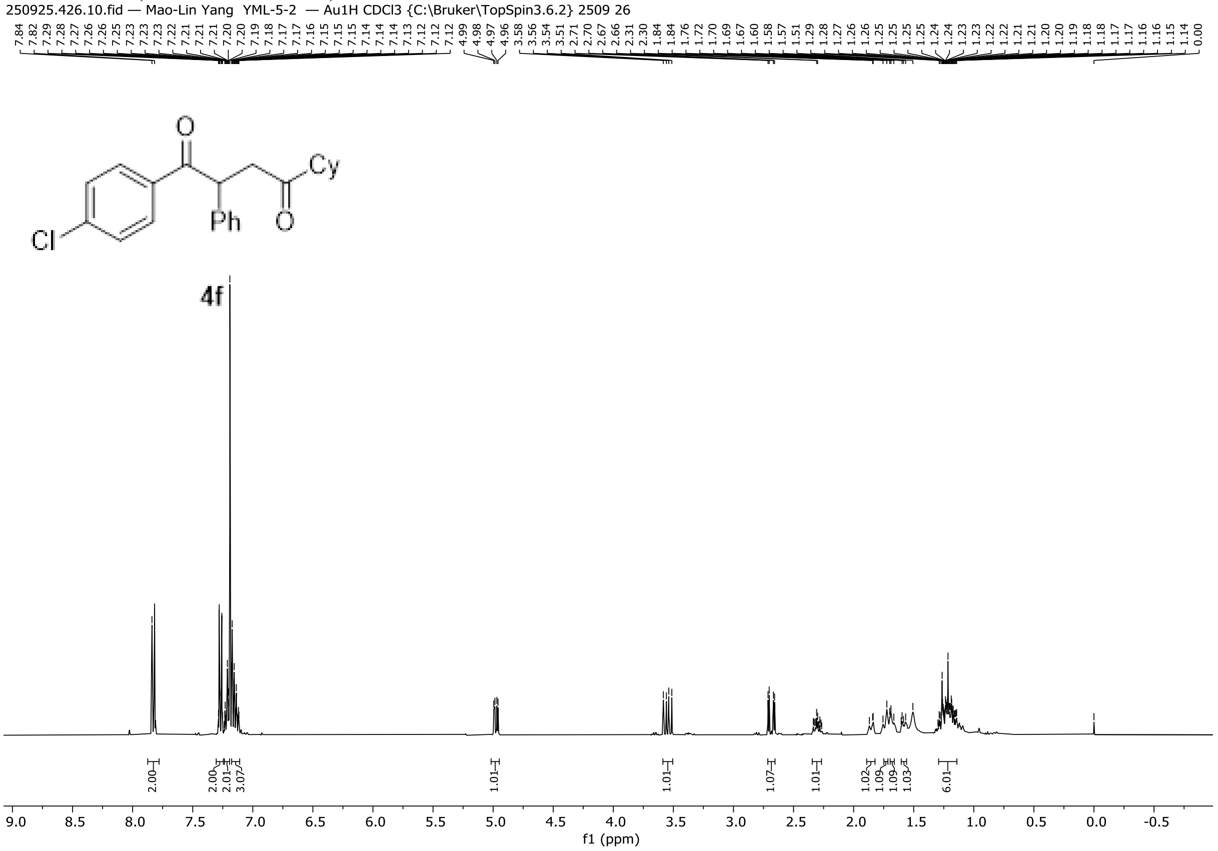

4f ^{13}C NMR (101 MHz, $CDCl_3$)

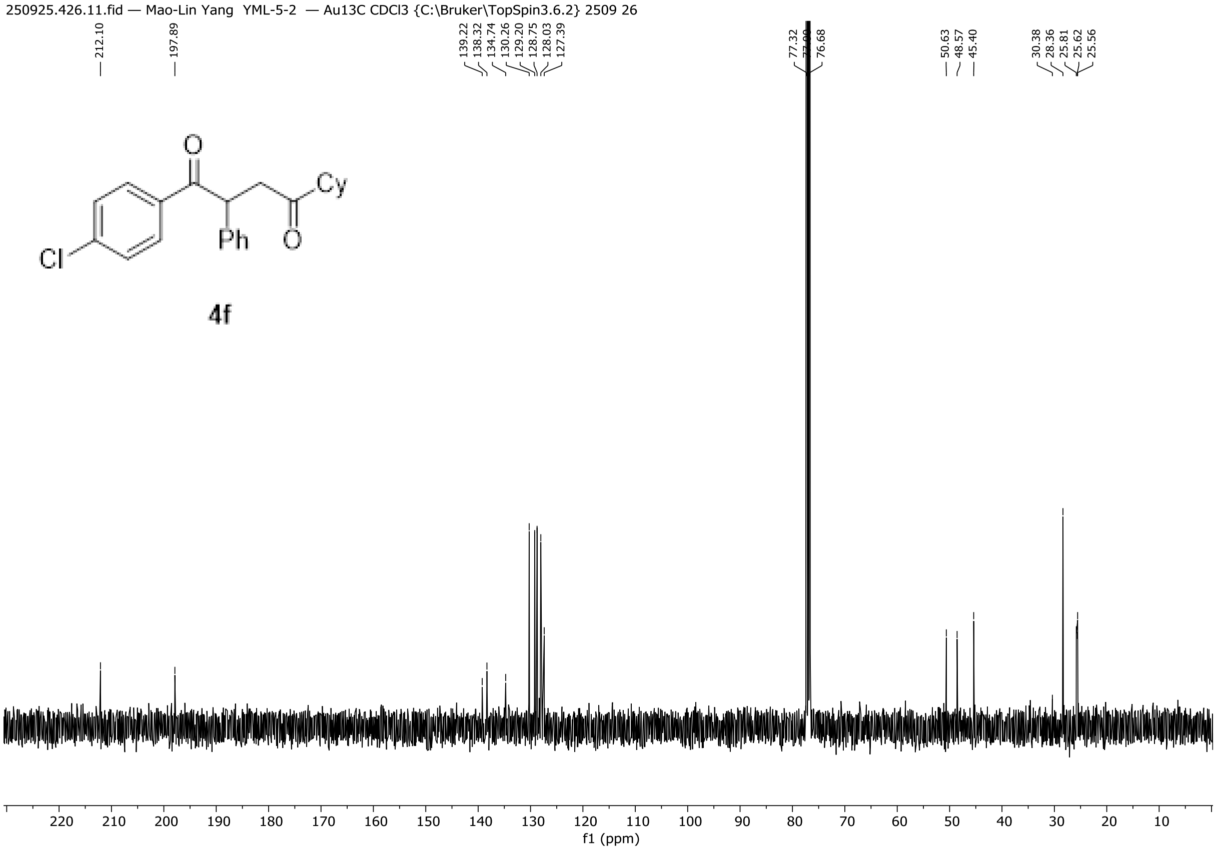

4g ^{1}H NMR (300 MHz, $CDCl_3$)

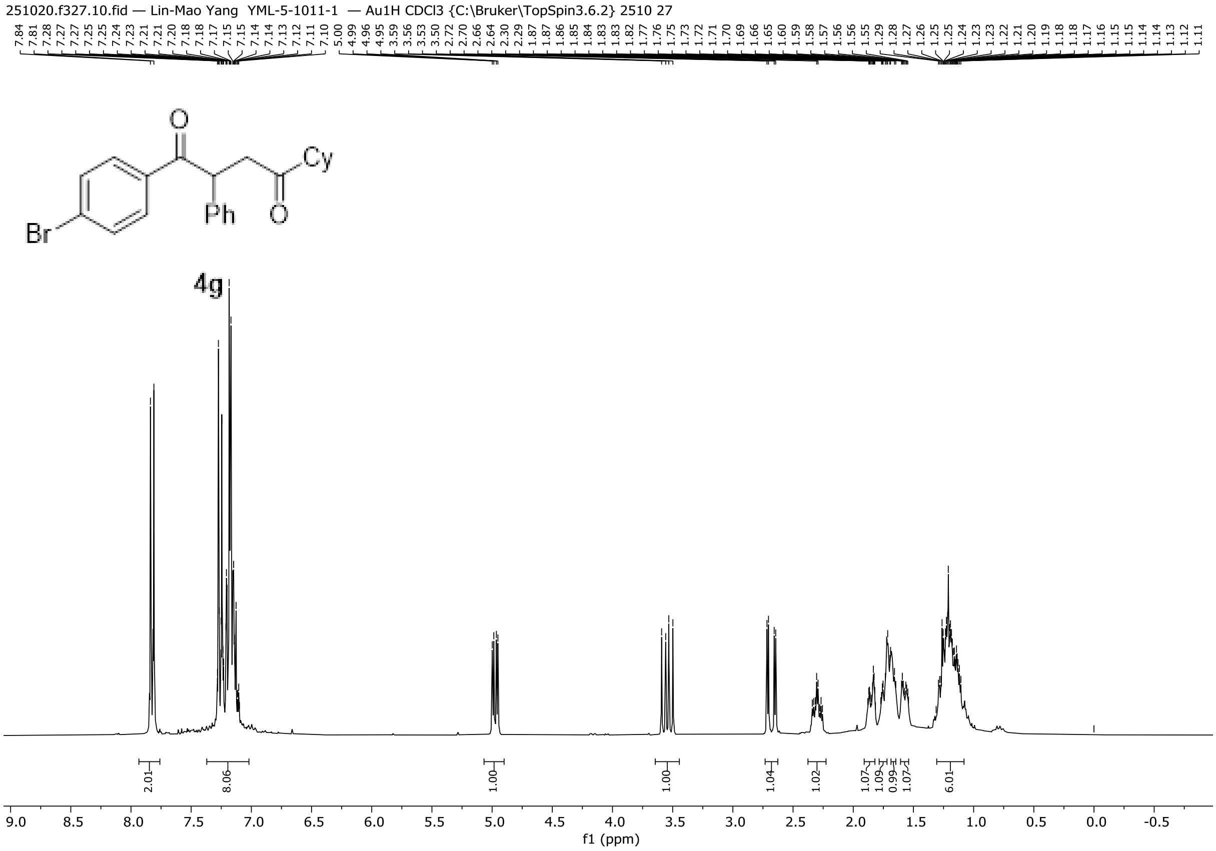

4g ^{13}C NMR (75 MHz, $CDCl_3$)

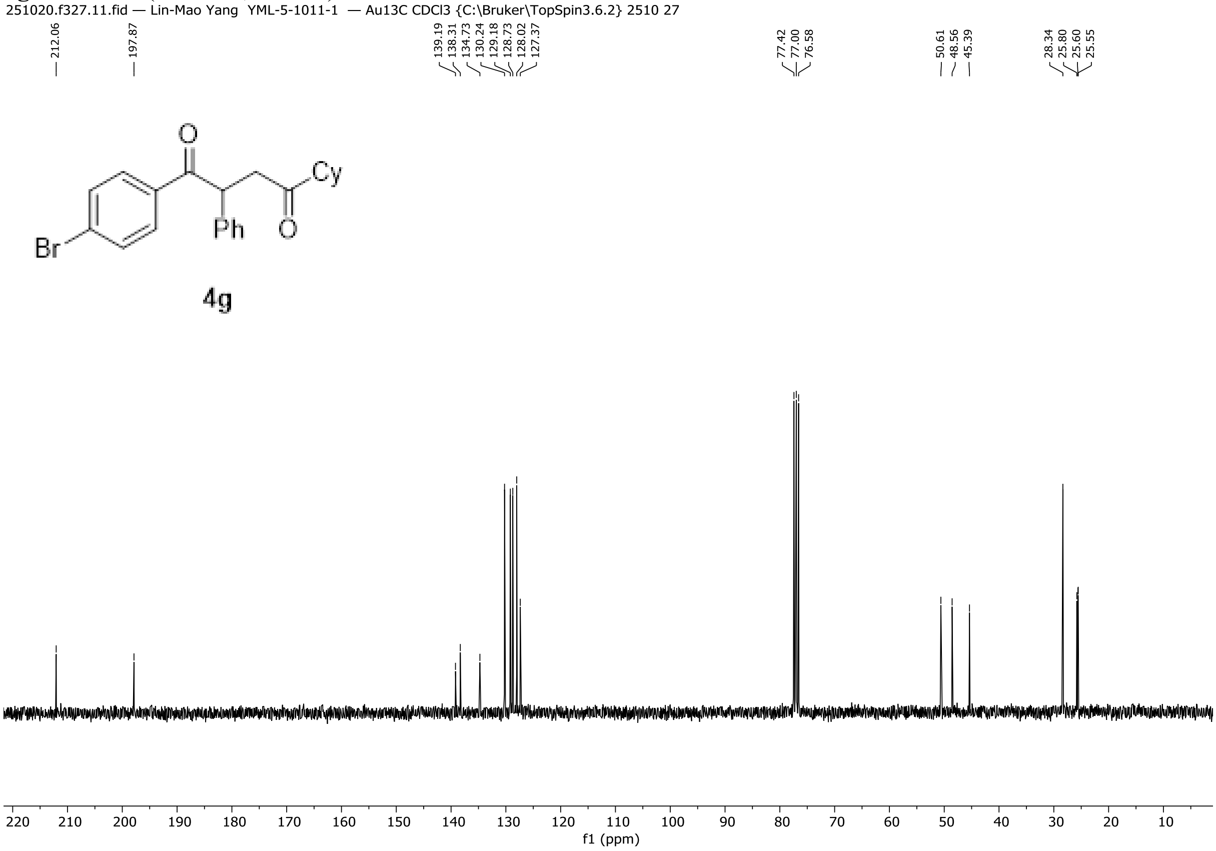

4h ^{1}H NMR (400 MHz, $CDCl_3$)

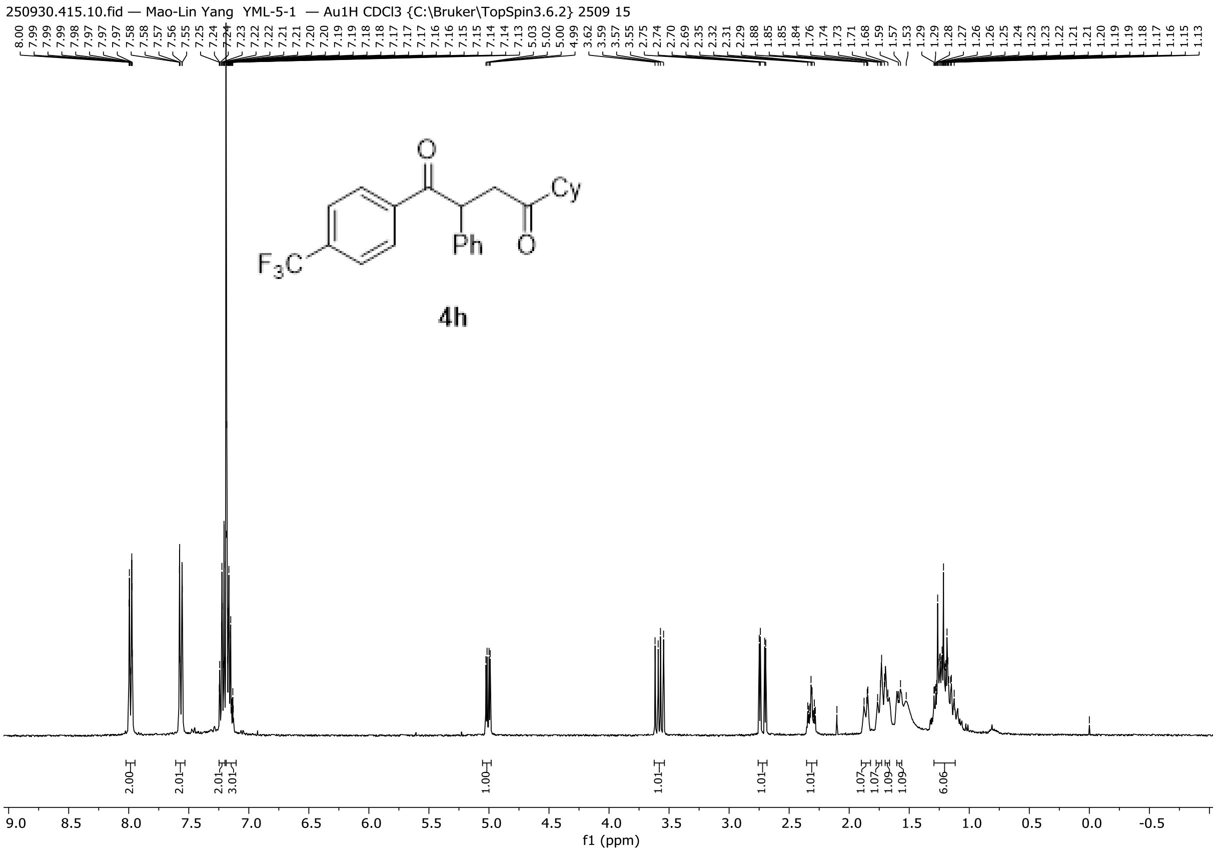

4h ^{13}C NMR (101 MHz, $CDCl_3$)

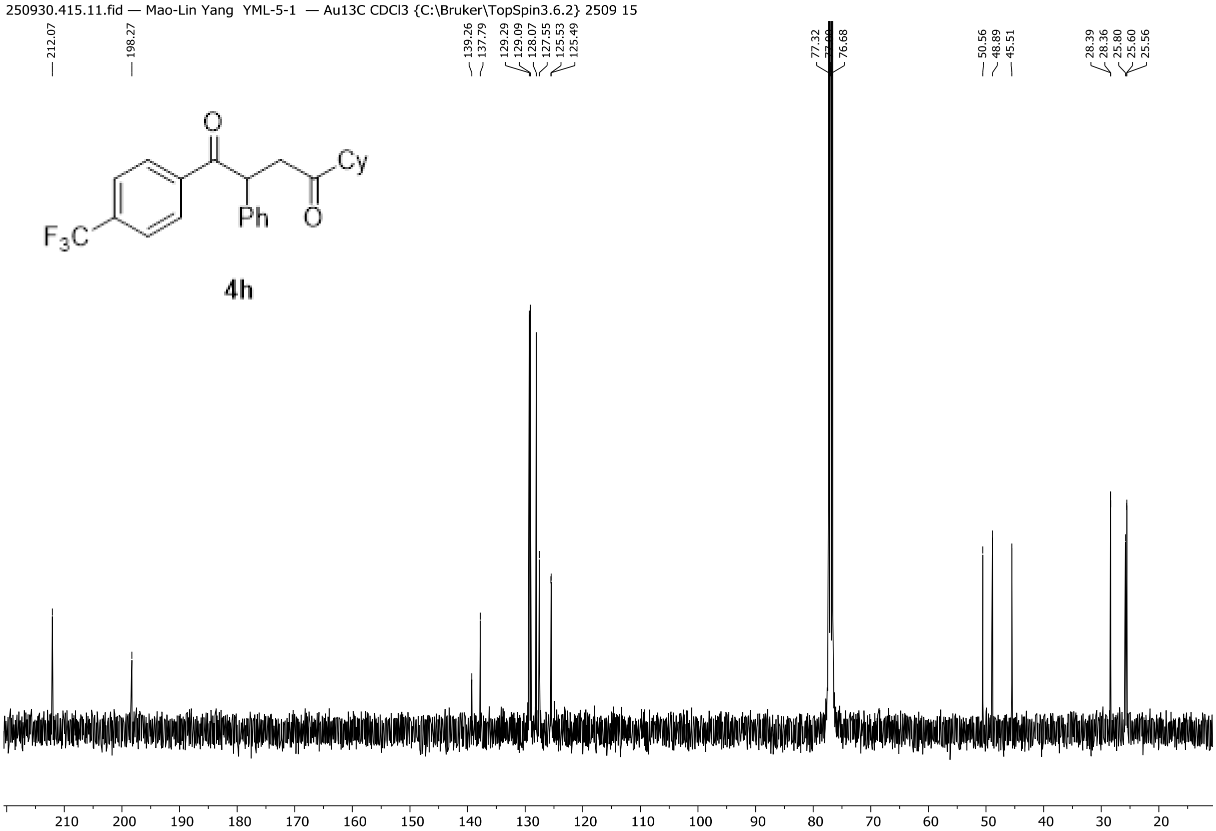

4h ^{19}F NMR (282 MHz, $CDCl_3$)

250930.415.12.fid — Mao-Lin Yang YML-5-1 — Au19F CDCl3 {C:\Bruker\TopSpin3.6.2} 2509 15

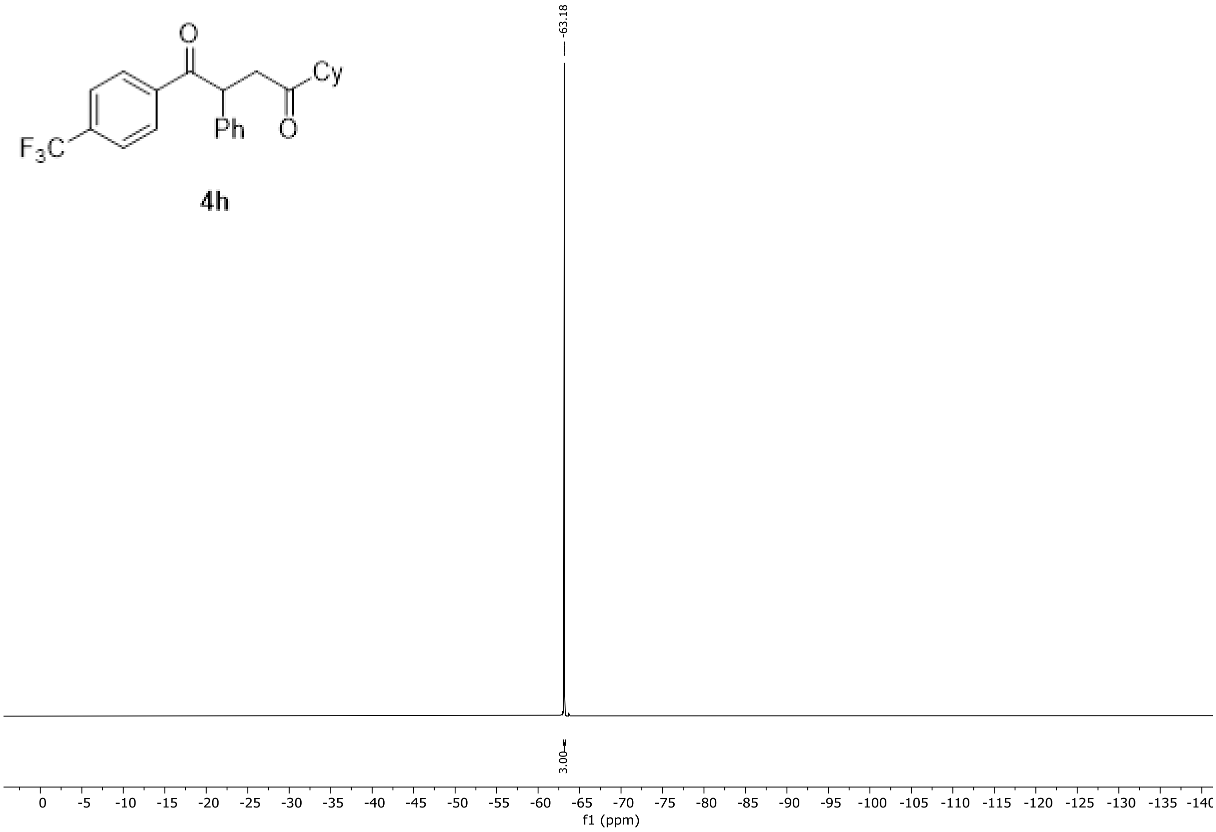

4i ^{1}H NMR (400 MHz, $CDCl_3$)

251020.404.10.fid — Mao-Lin Yang YML-5-1011-15 — Au1H CDCl3 {C:\Bruker\TopSpin3.6.2} 2510 4

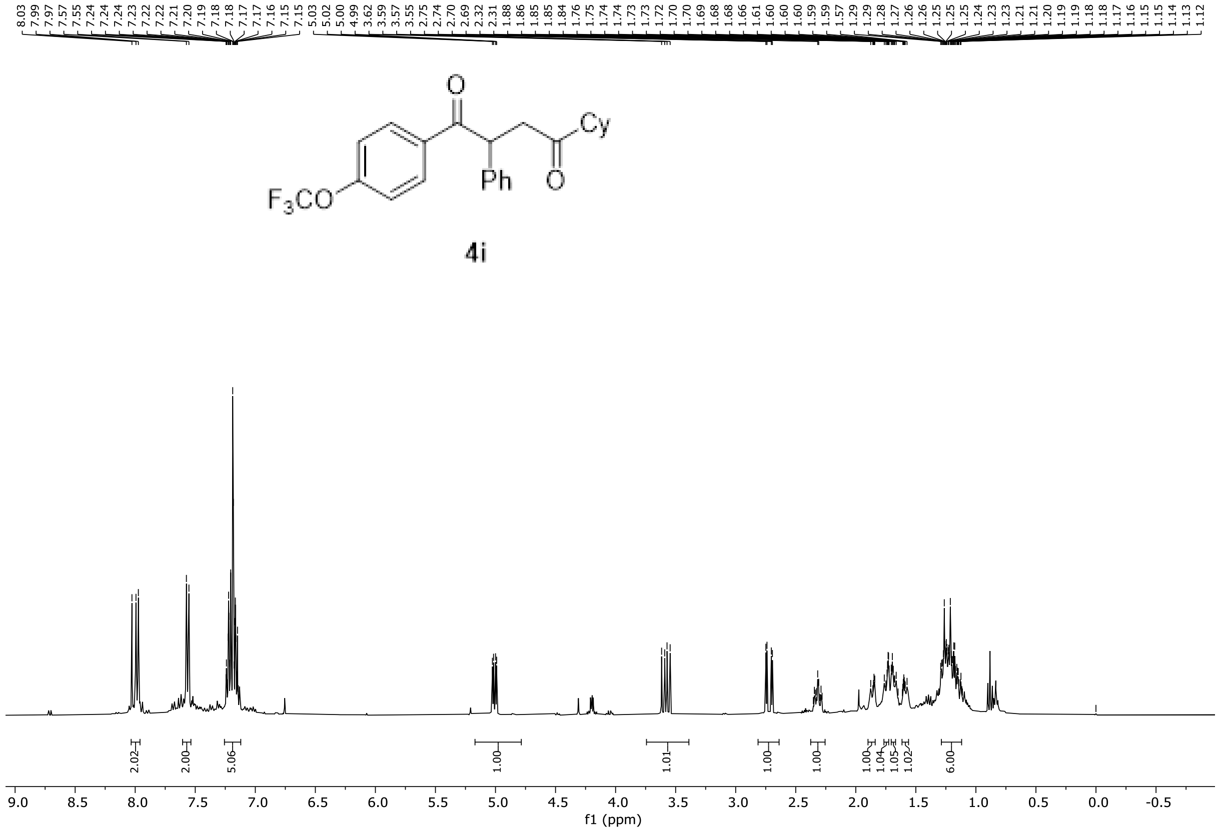

4i ^{13}C NMR (101 MHz, $CDCl_3$)

251020.404.11.fid — Mao-Lin Yang YML-5-1011-15 — Au13C CDCl3 {C:\Bruker\TopSpin3.6.2} 2510 4

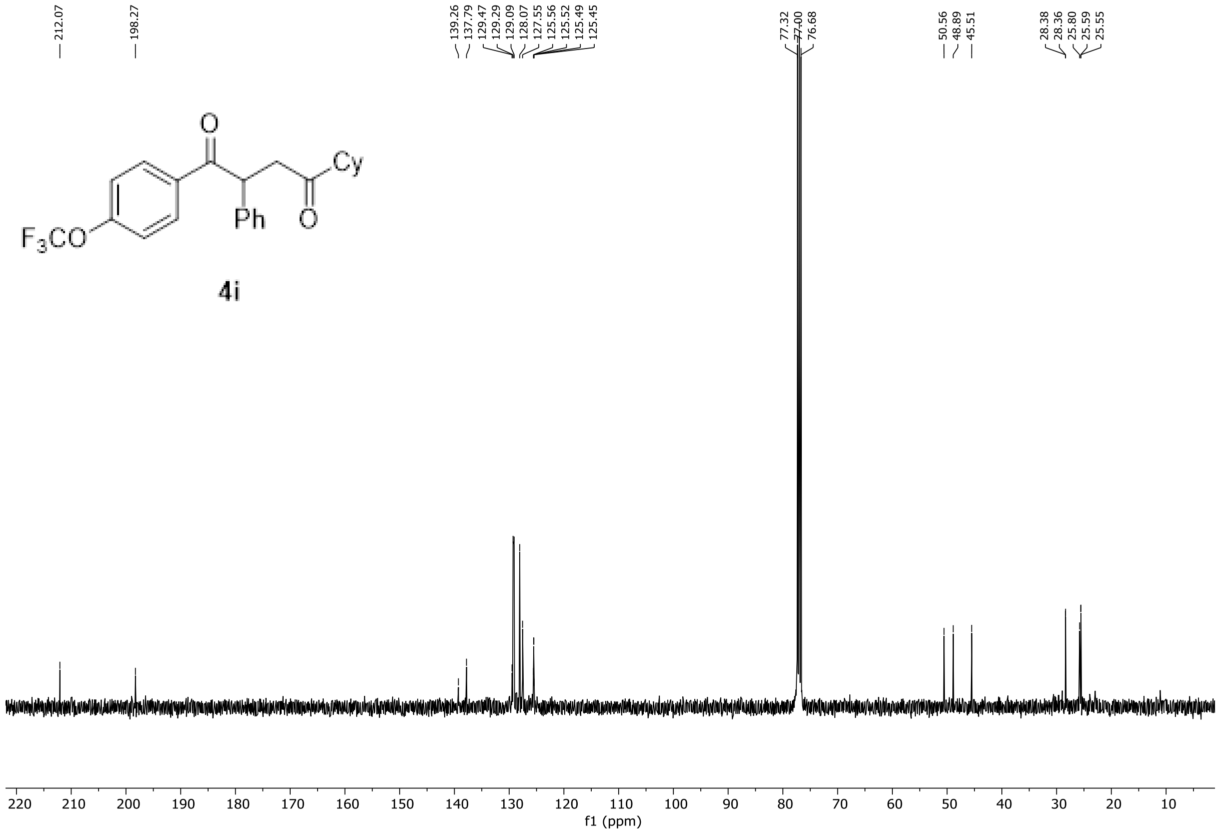

4i ^{19}F NMR (282 MHz, $CDCl_3$)

251021.422.10.fid — Lin-Mao Yang YML-5-1011-15 — Au19F CDCl3 {C:\Bruker\TopSpin3.6.2} 2510 22

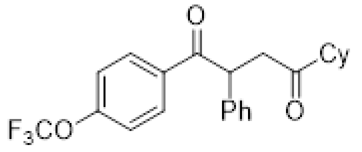

4i

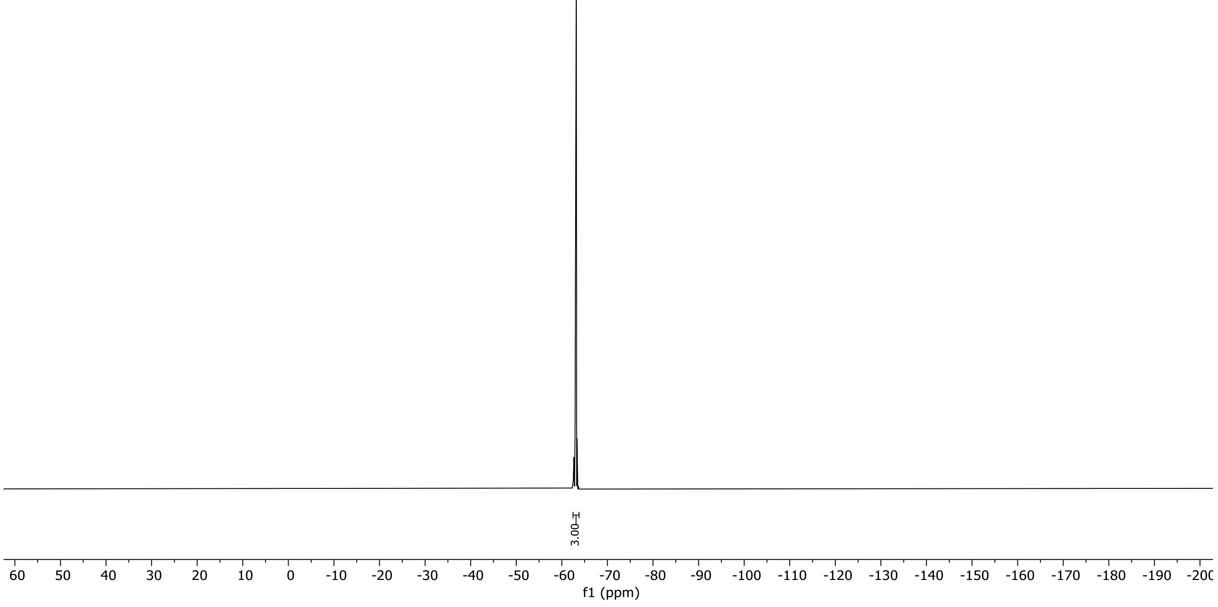

4j ^{1}H NMR (300 MHz, $CDCl_3$)

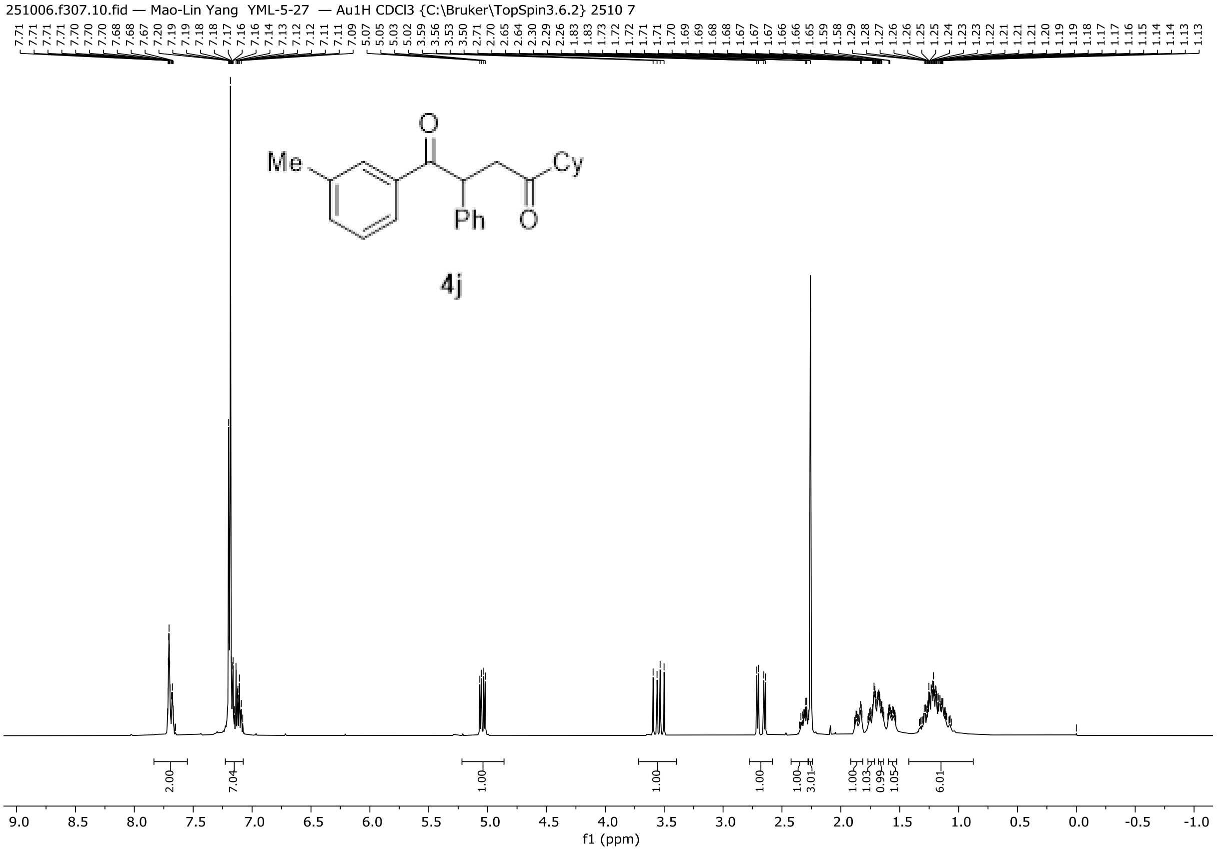

4j ^{13}C NMR (75 MHz, $CDCl_3$)

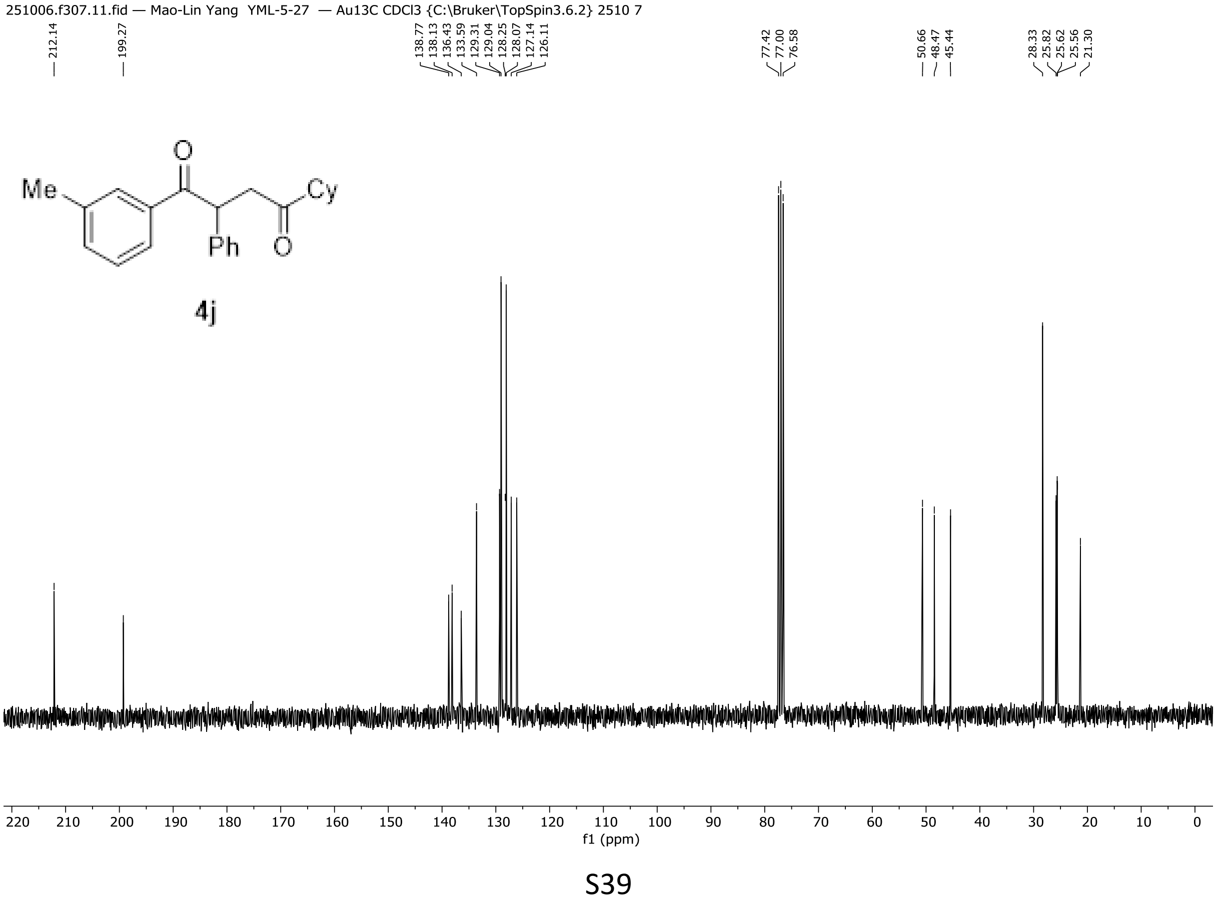

4k ^{1}H NMR (300 MHz, $CDCl_3$)

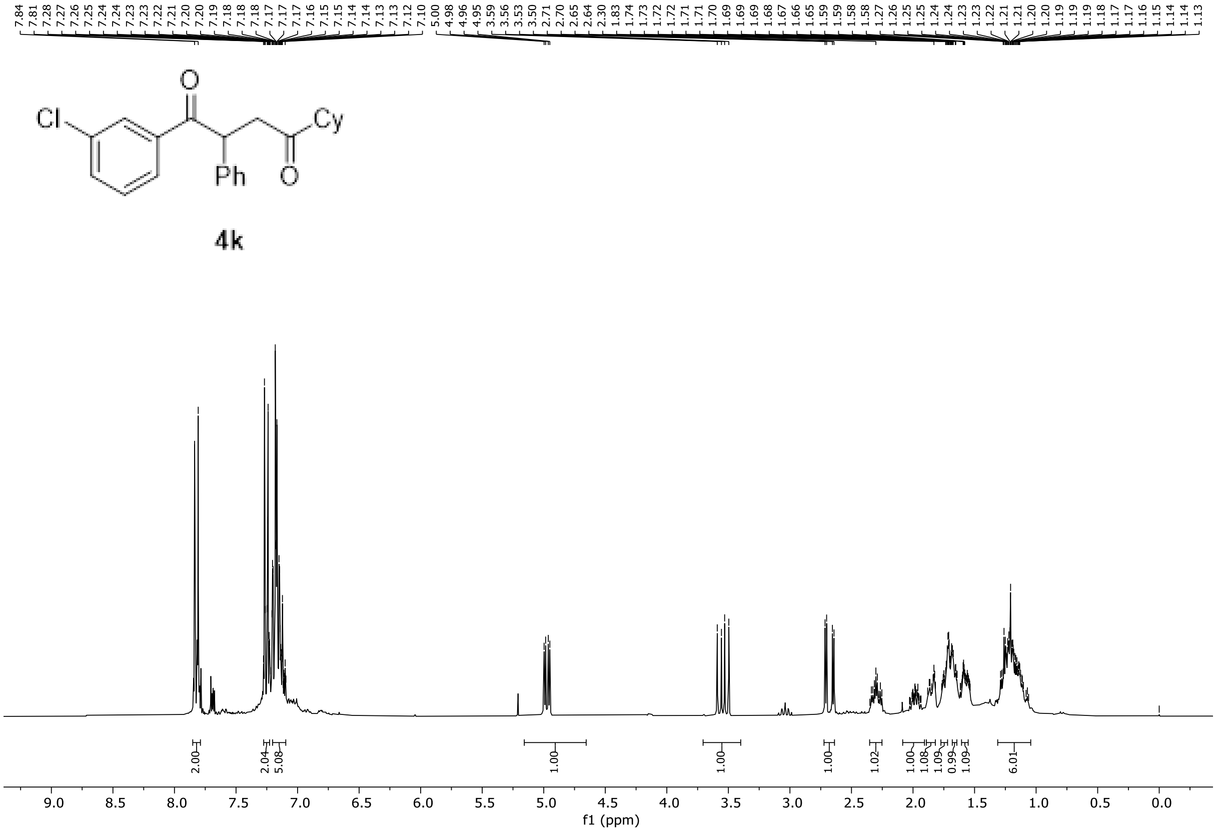

4k ^{13}C NMR (75 MHz, $CDCl_3$)

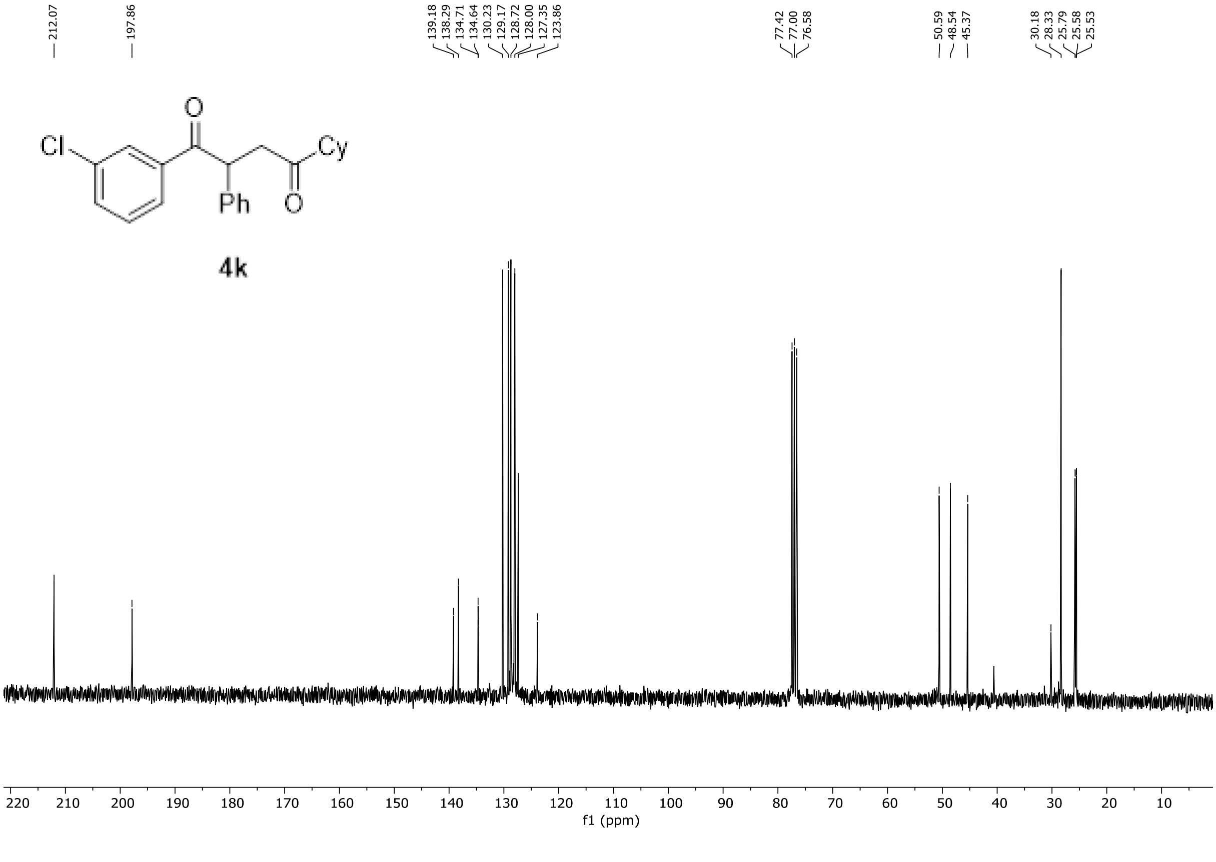

4l ^{1}H NMR (300 MHz, $CDCl_3$)

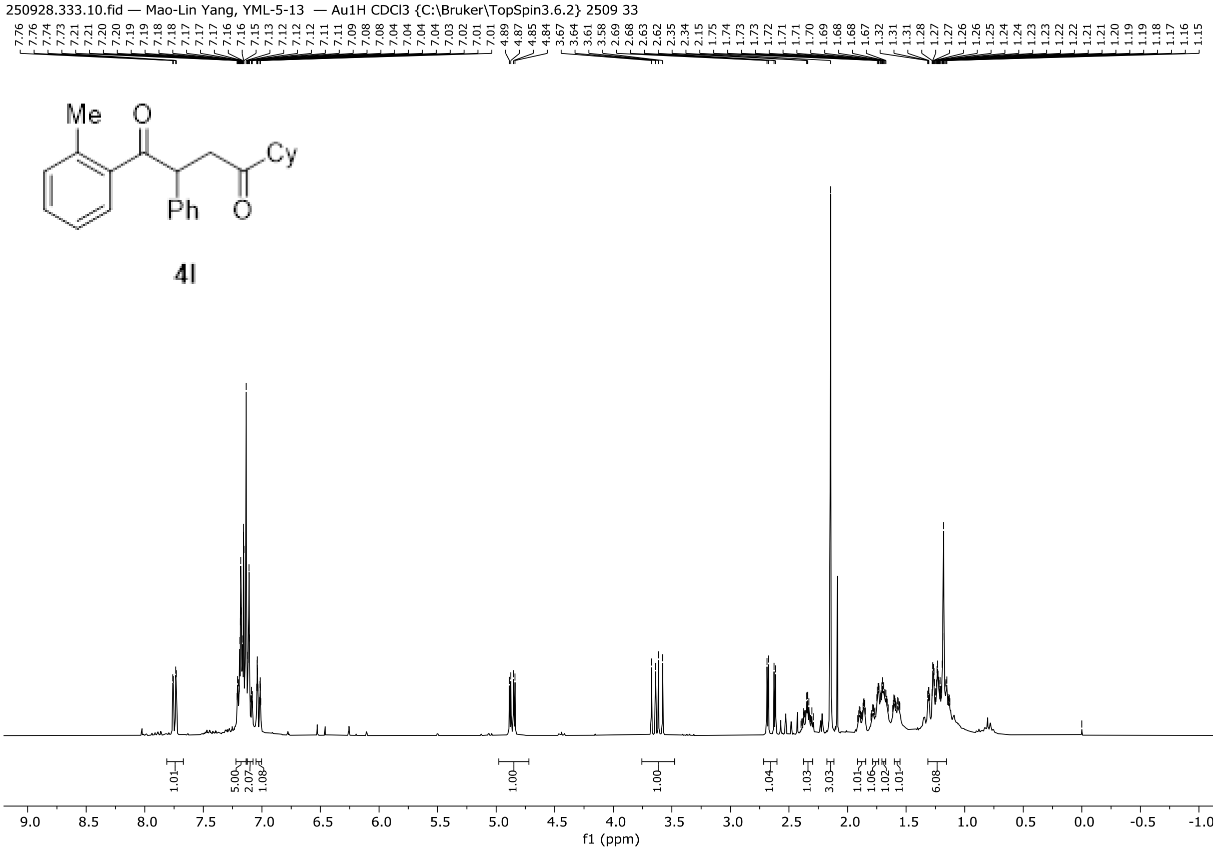

4l ^{13}C NMR (75 MHz, $CDCl_3$)

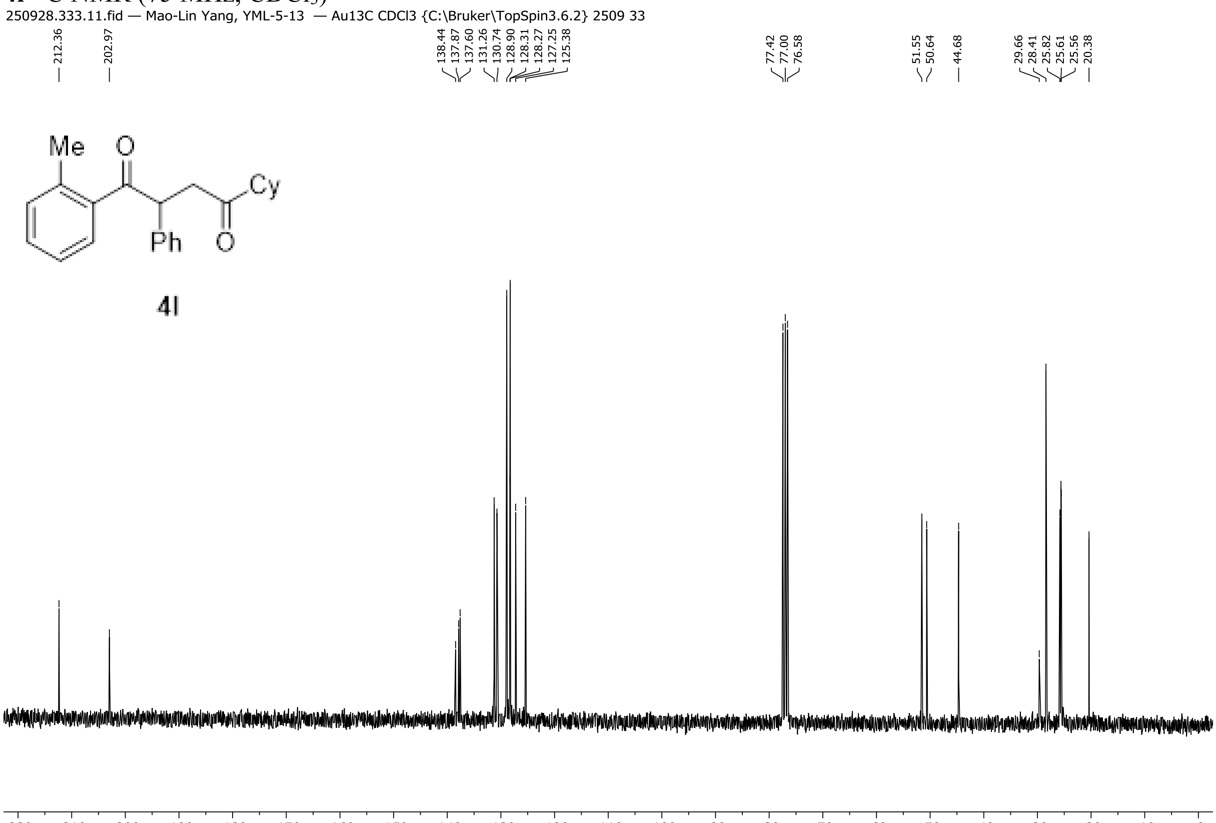

4m ^{1}H NMR (300 MHz, $CDCl_3$)

250928.337.10.fid — Mao-Lin Yang, YML-5-17 — Au1H CDCl3 {C:\Bruker\TopSpin3.6.2} 2509 37

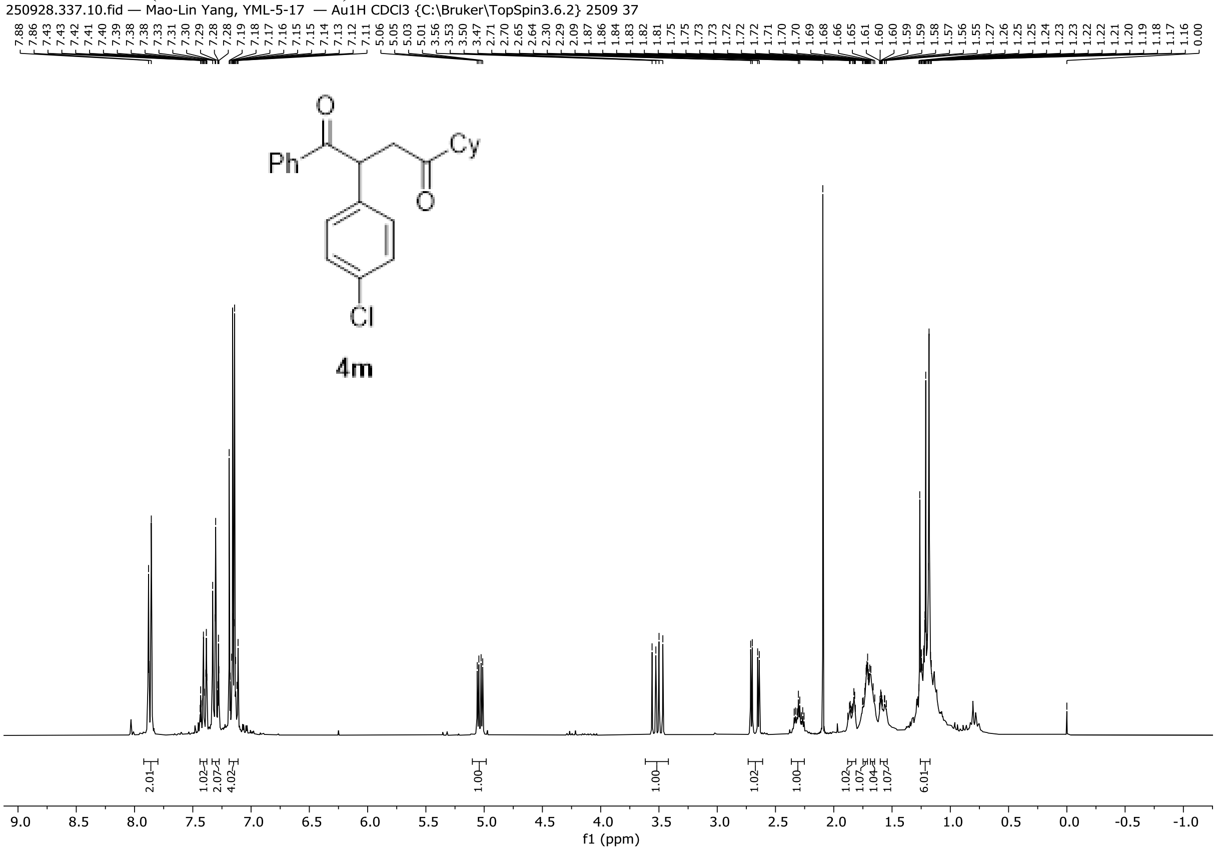

4m ^{13}C NMR (75 MHz, $CDCl_3$)

250928.337.11.fid — Mao-Lin Yang, YML-5-17 — Au13C CDCl3 {C:\Bruker\TopSpin3.6.2} 2509 37

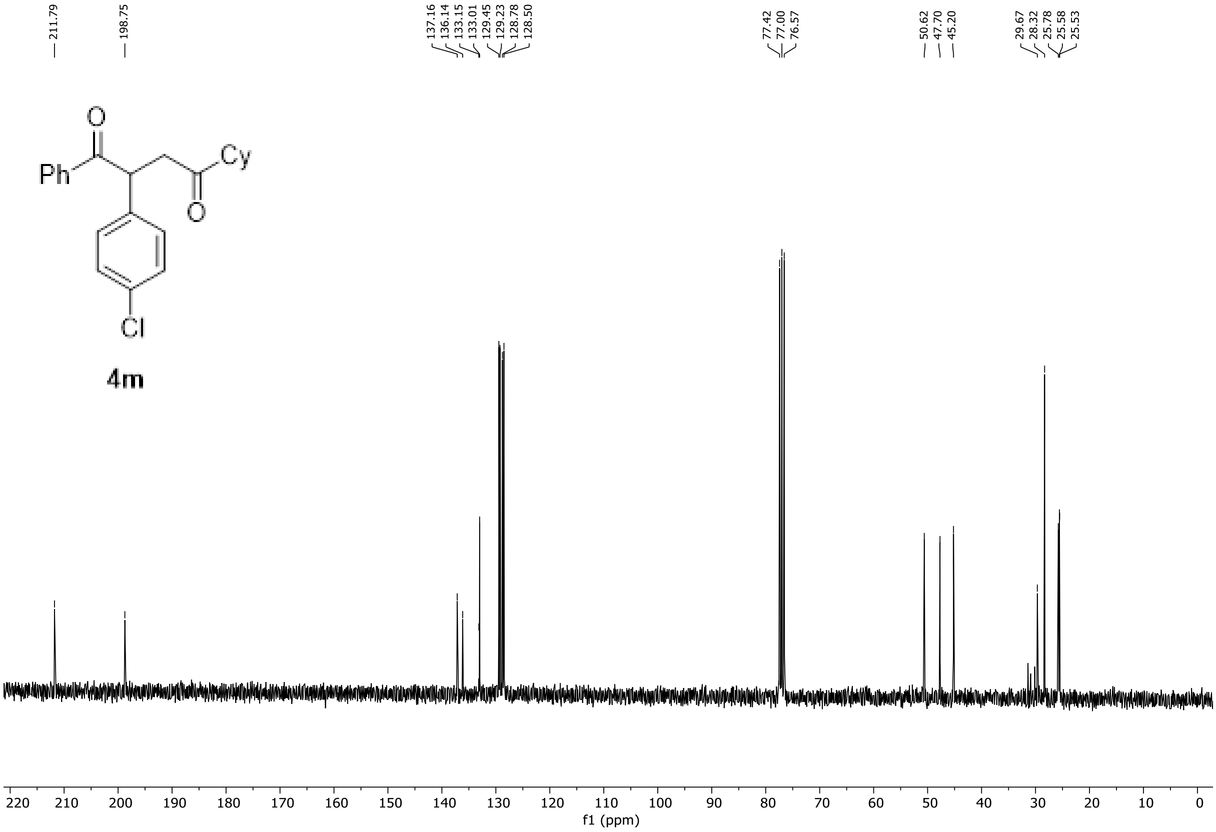

4n ^{1}H NMR (400 MHz, $CDCl_3$)

251021.418.10.fid — Lin-Mao Yang YML-5-1011-17 — Au1H CDCl3 {C:\Bruker\TopSpin3.6.2} 2510 18

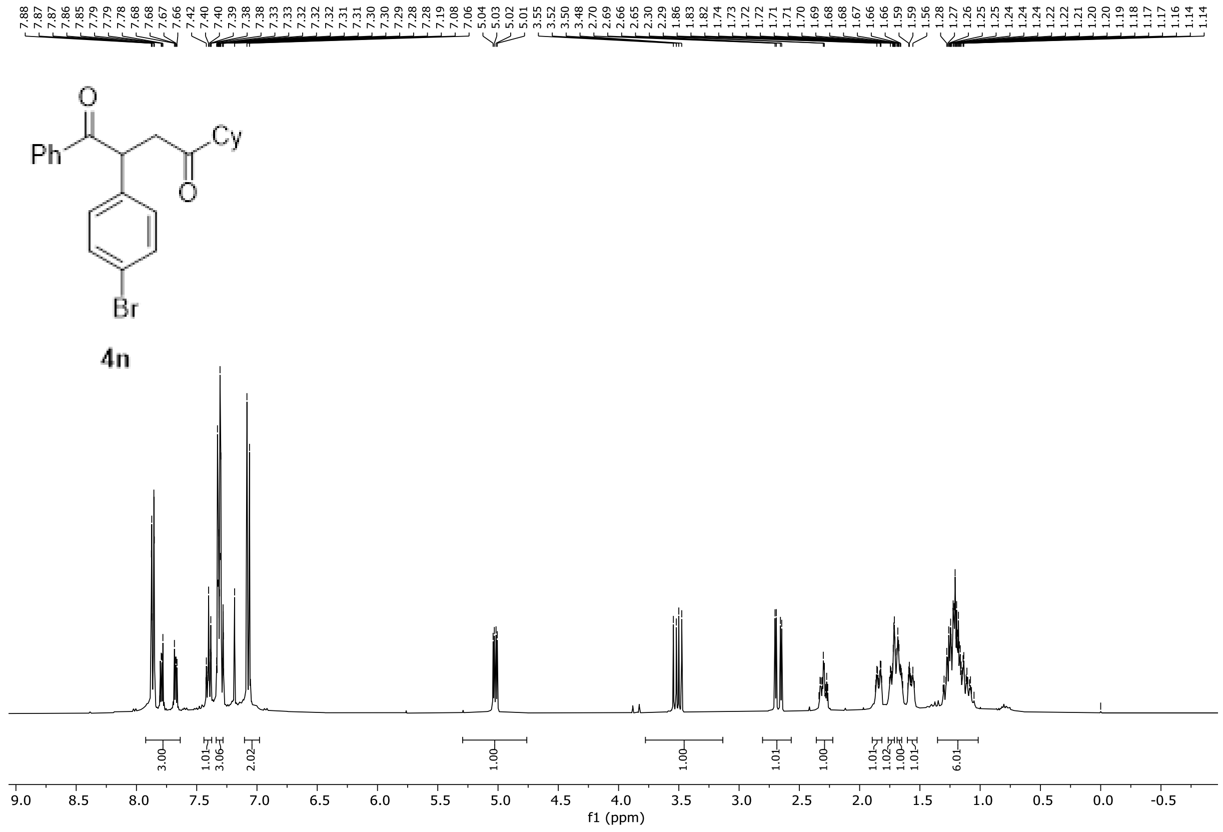

4n ^{13}C NMR (101 MHz, $CDCl_3$)

251021.418.11.fid — Lin-Mao Yang YML-5-1011-17 — Au13C CDCl3 {C:\Bruker\TopSpin3.6.2} 2510 18

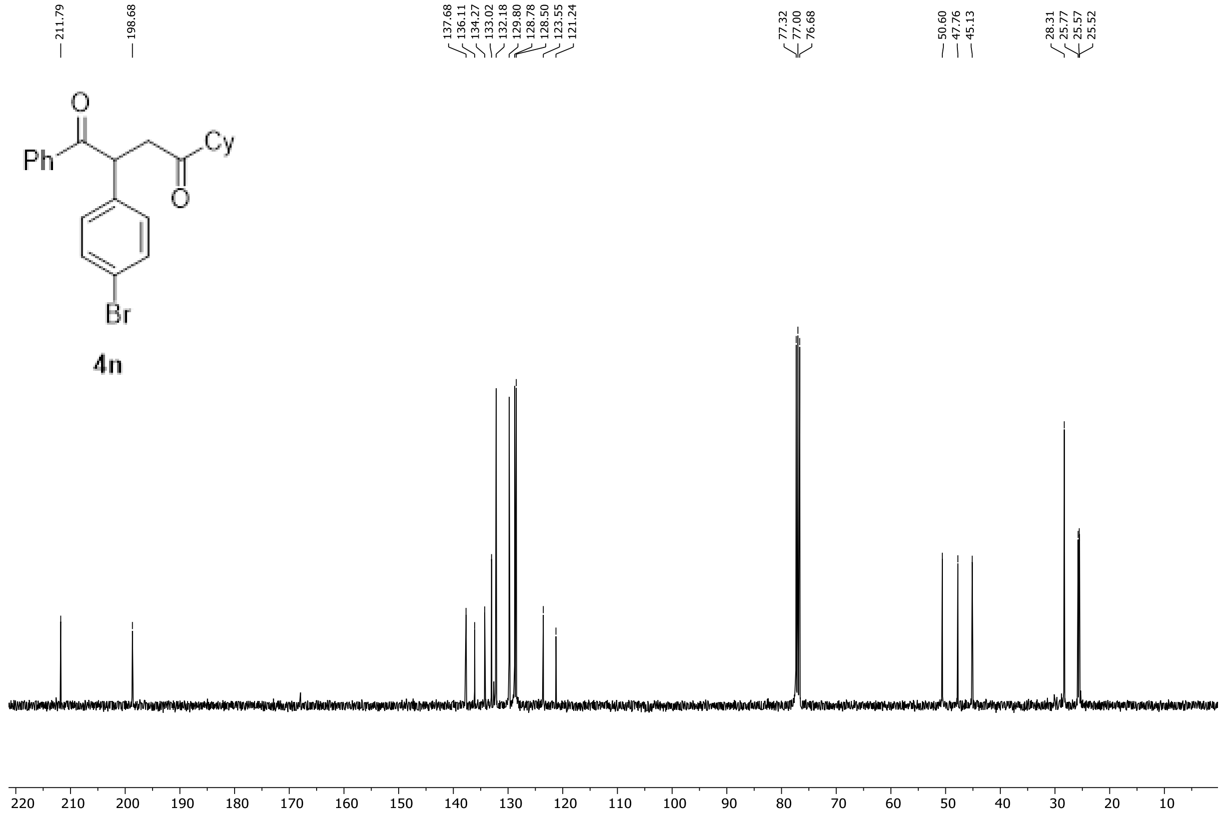

4o ^{1}H NMR (300 MHz, $CDCl_3$)

250928.338.10.fid — Mao-Lin Yang, YML-5-18 — Au1H CDCl3 {C:\Bruker\TopSpin3.6.2} 2509 38

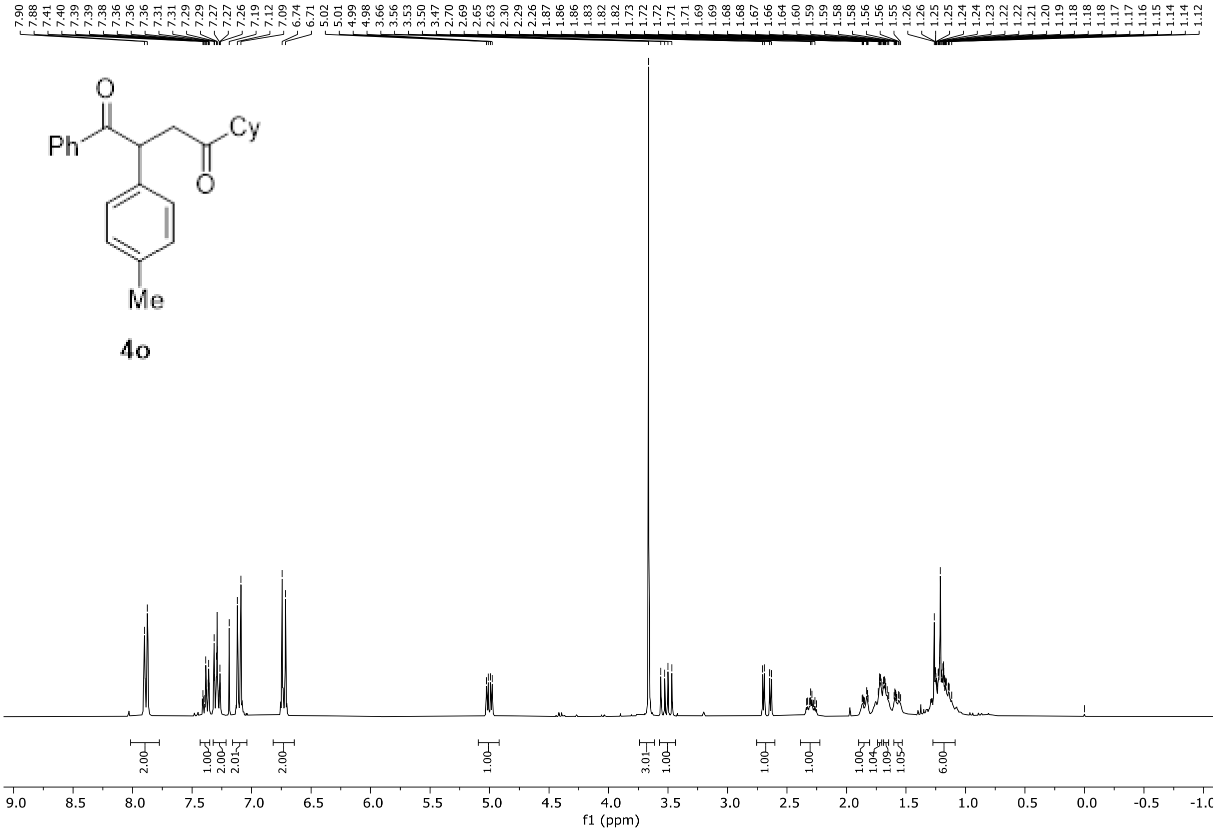

4o ^{13}C NMR (75 MHz, $CDCl_3$)

250928.338.11.fid — Mao-Lin Yang, YML-5-18 — Au13C CDCl3 {C:\Bruker\TopSpin3.6.2} 2509 38

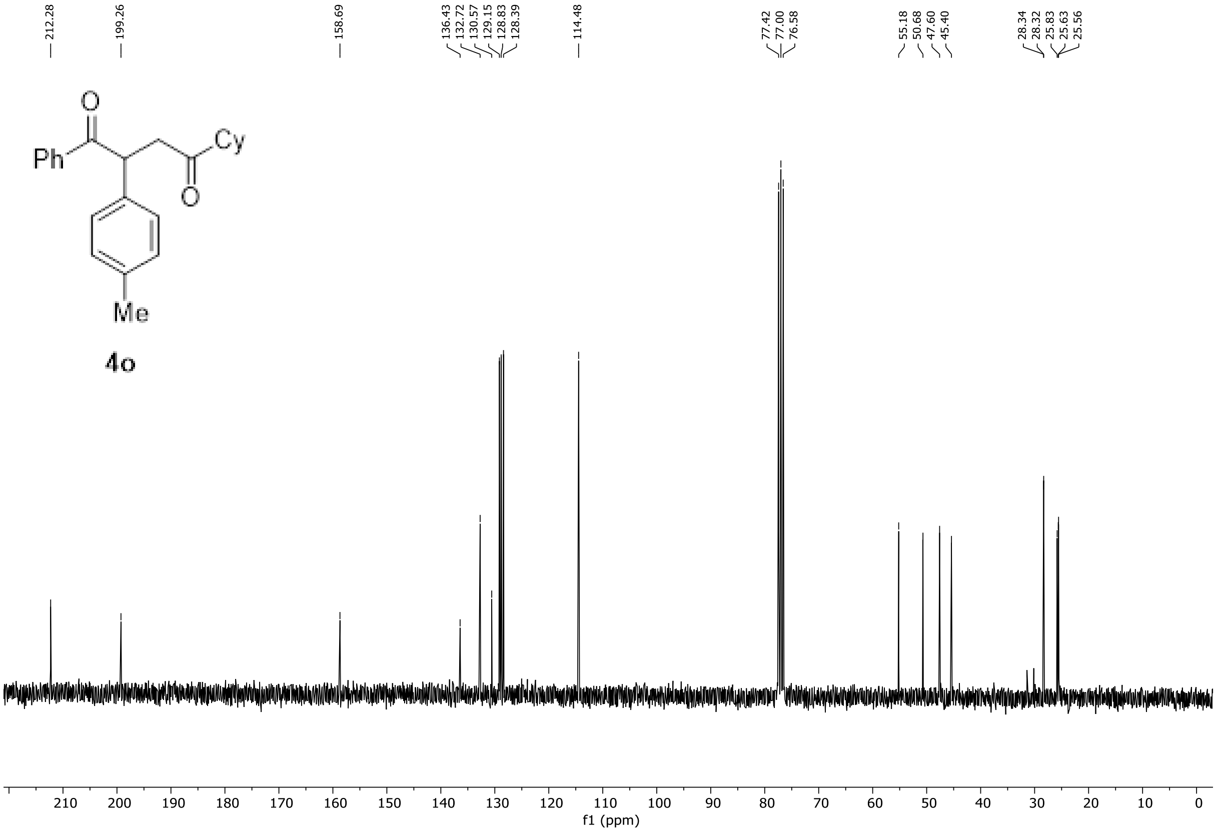

4p ^{1}H NMR (400 MHz, $CDCl_3$)

251020.403.10.fid — Mao-Lin Yang YML-5-1009-4 — Au1H CDCl3 {C:\Bruker\TopSpin3.6.2} 2510 3

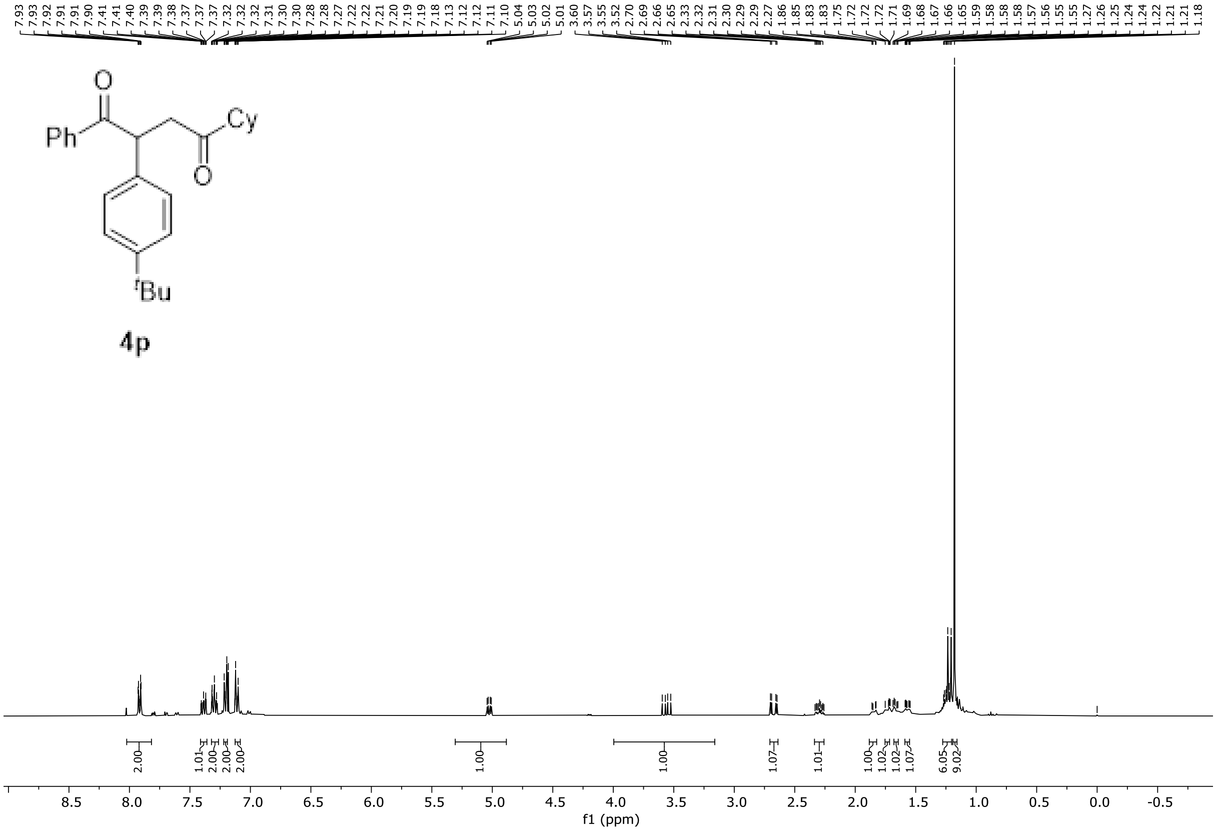

4p ^{13}C NMR (101 MHz, $CDCl_3$)

251020.403.11.fid — Mao-Lin Yang YML-5-1009-4 — Au13C CDCl3 {C:\Bruker\TopSpin3.6.2} 2510 3

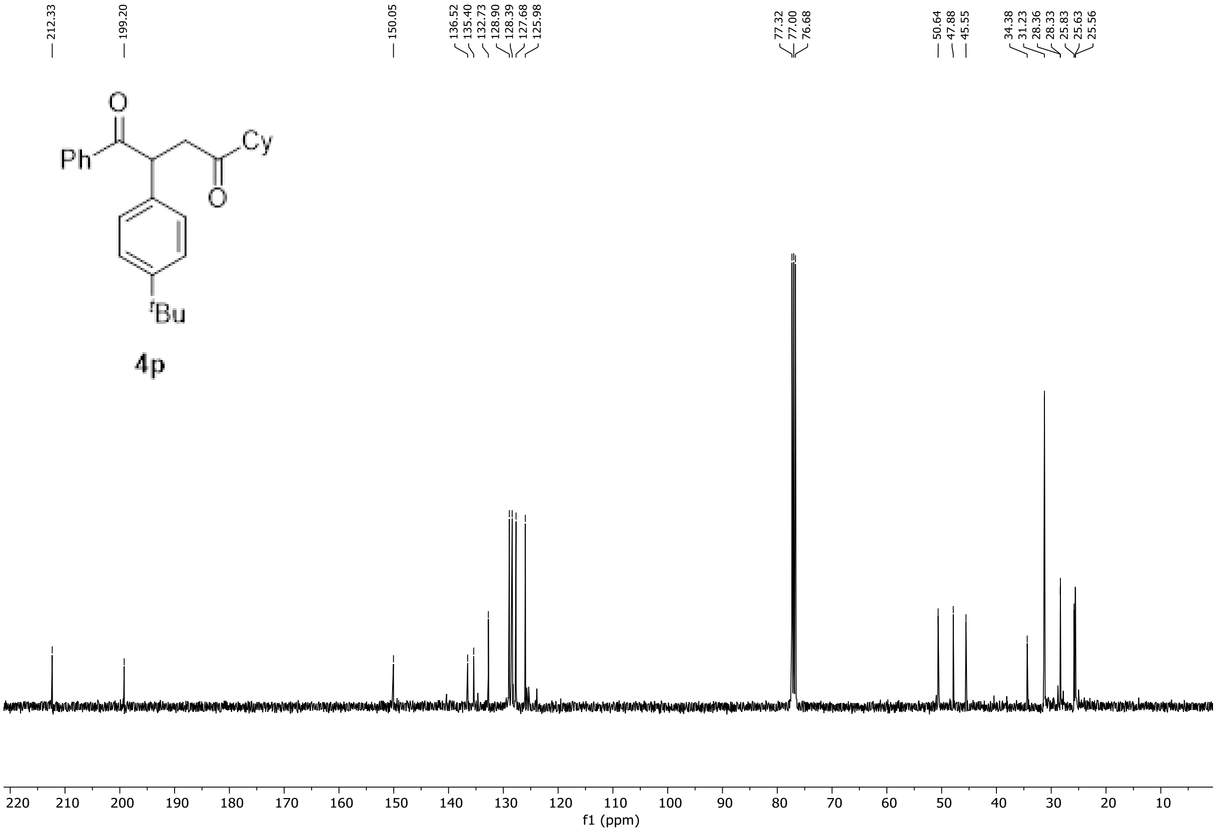

4q ^{1}H NMR (300 MHz, $CDCl_3$)

251117.304.10.fid — Mao-Lin Yang YML-5-1110-1 — Au1H CDCl3 {C:\Bruker\TopSpin3.6.2} 2511 4

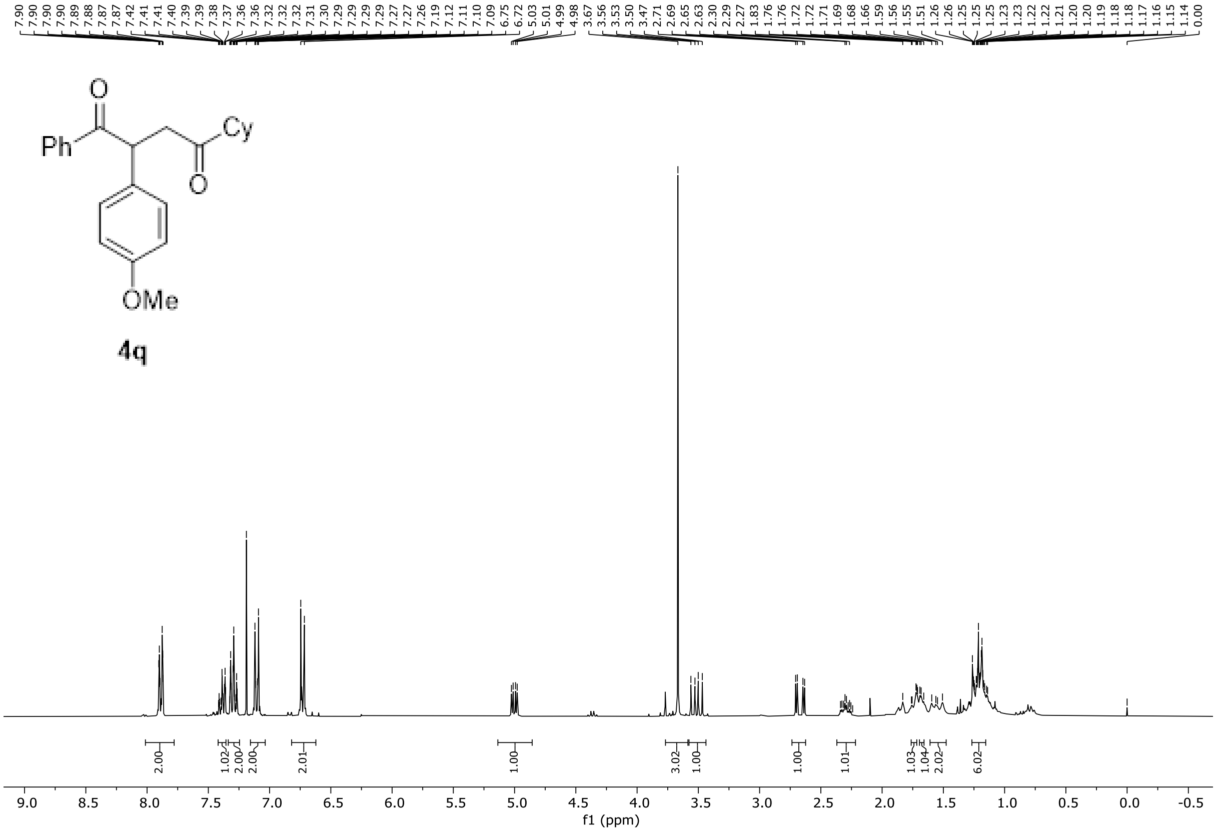

4q ^{13}C NMR (75 MHz, $CDCl_3$)

251117.304.11.fid — Mao-Lin Yang YML-5-1110-1 — Au13C CDCl3 {C:\Bruker\TopSpin3.6.2} 2511 4

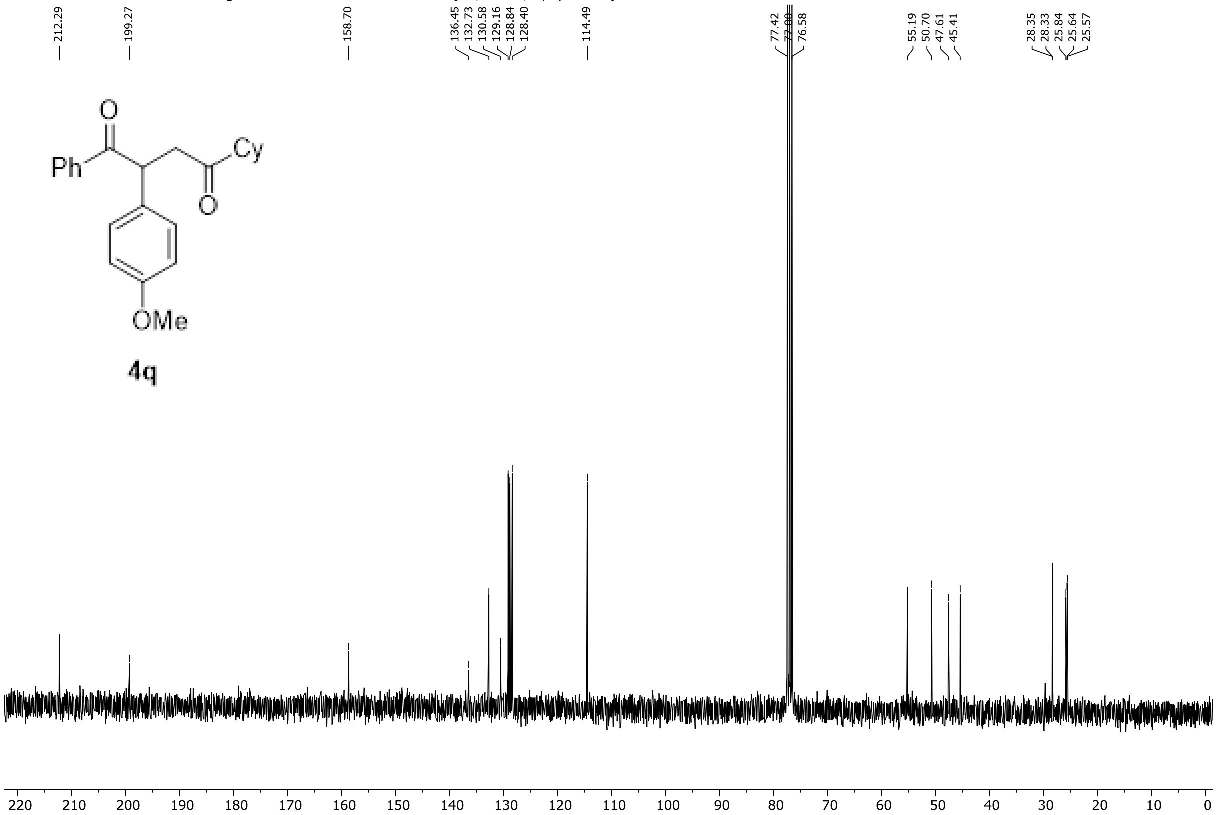

4r ^{1}H NMR (300 MHz, $CDCl_3$)

250928.339.10.fid — Mao-Lin Yang, YML-5-19 — Au1H CDCl3 {C:\Bruker\TopSpin3.6.2} 2509 39

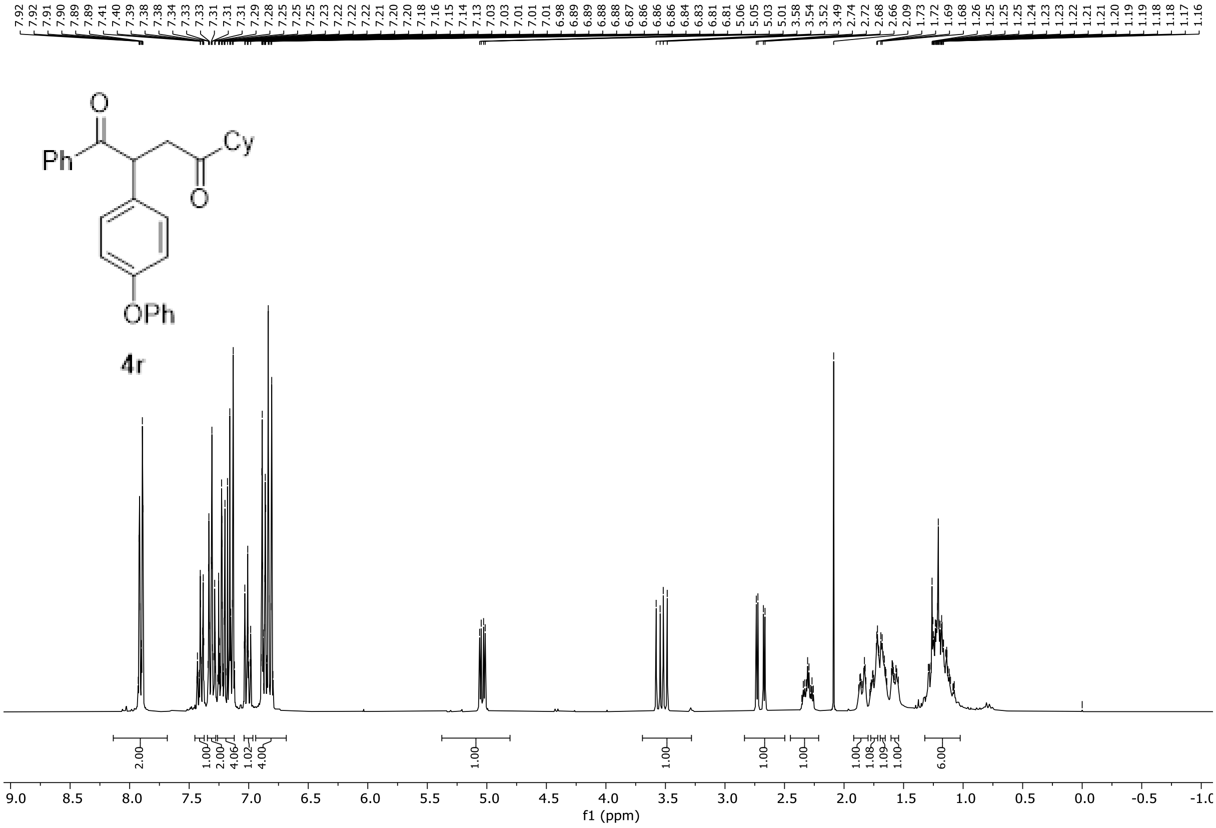

4r ^{13}C NMR (75 MHz, $CDCl_3$)

250928.339.11.fid — Mao-Lin Yang, YML-5-19 — Au13C CDCl3 {C:\Bruker\TopSpin3.6.2} 2509 39

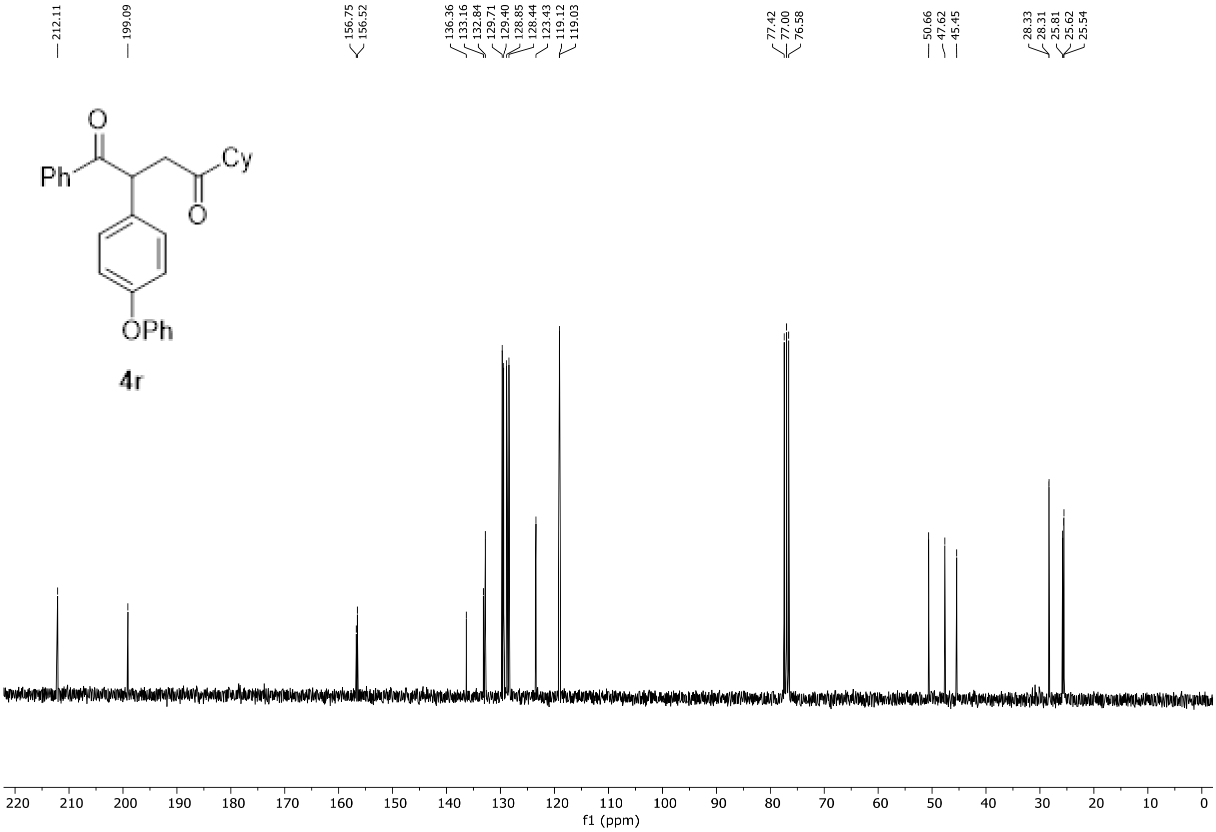

4s ^{1}H NMR (300 MHz, $CDCl_3$)

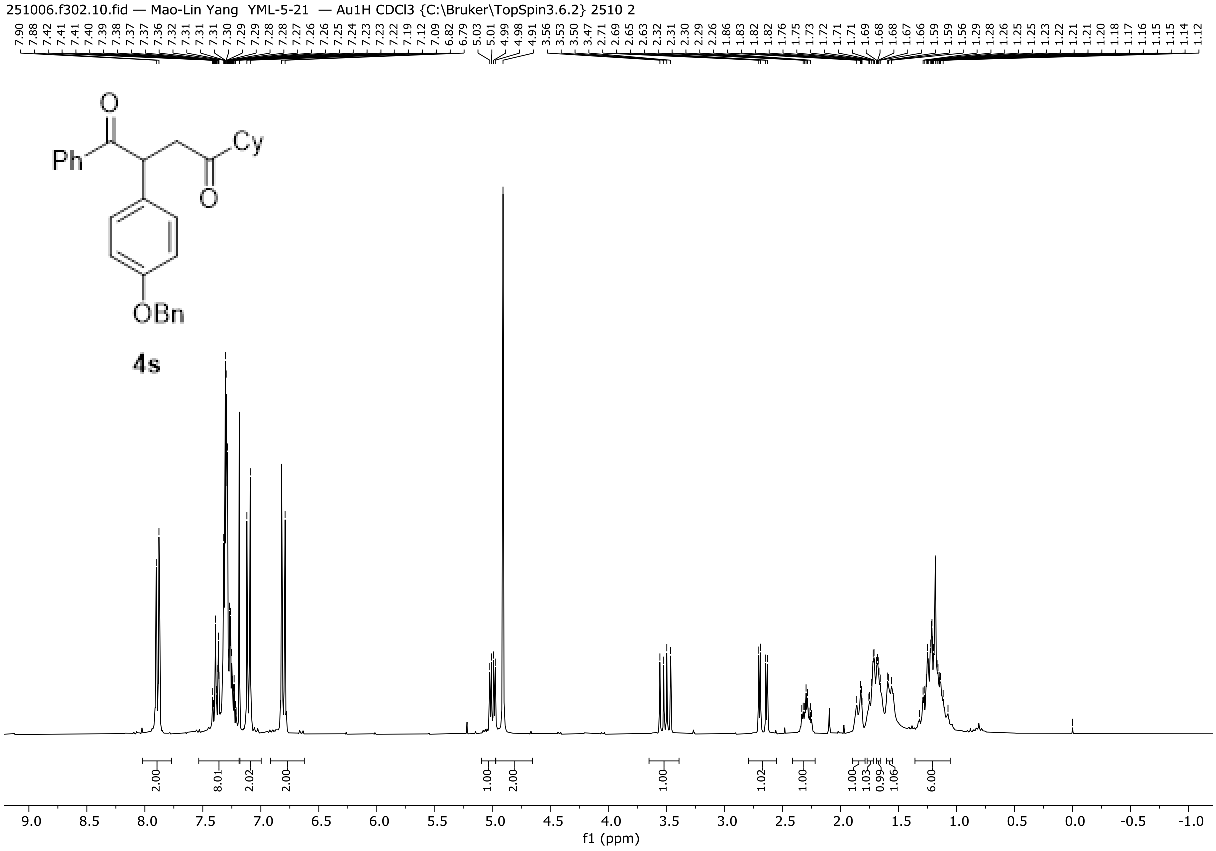

4s ^{13}C NMR (75 MHz, $CDCl_3$)

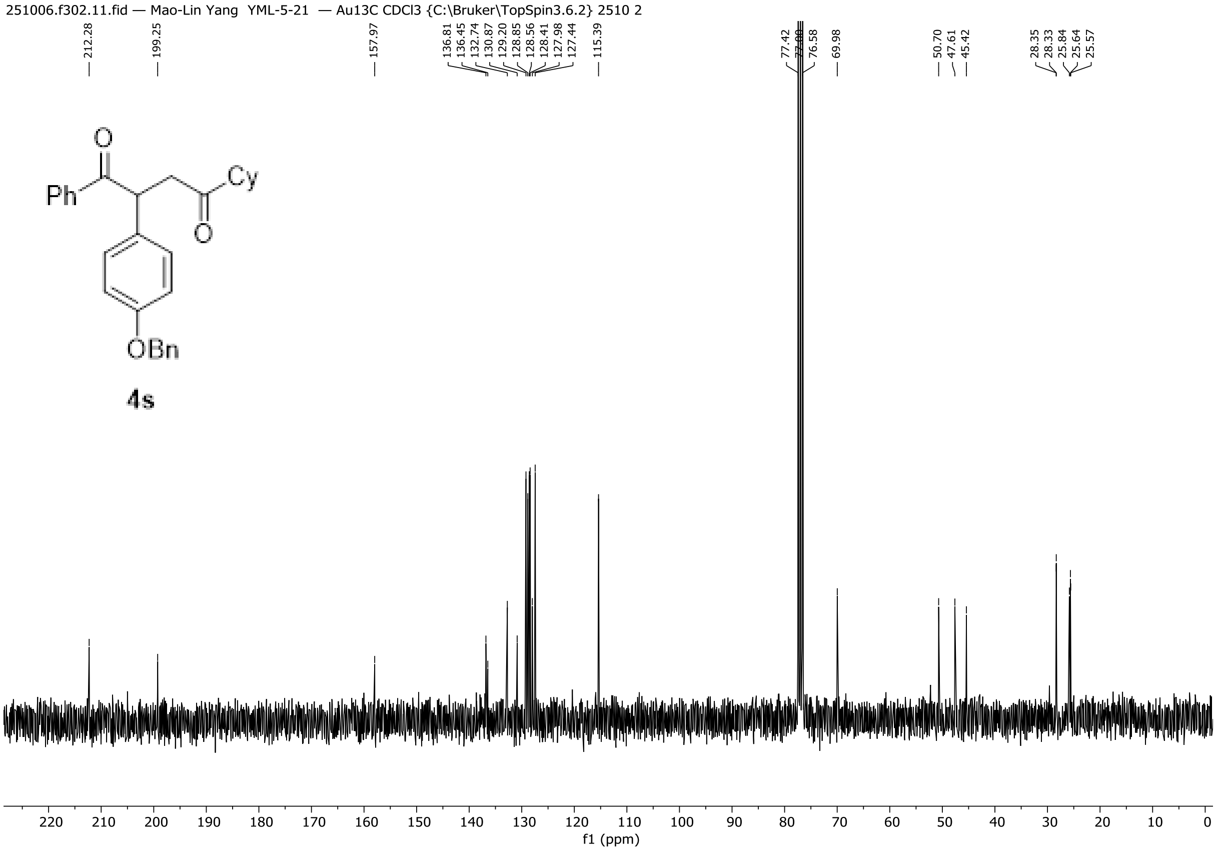

4t ^{1}H NMR (300 MHz, $CDCl_3$)

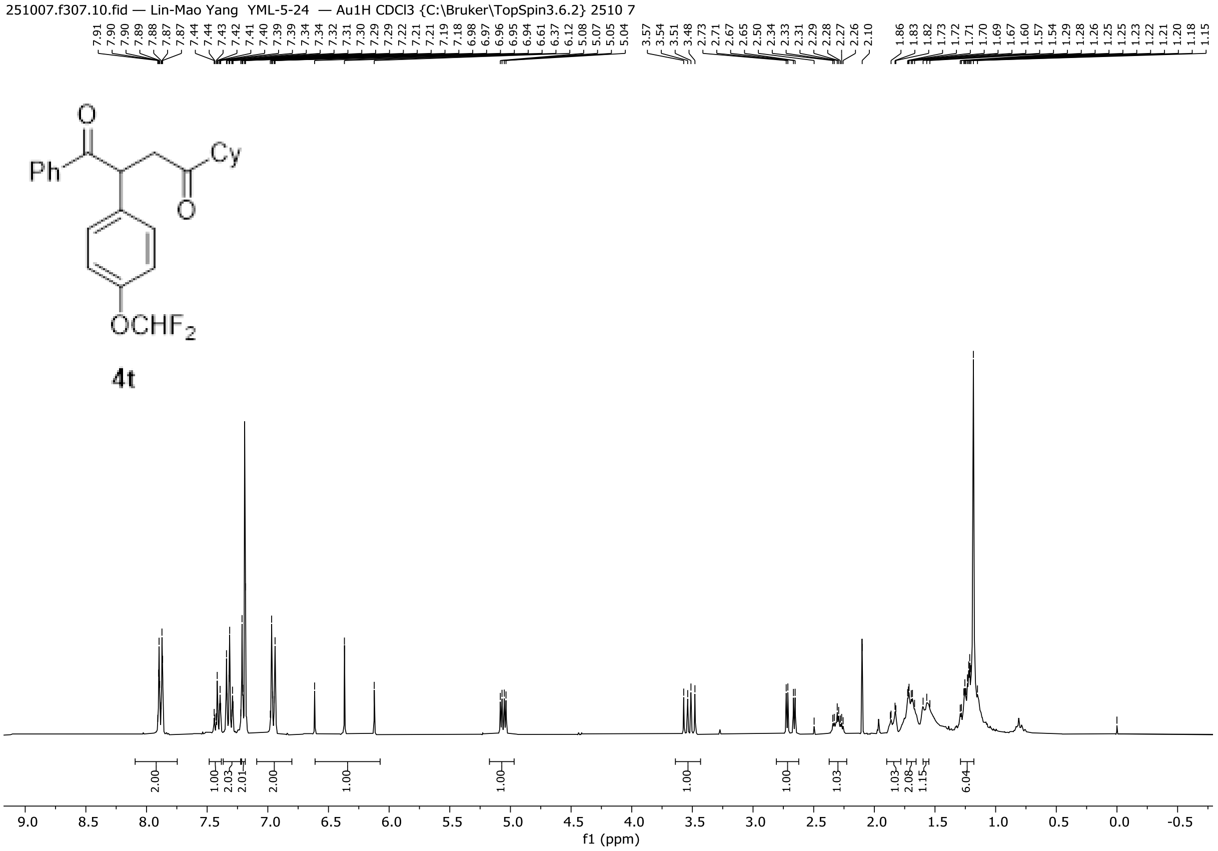

4t ^{13}C NMR (75 MHz, $CDCl_3$)

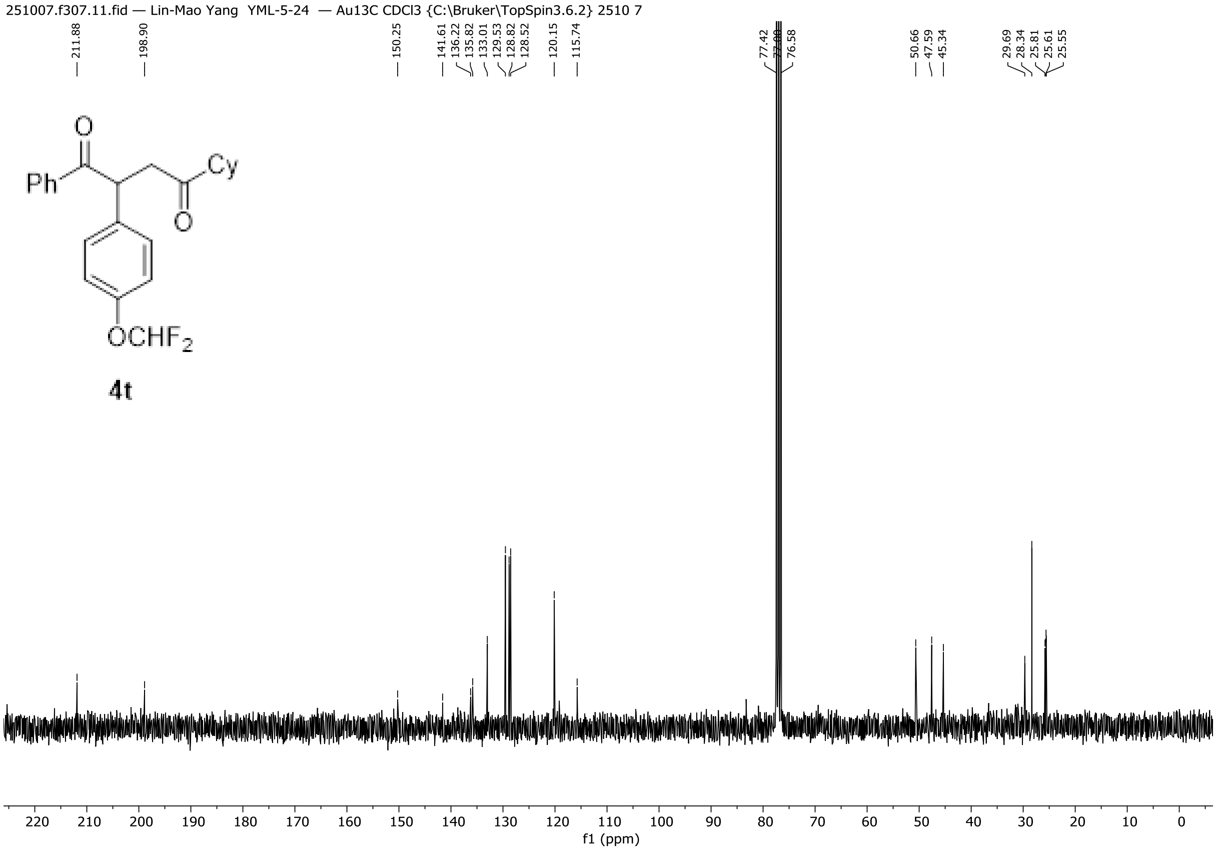

4t ^{19}F NMR (282 MHz, $CDCl_3$)

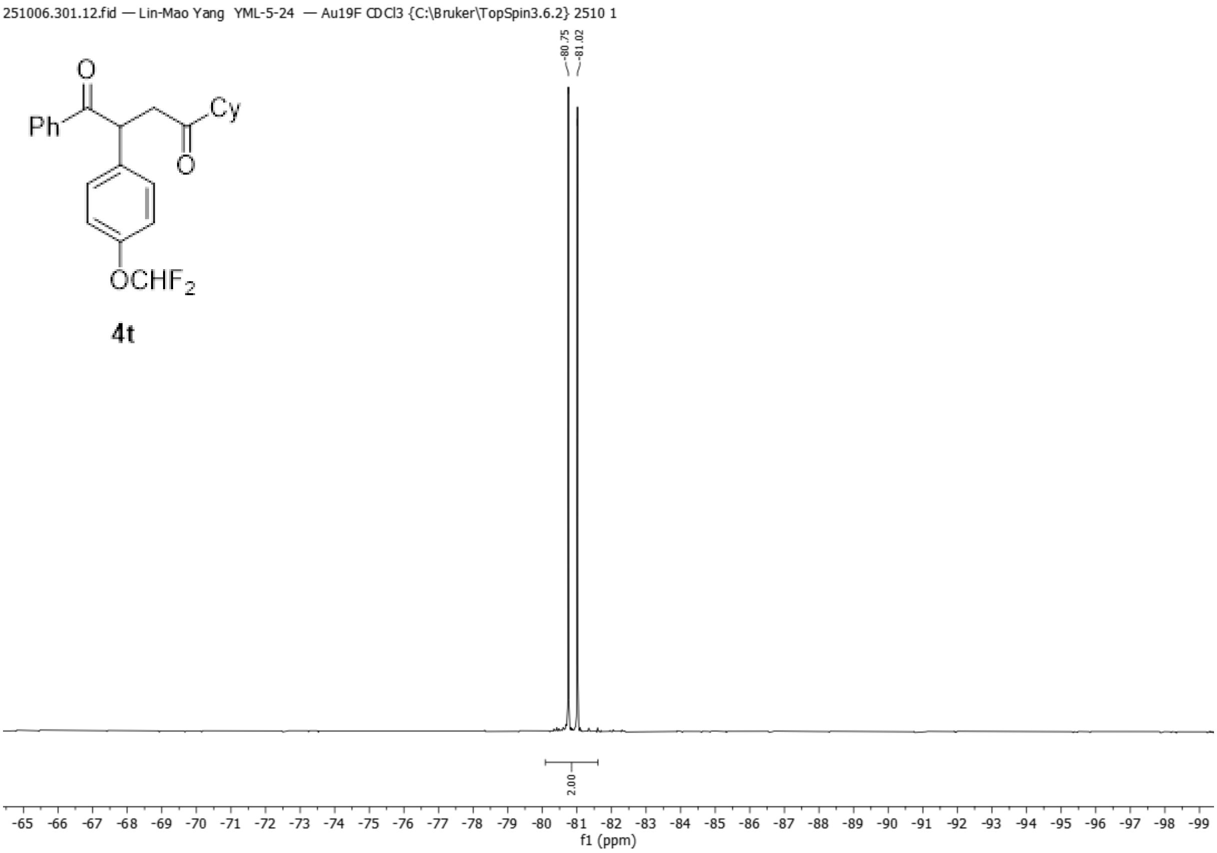

4u ^{1}H NMR (300 MHz, $CDCl_3$)

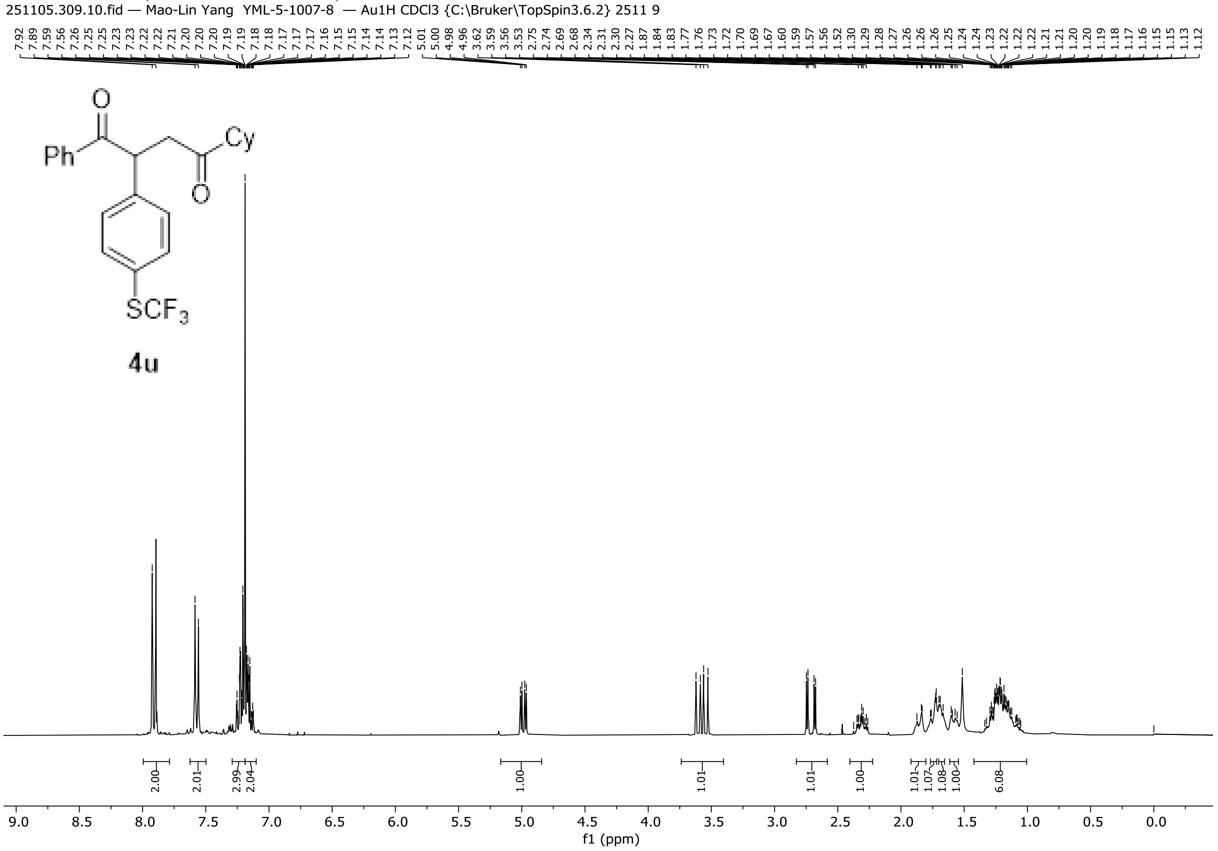

4u ^{13}C NMR (75 MHz, $CDCl_3$)

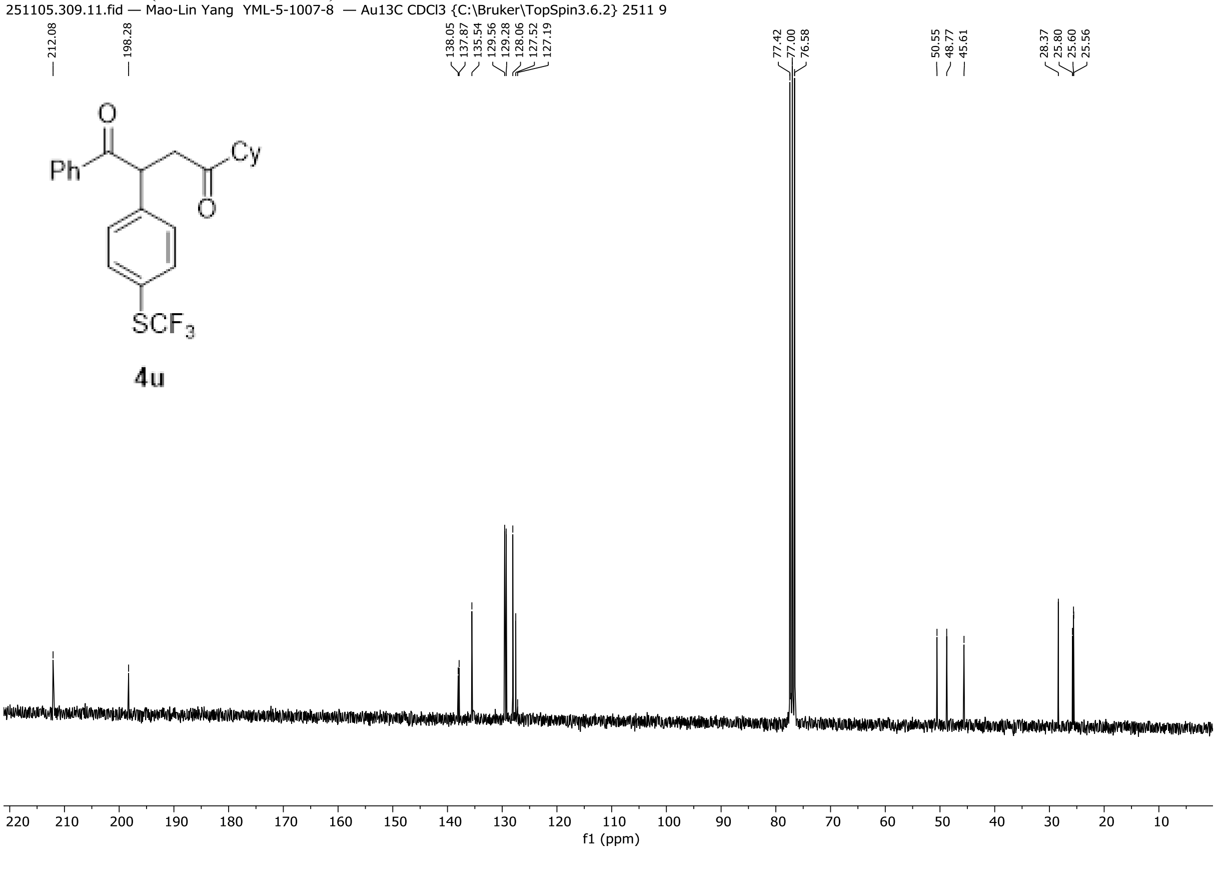

4u ^{19}F NMR (282 MHz, $CDCl_3$)

251105.309.12.fid — Mao-Lin Yang YML-5-1007-8 — Au19F CDCl3 {C:\Bruker\TopSpin3.6.2} 2511 9

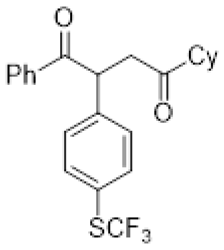

4u

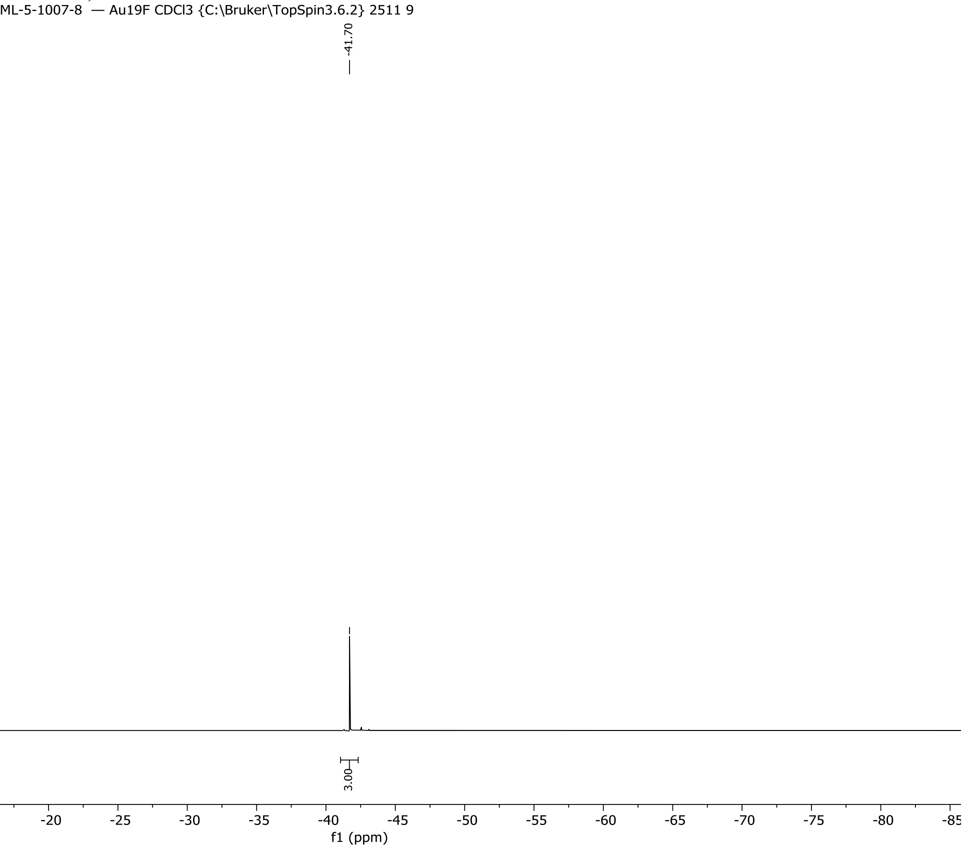

4v ^{1}H NMR (300 MHz, $CDCl_3$)

251023.f310.10.fid — Lin-Mao Yang YML-5-0930-3 — Au1H CDCl3 {C:\Bruker\TopSpin3.6.2} 2510 10

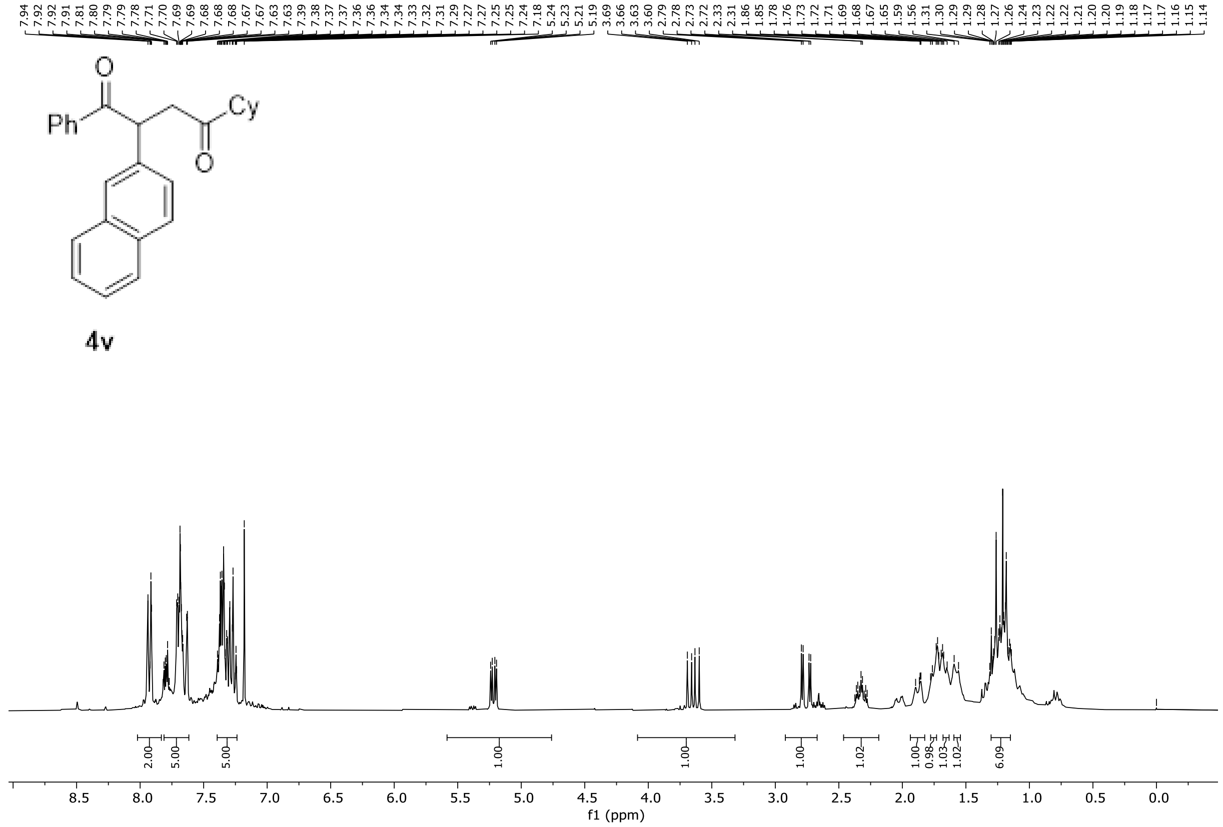

4v ^{13}C NMR (75 MHz, $CDCl_3$)

251023.f310.11.fid — Lin-Mao Yang YML-5-0930-3 — Au13C CDCl3 {C:\Bruker\TopSpin3.6.2} 2510 10

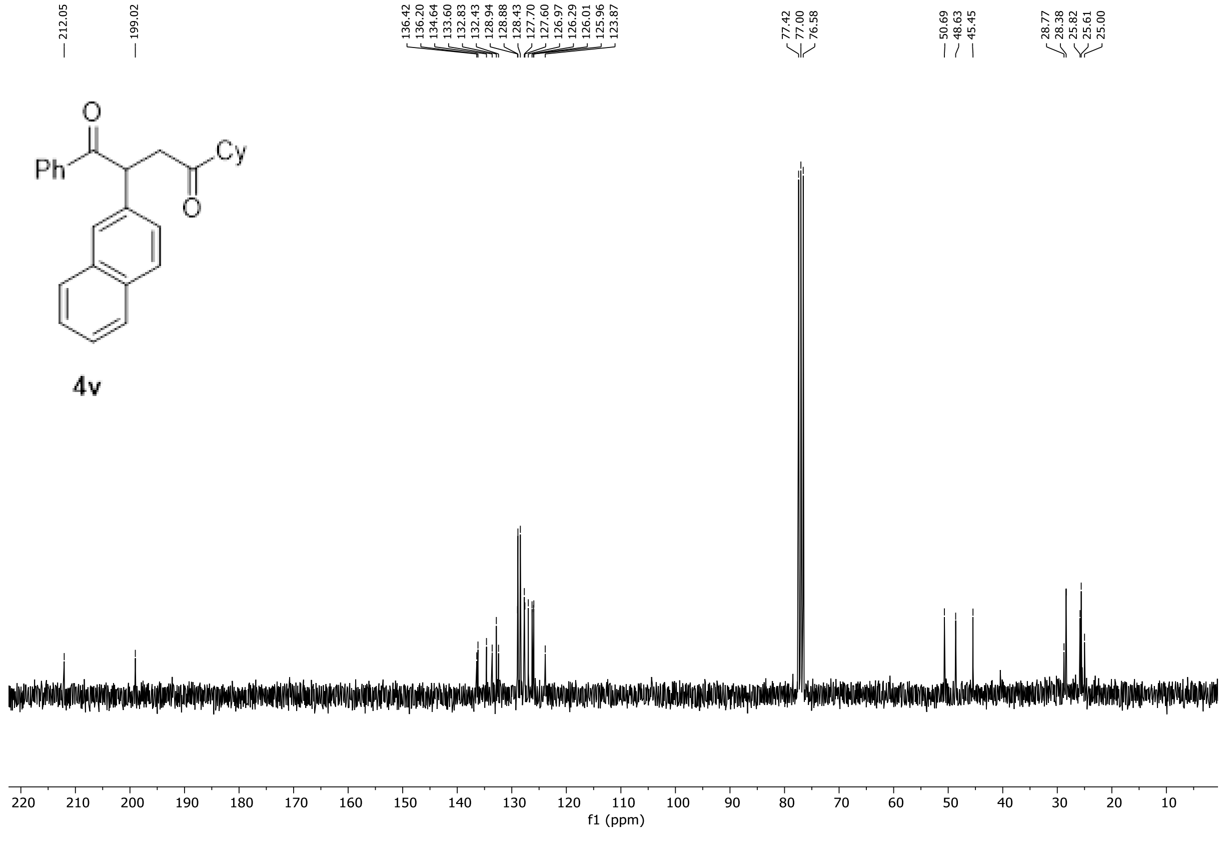

4w ^{1}H NMR (400 MHz, $CDCl_3$)

251020.405.10.fid — Mao-Lin Yang YML-5-1011-19 — Au1H CDCl3 {C:\Bruker\TopSpin3.6.2} 2510 5

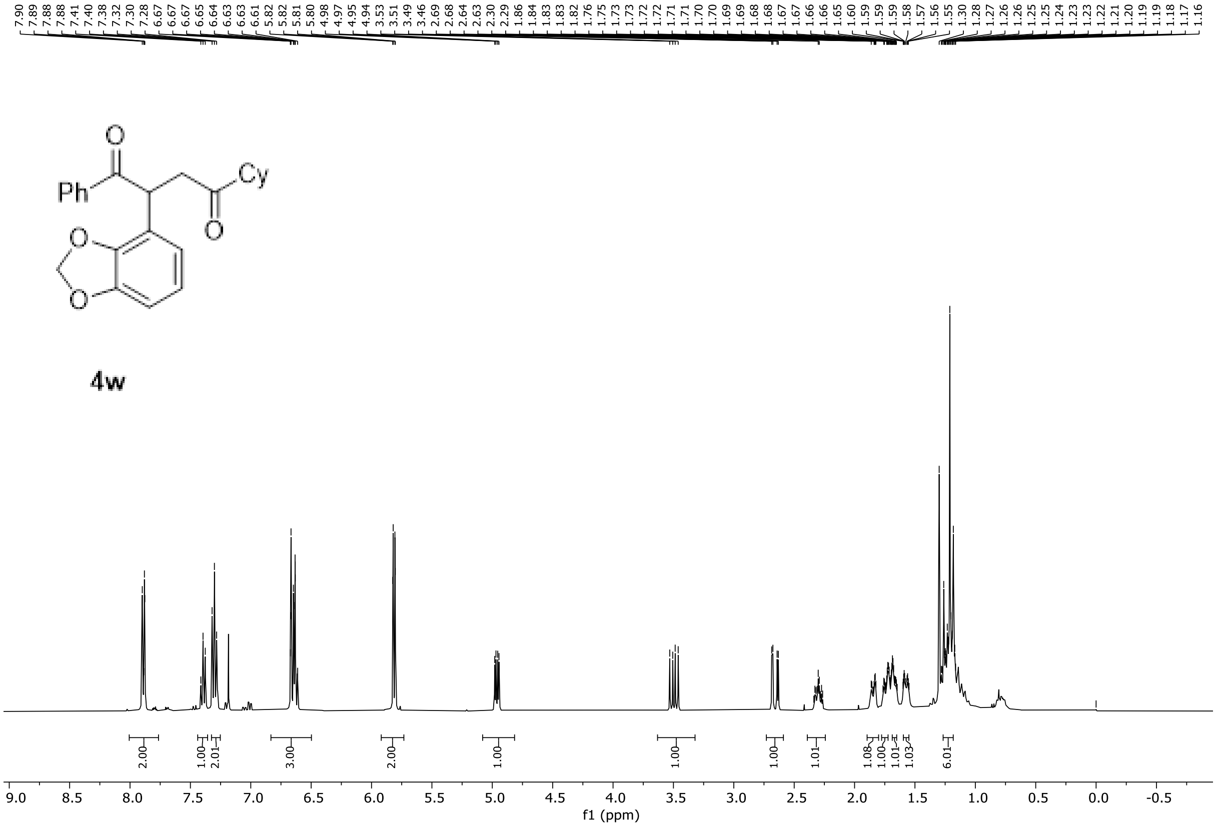

4w ^{13}C NMR (101 MHz, $CDCl_3$)

251020.405.11.fid — Mao-Lin Yang YML-5-1011-19 — Au13C CDCl3 {C:\Bruker\TopSpin3.6.2} 2510 5

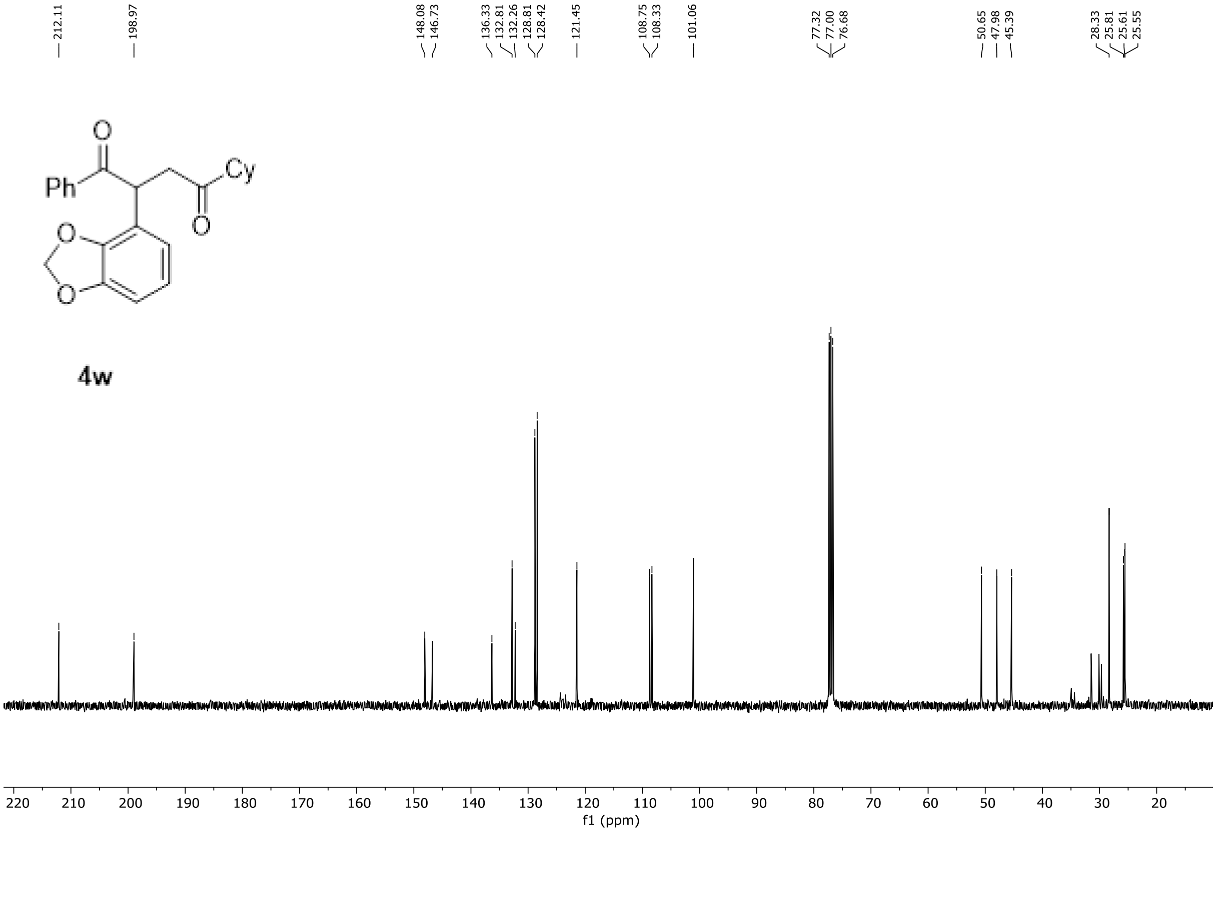

4x ^{1}H NMR (300 MHz, $CDCl_3$)

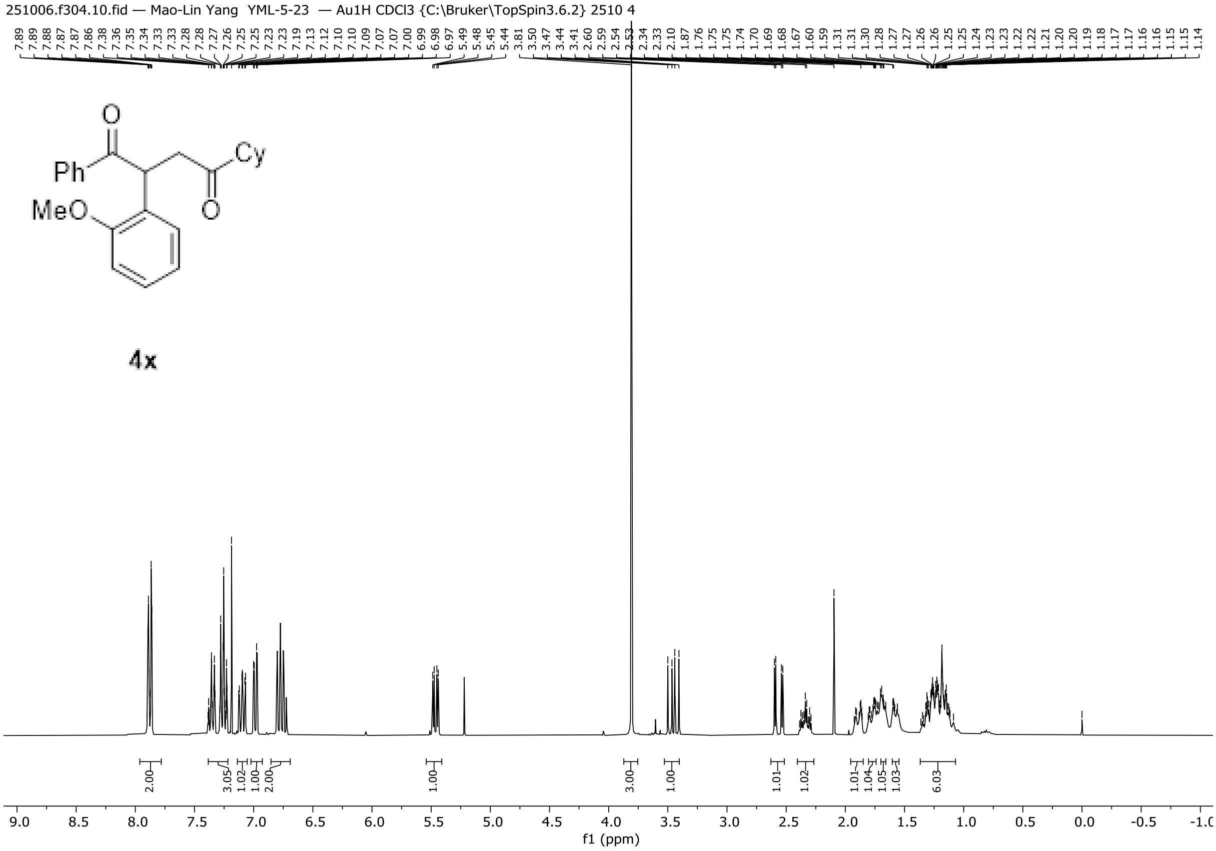

4x ^{13}C NMR (75 MHz, $CDCl_3$)

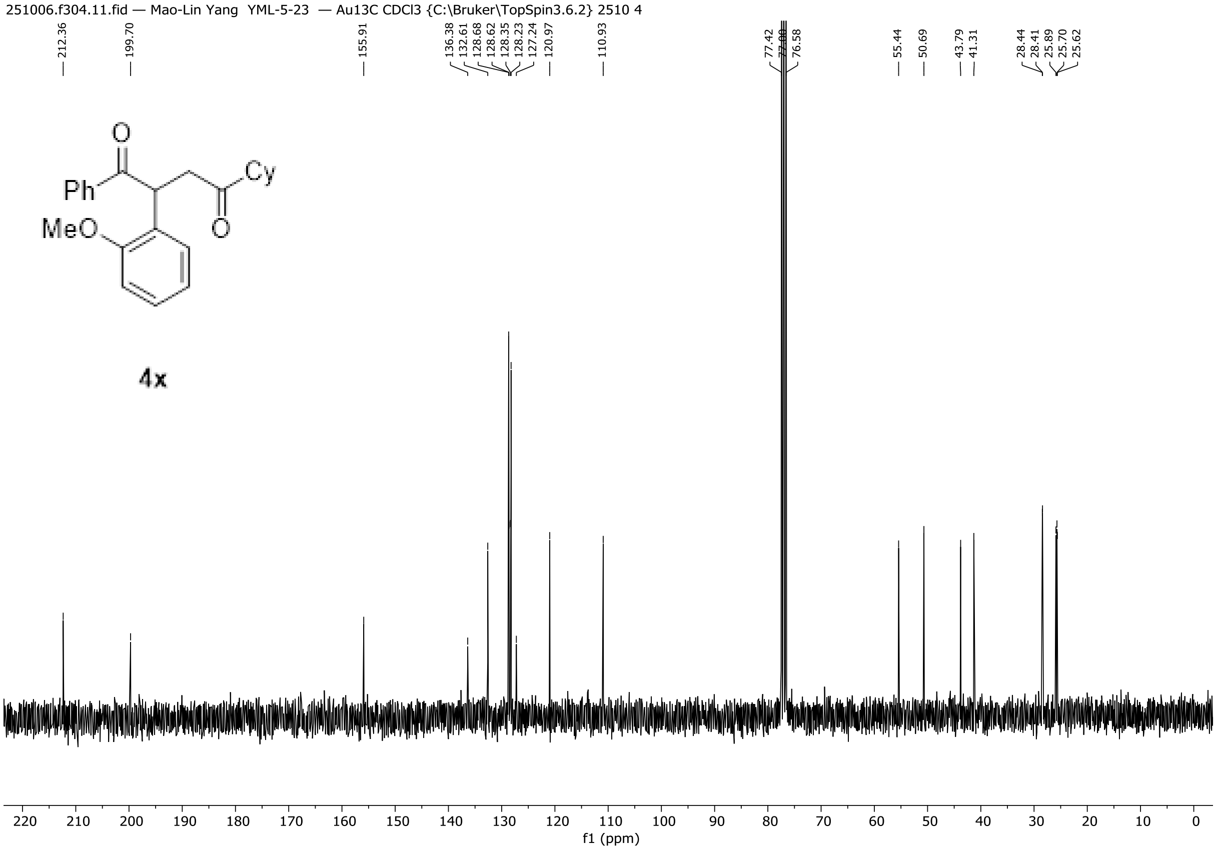

4y ^{1}H NMR (300 MHz, $CDCl_3$)

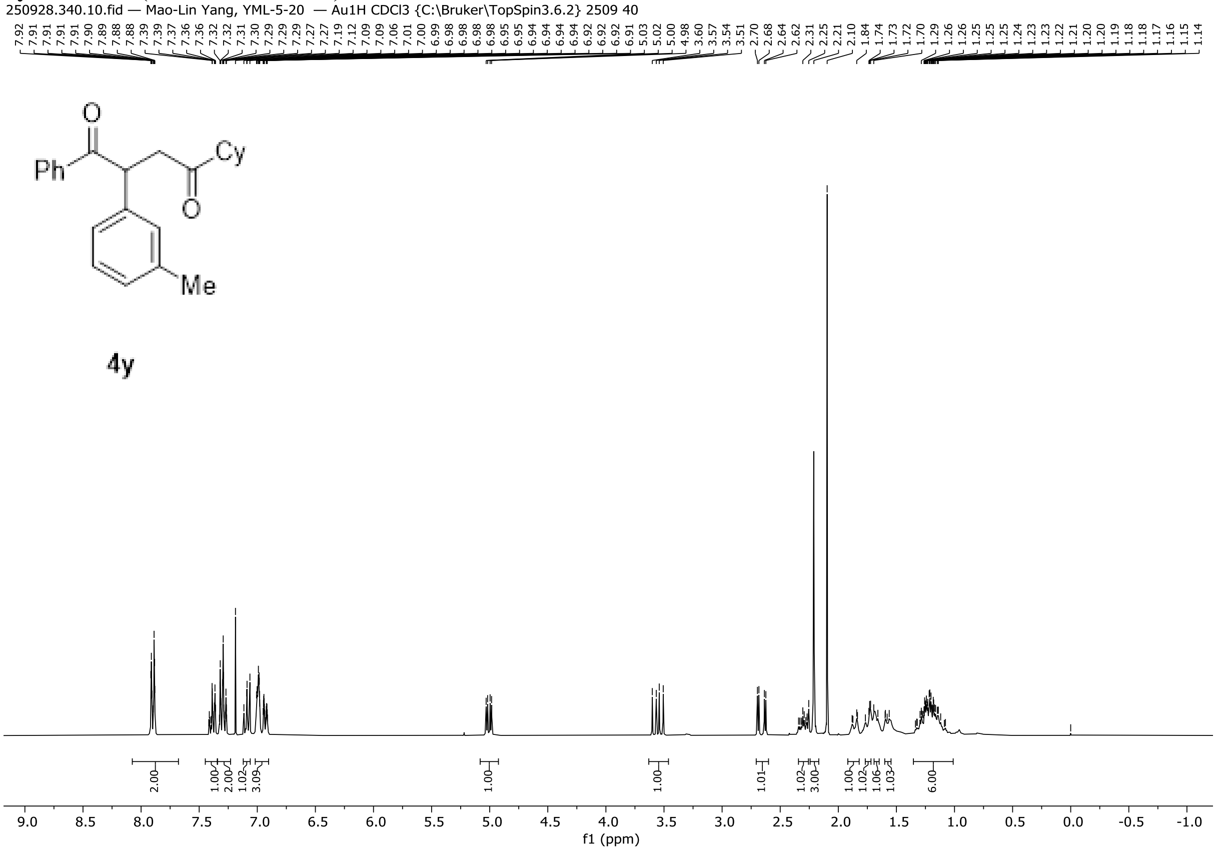

4y ^{13}C NMR (75 MHz, $CDCl_3$)

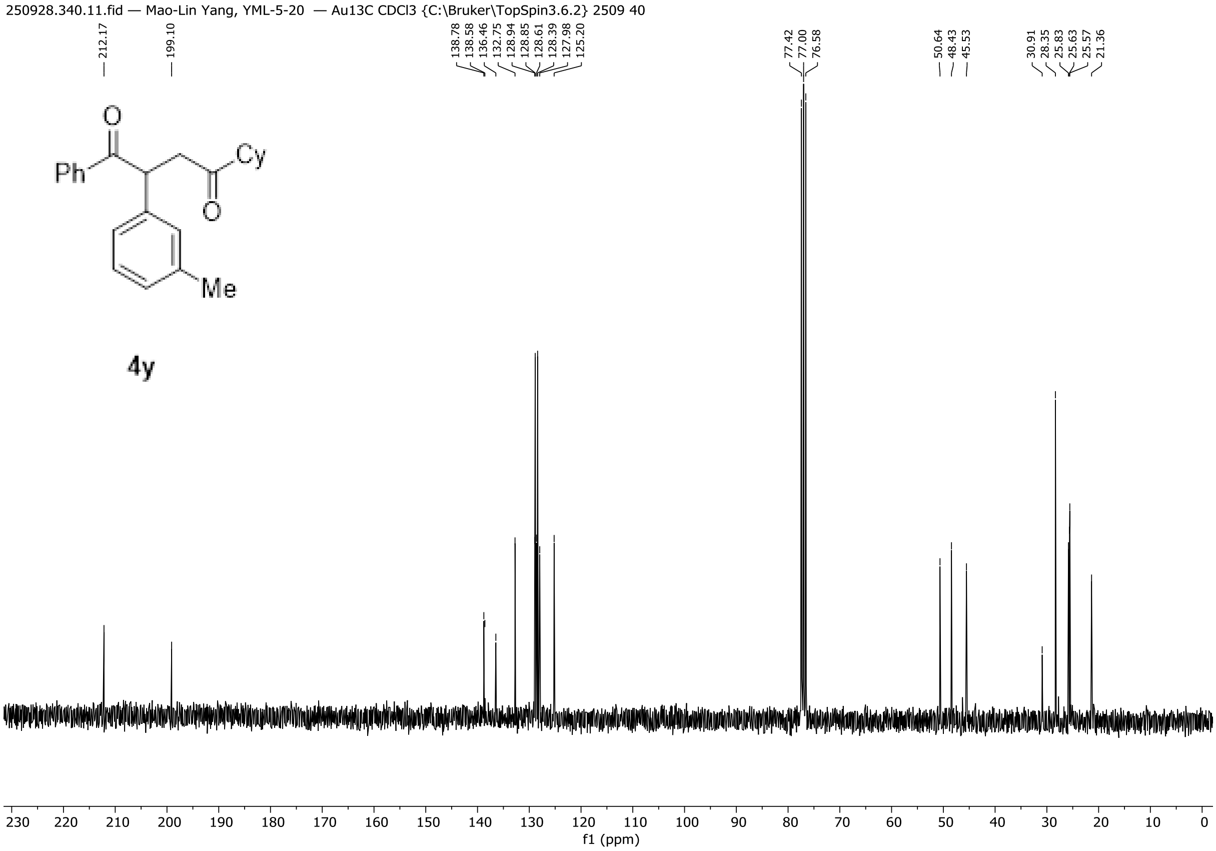

4z ^{1}H NMR (300 MHz, $CDCl_3$)

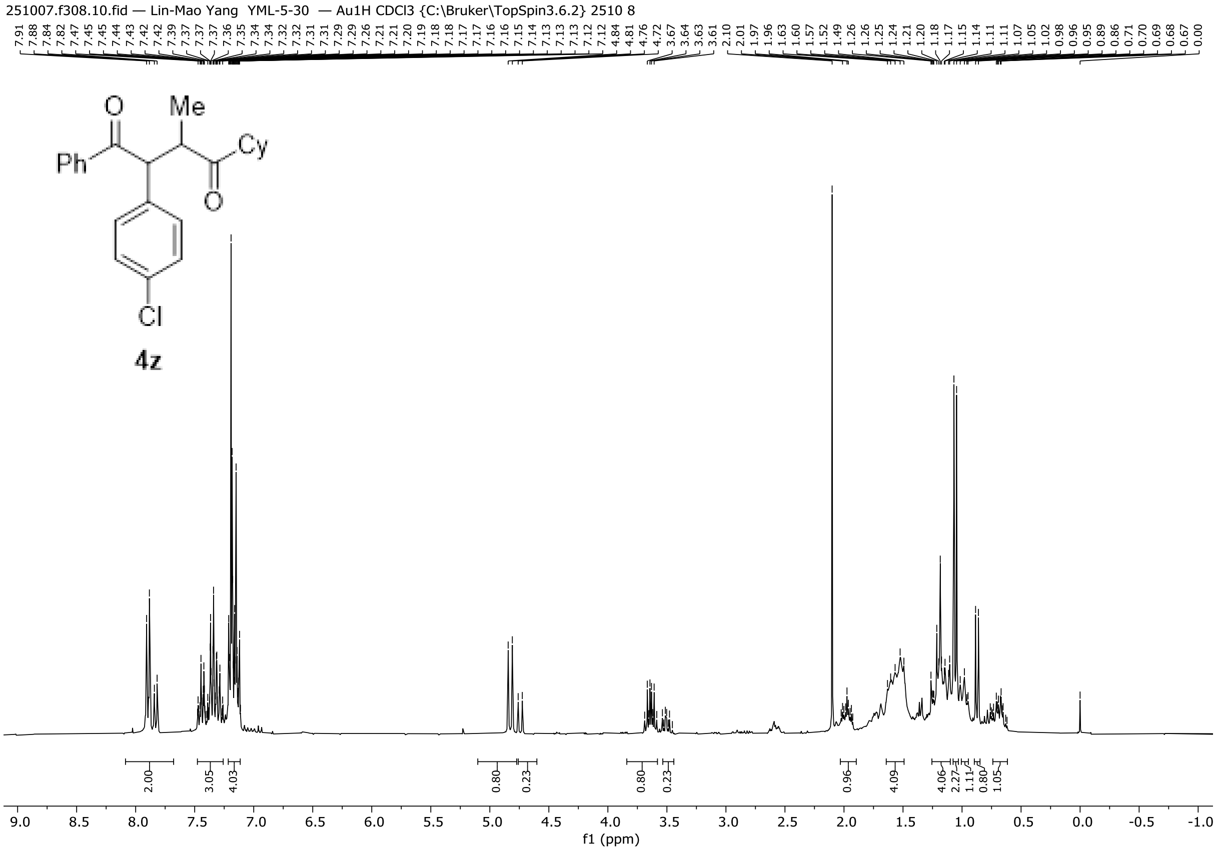

4z ^{13}C NMR (75 MHz, $CDCl_3$)

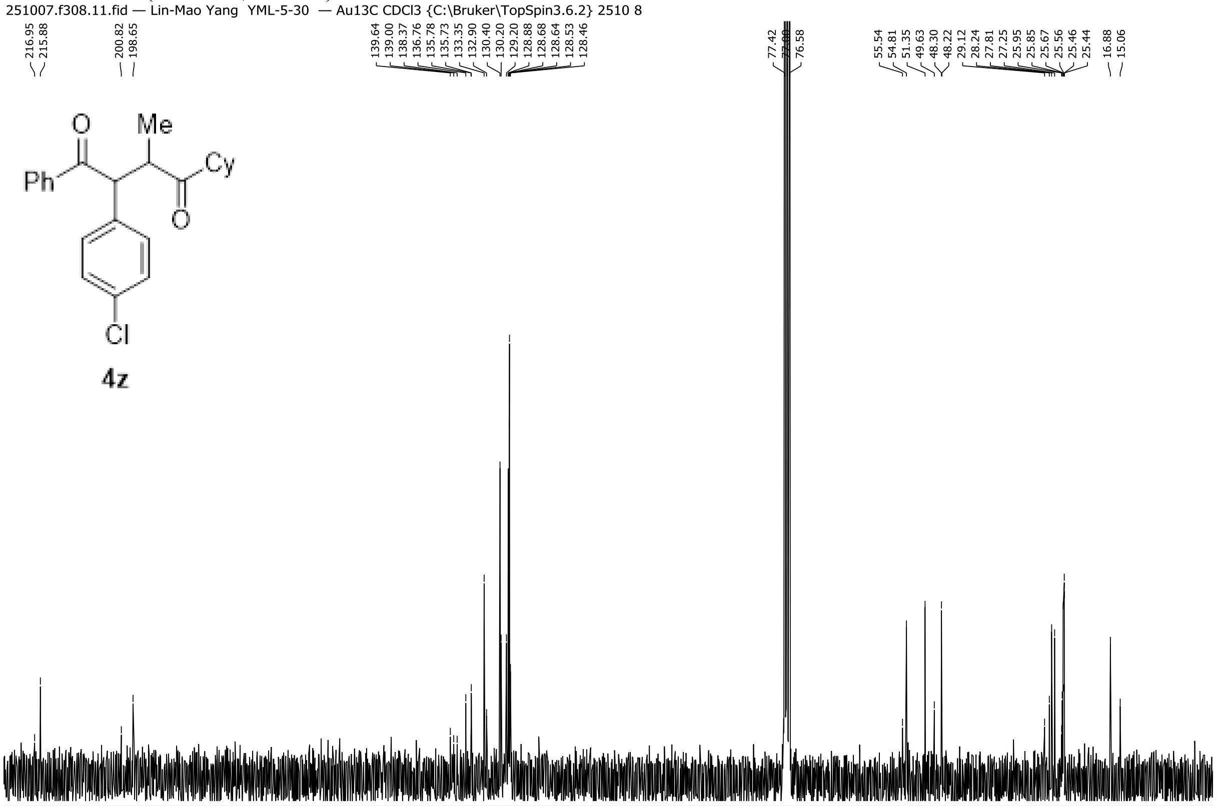

4aa ^{1}H NMR (300 MHz, $CDCl_3$)

251024.316.10.fid — Lin-Mao Yang YML-5-1021-1 — Au1H CDCl3 {C:\Bruker\TopSpin3.6.2} 2510 16

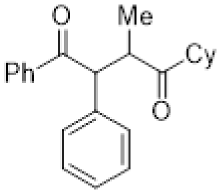

4aa

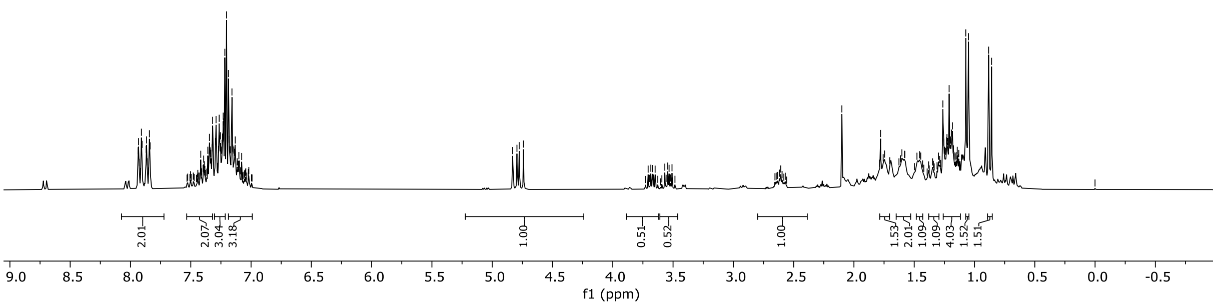

4aa ^{13}C NMR (75 MHz, $CDCl_3$)

251024.316.11.fid — Lin-Mao Yang YML-5-1021-1 — Au13C CDCl3 {C:\Bruker\TopSpin3.6.2} 2510 16

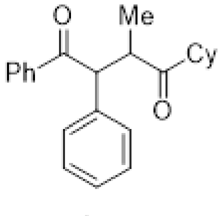

4aa

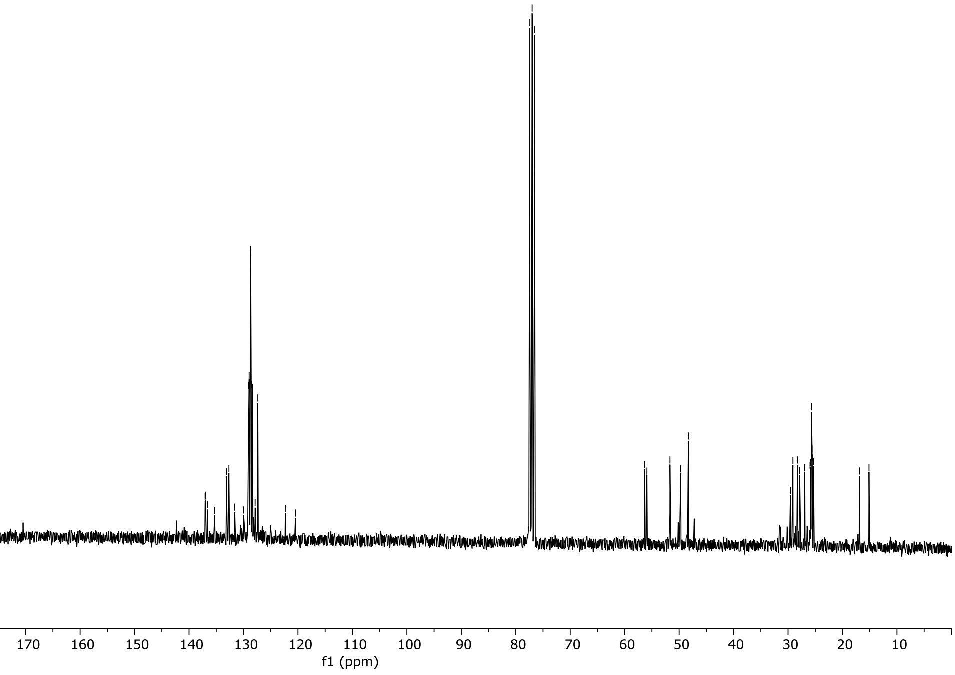

5a ^{1}H NMR (300 MHz, $CDCl_3$)

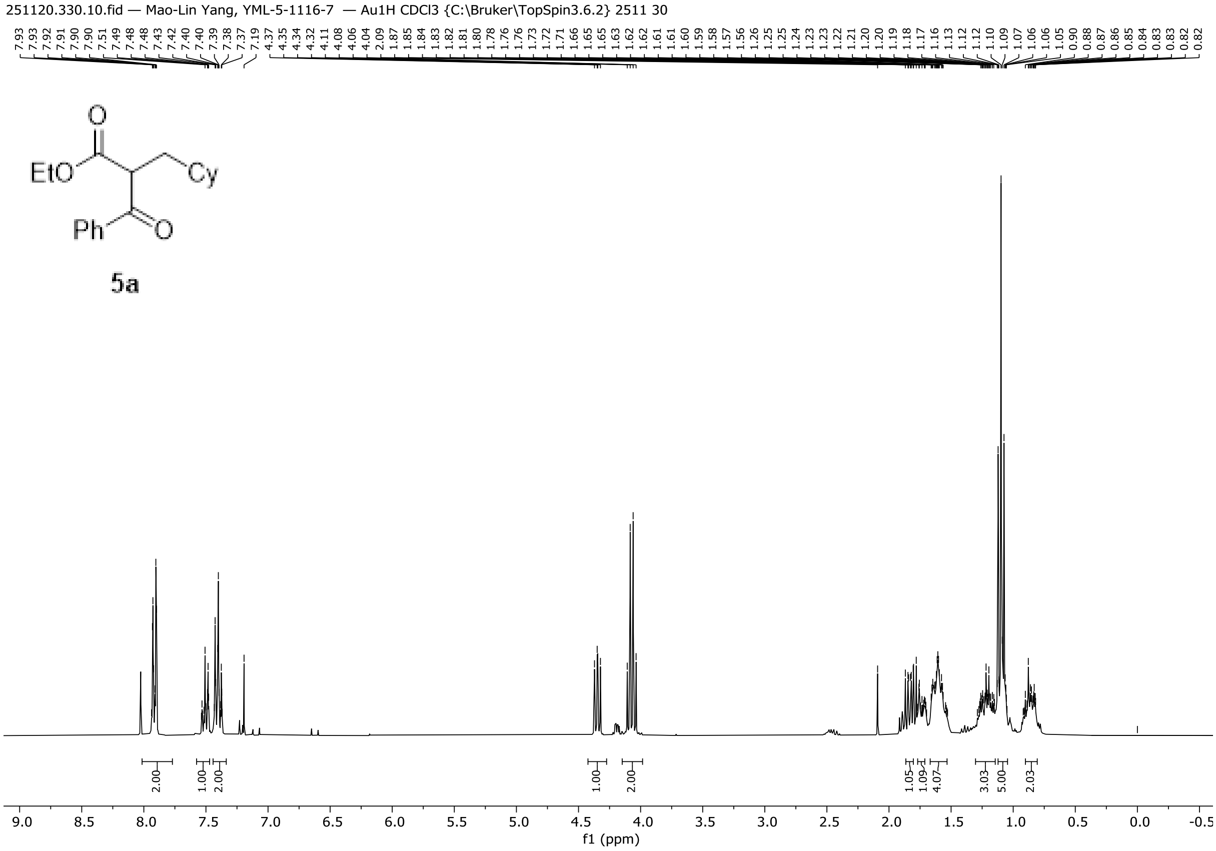

5a ^{13}C NMR (75 MHz, $CDCl_3$)

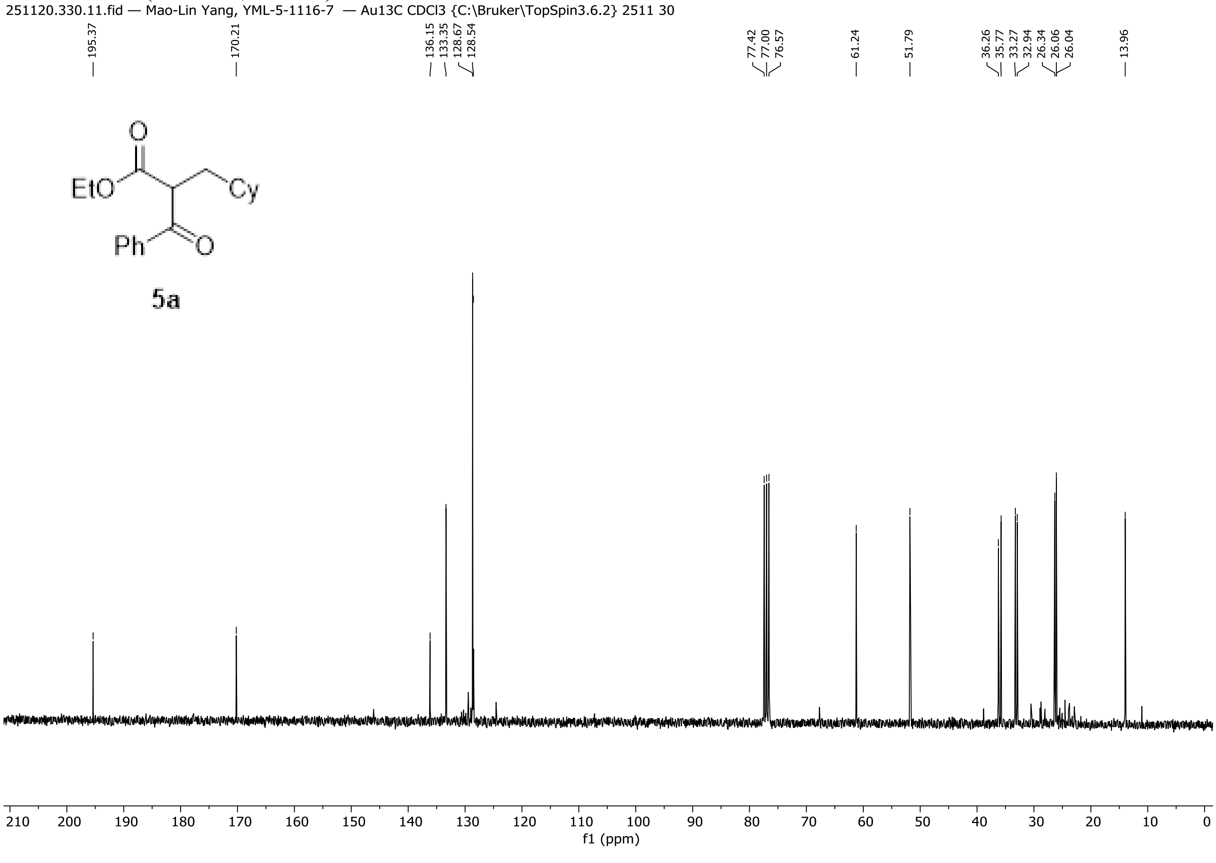

5b ^{1}H NMR (300 MHz, $CDCl_3$)

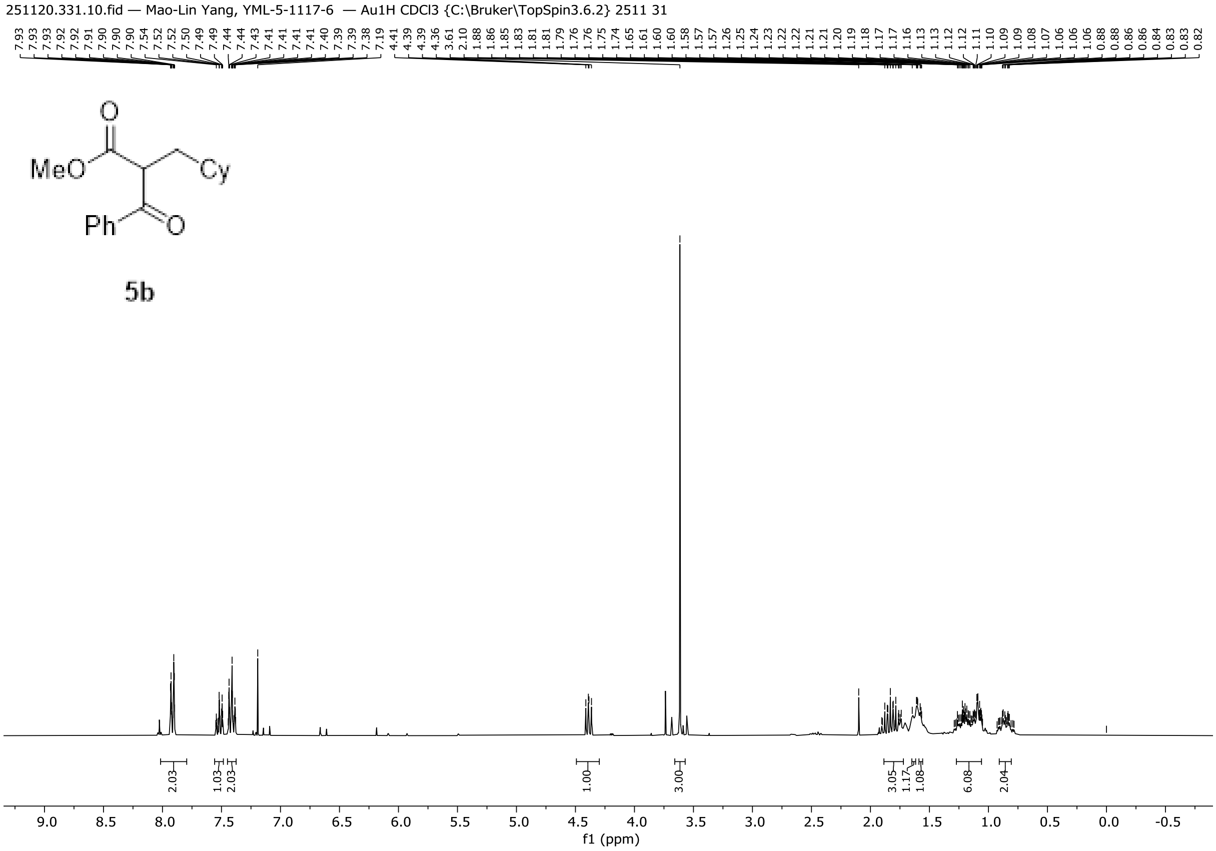

5b ^{13}C NMR (75 MHz, $CDCl_3$)

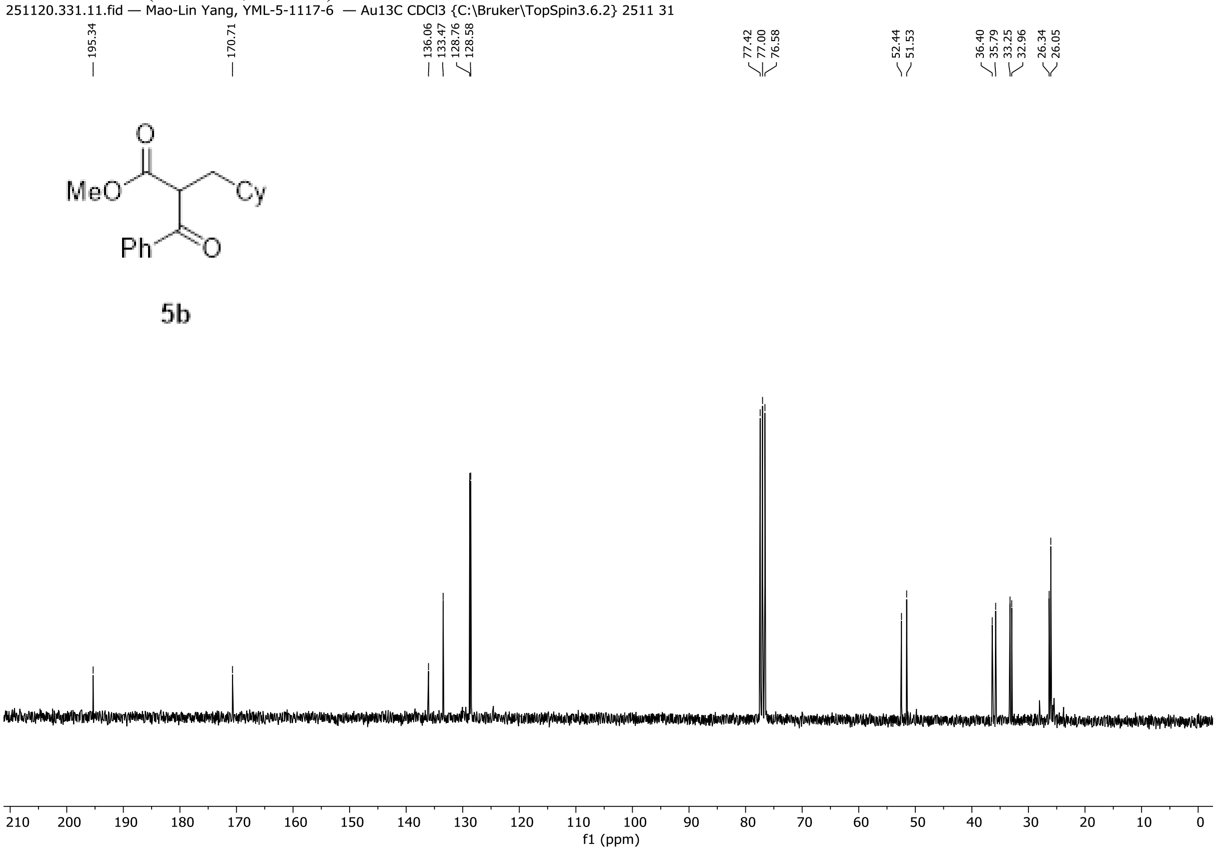

4ab ^{1}H NMR (300 MHz, $CDCl_3$)

251024.f305.10.fid — Lin-Mao Yang YML-5-1009-8 — Au1H CDCl3 {C:\Bruker\TopSpin3.6.2} 2510 5

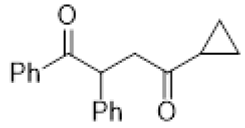

4ab

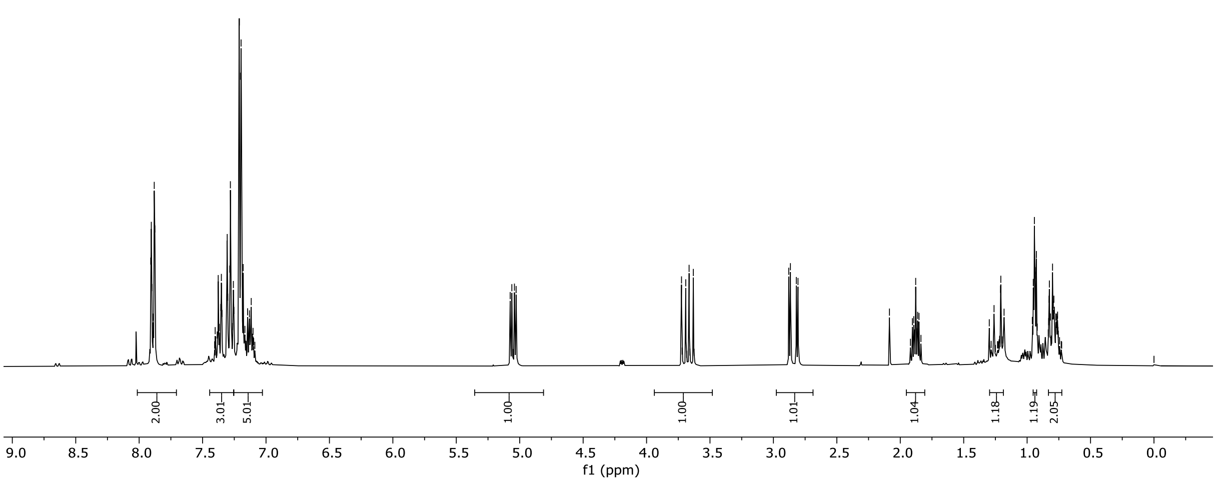

4ab ^{13}C NMR (75 MHz, $CDCl_3$)

251024.f305.11.fid — Lin-Mao Yang YML-5-1009-8 — Au13C CDCl3 {C:\Bruker\TopSpin3.6.2} 2510 5

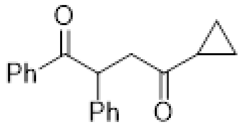

4ab

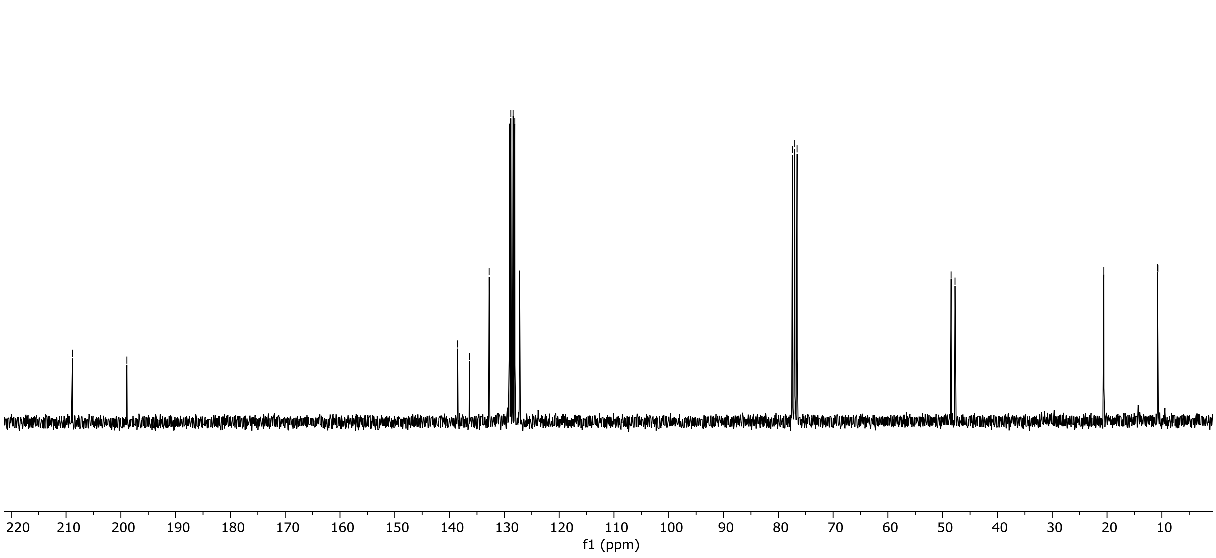

4ac ^{1}H NMR (300 MHz, $CDCl_3$)

251006.303.10.fid — Mao-Lin Yang YML-5-0927-2 — Au1H CDCl3 {C:\Bruker\TopSpin3.6.2} 2510 3

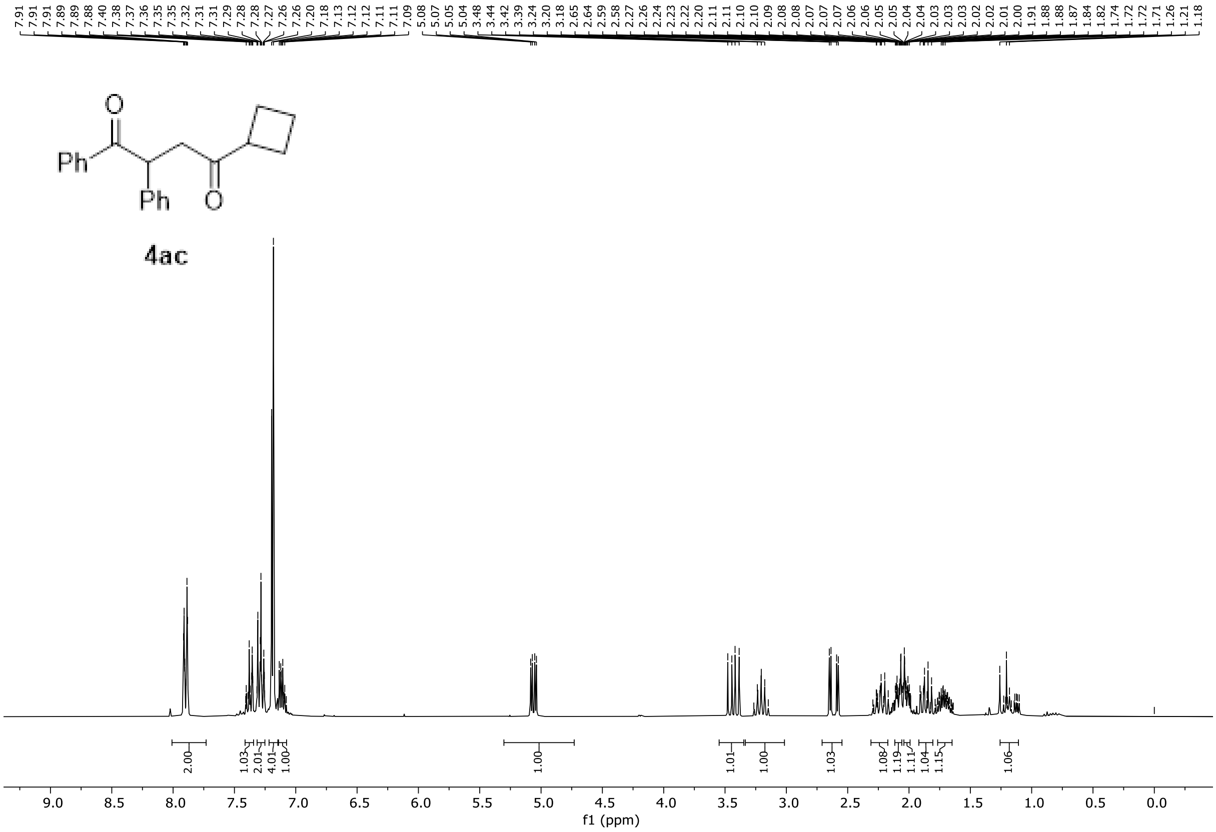

4ac ^{13}C NMR (75 MHz, $CDCl_3$)

251006.303.11.fid — Mao-Lin Yang YML-5-0927-2 — Au13C CDCl3 {C:\Bruker\TopSpin3.6.2} 2510 3

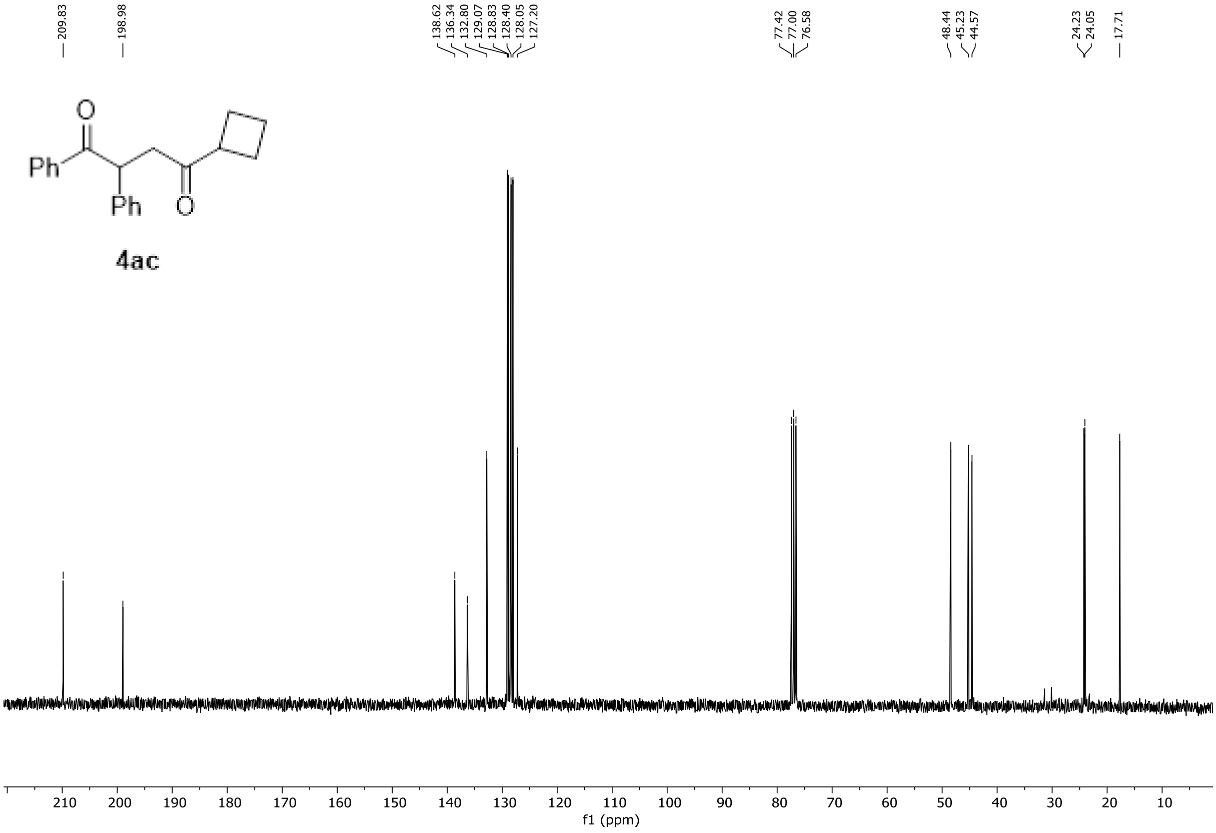

4ad ^{1}H NMR (300 MHz, $CDCl_3$)

251006.304.10.fid — Lin-Mao Yang YML-0927-3 — Au1H CDCl3 {C:\Bruker\TopSpin3.6.2} 2510 4

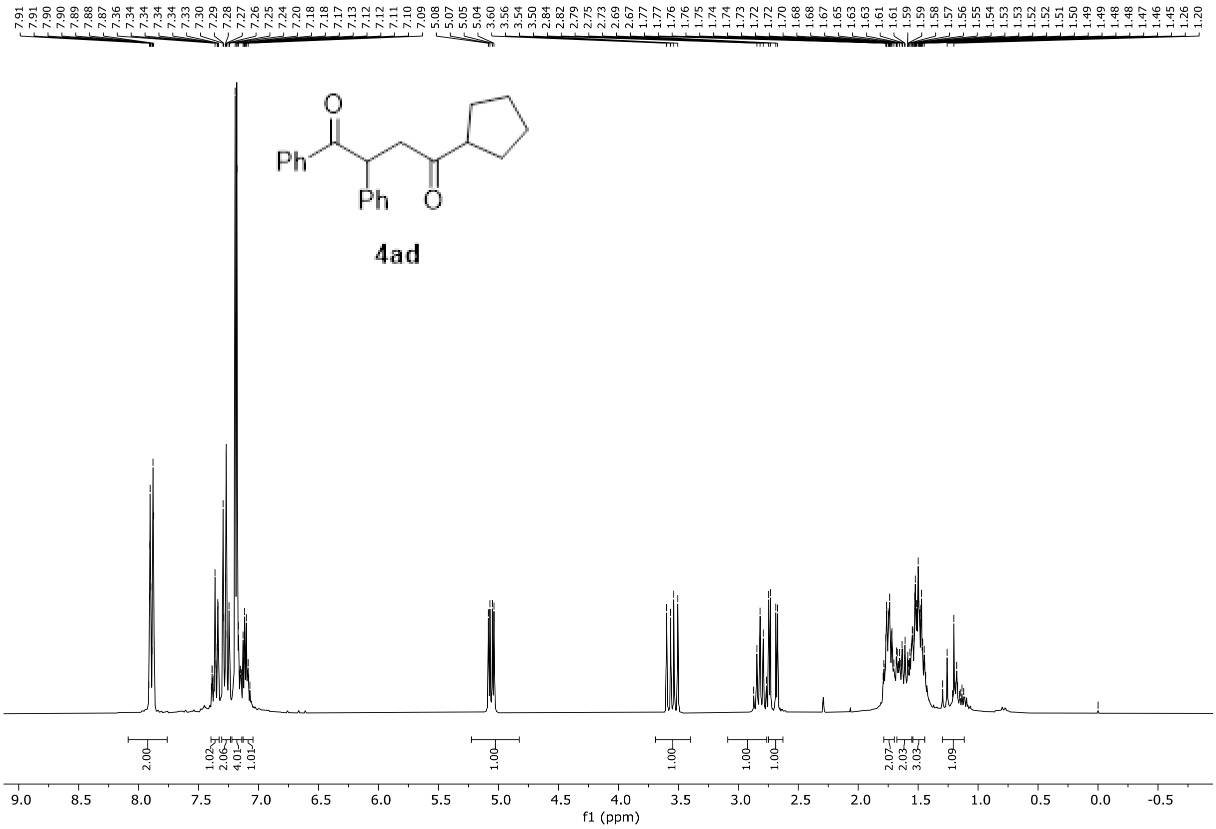

4ad ^{13}C NMR (75 MHz, $CDCl_3$)

251006.304.11.fid — Lin-Mao Yang YML-0927-3 — Au13C CDCl3 {C:\Bruker\TopSpin3.6.2} 2510 4

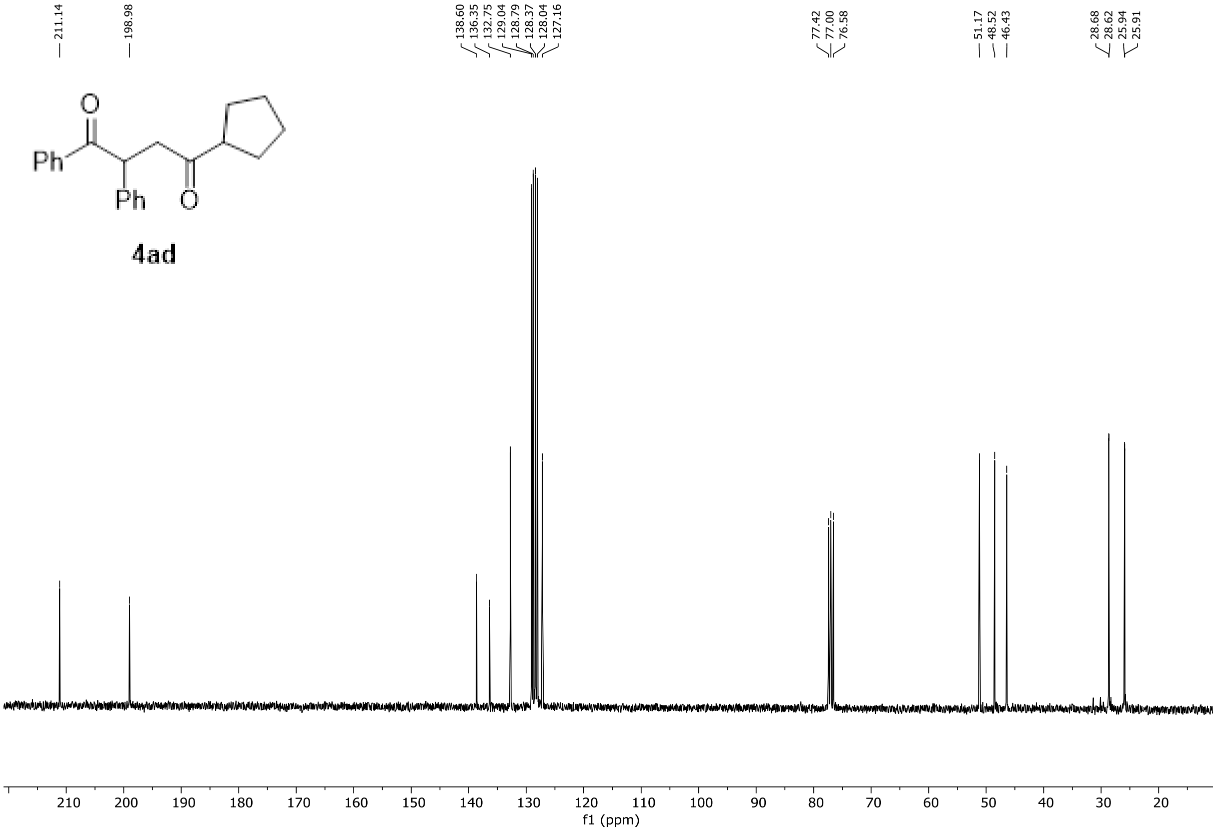

4ae ^{1}H NMR (400 MHz, $CDCl_3$)

251022.412.10.fid — Lin-Mao Yang YML-5-1017-6 — Au1H CDCl3 {C:\Bruker\TopSpin3.6.2} 2510 12

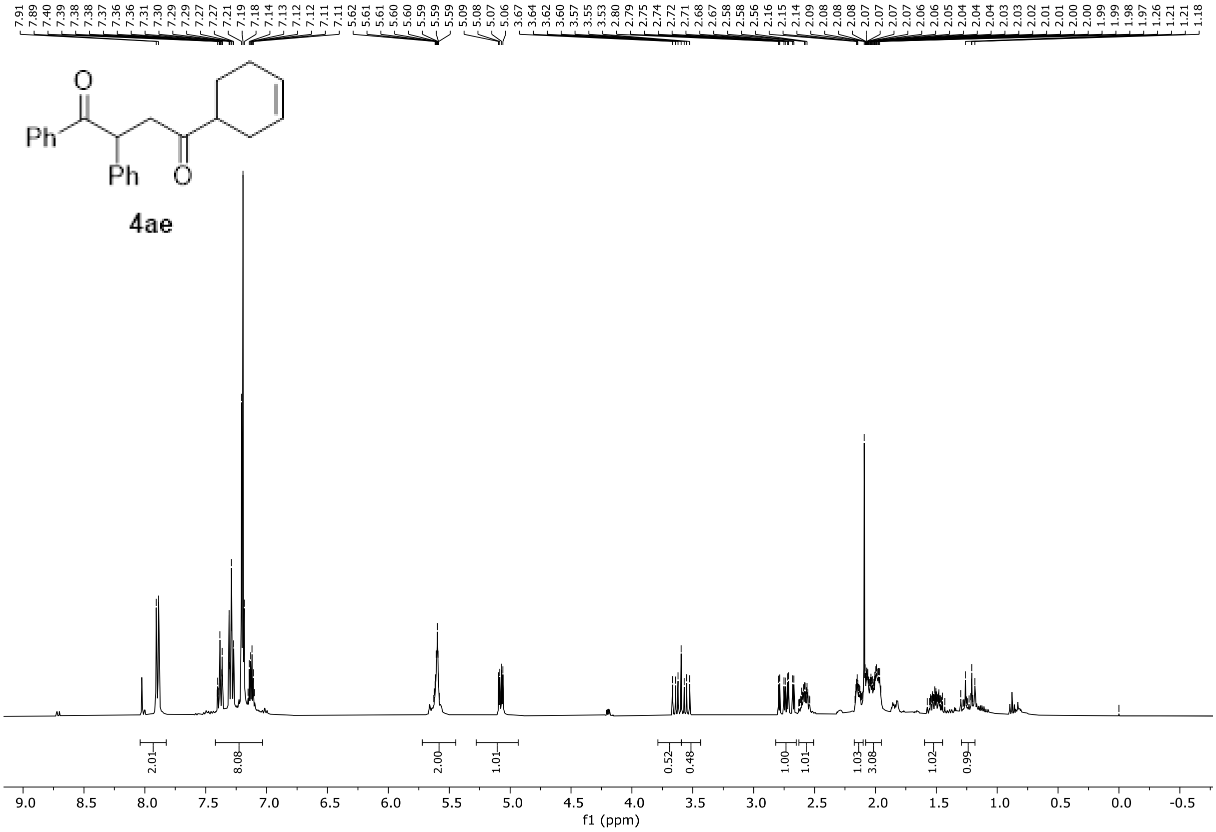

4ae ^{13}C NMR (101 MHz, $CDCl_3$)

251022.412.11.fid — Lin-Mao Yang YML-5-1017-6 — Au13C CDCl3 {C:\Bruker\TopSpin3.6.2} 2510 12

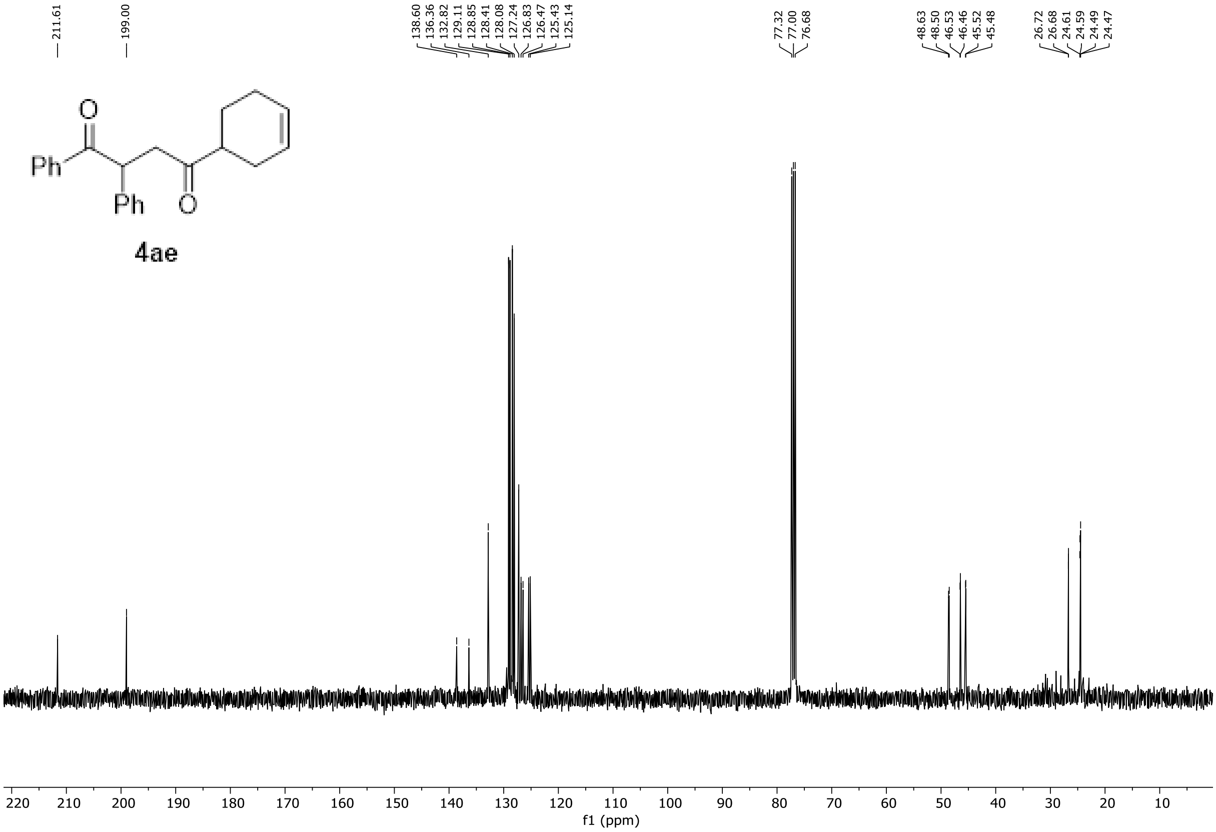

4af ^{1}H NMR (300 MHz, $CDCl_3$)

251015.f318.10.fid — Lin-Mao Yang YML-5-1011-8 — Au1H CDCl3 {C:\Bruker\TopSpin3.6.2} 2510 18

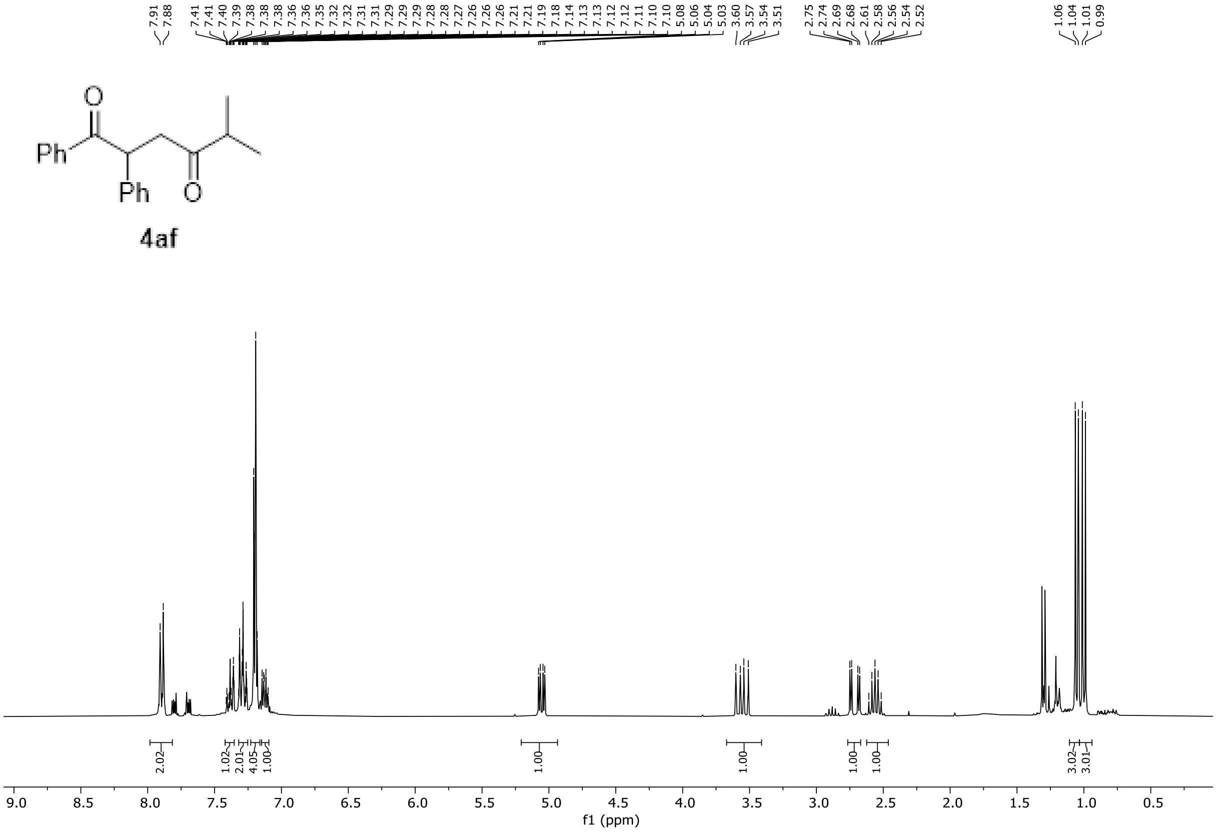

4af ^{13}C NMR (75 MHz, $CDCl_3$)

251015.f318.11.fid — Lin-Mao Yang YML-5-1011-8 — Au13C CDCl3 {C:\Bruker\TopSpin3.6.2} 2510 18

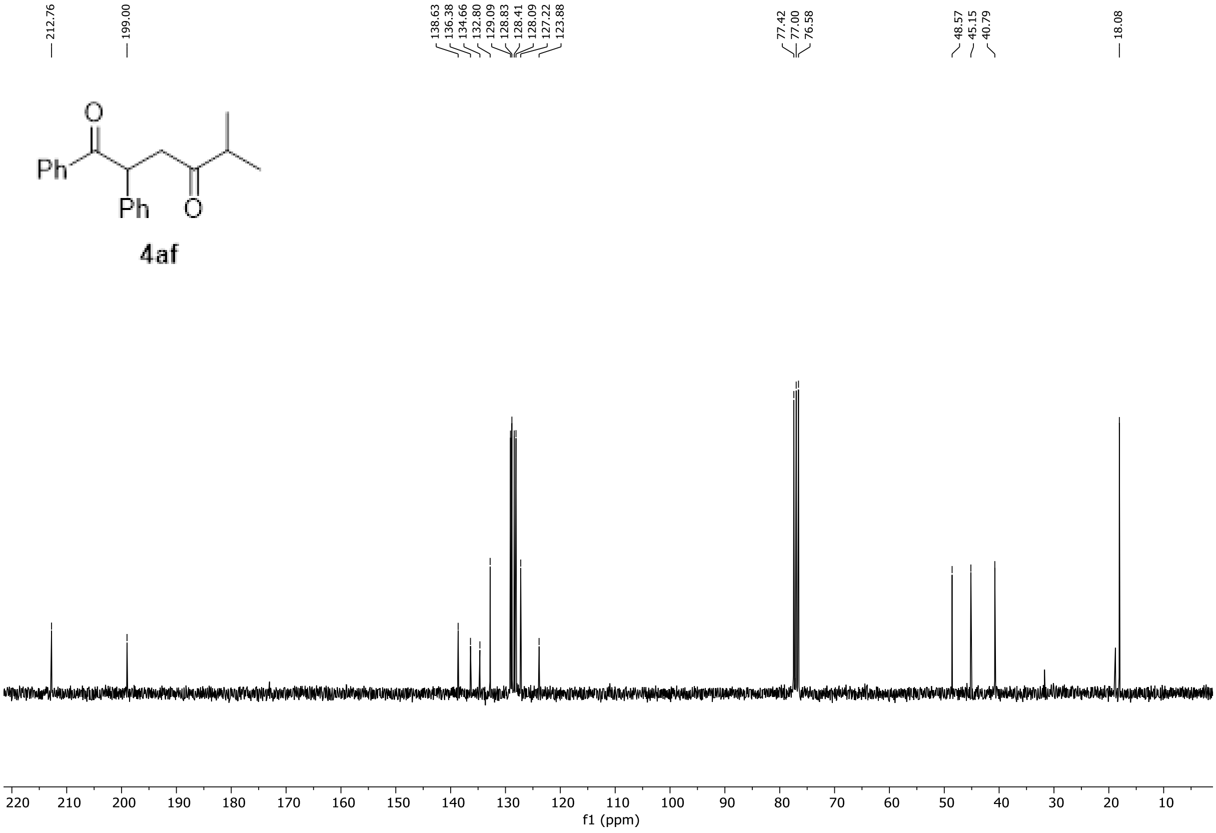

4ag ^{1}H NMR (400 MHz, $CDCl_3$)

251021.419.10.fid — Lin-Mao Yang YML-5-1017-3 — Au1H CDCl3 {C:\Bruker\TopSpin3.6.2} 2510 19

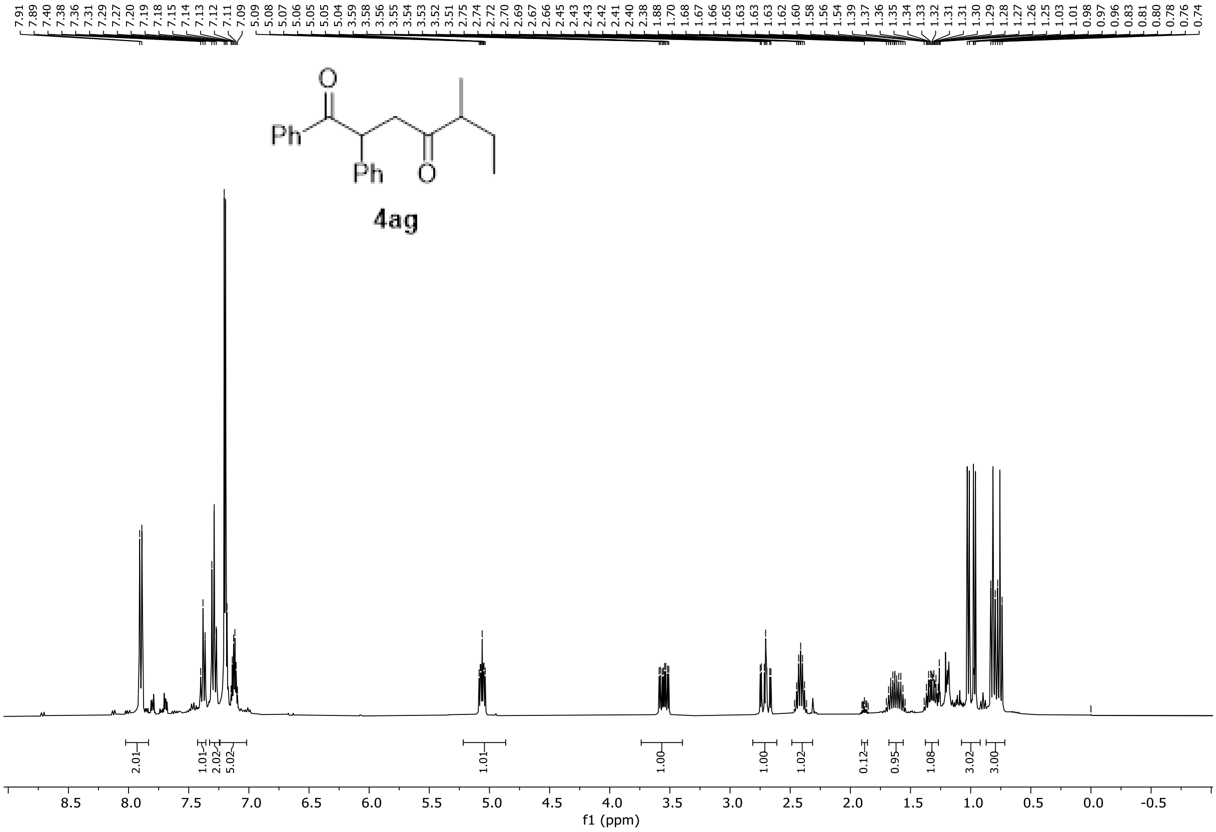

4ag ^{13}C NMR (101 MHz, $CDCl_3$)

251021.419.11.fid — Lin-Mao Yang YML-5-1017-3 — Au13C CDCl3 {C:\Bruker\TopSpin3.6.2} 2510 19

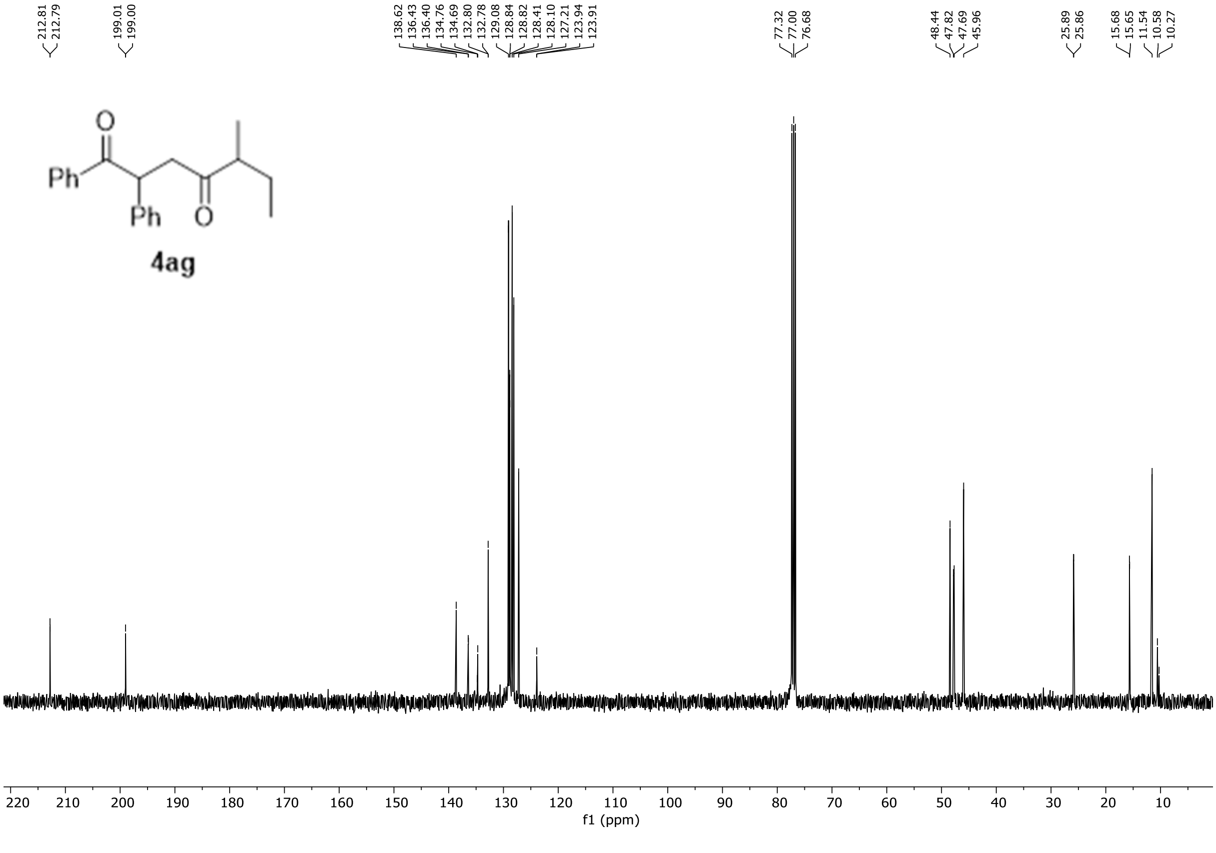

4ah ^{1}H NMR (400 MHz, $CDCl_3$)

251021.421.10.fid — Lin-Mao Yang YML-5-1017-5 — Au1H CDCl3 {C:\Bruker\TopSpin3.6.2} 2510 21

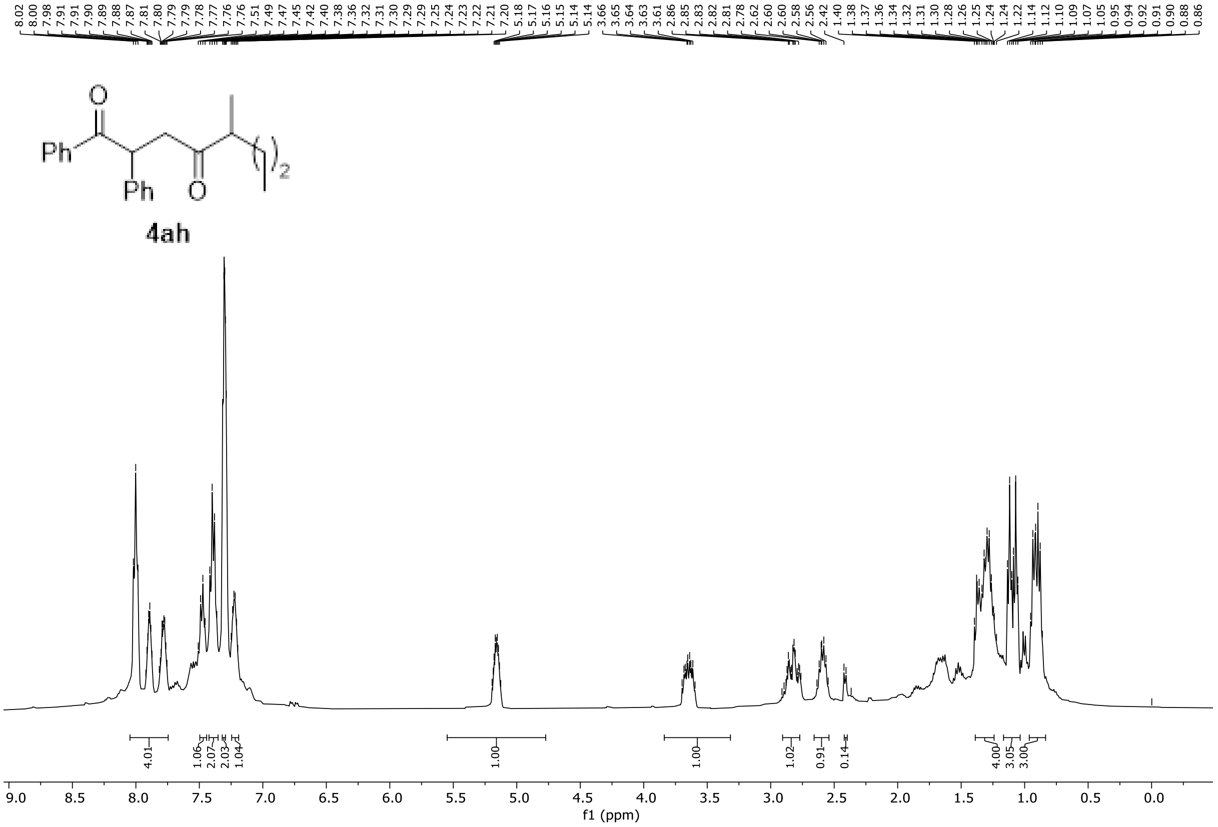

4ah ^{13}C NMR (101 MHz, $CDCl_3$)

251021.421.11.fid — Lin-Mao Yang YML-5-1017-5 — Au13C CDCl3 {C:\Bruker\TopSpin3.6.2} 2510 21

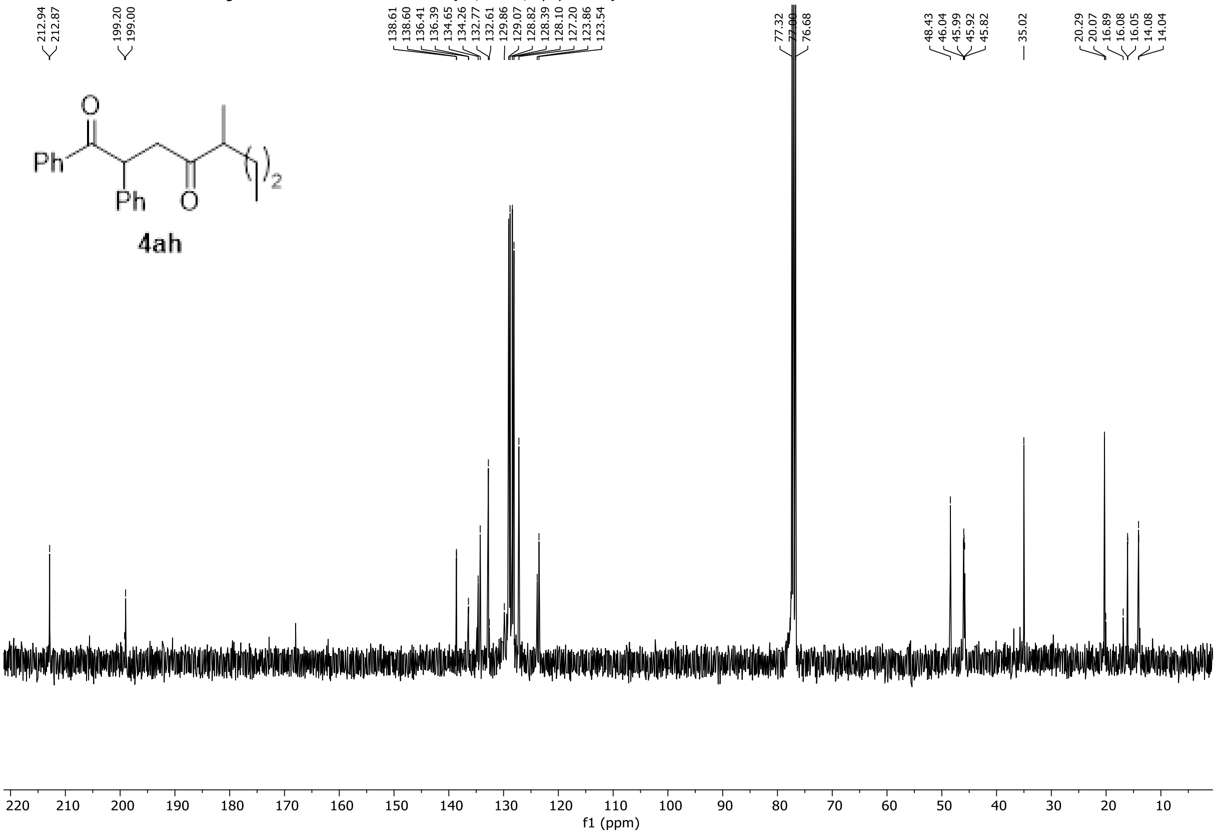

4ai ^{1}H NMR (400 MHz, $CDCl_3$)

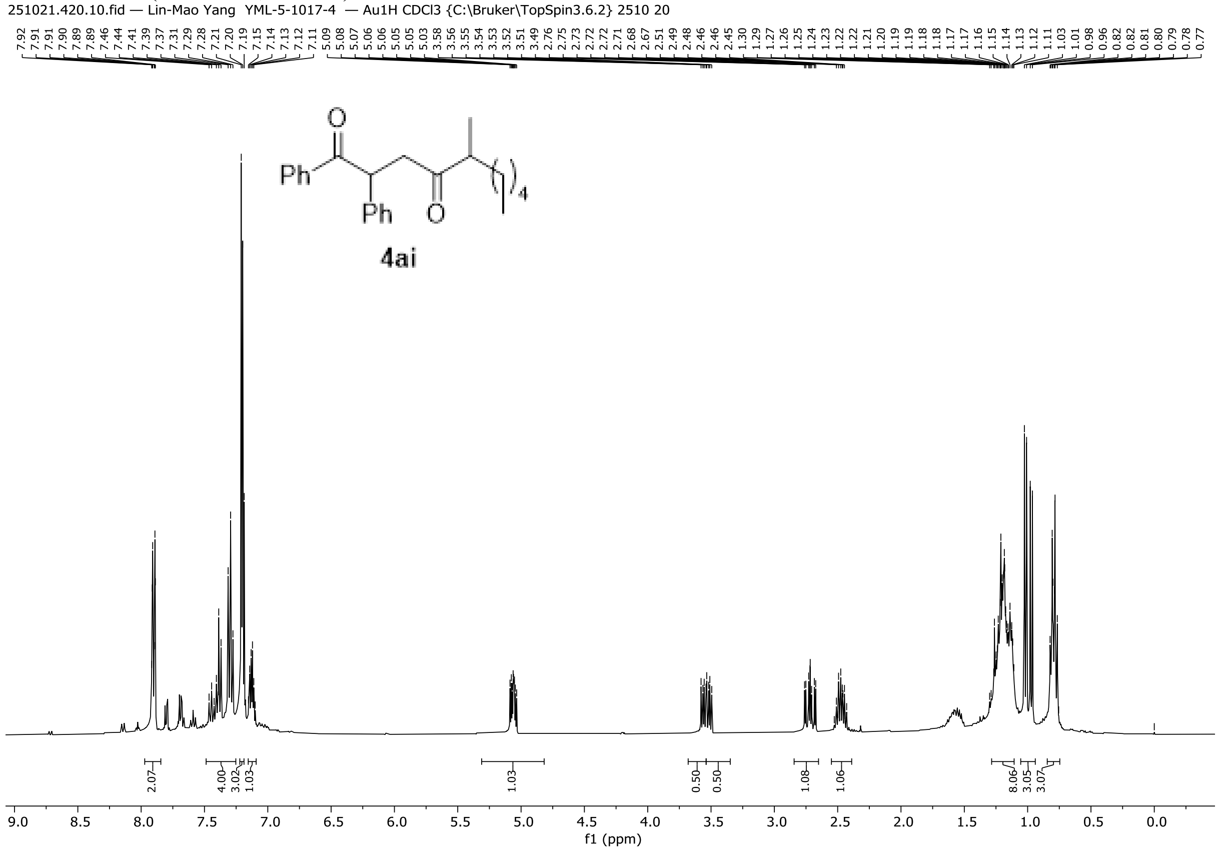

4ai ^{13}C NMR (101 MHz, $CDCl_3$)

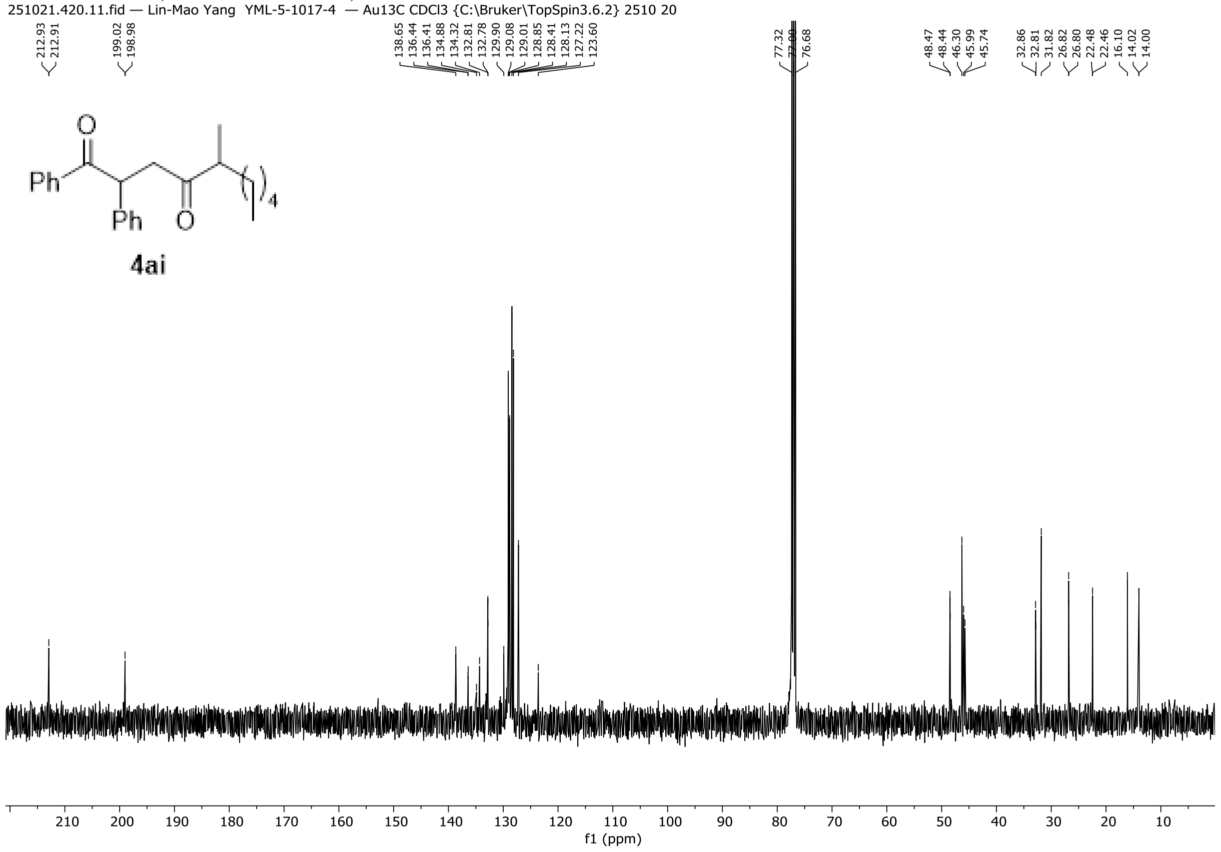

4aj ^{1}H NMR (300 MHz, $CDCl_3$)

251020.f328.10.fid — Lin-Mao Yang YML-5-1017-2 — Au1H CDCl3 {C:\Bruker\TopSpin3.6.2} 2510 28

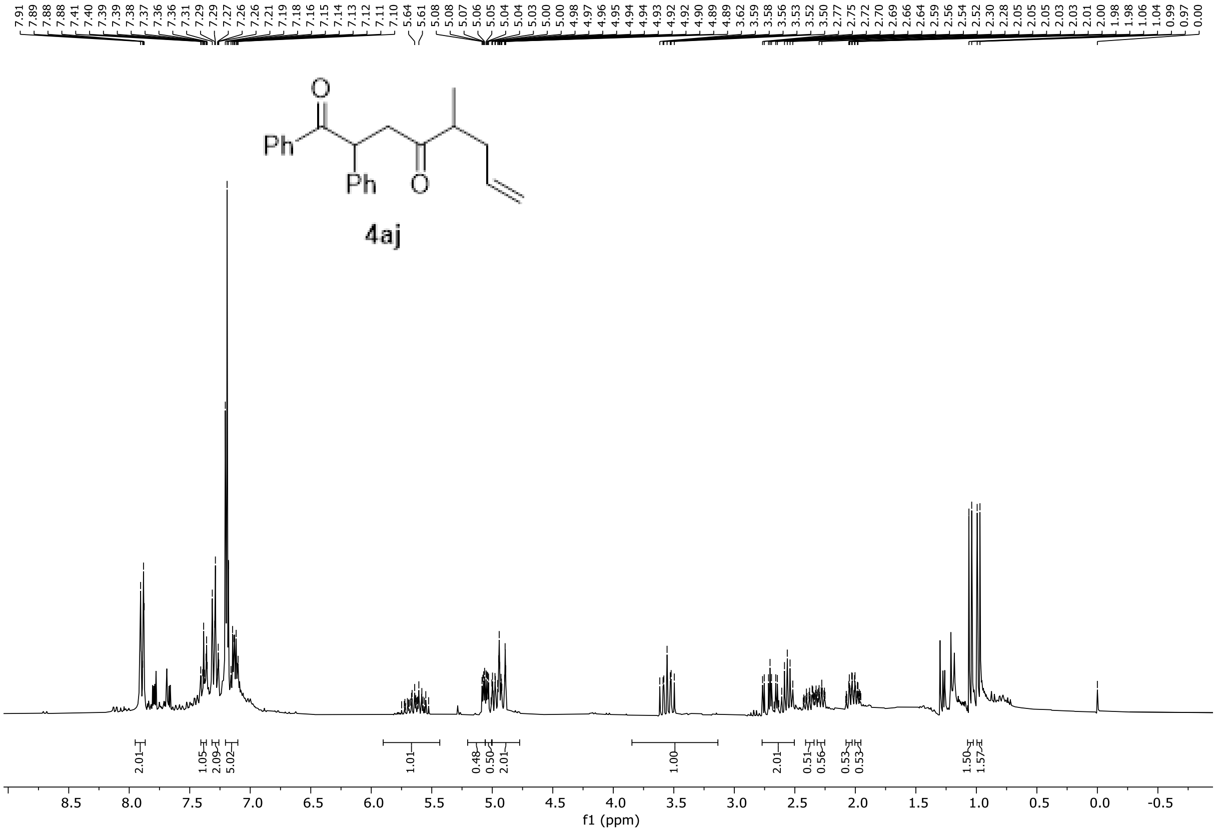

4aj ^{13}C NMR (75 MHz, $CDCl_3$)

251020.f328.11.fid — Lin-Mao Yang YML-5-1017-2 — Au13C CDCl3 {C:\Bruker\TopSpin3.6.2} 2510 28

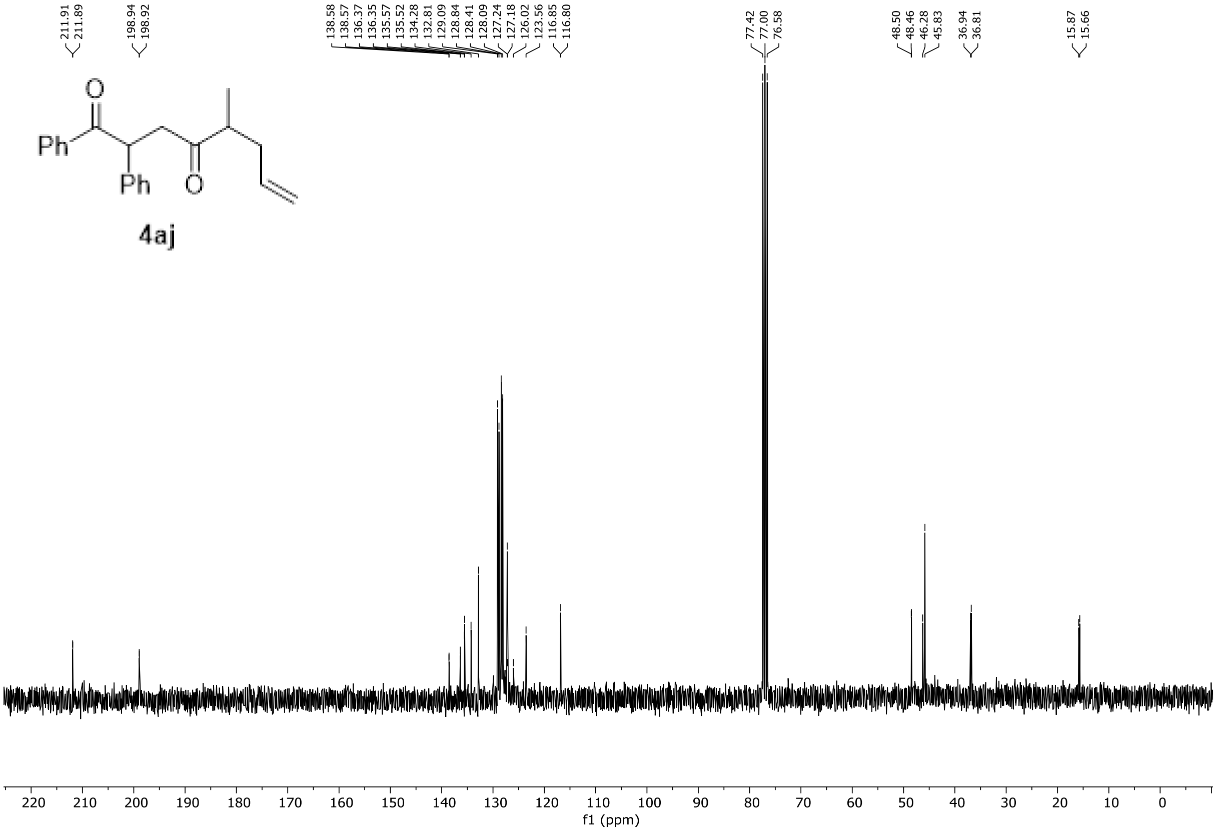

4ak ^{1}H NMR (400 MHz, $CDCl_3$)

251016.406.10.fid — Mao-Lin Yang YML-5-1009-9 — Au1H CDCl3 {C:\Bruker\TopSpin3.6.2} 2510 6

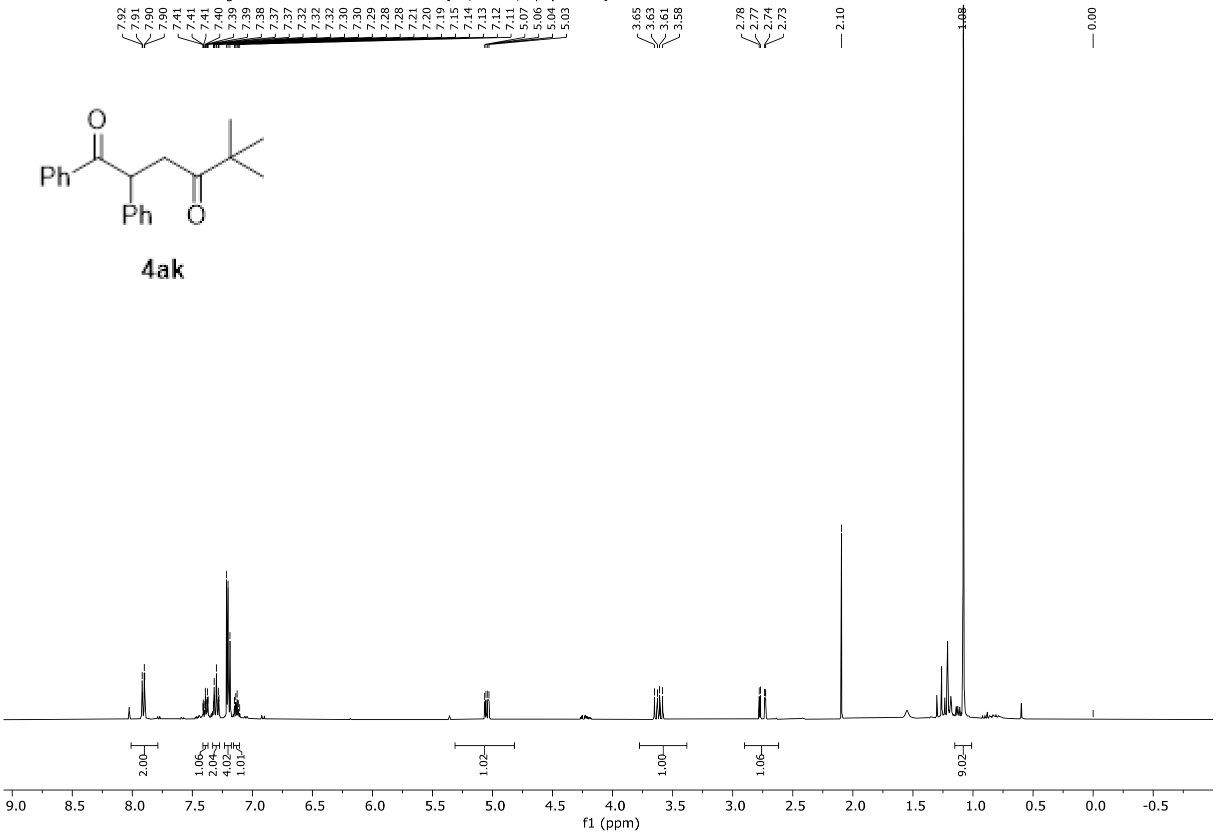

4ak ^{13}C NMR (101 MHz, $CDCl_3$)

251016.406.11.fid — Mao-Lin Yang YML-5-1009-9 — Au13C CDCl3 {C:\Bruker\TopSpin3.6.2} 2510 6

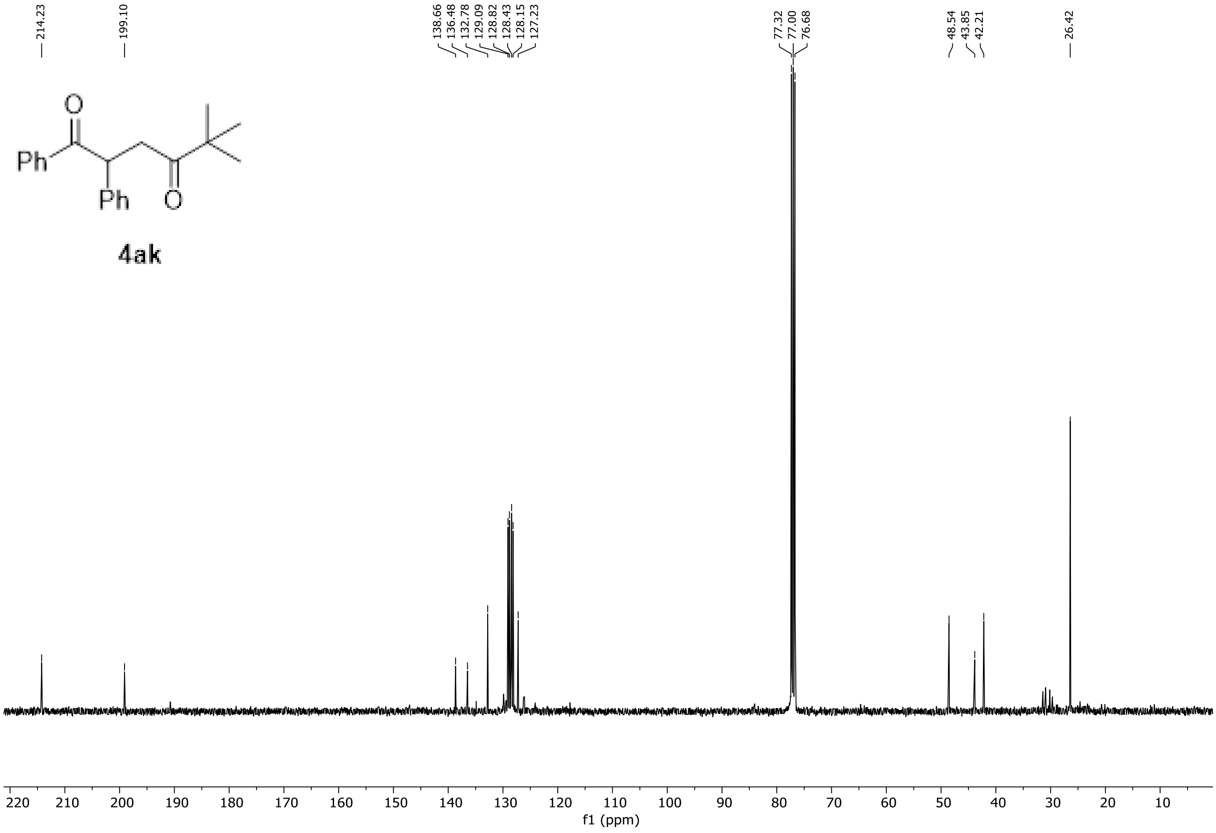

4al ^{1}H NMR (300 MHz, $CDCl_3$)

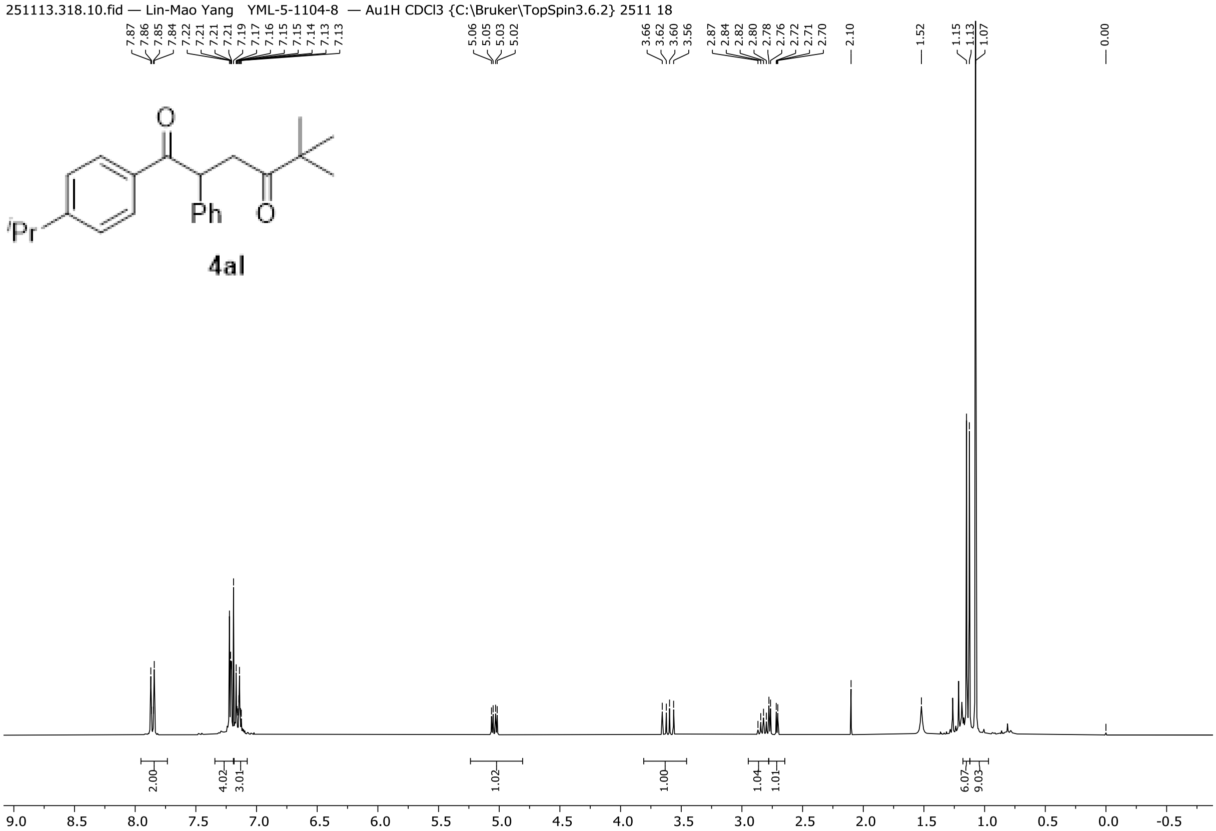

4al ^{13}C NMR (75 MHz, $CDCl_3$)

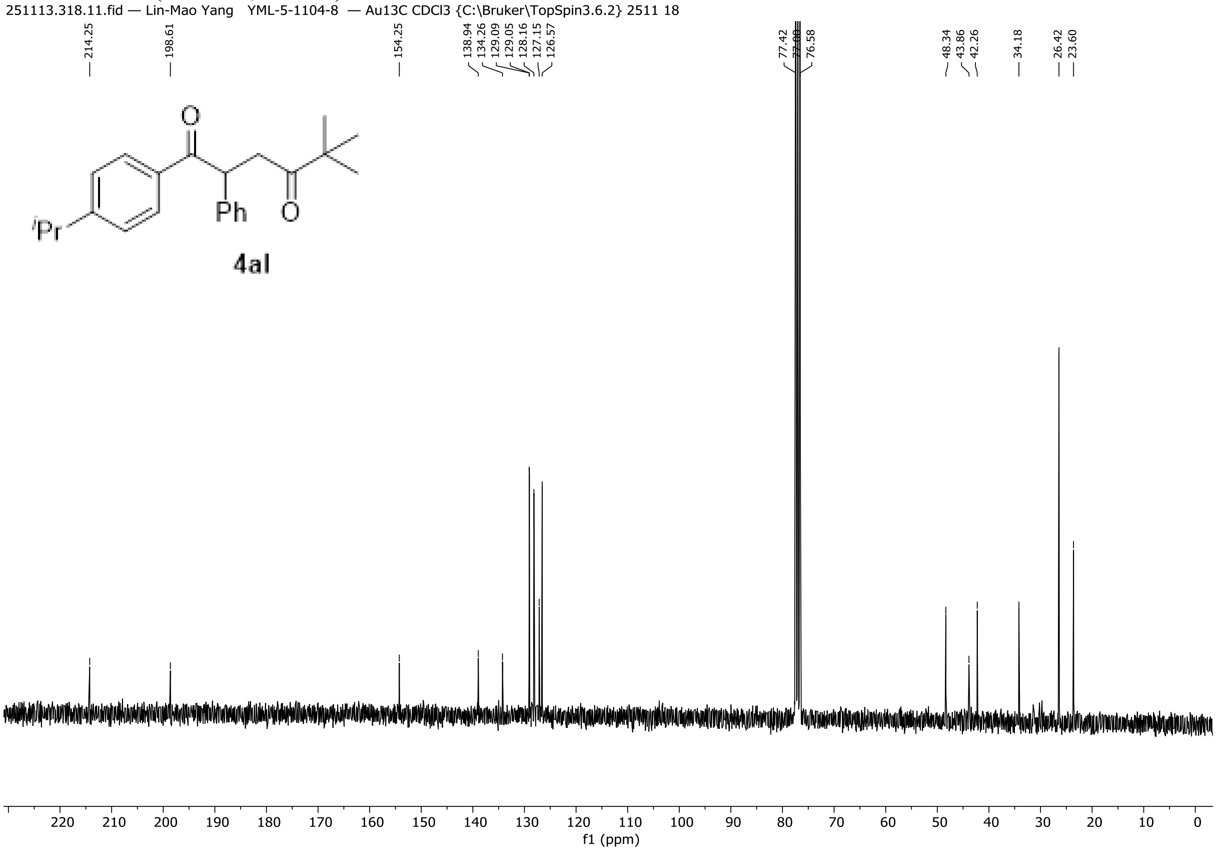

4am ^{1}H NMR (300 MHz, $CDCl_3$)

251107.318.10.fid — Mao-Lin Yang YML-5-1104-5-1 — Au1H CDCl3 {C:\Bruker\TopSpin3.6.2} 2511 18

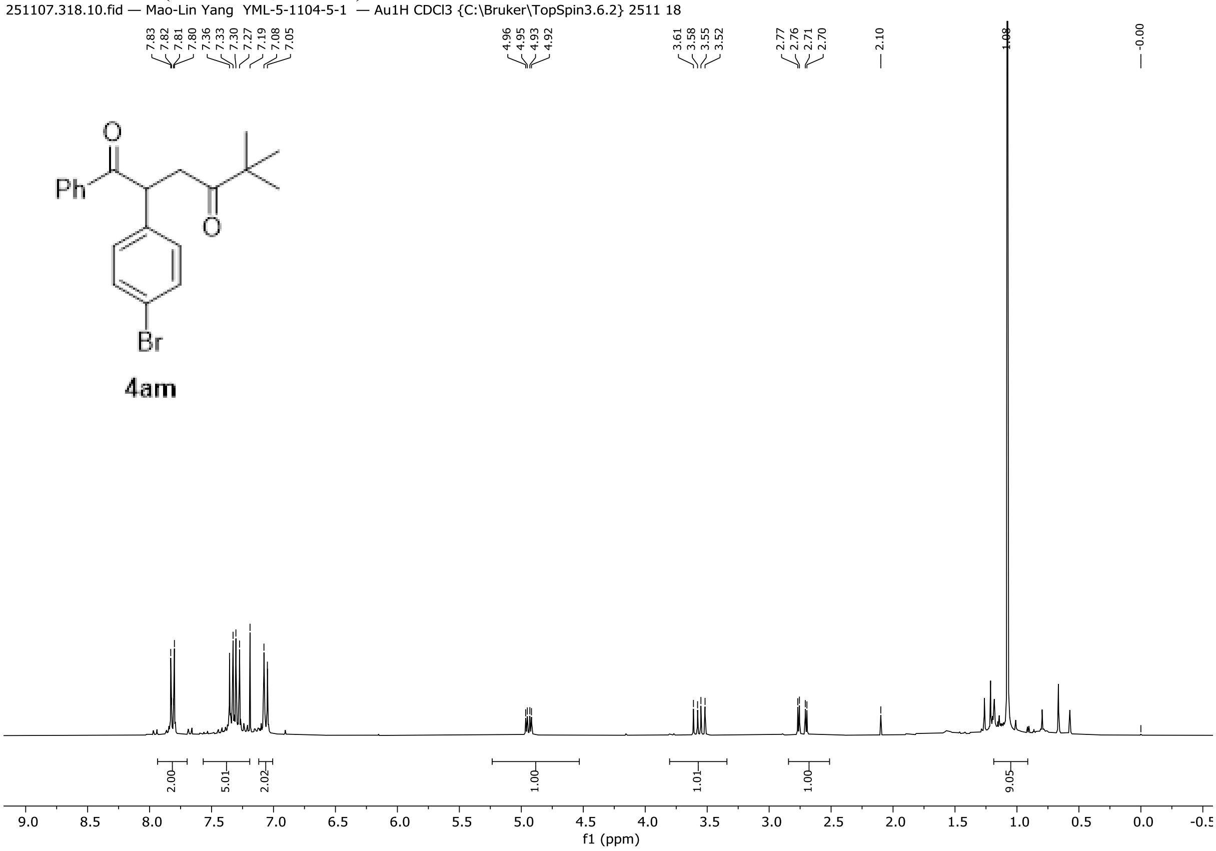

4am ^{13}C NMR (75 MHz, $CDCl_3$)

251107.318.11.fid — Mao-Lin Yang YML-5-1104-5-1 — Au13C CDCl3 {C:\Bruker\TopSpin3.6.2} 2511 18

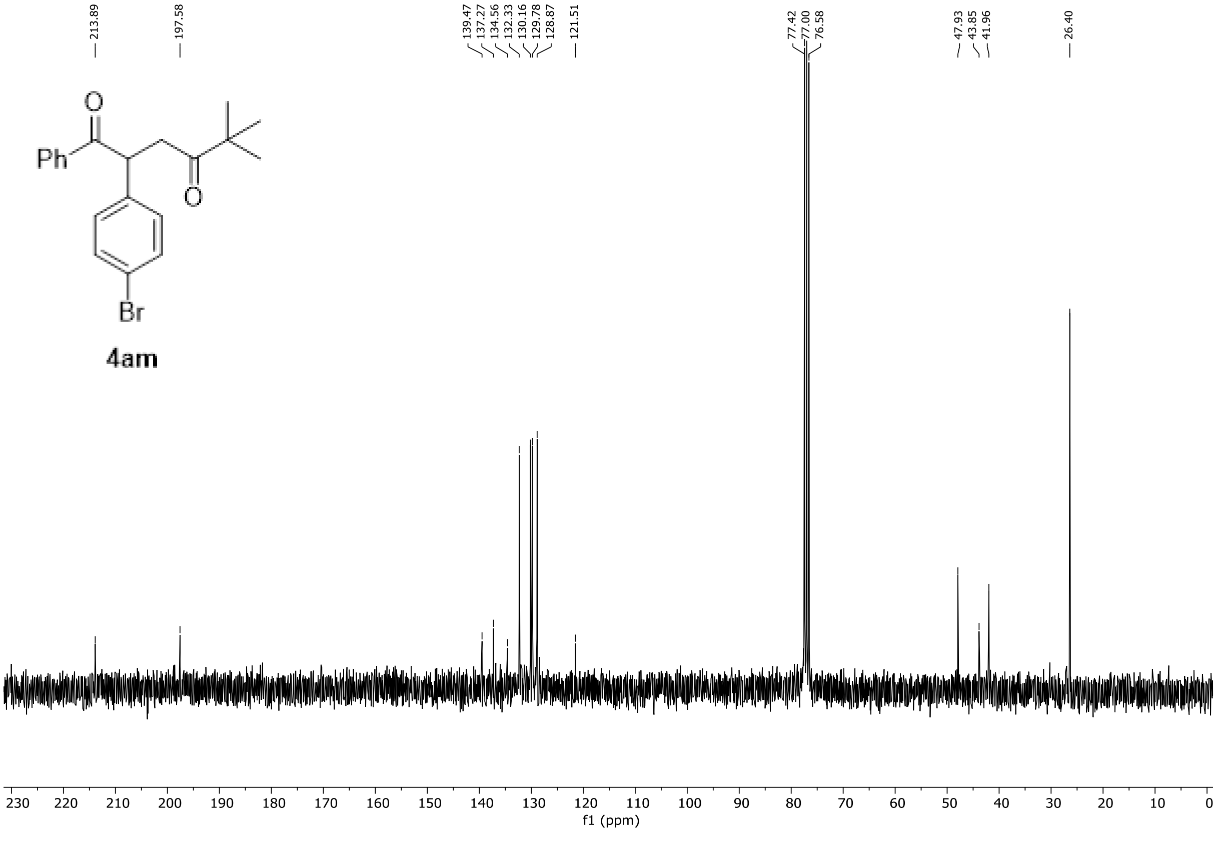

4an ^{1}H NMR (300 MHz, $CDCl_3$)

251110.318.10.fid — Mao-Lin Yang, YML-5-1104-9 — Au1H CDCl3 {C:\Bruker\TopSpin3.6.2} 2511 18

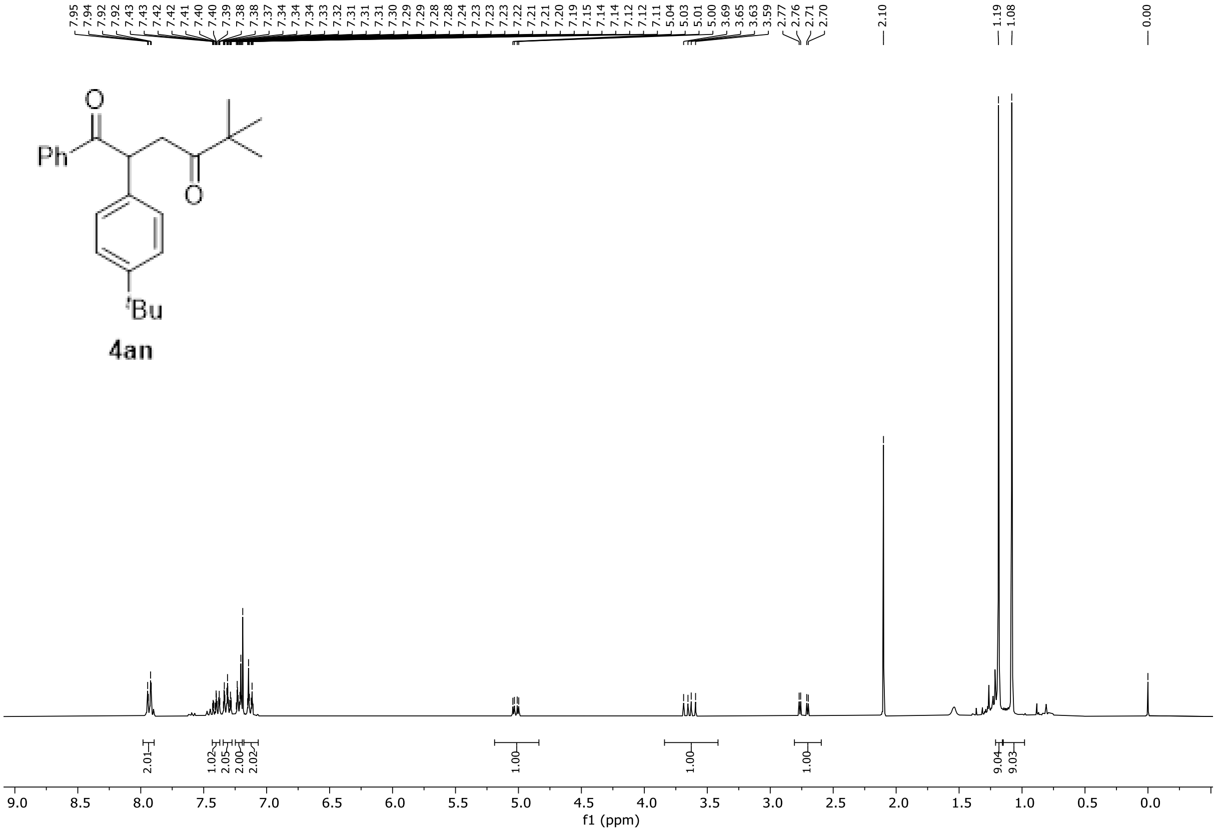

4an ^{13}C NMR (75 MHz, $CDCl_3$)

251110.318.11.fid — Mao-Lin Yang, YML-5-1104-9 — Au13C CDCl3 {C:\Bruker\TopSpin3.6.2} 2511 18

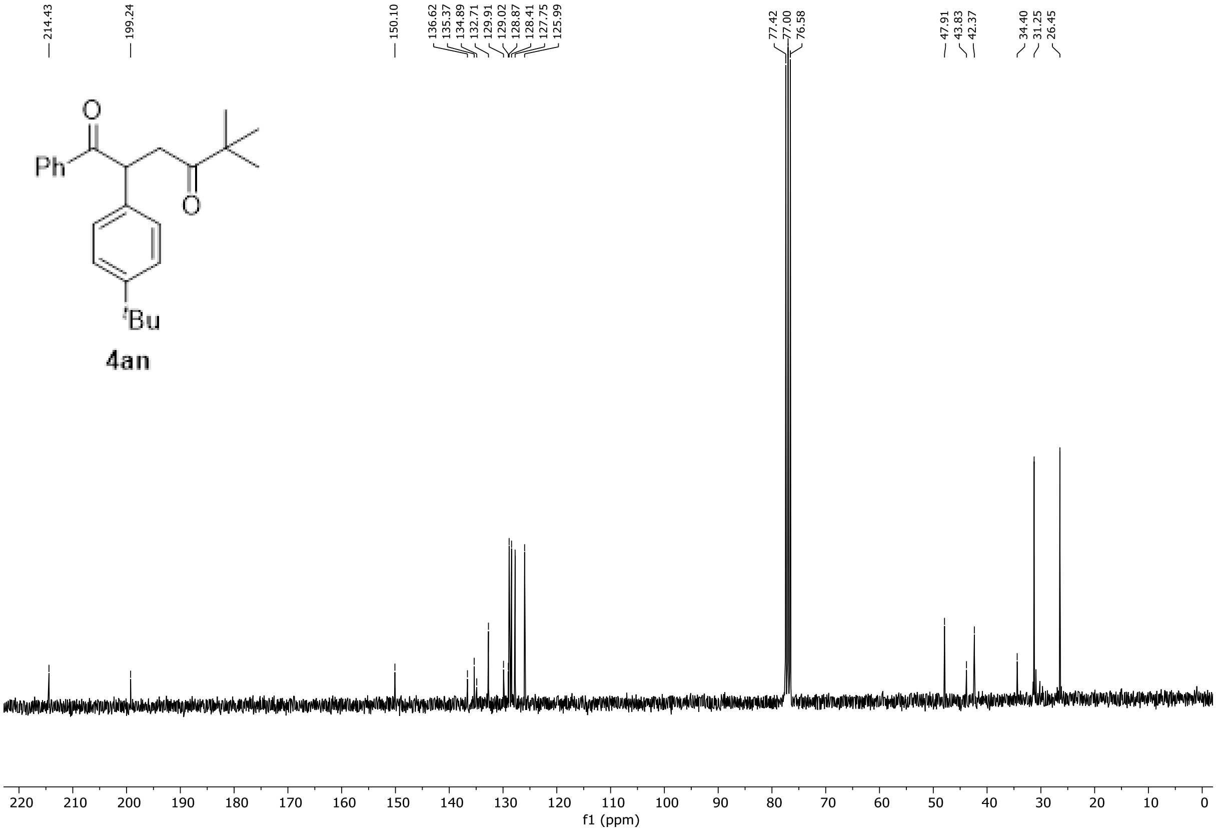

4ao ^{1}H NMR (300 MHz, $CDCl_3$)

251117.306.10.fid — Mao-Lin Yang YML-5-1112-7 — Au1H CDCl3 {C:\Bruker\TopSpin3.6.2} 2511 6

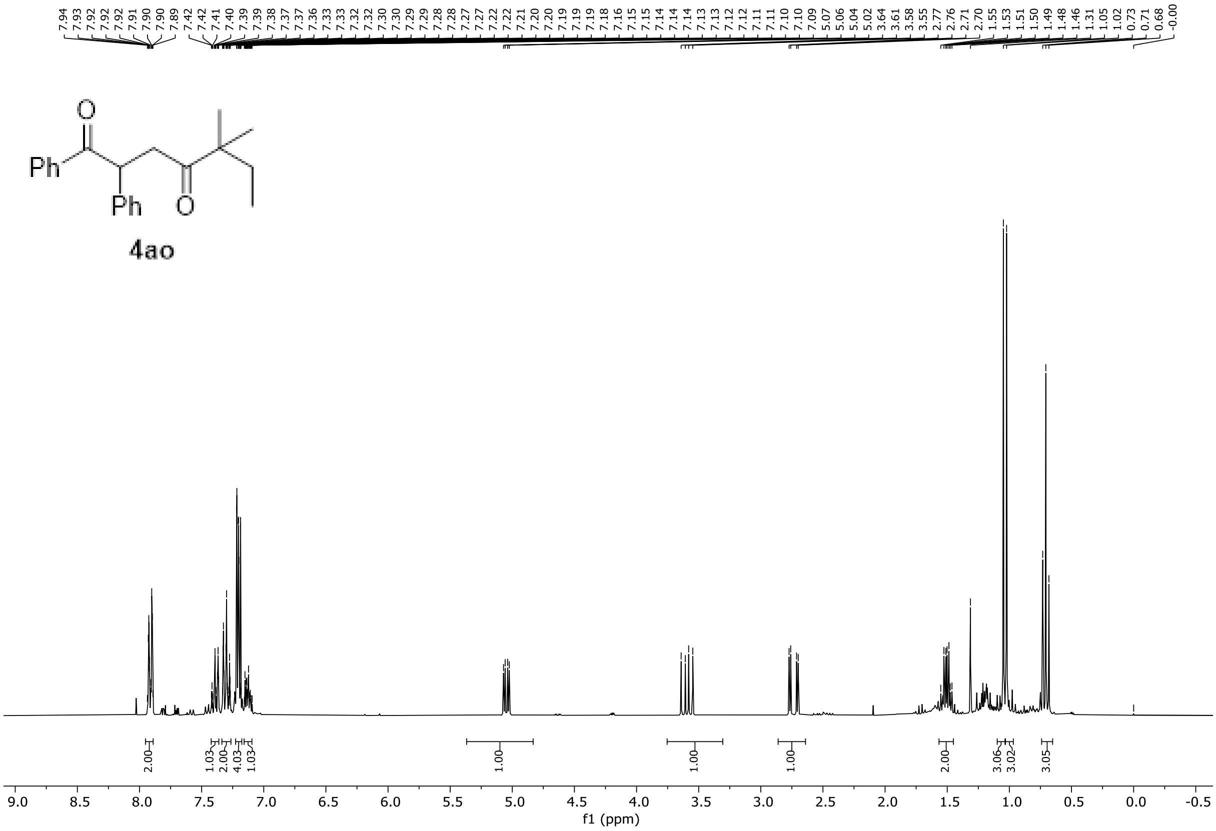

4ao ^{13}C NMR (75 MHz, $CDCl_3$)

251117.306.11.fid — Mao-Lin Yang YML-5-1112-7 — Au13C CDCl3 {C:\Bruker\TopSpin3.6.2} 2511 6

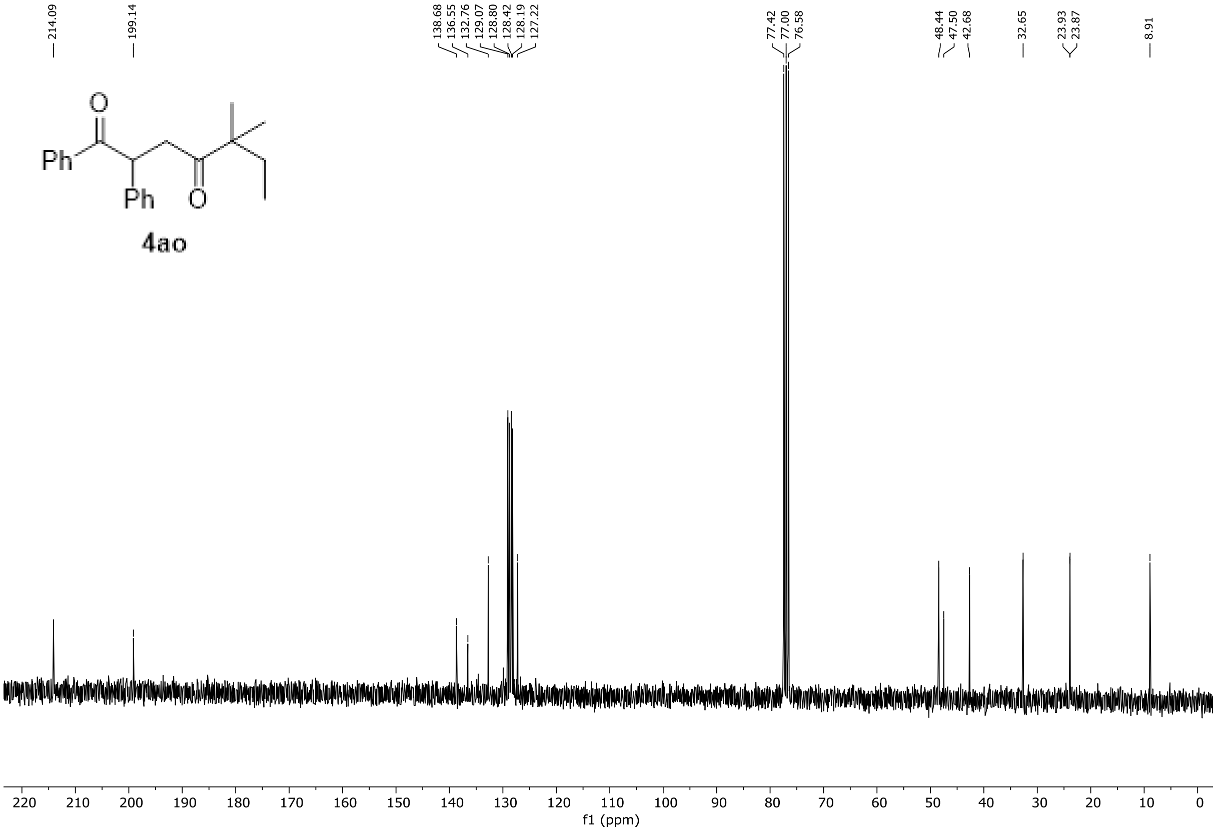

4ap ^{1}H NMR (300 MHz, $CDCl_3$)

251120.329.10.fid — Mao-Lin Yang, YML-5-1116-1 — Au1H CDCl3 {C:\Bruker\TopSpin3.6.2} 2511 29

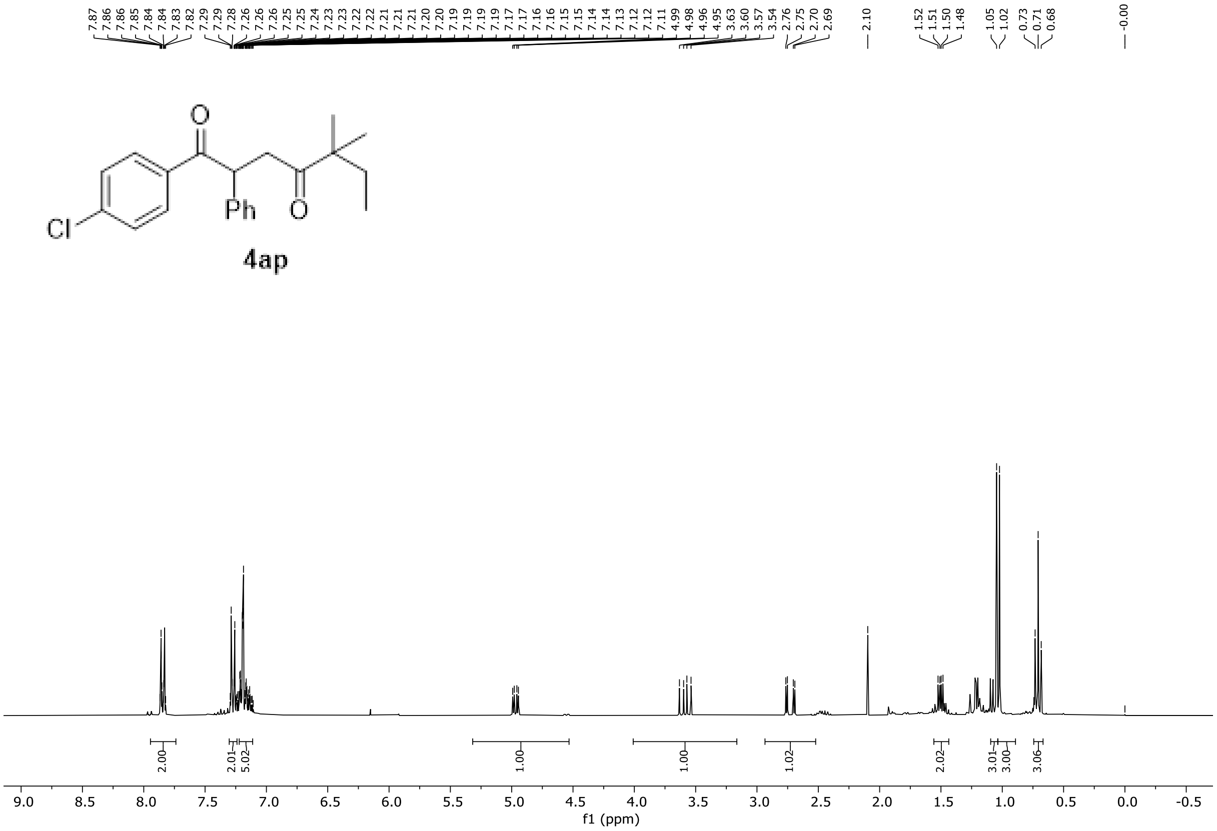

4ap ^{13}C NMR (75 MHz, $CDCl_3$)

251120.329.11.fid — Mao-Lin Yang, YML-5-1116-1 — Au13C CDCl3 {C:\Bruker\TopSpin3.6.2} 2511 29

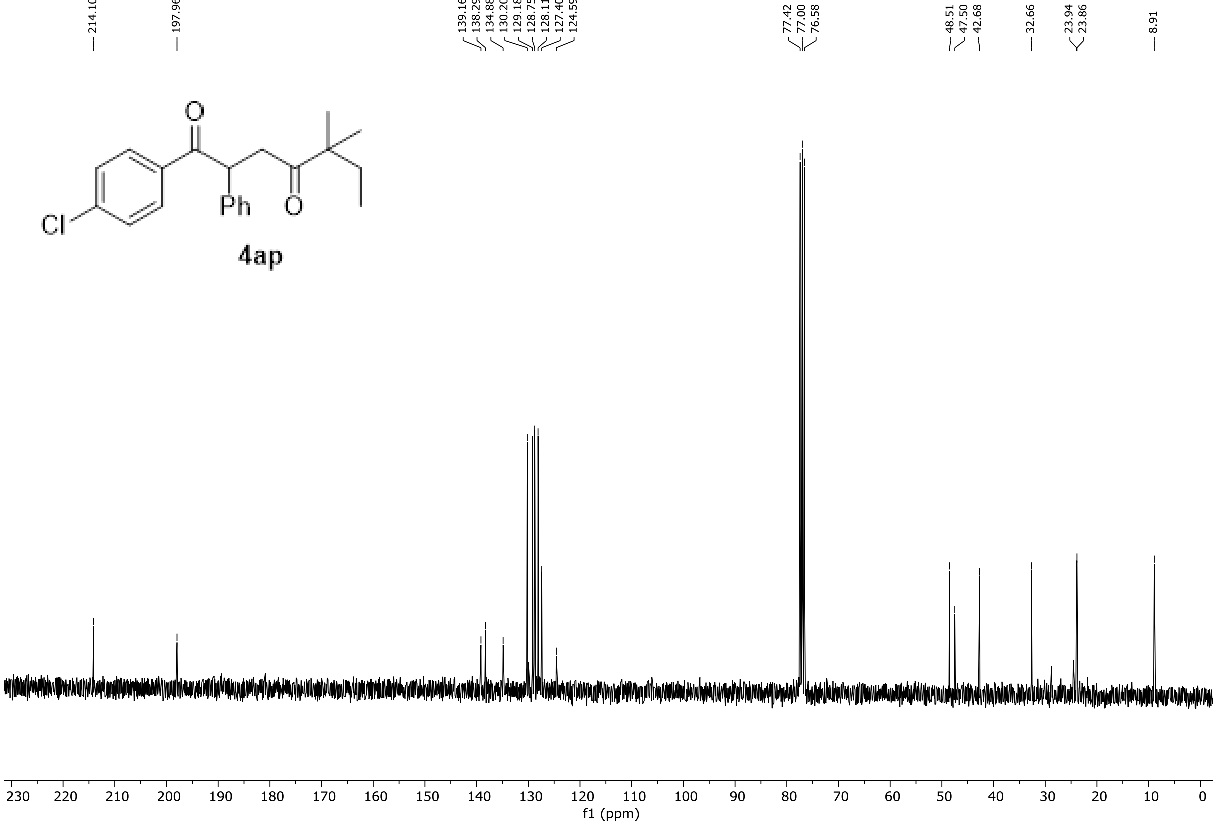

4aq ^{1}H NMR (300 MHz, $CDCl_3$)

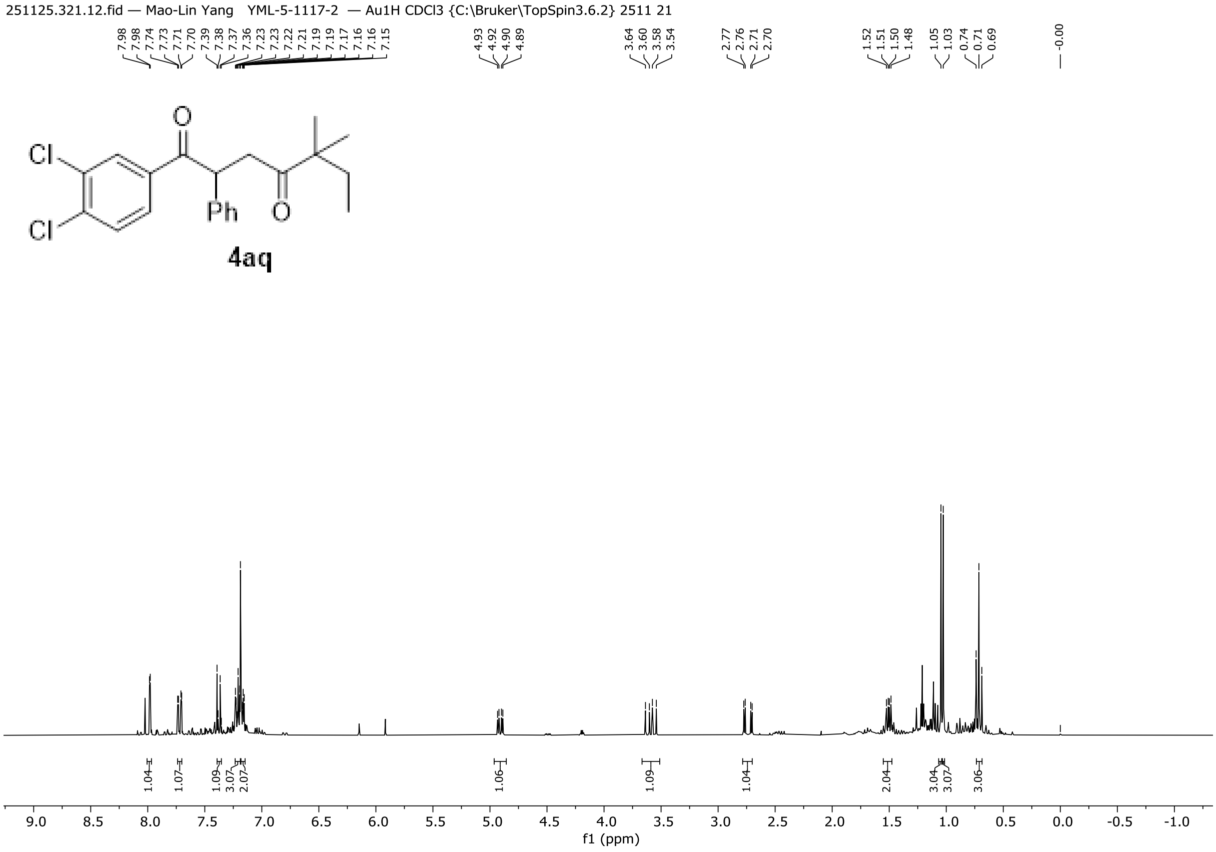

4aq ^{13}C NMR (75 MHz, $CDCl_3$)

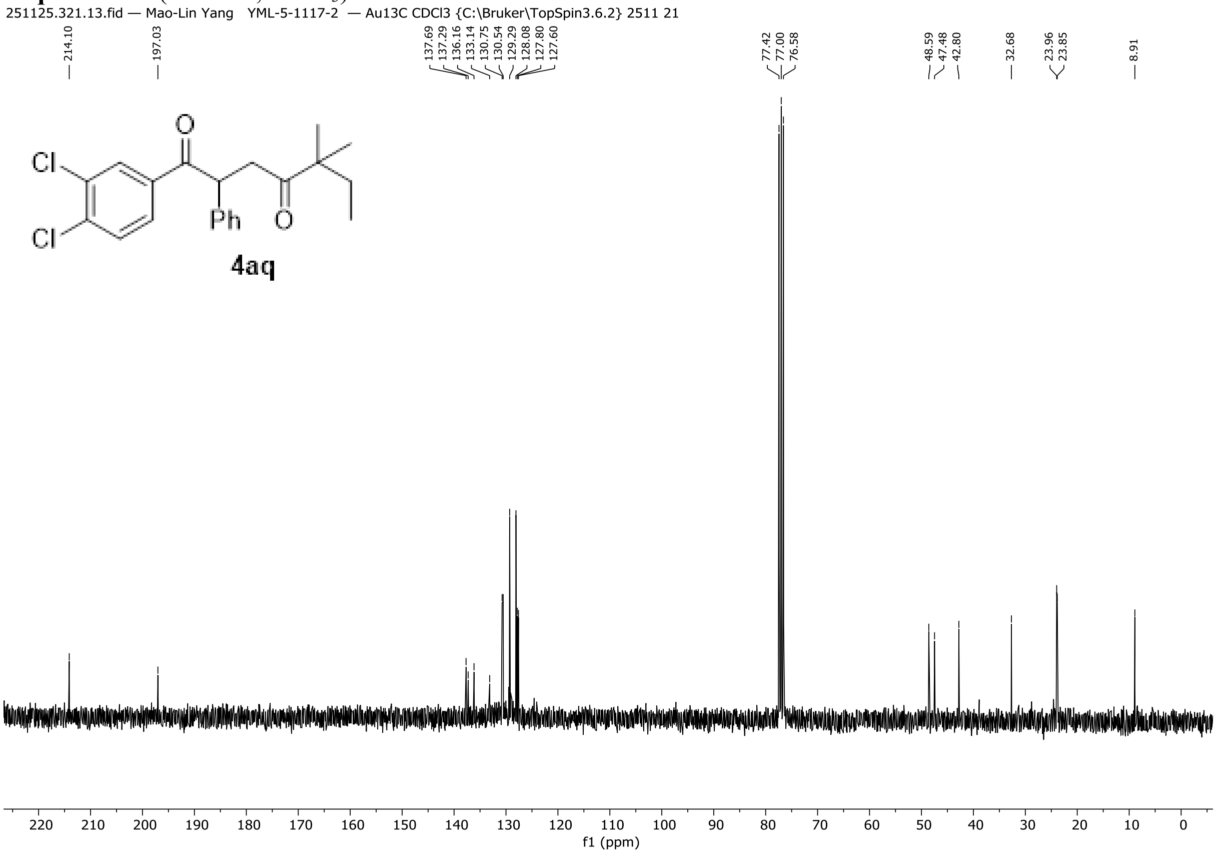

4ar ^{1}H NMR (300 MHz, $CDCl_3$)

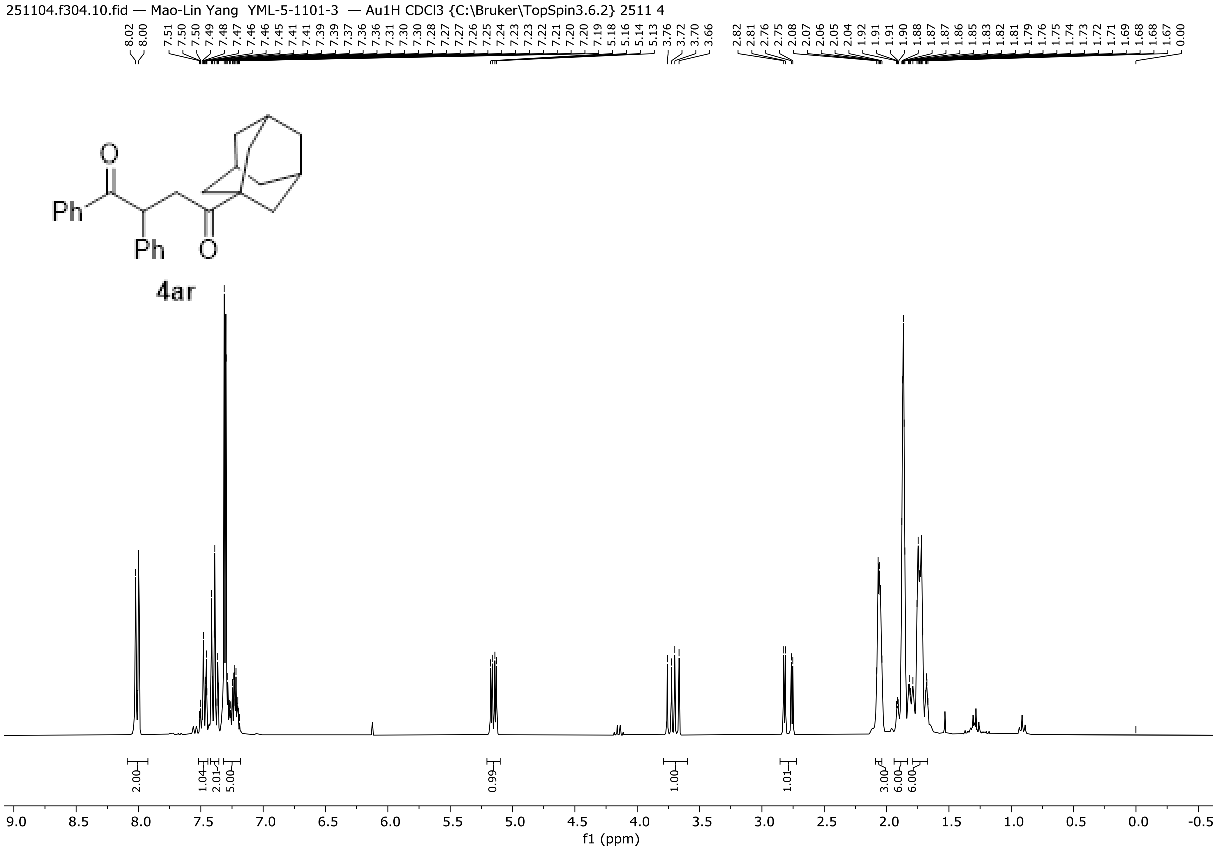

4ar ^{13}C NMR (75 MHz, $CDCl_3$)

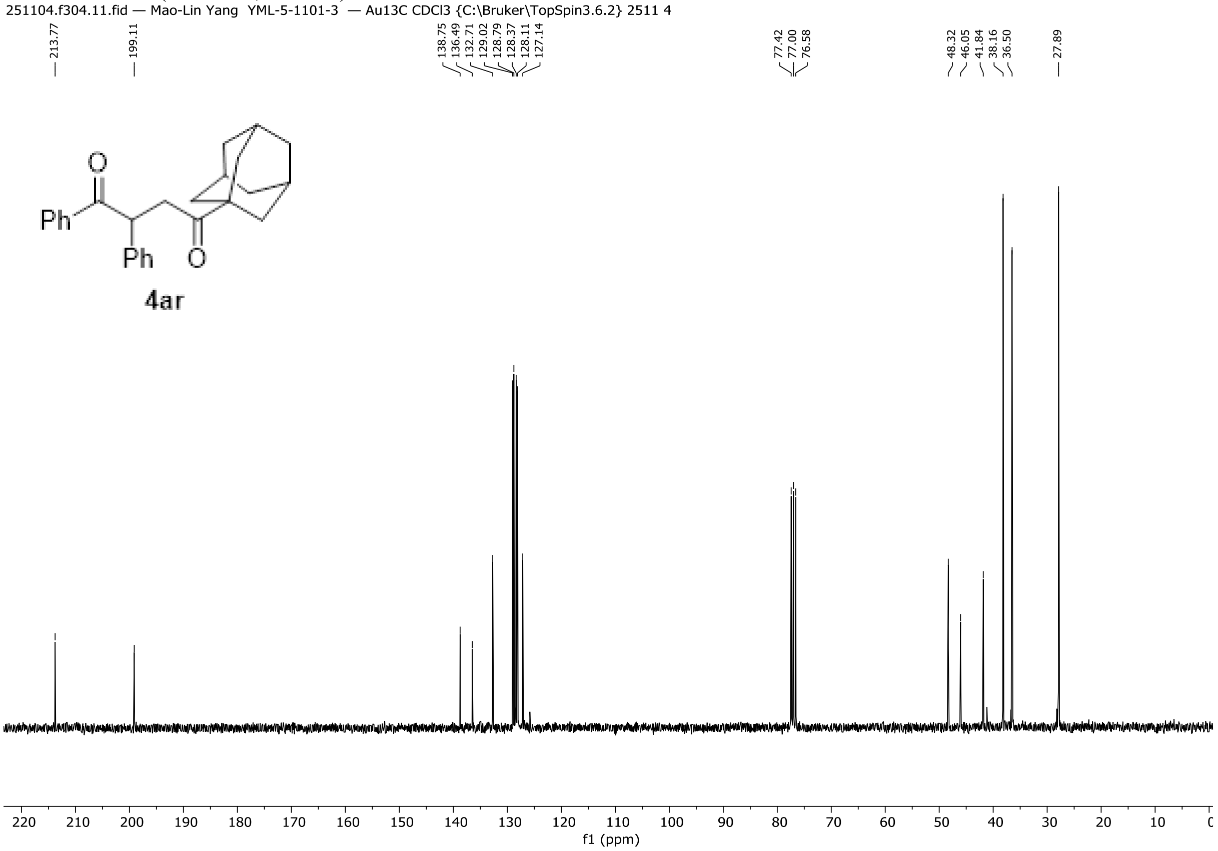

4as ^{1}H NMR (300 MHz, $CDCl_3$)

251103.313.10.fid — Mao-Lin Yang YML-5-1030-2 — Au1H CDCl3 {C:\Bruker\TopSpin3.6.2} 2511 13

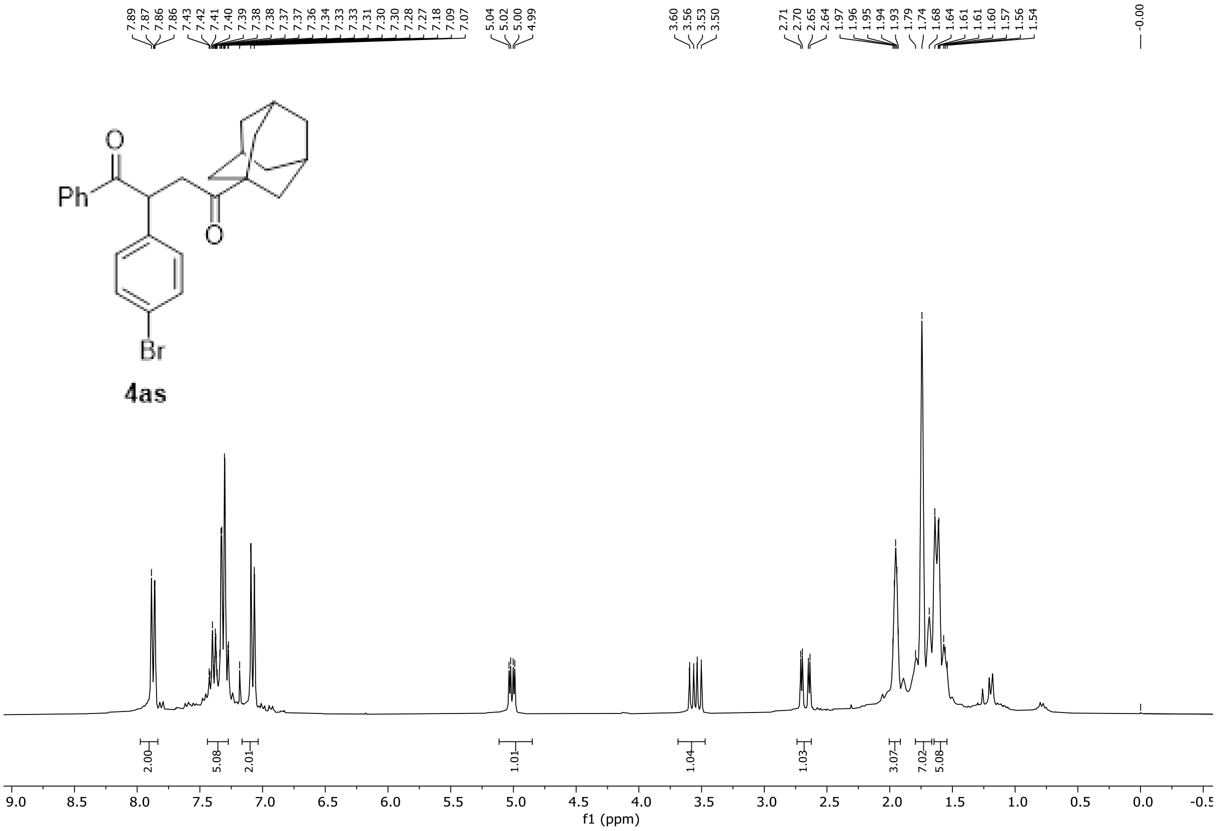

4as ^{13}C NMR (75 MHz, $CDCl_3$)

251103.313.11.fid — Mao-Lin Yang YML-5-1030-2 — Au13C CDCl3 {C:\Bruker\TopSpin3.6.2} 2511 13

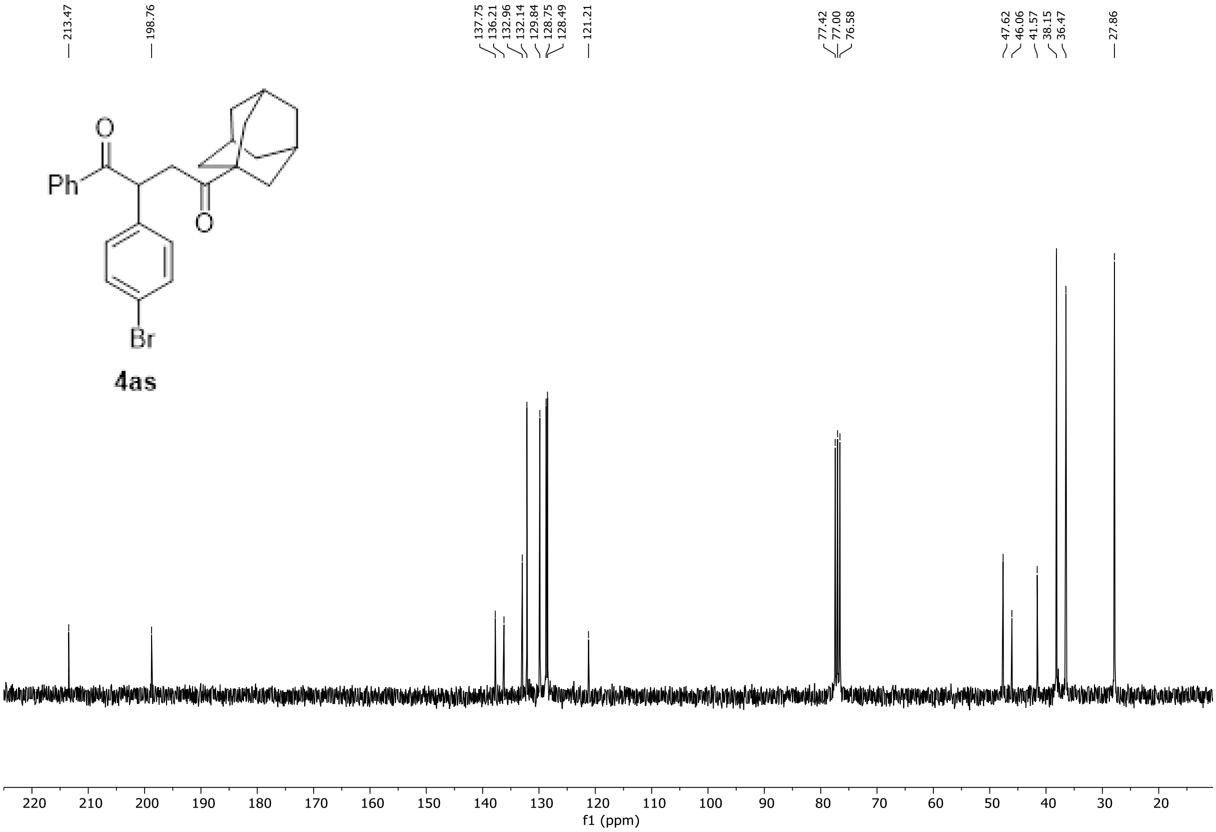

4at ^{1}H NMR (300 MHz, $CDCl_3$)

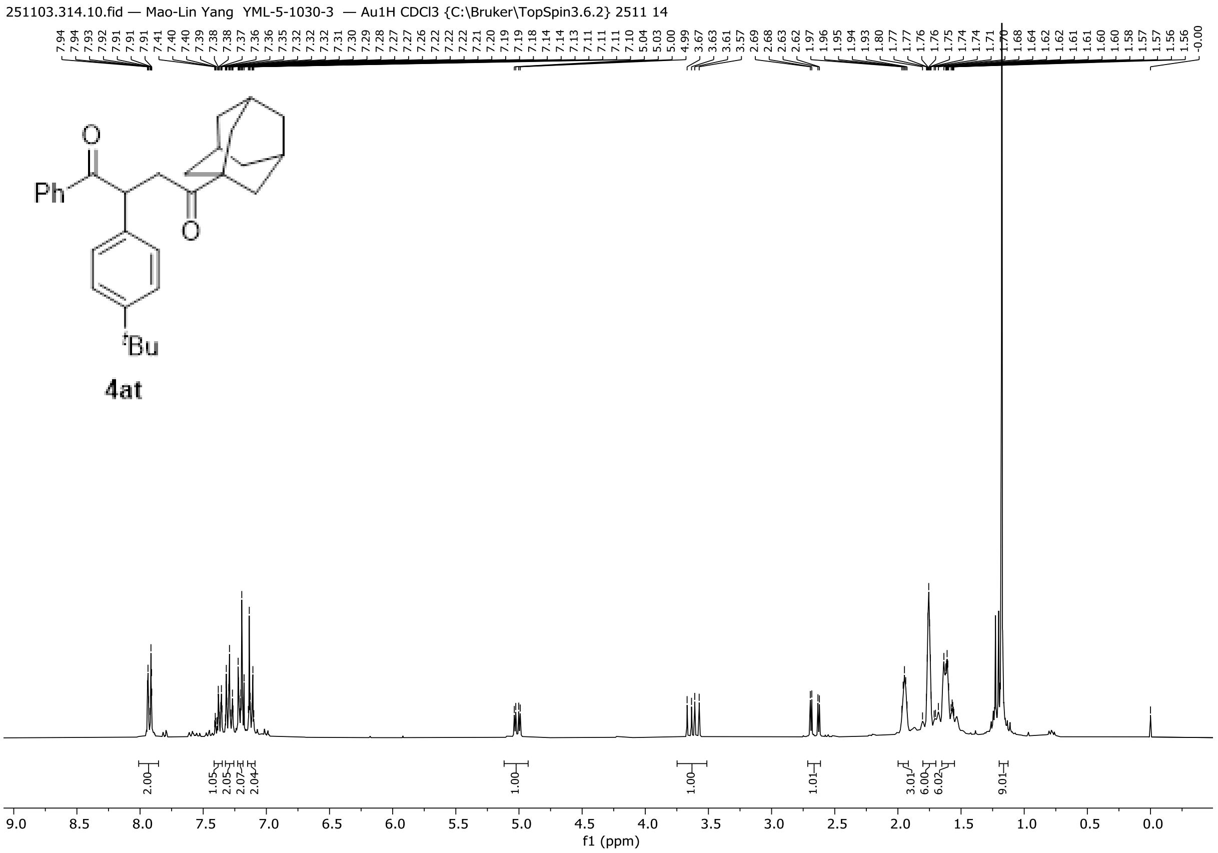

4at ^{13}C NMR (75 MHz, $CDCl_3$)

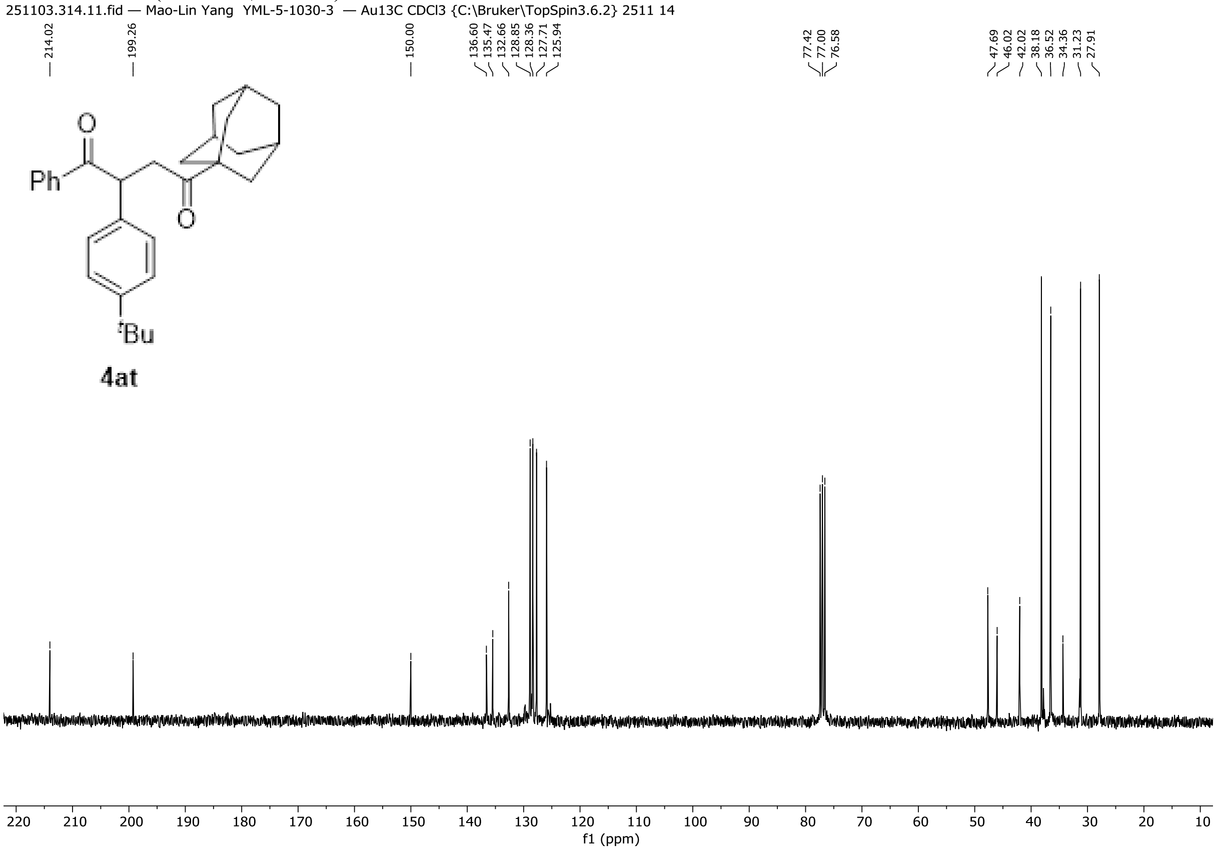

4au ^{1}H NMR (300 MHz, $CDCl_3$)

251103.315.10.fid — Mao-Lin Yang YML-5-1030-4 — Au1H CDCl3 {C:\Bruker\TopSpin3.6.2} 2511 15

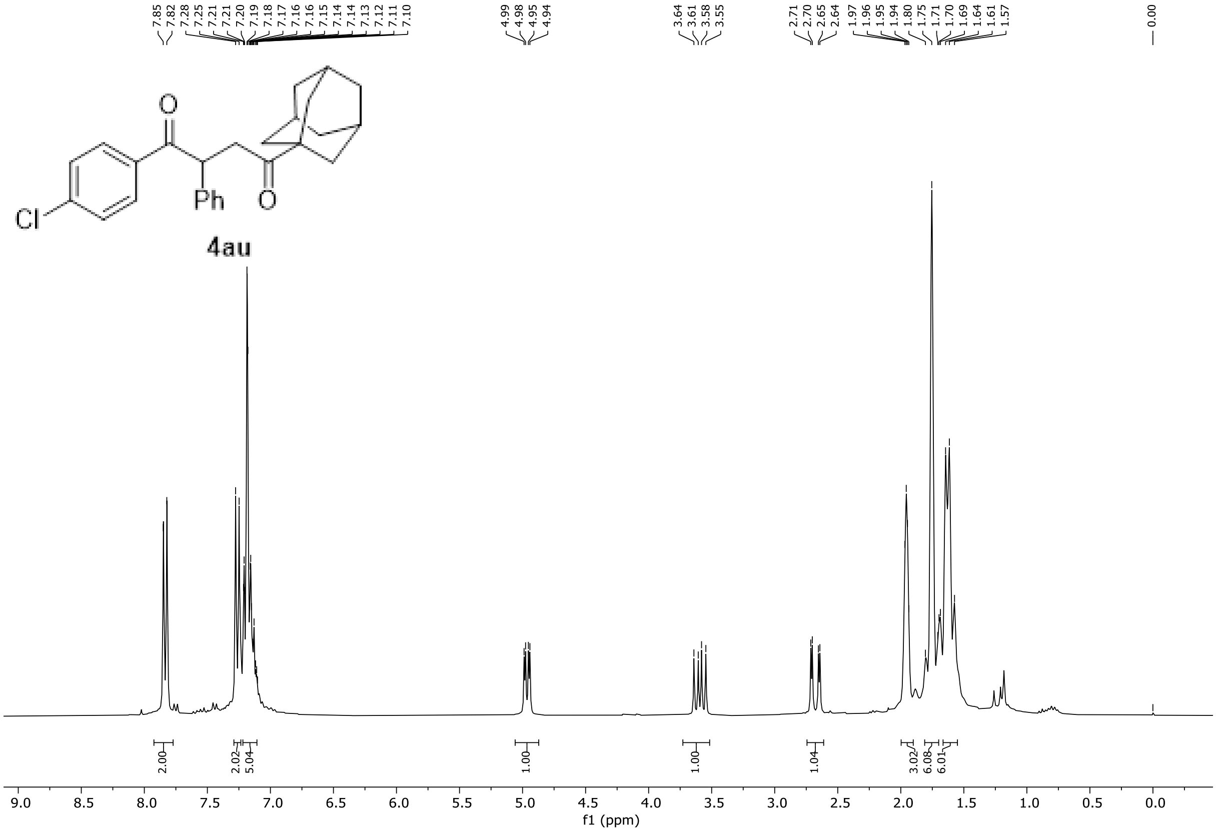

4au ^{13}C NMR (75 MHz, $CDCl_3$)

251103.315.11.fid — Mao-Lin Yang YML-5-1030-4 — Au13C CDCl3 {C:\Bruker\TopSpin3.6.2} 2511 15

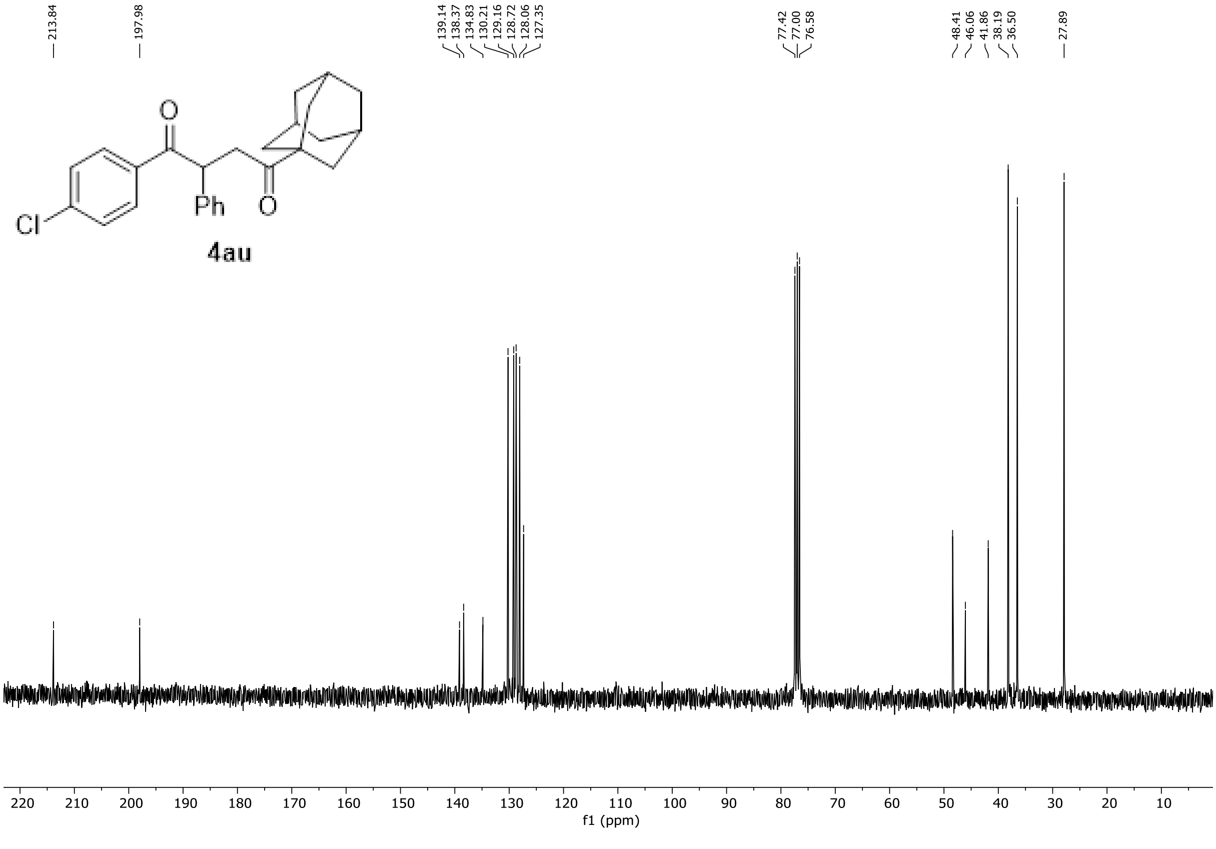

4av ^{1}H NMR (300 MHz, $CDCl_3$)

251103.316.10.fid — Mao-Lin Yang YML-5-1030-5 — Au1H CDCl3 {C:\Bruker\TopSpin3.6.2} 2511 16

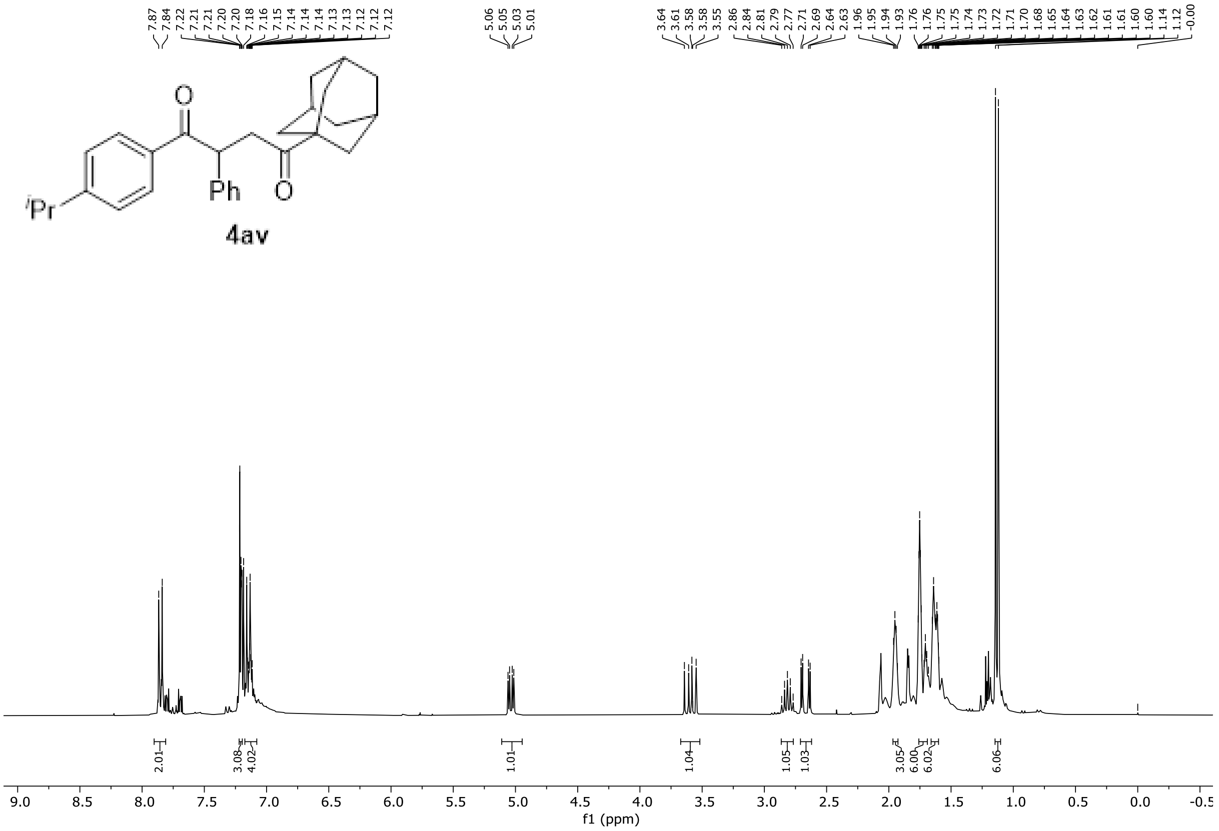

4av ^{13}C NMR (75 MHz, $CDCl_3$)

251103.316.11.fid — Mao-Lin Yang YML-5-1030-5 — Au13C CDCl3 {C:\Bruker\TopSpin3.6.2} 2511 16

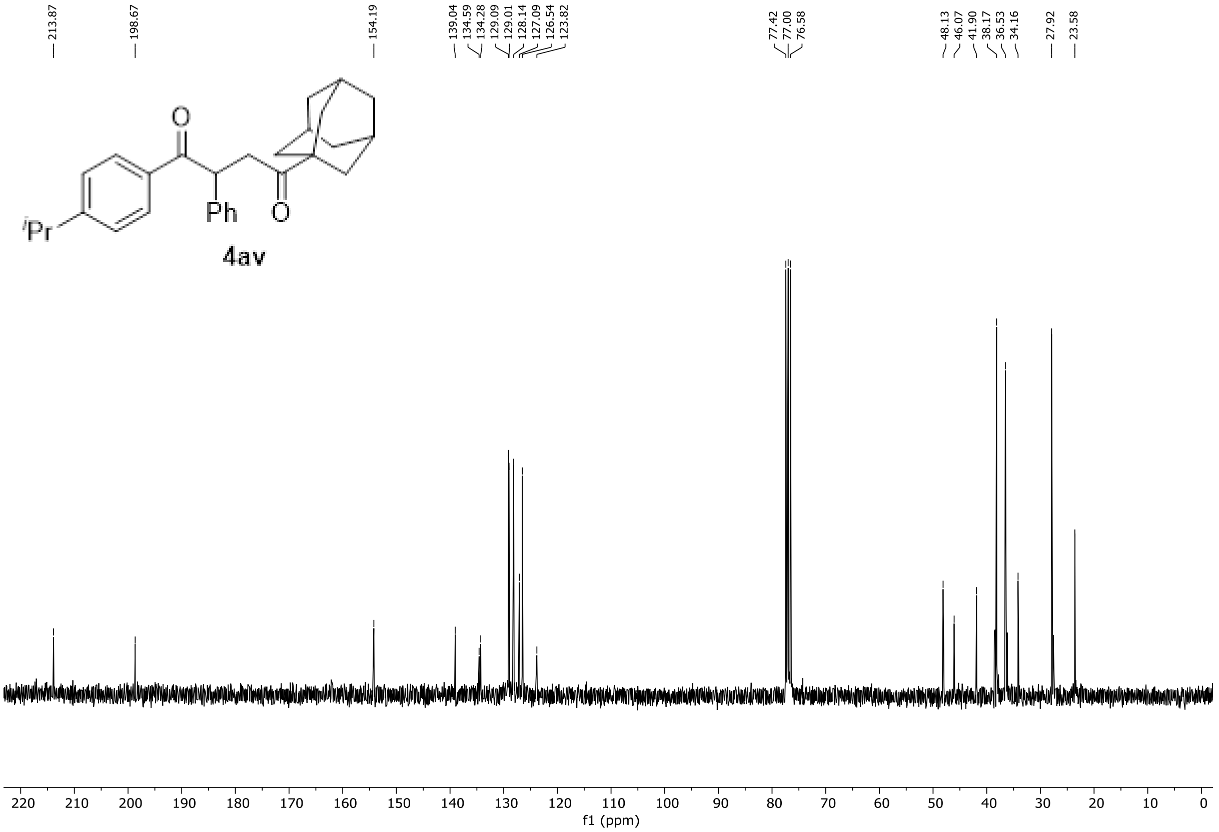

4aw ^{1}H NMR (300 MHz, $CDCl_3$)

251117.305.10.fid — Mao-Lin Yang YML-5-1112-5 — Au1H CDCl3 {C:\Bruker\TopSpin3.6.2} 2511 5

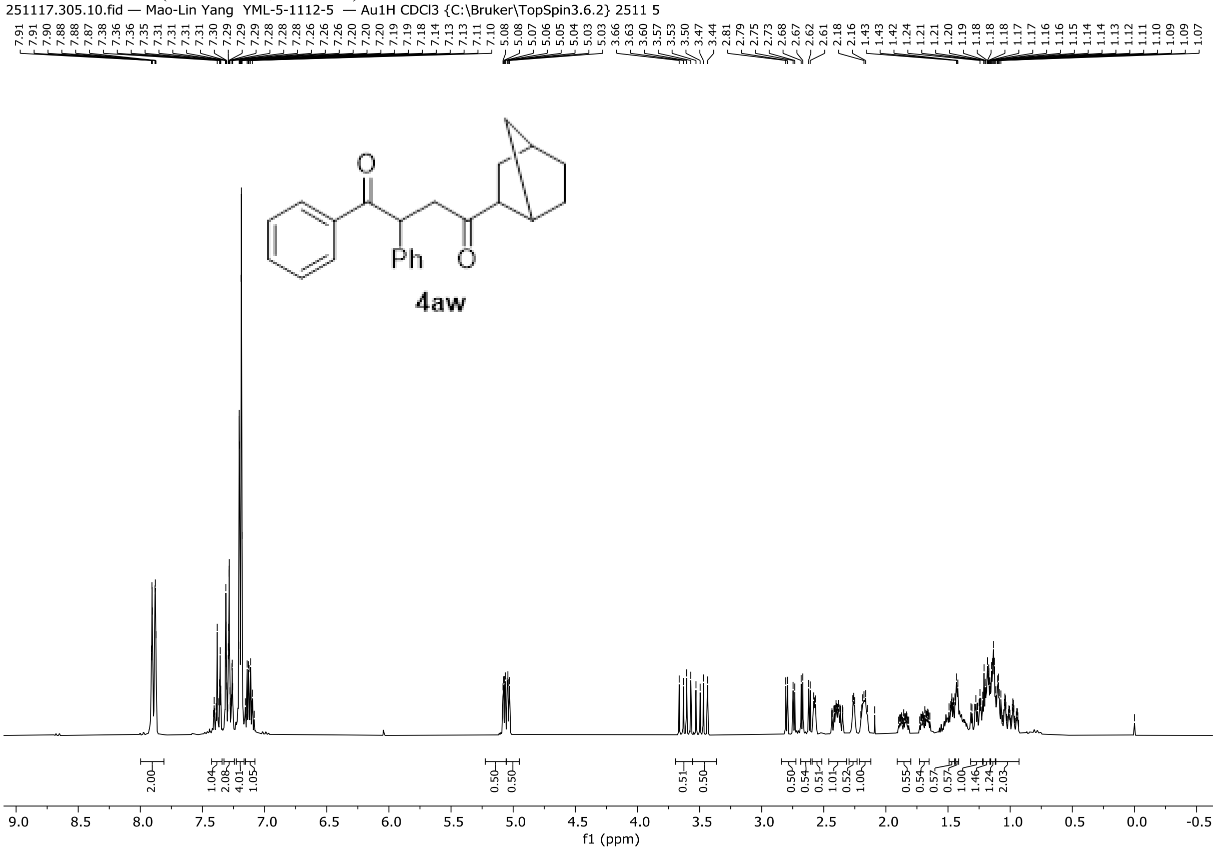

4aw ^{13}C NMR (75 MHz, $CDCl_3$)

251117.305.11.fid — Mao-Lin Yang YML-5-1112-5 — Au13C CDCl3 {C:\Bruker\TopSpin3.6.2} 2511 5

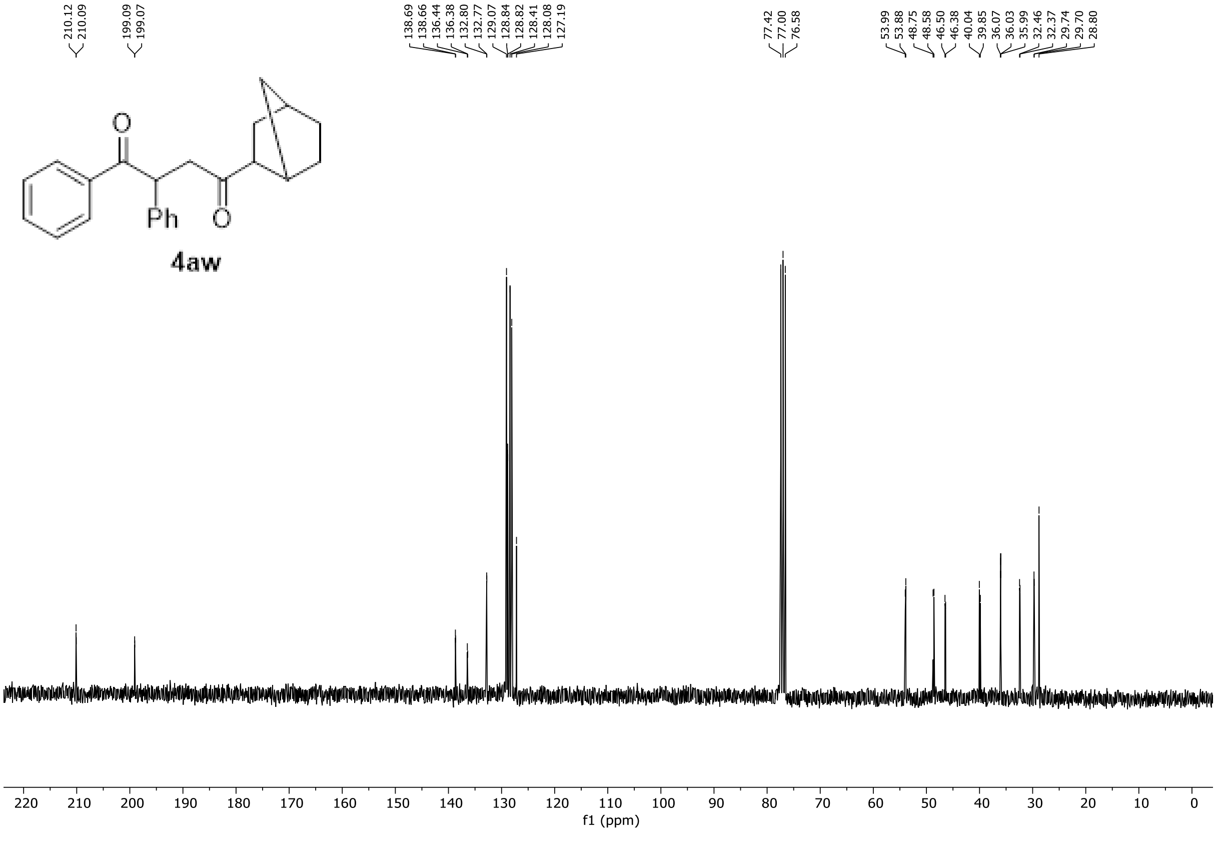

4ax ^{1}H NMR (300 MHz, $CDCl_3$)

251117.307.10.fid — Mao-Lin Yang YML-5-1112-9 — Au1H CDCl3 {C:\Bruker\TopSpin3.6.2} 2511 7

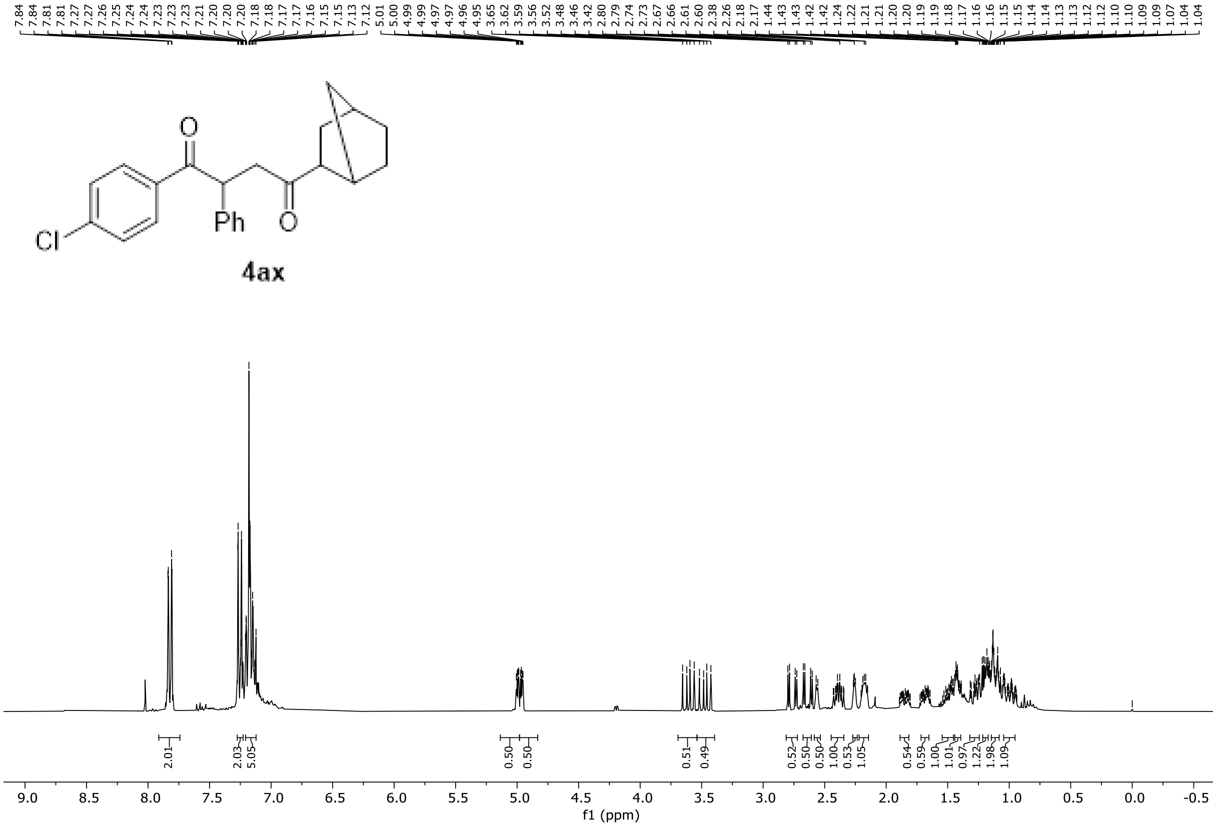

4ax ^{13}C NMR (75 MHz, $CDCl_3$)

251117.307.11.fid — Mao-Lin Yang YML-5-1112-9 — Au13C CDCl3 {C:\Bruker\TopSpin3.6.2} 2511 7

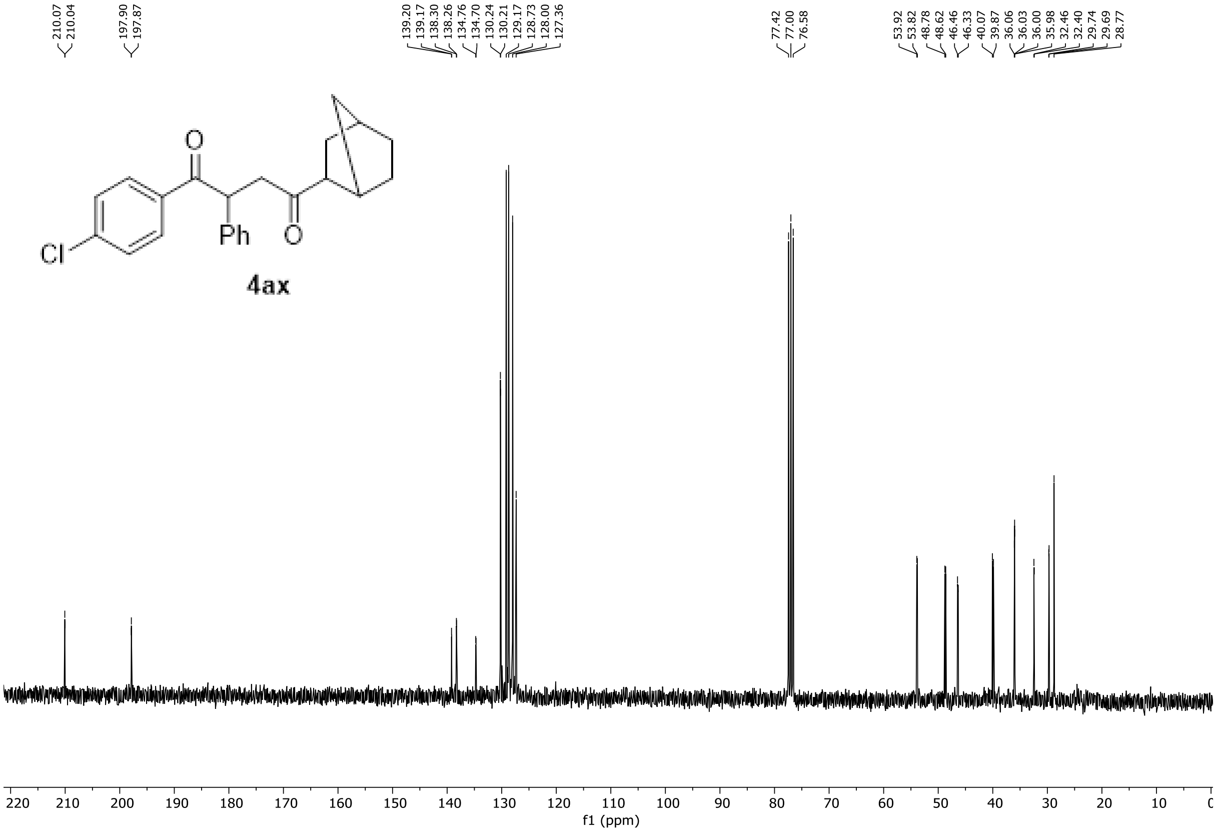

8a ^{1}H NMR (300 MHz, $CDCl_3$)

251207.335.10.fid — Mao-Lin yang, YML-5-cy-y-1 — Au1H CDCl3 {C:\Bruker\TopSpin3.6.2} 2512 35

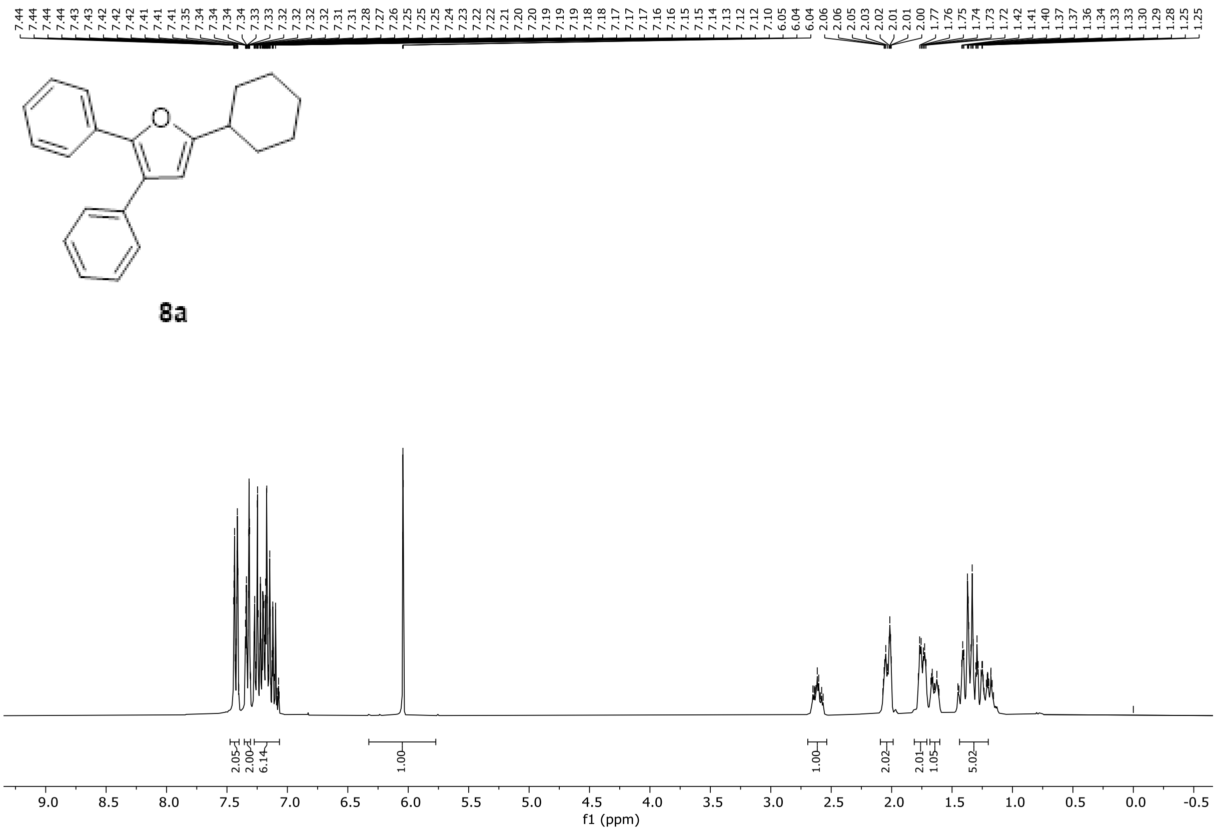

8a ^{13}C NMR (75 MHz, $CDCl_3$)

251207.335.11.fid — Mao-Lin yang, YML-5-cy-y-1 — Au13C CDCl3 {C:\Bruker\TopSpin3.6.2} 2512 35

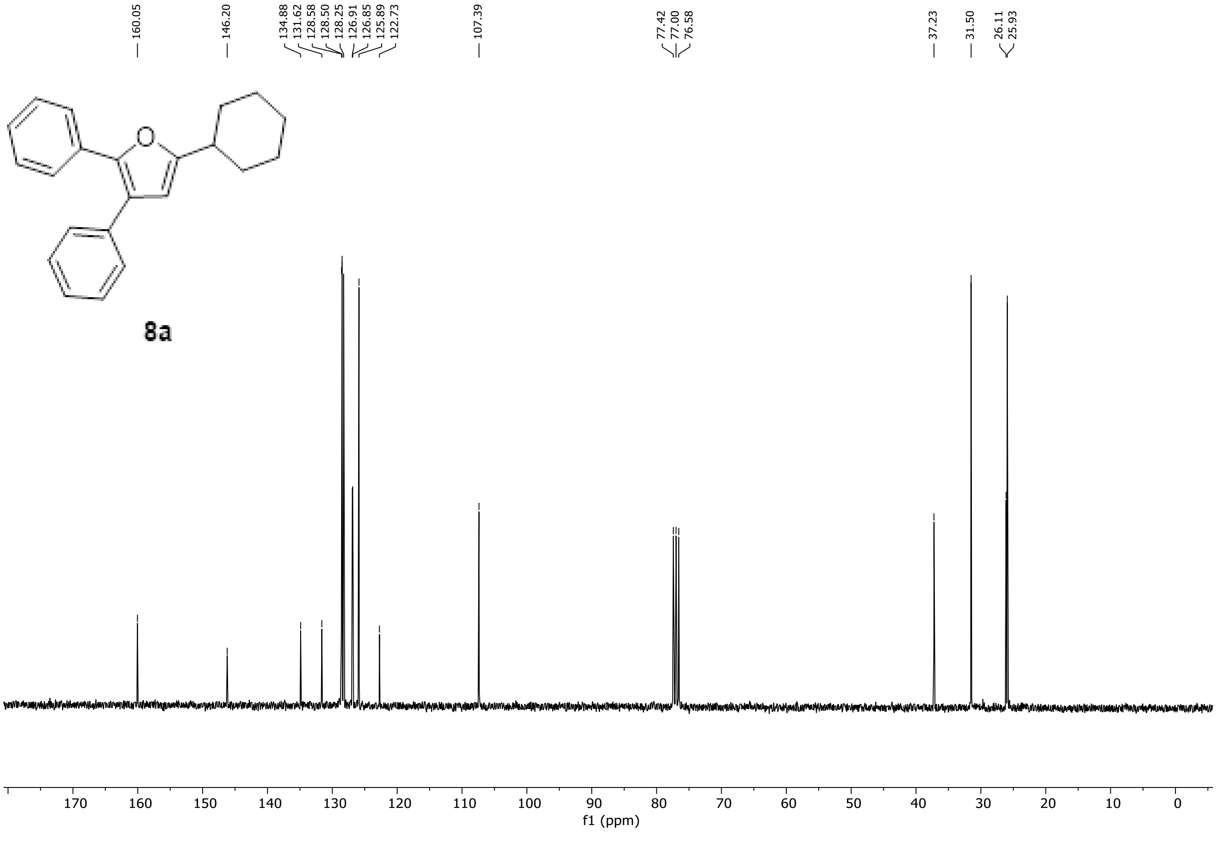

8a‘ ^{1}H NMR (300 MHz, $CDCl_3$)

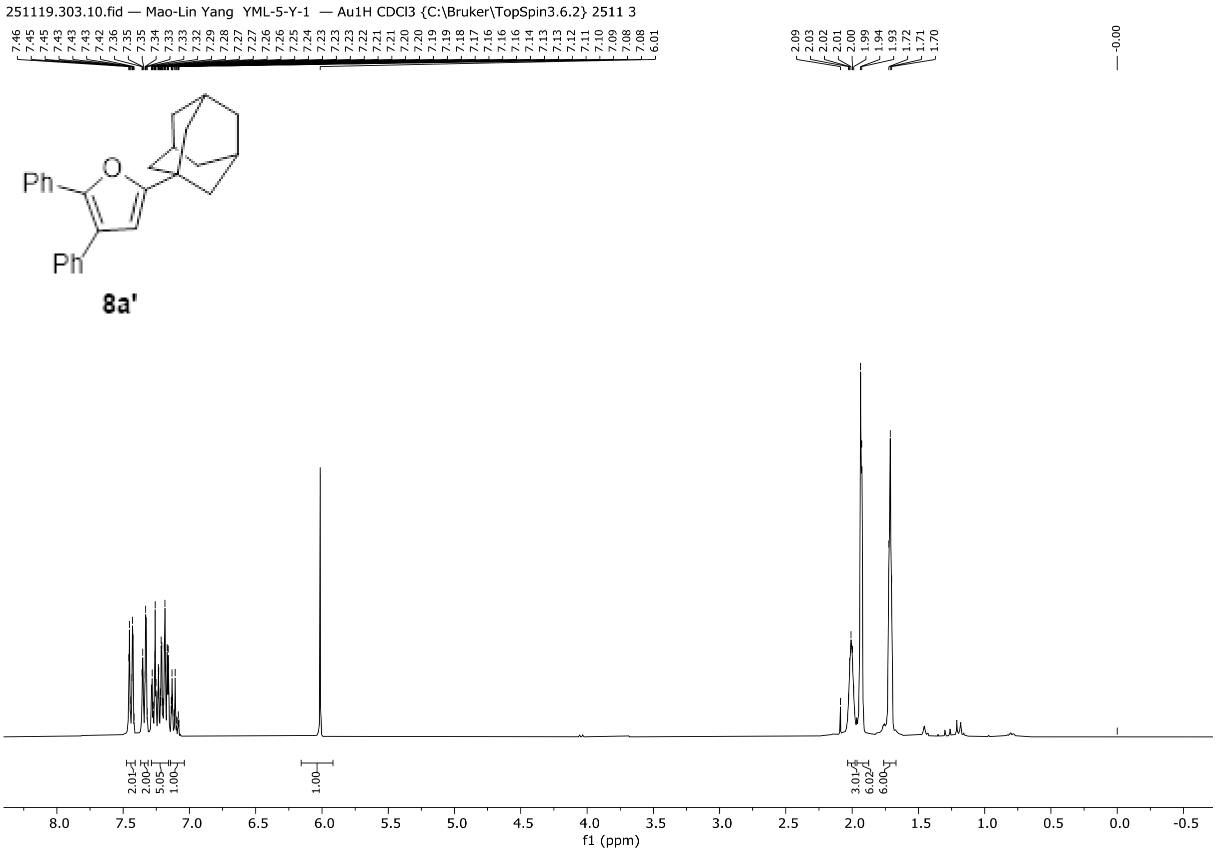

8a‘ ^{13}C NMR (75 MHz, $CDCl_3$)

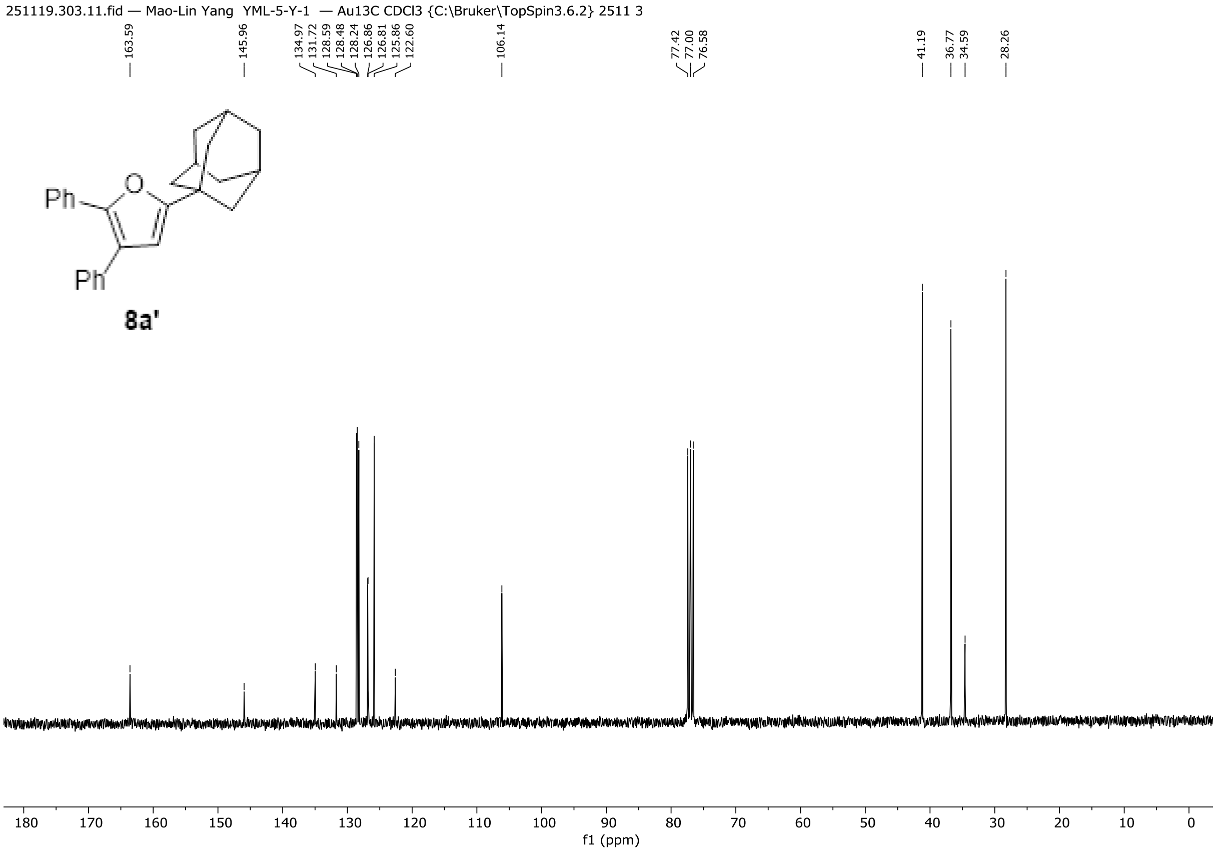

8b ^{1}H NMR (300 MHz, $CDCl_3$)

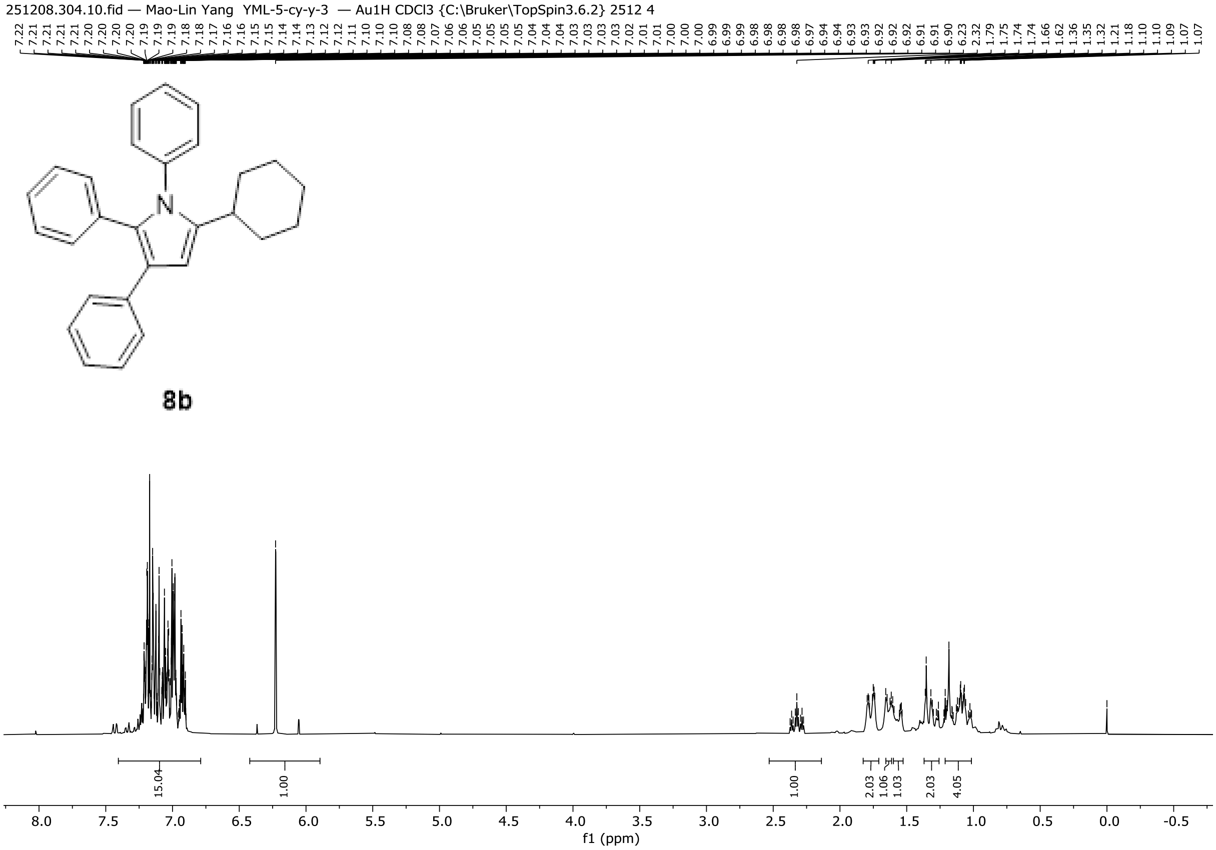

8b ^{13}C NMR (75 MHz, $CDCl_3$)

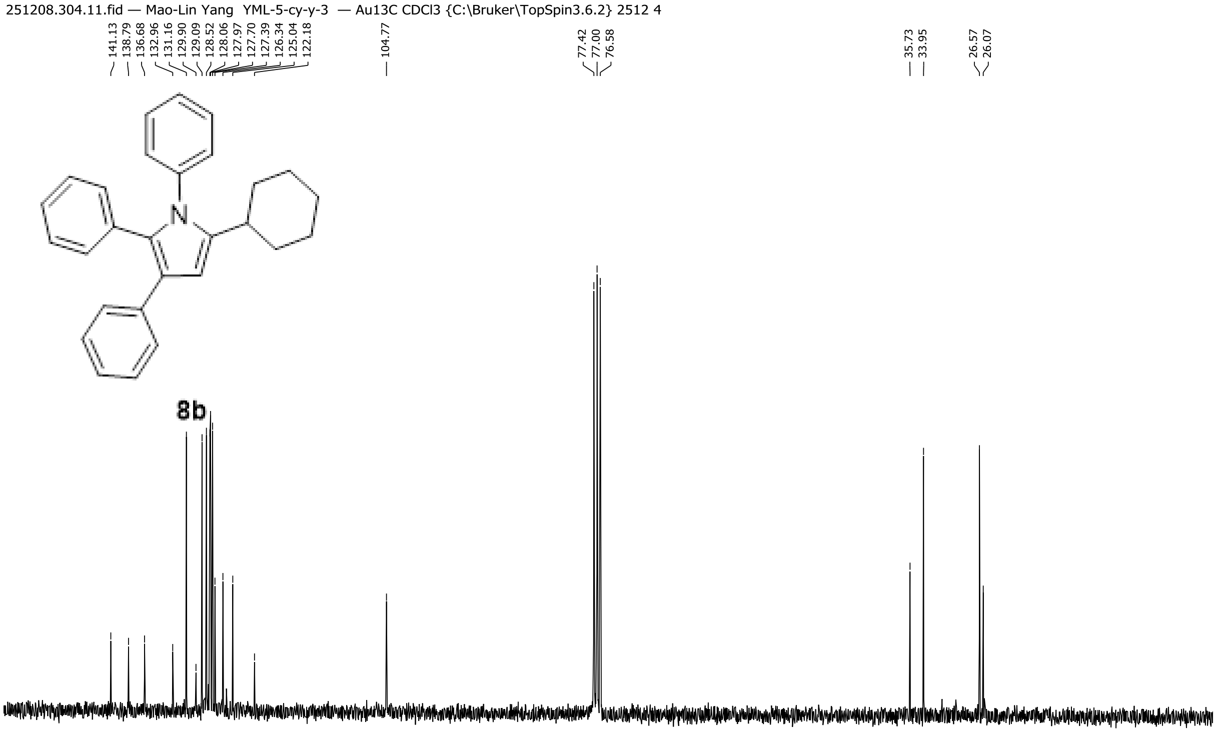

8b' ^{1}H NMR (300 MHz, $CDCl_3$)

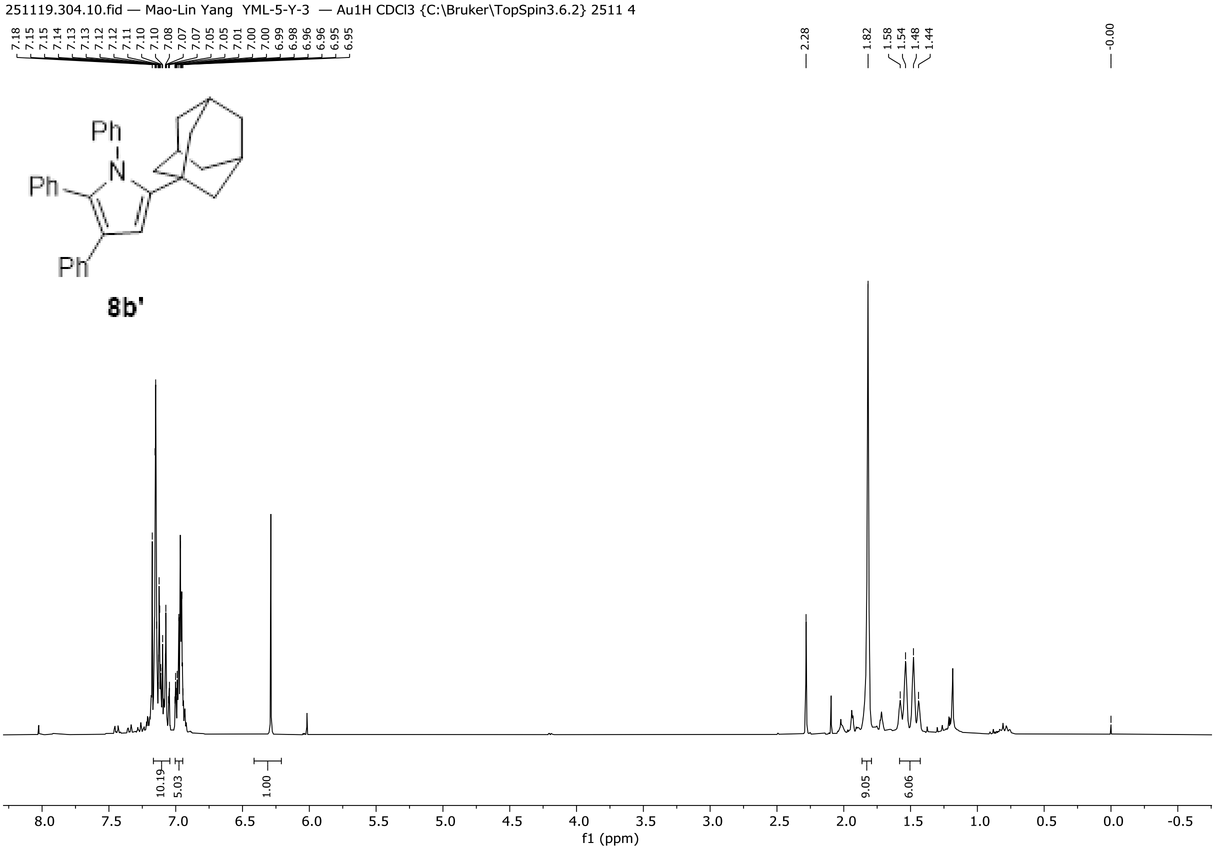

8b' ^{13}C NMR (75 MHz, $CDCl_3$)

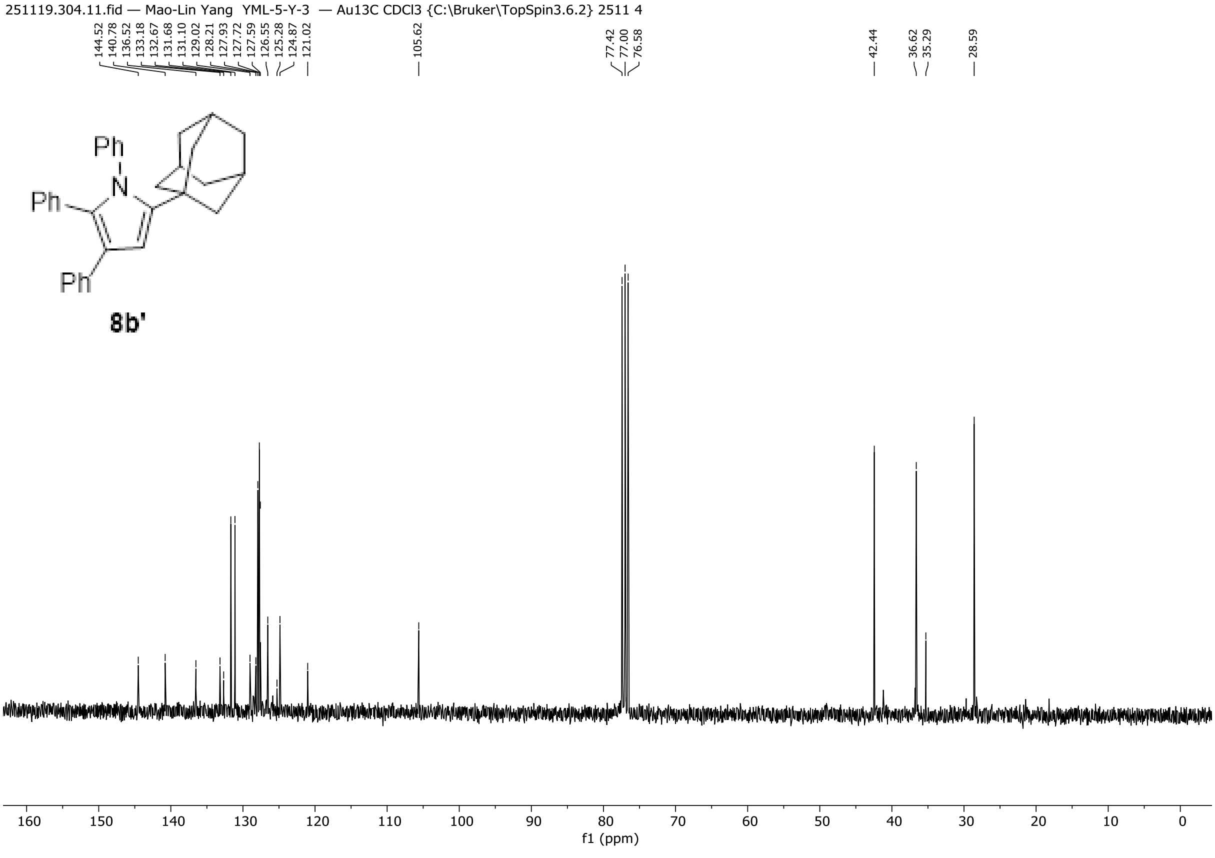

8c ^{1}H NMR (300 MHz, $CDCl_3$)

251207.336.10.fid — Mao-Lin yang, YML-5-cy-y-2 — Au1H CDCl3 {C:\Bruker\TopSpin3.6.2} 2512 36

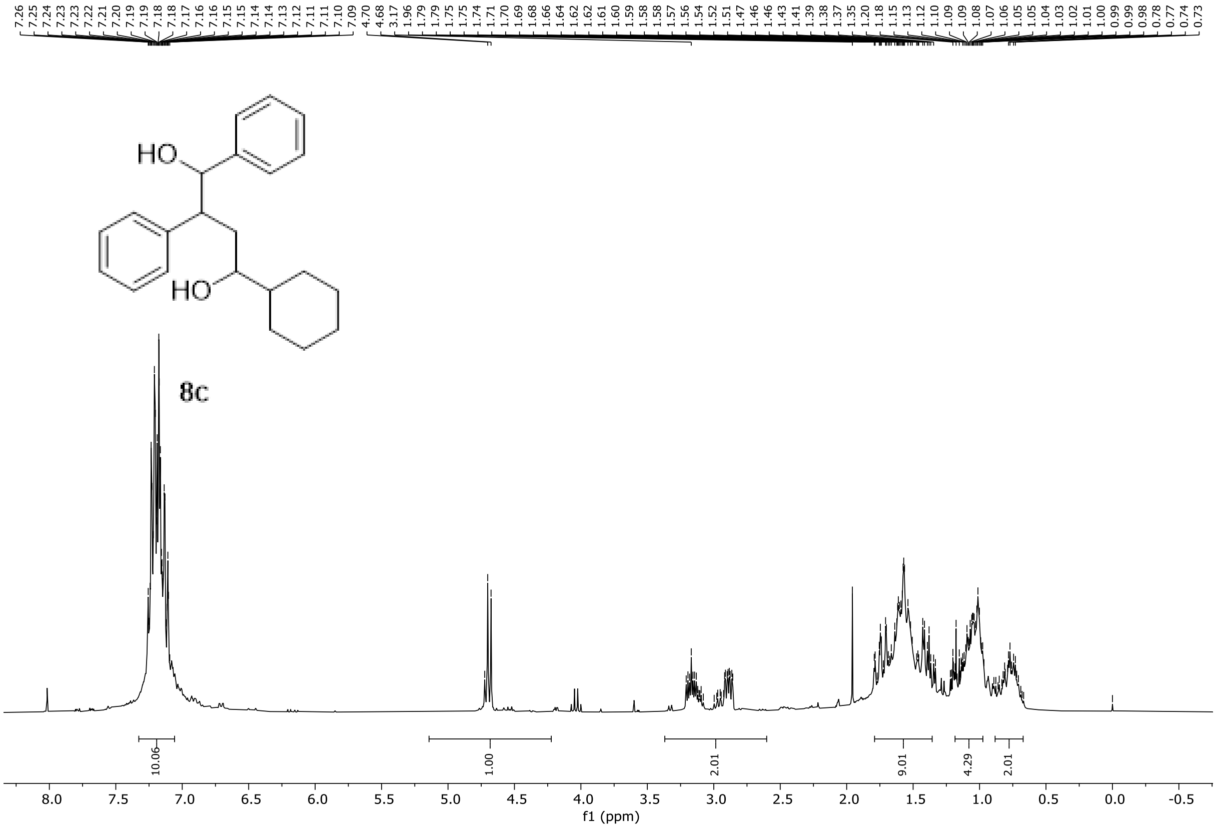

8c ^{13}C NMR (75 MHz, $CDCl_3$)

251207.336.11.fid — Mao-Lin yang, YML-5-cy-y-2 — Au13C CDCl3 {C:\Bruker\TopSpin3.6.2} 2512 36

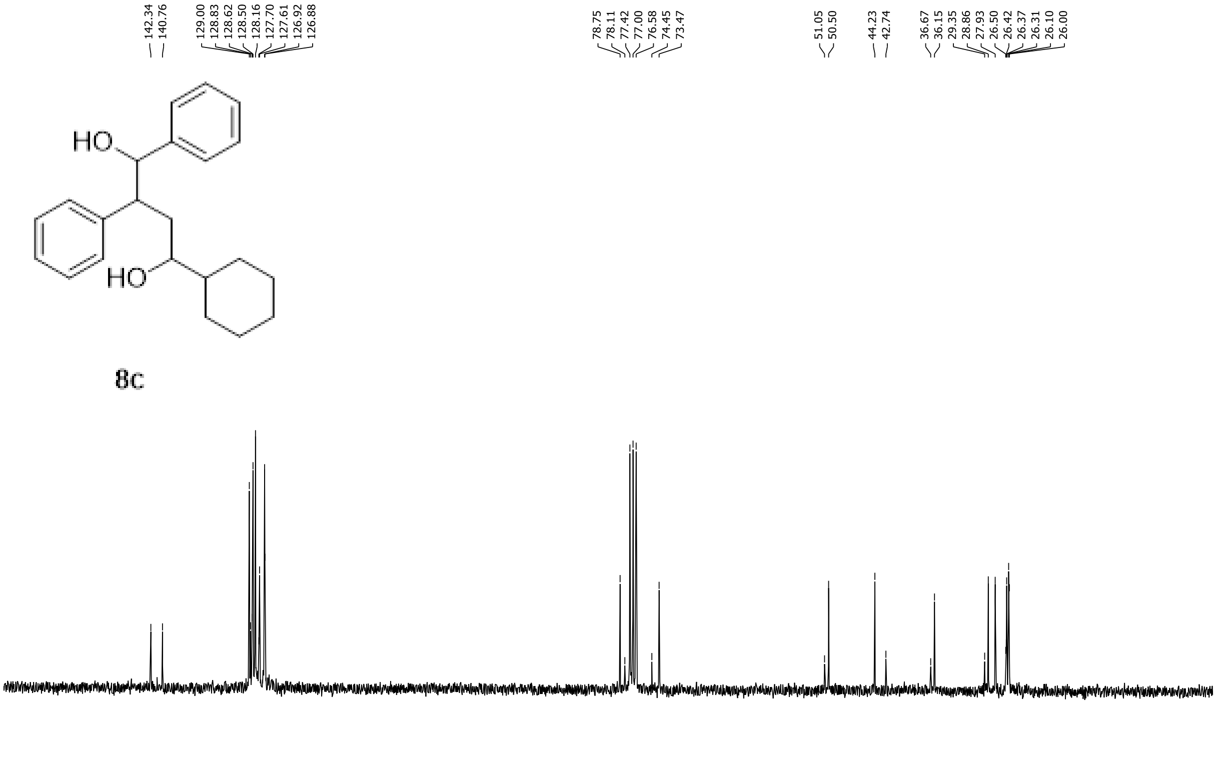

8d ¹H NMR (300 MHz, CDCl₃)

251208.306.10.fid — Mao-Lin Yang YML-5-cy-y-5 — Au1H CDCl3 {C:\Bruker\TopSpin3.6.2} 2512 6

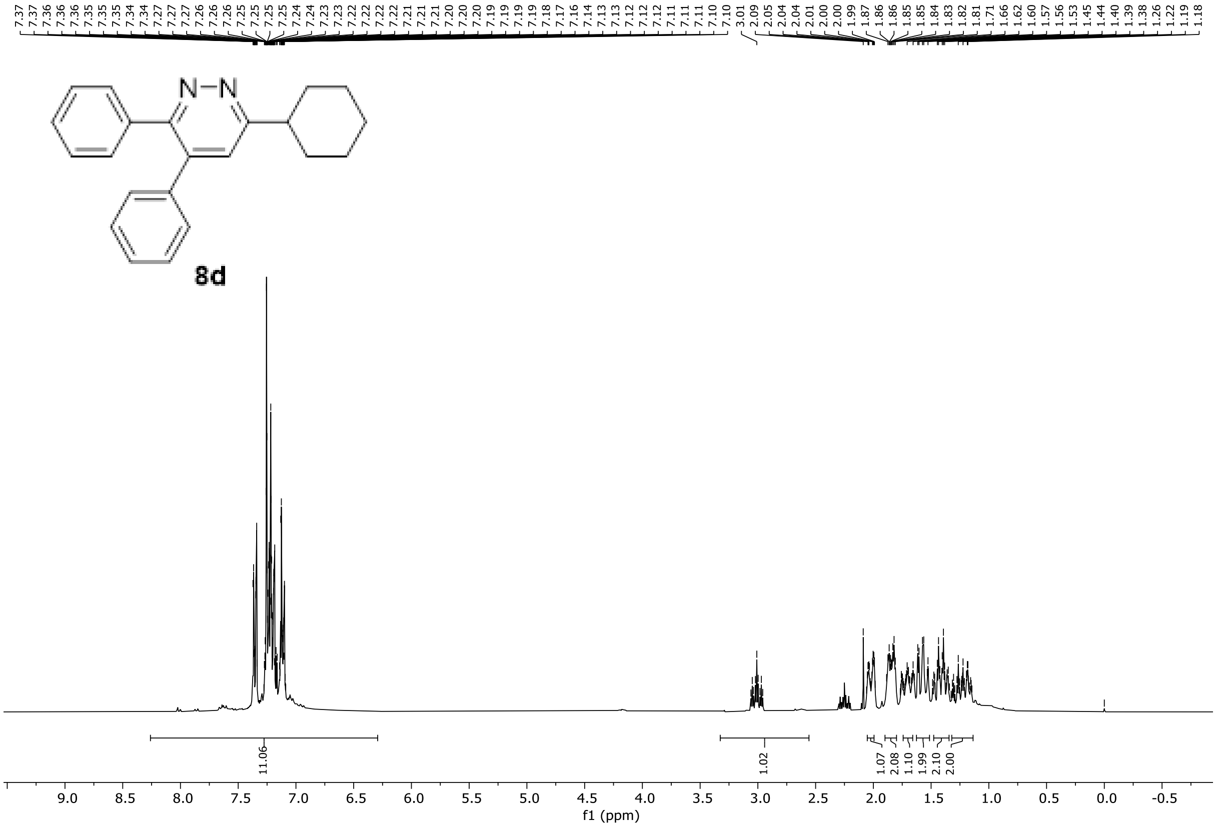

8d ¹³C NMR (75 MHz, CDCl₃)

251208.306.11.fid — Mao-Lin Yang YML-5-cy-y-5 — Au13C CDCl3 {C:\Bruker\TopSpin3.6.2} 2512 6

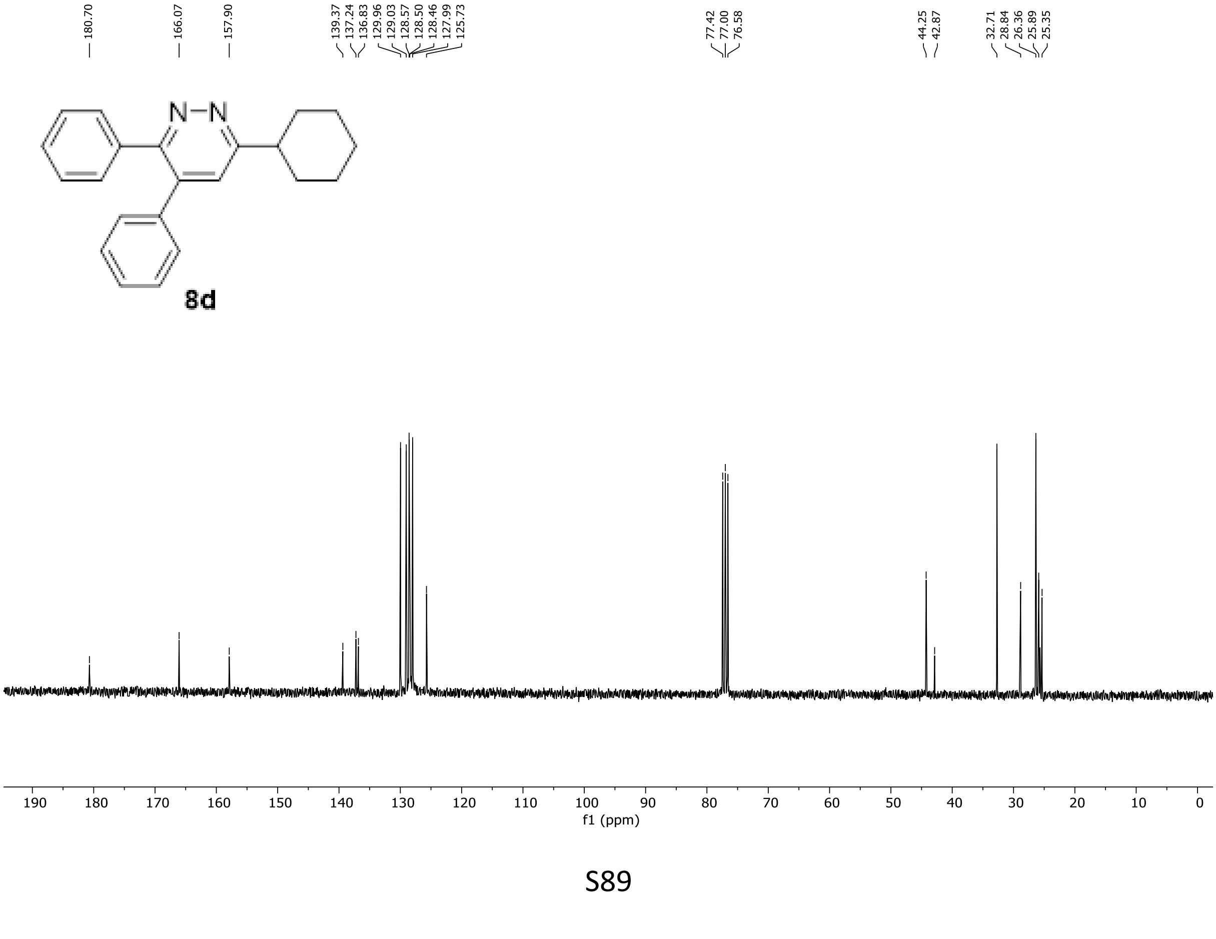

8e ¹H NMR (300 MHz, $CDCl_3$)

251208.305.10.fid — Mao-Lin Yang YML-5-cy-y-4 — Au1H CDCl3 {C:\Bruker\TopSpin3.6.2} 2512 5

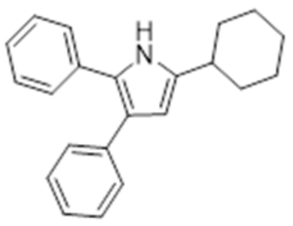

8e

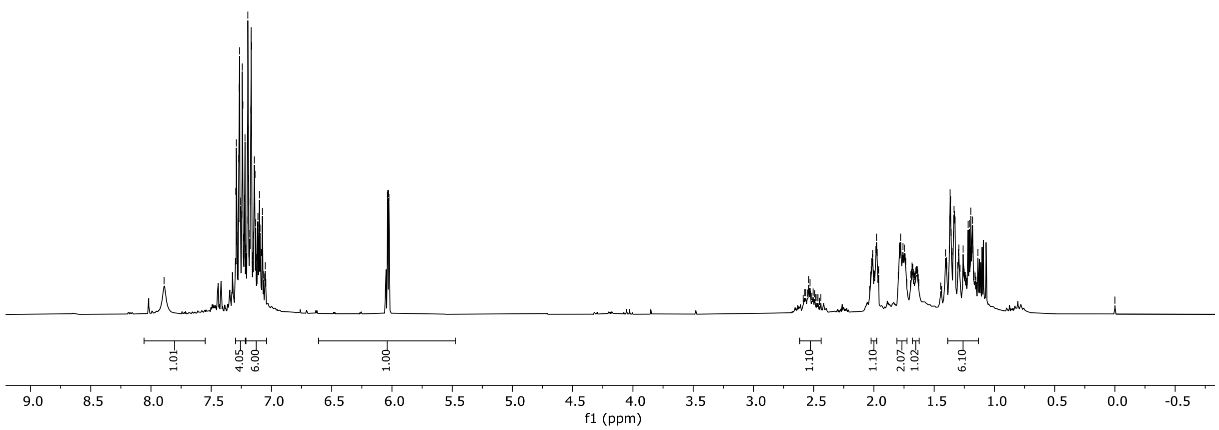

8e ¹³C NMR (75 MHz, $CDCl_3$)

251208.305.11.fid — Mao-Lin Yang YML-5-cy-y-4 — Au13C CDCl3 {C:\Bruker\TopSpin3.6.2} 2512 5

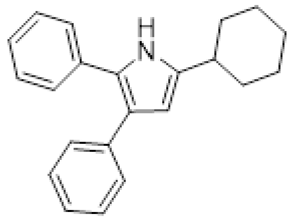

8e

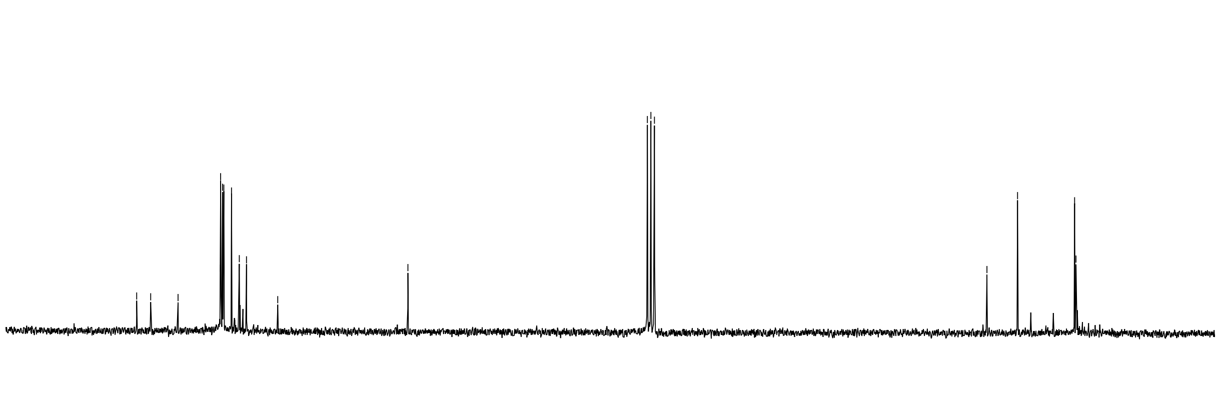

8e' ^{1}H NMR (300 MHz, $CDCl_3$)

251215.321.10.fid — Mao-Lin Yang, YML-jin-y-4-1 — Au1H CDCl3 {C:\Bruker\TopSpin3.6.2} 2512 21

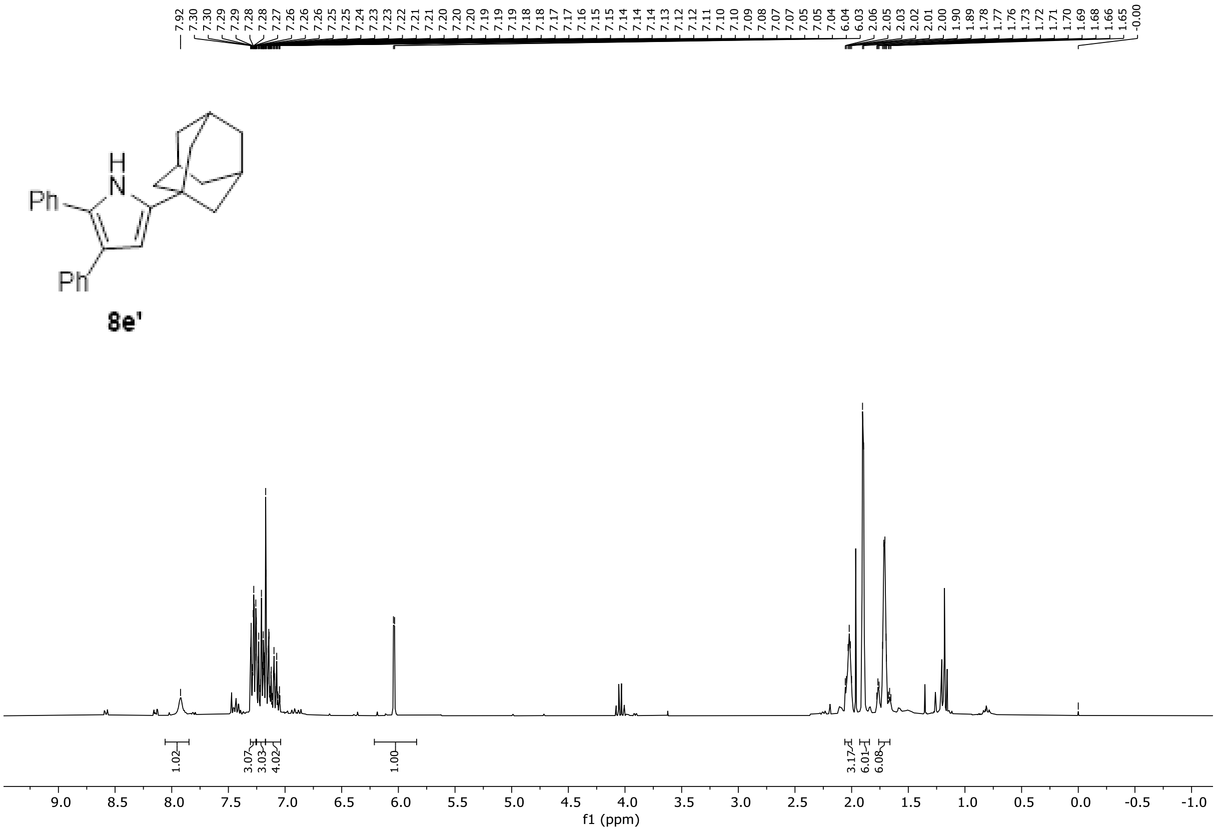

8e' ^{13}C NMR (75 MHz, $CDCl_3$)

251215.321.11.fid — Mao-Lin Yang, YML-jin-y-4-1 — Au13C CDCl3 {C:\Bruker\TopSpin3.6.2} 2512 21

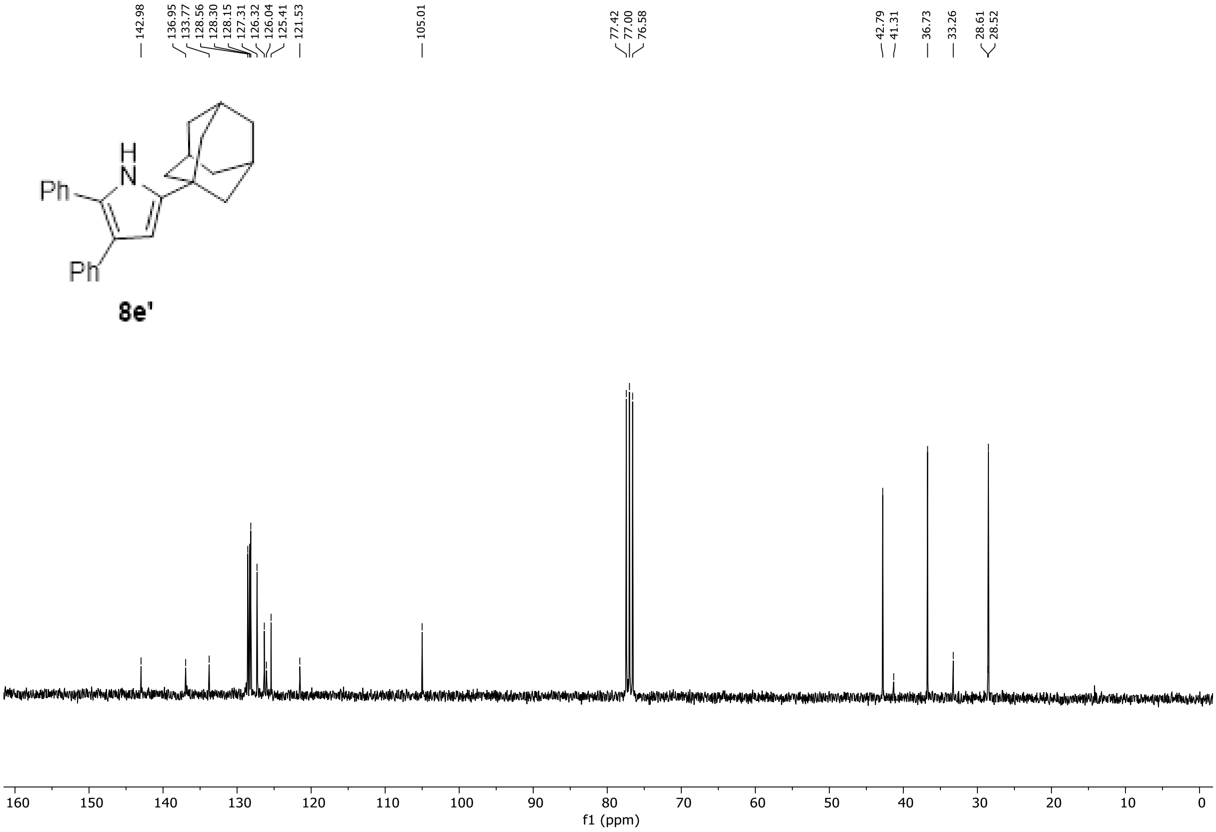

Supplement: SC-017-D5SC09978A-s001 [file SC-017-D5SC09978A-s001.pdf]
